# Cationic Phosphinidene as a Versatile P_1_ Building Block: [L_C_–P]^+^ Transfer from Phosphonio–Phosphanides [L_C_–P–PR_3_]^+^ and Subsequent L_C_ Replacement Reactions (L_C_ = N-Heterocyclic Carbene)

**DOI:** 10.1021/jacs.3c02256

**Published:** 2023-04-27

**Authors:** Philipp Royla, Kai Schwedtmann, Zeyu Han, Jannis Fidelius, Derek P. Gates, Rosa M. Gomila, Antonio Frontera, Jan J. Weigand

**Affiliations:** † Chair of Inorganic Molecular Chemistry, Faculty of Chemistry and Food Chemistry, Technische Universität Dresden, 01069 Dresden, Germany; ‡ Department of Chemistry, University of British Columbia, 2036 Main Mall, V6T 1Z1 Vancouver, Canada; § Department of Chemistry, Universitat de Illes Balears, 07122 Palma de Mallorca, Spain

## Abstract

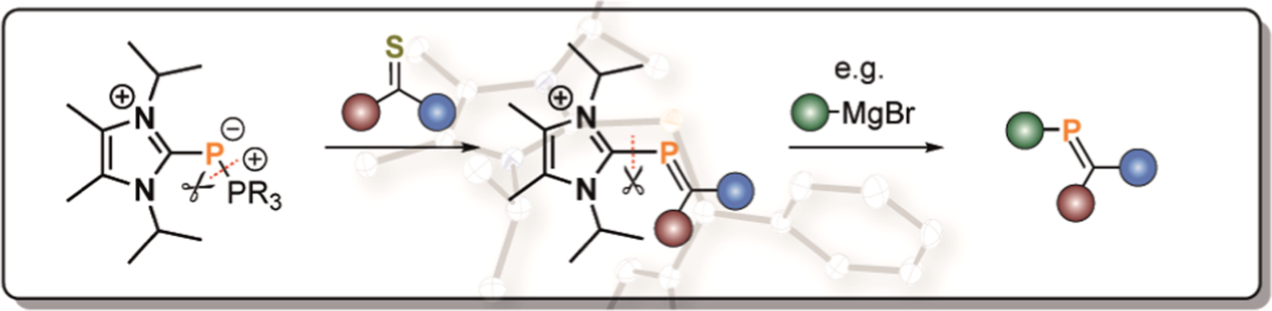

Cationic imidazoliumyl(phosphonio)-phosphanides [L_C_–P–PR_3_]^+^ (**1a–e**^**+**^, L_C_ = 4,5-dimethyl-1,3-diisopropylimidazolium-2-yl; R = alkyl, aryl) are obtained via the nucleophilic fragmentation of tetracationic tetraphosphetane [(L_C_–P)_4_][OTf]_4_ (**2**[OTf]_4_) with tertiary phosphanes. They act as [L_C_–P]^+^ transfer reagents in phospha-Wittig-type reactions, when converted with various thiocarbonyls, giving unprecedented cationic phosphaalkenes [L_C_–P=CR_2_]^+^ (**5a-f**[OTf]) or phosphanides [L_C_–P–CR(NR_2_^′^)]^+^ (**6a-d**[OTf]). Theoretical calculations suggest that three-membered cyclic thiophosphiranes are crucial intermediates of this reaction. To test this hypothesis, treatment of [L_C_–P–PPh_3_]^+^ with phosphaalkenes, that are isolobal to thioketones, permits the isolation of diphosphirane salts **11a,b**[OTf]. Furthermore, preliminary studies suggest that the cationic phosphaalkene [L_C_–P=CPh_2_]^+^ may be employed to access rare examples of η^2^–P=C π-complexes with Pd^0^ and Pt^0^ when treated with [Pd(PPh_3_)_4_] and [Pt(PPh_3_)_3_] for which analogous complexes of neutral phosphaalkenes are scarce. The versatility of [L_C_–P]^+^ as a valuable P_1_ building block was showcased in substitution reactions of the transferred L_C_-substituent using nucleophiles. This is demonstrated through the reactions of **5a**[OTf] and **6c**[OTf] with Grignard reagents and KNPh_2_, providing a convenient, high-yielding access to MesP=CPh_2_ (**16**) and otherwise difficult-to-synthesize 1,3-diphosphetane **17** and P-aminophosphaalkenes.

## Introduction

Simple phosphorus-containing functionalities can represent important tools for the construction of novel structural architectures with application in areas such as catalysis, polymers, and materials. For instance, phosphinidenes [R–P] are considered valuable and simple P_1_ building blocks for the synthesis of organophosphorus substrates or as diverse ligands in transition-metal complexes.^1^ Despite their synthetic utility and fundamental curiosity, phosphinidenes display exceedingly high reactivity, and the first isolable “free” phosphinidene, reported in 2016, remains the only example.^2^ Thus, researchers have designed a variety of more applicable precursors, phosphinidenoids, which can be employed in phosphinidene transfer reactions. Of particular importance were the early investigations of transition-metal-supported phosphinidenoid reagents as [R–P] building blocks, thereby affording otherwise difficult to access organophosphorus compounds.^1,3^ In a few cases, the release of [R–P] from metal-free precursors has also been described, for example, from so-called inversely polarized phosphaalkenes R–P^δ−^=C^δ+^R_2_.^4^

**Figure 1:**
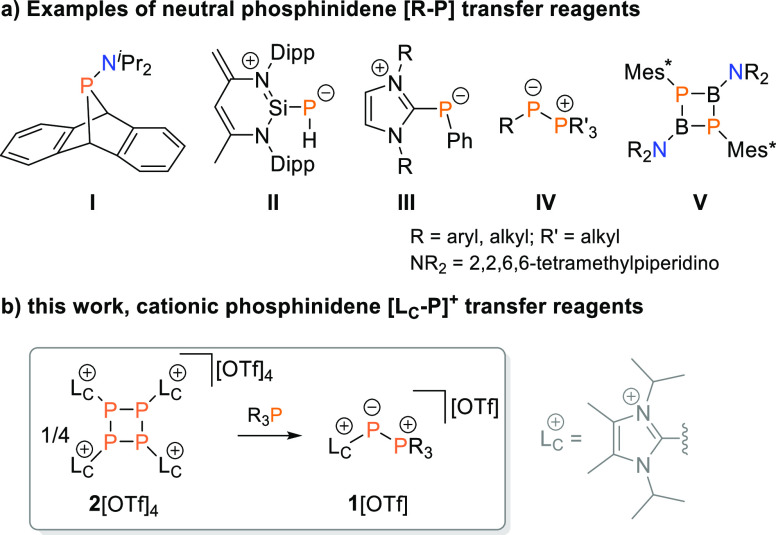
(a) Examples of neutral phosphinidene [R–P] transfer reagents; (b) synthesis of cationic [L_C_–P]^+^ transfer reagents reported here. Mes* = 2,4,6-^*t*^BuC_6_H_2_, Dipp = 2,6-^*i*^Pr_2_C_6_H_3_.

More recently, a new generation of phosphinidene chemistry has evolved with exciting breakthroughs involving isolable, neutral, and metal-free singlet [R–P] transfer reagents that are tolerable of a variety of substituents (Figure 1a).

For instance, amino-phosphinidene, [R_2_N–P], transfer has been enabled from precursor **I** with concomitant formation of anthracene.^5^ Importantly, the first transfers of the parent phosphinidene, [H–P], were observed from **II**.^6^ Carbene-phosphinidene adducts **III** and phosphanylidenephosphoranes **IV** (or “phospha-Wittig reagents”^7^) have been shown to transfer aryl- and alkyl-substituted phosphinidenes to a wide range of substrates [e.g., organic electrophiles,^8^ aldehydes,^9^ NHCs (N-heterocyclic carbenes),^10^ isonitriles,^11^ ammonia,^12^ and Al^I^ species.^13^ Diphosphadiboretane **V** has been utilized as a [Mes*–P] transfer agent to ketones, amides, and esters in the unprecedented phospha-bora-Wittig reaction.^14^ Despite advances in the field, the development of a single phosphinidene transfer reagent capable of transferring phosphinidenes [R–P] with a multitude of different substituents R is still desired. In a recent study, we demonstrated the versatility of cationically substituted phosphorus compounds for the formation of P–C, P–N, and P–O bonds by easily replacing the cationic substituent using commercially available reagents.^15^ This inspired us to explore the potential of employing cationic substituents for phosphinidenes to create unprecedented cationic phosphinidene transfer reagents, namely, [L_C_–P]^+^ (Figure 1b). This could enable further functionalization at the P atom after the transfer reaction and render [L_C_–P]^+^ a versatile P_1_ building block.

**Scheme 1:**
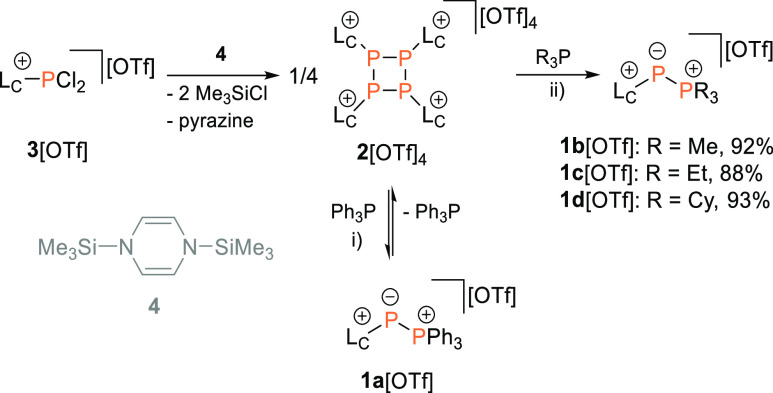
Synthesis of 2[OTf]_4_ and Its Nucleophilic Fragmentation with Tertiary Phosphanes R_3_P (R = Me, Et, Cy, and Ph) Reagents and conditions: (i) +4 Ph_3_P, CD_3_CN, rt, 16 h; (ii) +4 R_3_P, CH_3_CN, rt, 4–16 h, 88–93%.

We recently discovered that the tetracationic tetraphosphetane **2**^4**+**^ (Figure 1), formally a tetramer of [L_C_–P]^+^, may be conveniently obtained in good yields (86%) as its triflate salt from the reduction of **3**[OTf] with 1,4-bis(trimethylsilyl)-1,4-dihydropyrazine (**4**, Scheme 1).^16^ Computational studies on **2**^4**+**^ suggest a high electrophilicity due to the four imidazoliumyl substituents. We therefore hypothesized that nucleophilic cleavage with tertiary phosphanes R_3_P might provide suitable access to phosphonio–phosphanides **1**^**+**^ as potential [L_C_–P]^+^ transfer reagents.

We now report a straightforward route to simple [L_C_–P]^+^ transfer agents (**1a-d**^**+**^) from readily available starting reagents. Their utility is demonstrated by cationic phosphinidene transfer to substrates, including thioketones, thioamides, thiourea, thioesters, and phosphaalkenes R–P^δ+^=C^δ−^R_2_. Unprecedented phosphonio–phosphanides, **1b-d**^**+**^, have been characterized crystallographically as triflate salts, along with a series of hitherto unknown cationic phosphaalkenes, phosphanides, diphosphiranes, and metal complexes, including very rare η^2^–P=C–Pd^0^ and Pt^0^ complexes. In addition, we demonstrate the ability to perform substitution reactions of the transferred L_C_-substituent in selected substrates using widely applied nucleophilic aryl and alkyl Grignard reagents RMgBr (R = Mes, Me), as well as amide KNPh_2_. This results in the formation of differently P-functionalized organophosphorus compounds.

## Results and Discussion

### Preparation of Phosphonio–Phosphanides

**Figure 2:**
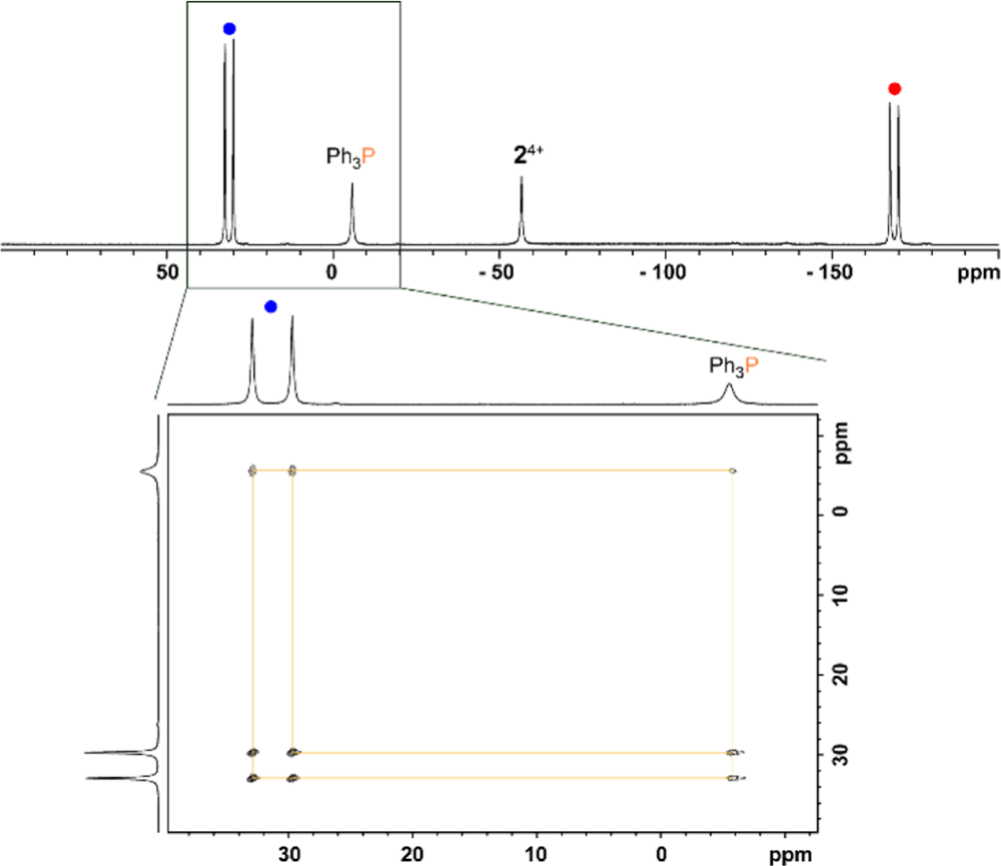
^31^P NMR spectrum of an aliquot of the reaction mixture of **2**[OTf]_4_ with four equivalents of Ph_3_P in CD_3_CN after 16 h (top, CD_3_CN, 300 K) and zoom in of a ^31^P–^31^P-EXSY NMR spectrum (bottom, CD_3_CN, 300 K) displaying spin polarization exchange between the phosphonio moiety in **1a**^**+**^ and Ph_3_P.

Upon adapting our published synthesis of **2**[OTf]_4_ to a larger scale (ca. 50 g, see Supporting Information S2.1), we opted to investigate the reaction of **2**[OTf]_4_ with Ph_3_P (Scheme 1). Thus, isolated **2**[OTf]_4_ was treated with Ph_3_P (4 equiv.) in CD_3_CN. Subsequent analysis of an aliquot removed from the reaction mixture revealed a new AX spin system [δ(^31^P_A_) = −168.6 ppm, δ(^31^P_X_) = 31.3 ppm, ^1^*J*(PP) = −519 Hz] in its ^31^P NMR spectrum assigned to phosphonio-phosphanide **1a**^**+**^ (Figure 2). In addition, the spectrum showed signals assigned to the starting materials suggestive of equilibrium. In comparison with phosphanylidenephosphorane DmpP–PPh_3_ (Dmp = 2,6-Mes_2_C_6_H_3_, Table 1),^17^ the phosphanide (P_A_) moiety in **1a**^**+**^ is further upfield, and the magnitude of ^1^*J*(PP) is significantly lower. In related triphosphenium cations {e.g., [(Ph_3_P)_2_P]^+^}^18^ the high field chemical shift and smaller coupling constant have been attributed to the dominance of the bis(ylidic) canonical structure.^19^ Further investigation of the reaction mixture by means of ^31^P–^31^P EXSY NMR experiments confirmed the underlying thermodynamic equilibrium (Figure 2). Notably, nucleophilic fragmentation of pentaphospholane (PhP)_5_^4a,20^ and the more electrophilic tetraphosphetane [(CF_3_)P)_4_]^21^ has been described previously, although stronger nucleophiles, that is, NHCs or Me_3_P, respectively, are required.

**Table 1:** Comparison of ^31^P NMR Chemical Shifts and Coupling Constants in **1a-e**^**+**^ and Selected Related Compounds^9,17,23^

compound	P_A_ (in ppm)	P_X_ (in ppm)	^1^*J*(PP) (in Hz)
**1a**[OTf] (R = Ph)	–168.6	31.3	–519.0
**1b**[OTf] (R = Me)	–167.0	12.0	–472.0
**1c**[OTf] (R = Et)	–202.0	36.0	–492.0
**1d**[OTf] (R = Cy)	–208.8	38.1	–545.0
**1e**[OTf] (R = Ph_2_(CH_2_PPh_2_))	–164.3	38.1	–519.0
DmpP–PPh_3_^17^	–138.8	25.2	–639.0
DmpP–PMe_3_^9^	–114.7	–2.8	–582.0
[(Ph_3_P)_2_P][AlCl_4_]^23^	–174.0	30.0	–502.0

**Figure 3:**
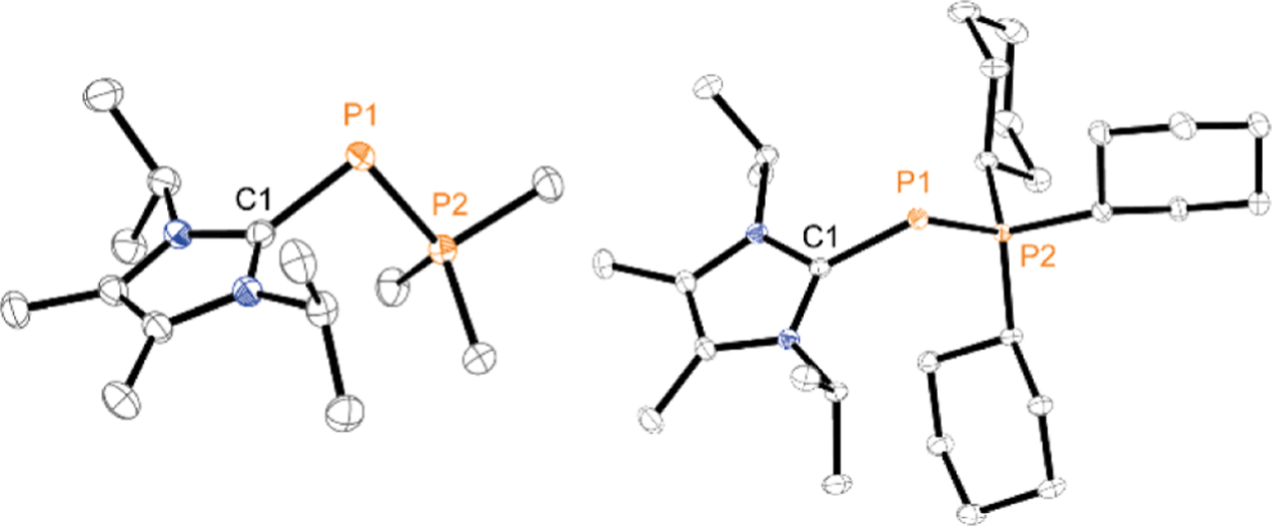
Molecular structures of phosphonio–phosphanides **1b**,**d**^**+**^ in **1b**,**d**[OTf]; hydrogen atoms and anions are omitted for clarity, and thermal ellipsoids are displayed at 50% probability; selected bond lengths (Å) and angles(°): for **1b**^**+**^: P1–P2 2.1162(4), P1–C1 1.8306(13), C1–P1–P2 97.98(4); **1c**^**+**^ (Supporting Information, Figure S14): P1–P2 2.1195(4), P1–C1 1.8299(11), C1–P1–P2 99.29(4); **1d**^**+**^: P1–P2 2.1446(4), P1–C1 1.8280(11), C1–P1–P2 105.93(4).

In an effort to prepare isolable phosphonio–phosphanides, the reaction of **2**[OTf]_4_ with more nucleophilic trialkyl-substituted tertiary phosphanes (R_3_P: R = Me, Et, and Cy) was conducted in CH_3_CN solution. The complete formation of the corresponding phosphonio–phosphanides **1b-d**^**+**^ was observed after 4–16 h, and they could be isolated by precipitation with Et_2_O in excellent yields as their triflate salts (88–92%, Scheme 1). Their respective ^31^P NMR spectra show the expected characteristic AX spin systems (see Table 1), in accordance with reported values for the related phosphanylidenephosphoranes ArP–PMe_3_^9,10^ and with the expected group contribution effects.^18,19,22^ Alternatively, **1b-d**[OTf] can be synthesized directly from the reduction of **3**[OTf] using an excess of R_3_P (Scheme S2, Figure S6); however, isolation is best achieved using the procedure described above. Vapor diffusion of Et_2_O into saturated CH_3_CN solutions of **1b-d**[OTf] at −30 °C afforded colorless crystals suitable for single crystal X-ray analysis. The molecular structures of **1b**[OTf] and **1d**[OTf] are shown in Figure 3 and that of **1c**[OTf] is shown in the Figure S14.

The observed P–P bond lengths [for **1b**^**+**^: P1–P2 2.1162(4) Å, **1c**^**+**^: P1–P2 2.1195(4) Å, and **1d**^**+**^: P1–P2 2.1446(4) Å] match values for related triphosphenium cations^19,23^ and range between a typical P–P single^24^ and P=P double bond.^25^ This shortening has previously been attributed to result from ylidic-type negative hyperconjugation between the lone pairs at the phosphanide moiety and the σ*(P–R) orbitals.^19^ Phosphonio–phosphanides **1b-d**[OTf] can be stored indefinitely under an inert atmosphere at ambient temperature, whereas neutral derivatives of phosphanylidenephosphoranes ArP–PMe_3_ have a tendency to decompose with respect to the formation of (ArP)_*n*_ (*n* = 2,3) under concomitant release of PMe_3_.^9,26^ In an effort to rationalize this apparent high stability, density functional theory (DFT) calculations were performed on the **1a**^**+**^, **1b**^**+**^**,** and **1c**^**+**^ cations using CH_3_CN as solvent (details are provided in the Supporting Information). As the energy of the HOMO–LUMO gap in **1a**^**+**^ (*E*_gap_ = 2.472 eV; **1b**^**+**^: R = Me: *E*_gap_ = 2.791 eV; **1c**^**+**^: R = Et: *E*_gap_ = 2.760 eV) is still slightly higher than in DmpP–PMe_3_ (*E*_gap_ = 2.443 eV), even larger HOMO–LUMO gaps can be achieved through the introduction of alkyl substituents at the phosphonio moiety (Table S9, Figure S124).

Notably, reaction of **2**[OTf]_4_ with ditopic phosphane 1,1-bis(diphenylphosphino)methane (dppm) leads to the formation of **1e**[OTf] (Table 1) instead of the corresponding bis(phosphonio-phosphanide). Changing the ditopic phosphane to bis(diphenylphosphino)ethane (dppe) gives rise to a mixture of previously reported cyclic triphosphenium cation [(Ph_2_PC_2_H_4_PPh_2_)P]^+^ and di(imidazoliumyl)phosphanide [(L_C_)_2_P]^+^ as evidenced by means of ^31^P NMR spectroscopy (Scheme S3 and Figure S8).^18,23,27^

### Phosphonio–Phosphanides as Cationic Phosphinidene Transfer Agents

We continued to investigated the ability of compounds **1a-d**[OTf] to transfer [L_C_–P]^+^ in phospha-Wittig-type reactions. An initial effort to treat **1b[**OTf] with 4-methoxybenzaldehyde resulted in encouraging ^31^P NMR spectra (see Supporting Information S2.14) of the reaction mixture, showing a small downfield signal at 178.3 ppm along with resonances assigned to free Me_3_P (δ = −61.5 ppm) and Me_3_PO (δ = 36.2 ppm). We speculated that the downfield signal observed was consistent with that anticipated for an unprecedented cationic phosphaalkene {i.e., [L_C_P=CH(C_6_H_4_OMe)]^+^}. However, the conversion to phosphaalkene was very low (<5%), thus we turned our attention to more reactive thiocarbonyls. The latter can typically be accessed directly by thionation of the respective ketone, for example, via conversion with H_2_S, P_4_S_10_, or Lawesson’s reagent.^28^

**Figure 4:**
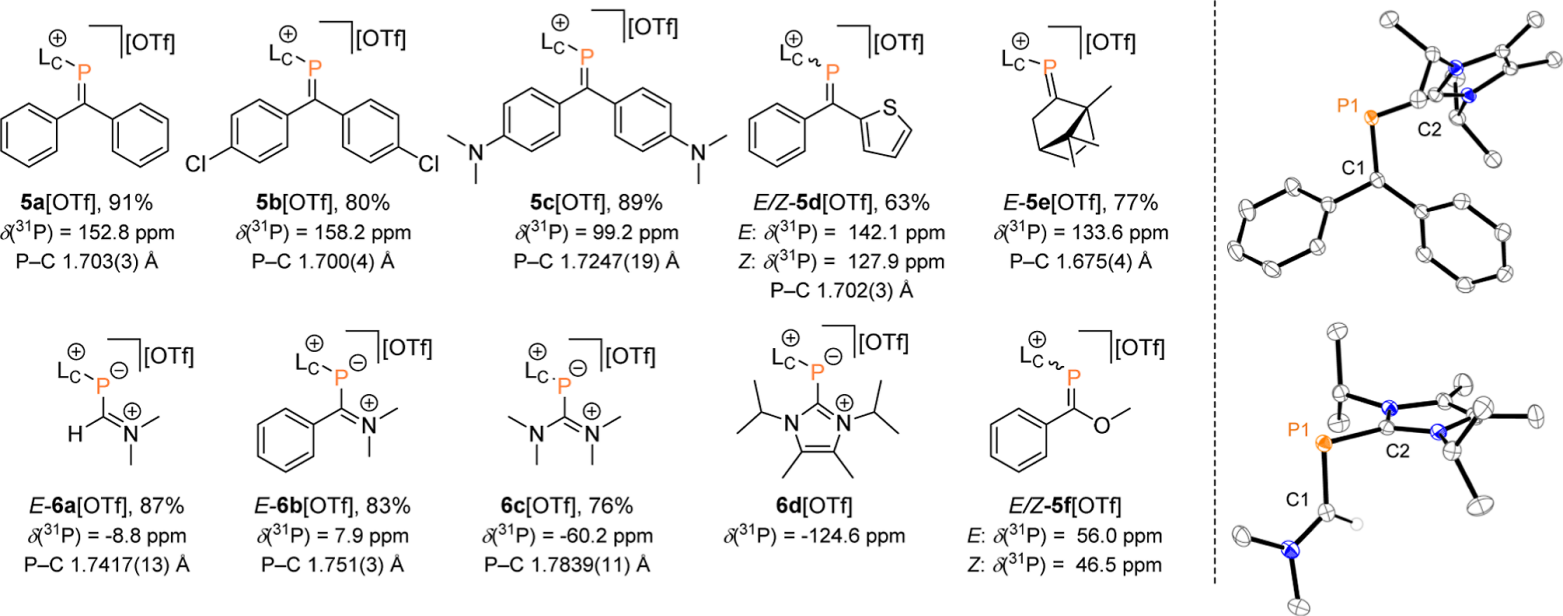
Synthesized phosphaalkenes **5a-f**[OTf] and phosphanides **6a-d**[OTf] (left); molecular structure of phosphaalkenes **5a**^**+**^ in **5a**[OTf] and phosphanide **6a**^**+**^ in **6a**[OTf] (right); hydrogen atoms and anions are omitted for clarity, and thermal ellipsoids are displayed at 50% probability; selected bond lengths (Å) and angles (°): for **5a**^**+**^: P1–C1 1.707(3), P1–C2 1.834(33), C1–P1–C2 104.55(16); **6a**^**+**^: P1–C1 1.7417(13), P1–C2 1.8382(12), C1–P1–C2 93.77(6).

**Scheme 2:**
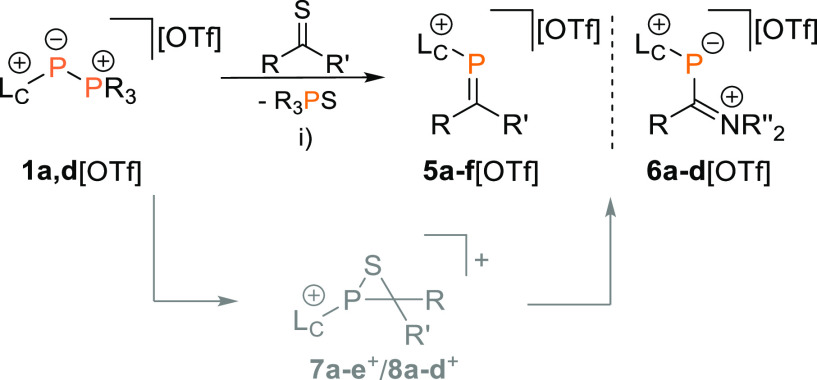
Reactions of **1a**,**d**^**+**^ with Thiocarbonyls Yield Phosphaalkenes **5a-f**[OTf] (R = Aryl, Alkyl; R′ = Aryl, Alkyl, OMe) or Phosphanides **6a-d**[OTf] (R = H, Aryl, NR_2_^″^; R′ = NR_2_^″^) Reagents and conditions: (i) for **5a-d**[OTf] and **6a**,**b**[OTf]: −R_3_PS, CH_3_CN, rt, 16 h, 63–91%; for **5e**[OTf] and **6c**,**d**[OTf]: −R_3_PS, CH_3_CN, 80 °C, 3 h, 76–77%; **5a**,**d**,**e**[OTf] and **6a-d**[OTf] were prepared using *in situ* generated **1a**^**+**^, **5b-c**[OTf] were prepared using isolated **1d**[OTf].

For our following studies, compound **1a**^**+**^ was selected to investigate its [L_C_–P]^+^ transfer capability, as it holds the greatest synthetic value compared with **1b**-**d**[OTf], owing to its ease of handling and the comparatively low cost of its starting material PPh_3_ compared with the other alkyl-substituted tertiary phosphines. When treated with equimolar amounts of selected thioketones in CH_3_CN, *in situ* generated **1a**^**+**^ completely converts into the respective phosphane sulfide R_3_PS and cationic phosphaalkenes **5a-e**^**+**^ within 16 h at room temperature (Scheme 2), as evidenced by ^31^P NMR spectroscopy. The resonances of **5a-e**[OTf] in CD_3_CN (Figure 4) are significantly upfield shifted relative to neutral phosphaalkenes [e.g., MesP=CPh_2_: δ(^31^P) = 233 ppm].^29^ For heteroleptic **5d**[OTf], both configurational diastereomers (*E*/*Z*) are observed in a near 1:1 ratio. The formation of (+)-camphor-derived **5e**[OTf] requires heating of the reaction mixture to 80 °C for 3 h in a microwave reactor. The title compounds can be isolated as analytically pure solids as their triflate salts in very good to excellent yields by precipitation from the respective reaction mixture by addition of Et_2_O (63–91%, Figure 4).

A second set of cationic phosphinidene transfer reactions were explored by treating *in situ* generated **1a**^**+**^ with thioamides [R(NMe_2_)C=S (R = H, Ph, NMe_2_)] and L_C_=S. In each case, analysis of the reaction mixtures by means of ^31^P NMR spectroscopy showed only a signal assigned to Ph_3_PS (δ = 42.4 ppm) along with a new singlet resonance {δ(^31^P) = −8.8 ppm (br), R = H; 7.9 ppm, R = Ph; −60.2 ppm (br), R = NMe_2_; −124.6 ppm, cf. known [(L_C_)_2_P]^+^^30^}. Remarkably, each were shifted considerably upfield compared to those of **5a-e**^**+**^. A similar trend to higher field shifts is observed in the ^31^P NMR spectra of inversely polarized phosphaalkenes bearing C-amino substituents when compared to conventional phosphaalkenes.^4e,31^ Given this apparent higher shielding/increased electron density at the P atoms, the products were formulated with the cationic phosphanide canonical form (i.e., **6a-d**^**+**^ in Figure 4) rather than cationic phosphaalkene (cf. **5**^**+**^).

A supporting trend for this observation was found in the molecular structures of **5a**-**f**[OTf] and **6a**-**c**[OTf] (**5a**[OTf] and **6a**[OTf] in Figure 4; **5b**-**f**[OTf] and **6b**,**c**[OTf] in Supporting Information). The P=C bond length in cationic phosphaalkene **5a**[OTf] [P=C 1.707(3) Å, Figure 4] is only slightly elongated compared to the related MesP=CPh_2_ [P=C 1.692(3) Å].^32^ Likewise, the P=C bonds of **5b**,**d**^**+**^ [1.700(4), 1.702(4) Å, respectively] are in the range typical of phosphaalkenes. In contrast, the camphor-substituted **5e**^**+**^ has a shorter P=C bond length [1.675(4) Å], presumably due to reduced delocalization of the P=C bond. The introduction of donating amino groups leads to significant elongation of the P–C bond [**5c**^**+**^: 1.7247(19) Å, **6a**^**+**^: 1.7417(13) Å, **6c**^**+**^: 1.7838(11) Å], consistent with increased contribution of the phosphanide canonical form, and comparable to values for reported inversely polarized phosphaalkenes.^4e,8a,20,33^ Generally, the L_C_–P=C bond angles are more acute for those formulated as phosphanides [C1–P1–C2 (°) = 93.77(6), **6a**^**+**^; 99.30(15), **6b**^**+**^; 100.81(5), **6c**^**+**^] when compared to those formulated a phosphaalkenes [C1–P1–C2 (°) = 104.55(16), **5a**^**+**^; 104.68(19), **5b**^**+**^; 103.86(9), **5c**^**+**^; 104.56(16), **5d**^**+**^; 99.3(3), **5e**^**+**^]. The reaction with O-methyl benzothioate proceeds likewise but results in multiple products, as evidenced by the ^31^P NMR spectra of the reaction mixture (Figure S74). Single crystal analysis of some crystalline material obtained by vapor diffusion of Et_2_O into the reaction mixture, however, confirms the formation of methoxy-substituted phosphaalkene **5f**[OTf] [δ(^31^P) = 53.0 ppm, see Supporting Information S2.25].

**Figure 5:**
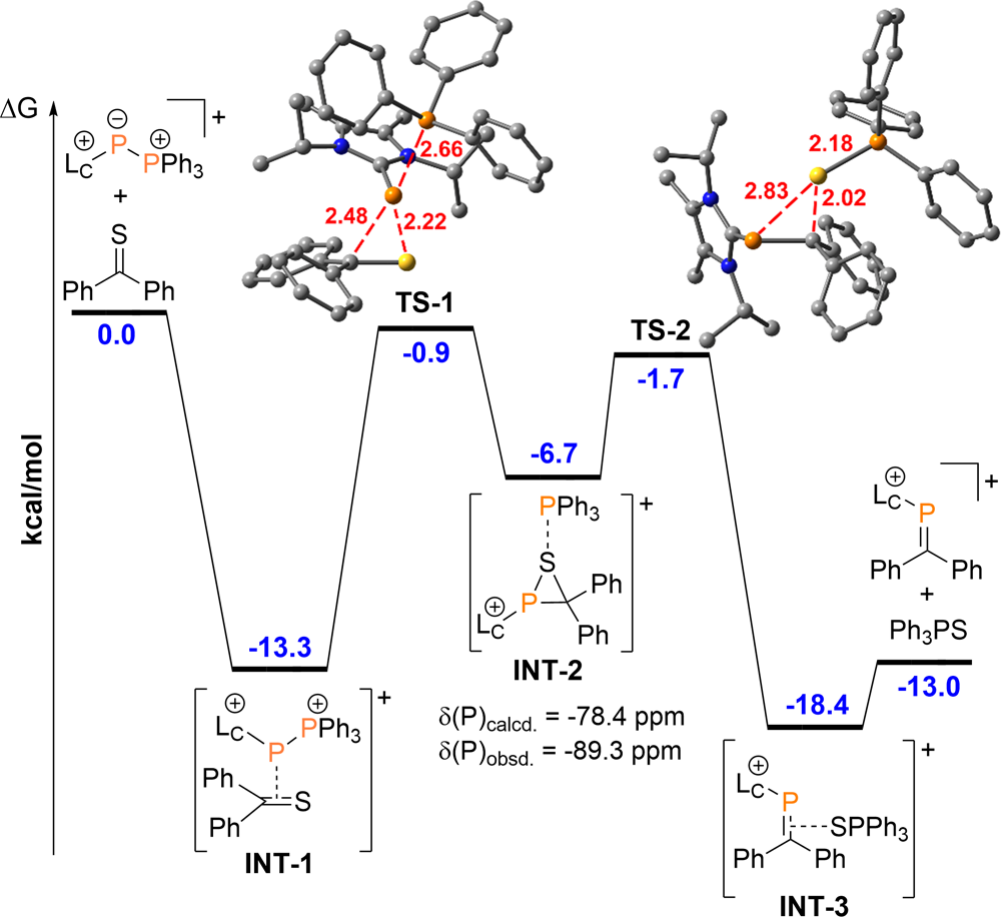
Reaction profile for the conversion of **1a**^**+**^ to **5a**^**+**^ at the RI-BP86-D3/def2-TZVP (acetonitrile) level of theory; optimized geometries of the transition states (TS) with distances (red) in Å. *Y* axis shows ΔG in kcal/mol.

In general, Wittig-type conversions are thought to proceed via open-chain betaine-type structures or four-membered oxaphosphetanes as intermediates.^14,34^ One report proposes an oxadiphosphetane intermediate for the related phospha-Wittig-Horner reaction.^35^ Next to this, detailed investigations on the mechanism of the phospha-Wittig reaction specifically are scarce. Therefore, we employed DFT calculations [RI-BP86-D3/def2-TZVP (acetonitrile)] to gain further insight into the reaction of **1a**^**+**^ with thiobenzophenone (Figure 5). The reaction profile reveals an overall exergonic transformation (−13.0 kcal/mol) that is initiated by the formation of a supramolecular complex **INT-1** (see Figure S126), which is 13.3 kcal/mol more stable than the isolated reactants and possibly results from interaction of the lone pairs at the phosphanide moiety of **1a**^**+**^ with the π*-orbital of the C=S double bond. This pre-organization is followed by a [L_C_–P]^+^ transfer onto the C=S double bond via **TS-1** with concomitant release of Ph_3_P to give the three-membered thiophosphirane^36^**INT-2**, which is more stable than **TS-1** by 5.8 kcal/mol. In an attempt to identify the formation of **INT-2** during the course of the reaction, we monitored the reaction between isolated **1d**[OTf] (in favor of **1a**^**+**^ to rule out possible [L_C_–P]^+^ transfer from **2**[OTf]_4_ present in the reaction mixture of *in situ* generated **1a**^**+**^) and thiobenzophenone by means of time-resolved ^31^P NMR spectroscopy. The spectrum of an aliquot of the reaction mixture after 10 min at room temperature reveals the formation of a new singlet resonance at δ(^31^P) = −89.3 ppm (Figure S1), which is within the margin of error^37^ for the calculated ^31^P NMR shift of **INT-2** [δ(^31^P) = −78.4 ppm]. We therefore assign this resonance to thiophosphirane **7a**^**+**^. Notably, the formation of thiophosphirane **7a**^**+**^ can also be observed in other transformations (see Figures S2, S4, and S7), as evidenced by its characteristic chemical shift in the ^31^P NMR spectra.

The formation of phosphanides **6a-d**^**+**^, exemplified for the conversion of **1a**^**+**^ with tetramethylthiourea, can be described by the same mechanism, although higher energy barriers are calculated (Figure S125). However, the corresponding thiophosphiranes **8c**^**+**^ could not be observed spectroscopically.

Following the initially reported protocols for the phospha-Wittig reaction,^9,38^ phosphaalkenes **5a-e**^**+**^ and phosphanides **6a-c**^**+**^ can also be synthesized using one-pot reactions of dichlorophosphane **3**[OTf] with thiocarbonyls in the presence of Ph_3_P and Zn, yet isolation cannot always be achieved satisfyingly (see Supporting Information S2.15).

### Cationic Disphosphiranes from [L_C_–P]^+^ Transfer to Phosphaalkenes

**Scheme 3:**
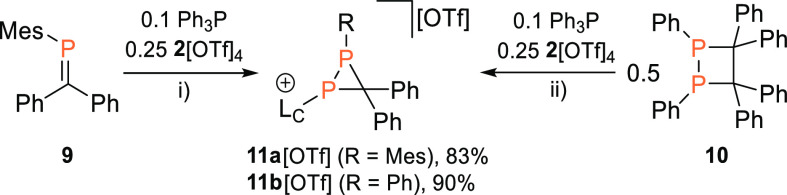
Synthesis of Diphosphiranes **11a**,**b** [OTf] (R = Mes, Ph) via [L_C_–P]^+^ Transfer from **1a**^**+**^ onto Phosphaalkenes Reagents and conditions: (i) +0.1 Ph_3_P, +0.25 **2**[OTf]_4_, CH_3_CN, rt, 2 h, 83%; (ii) +0.1 Ph_3_P, +0.5 **2**[OTf]_4_, CH_3_CN, 60 °C, 2 h, 90%.

We further explored the [L_C_–P]^+^ transfer reactivity of **1a**^**+**^ toward phosphaalkenes,^39^ which are isolobal to thioketones. Indeed, reacting **2**[OTf]_4_ with a phosphaalkene **9**(29) (4 equiv) or 1,2-diphosphetane **10**(40) (2 equiv) in the presence of catalytic Ph_3_P (0.1 equiv) afforded diphosphiranes **11a**,**b**[OTf] in very good or excellent yield (83 and 90%, respectively, Scheme 3). ^31^P NMR spectroscopic investigations on the isolated compounds showed the expected AB spin systems (**11a**[OTf]: δ(^31^P_A_) = −127.7 ppm, δ(^31^P_B_) = −100.8 ppm, ^1^*J*(PP) = 146 Hz, **11b**[OTf]: δ(^31^P_A_) = −139.5 ppm, δ(^31^P_B_) = −107.9 ppm, ^1^*J*(PP) = 133 Hz).

**Figure 6:**
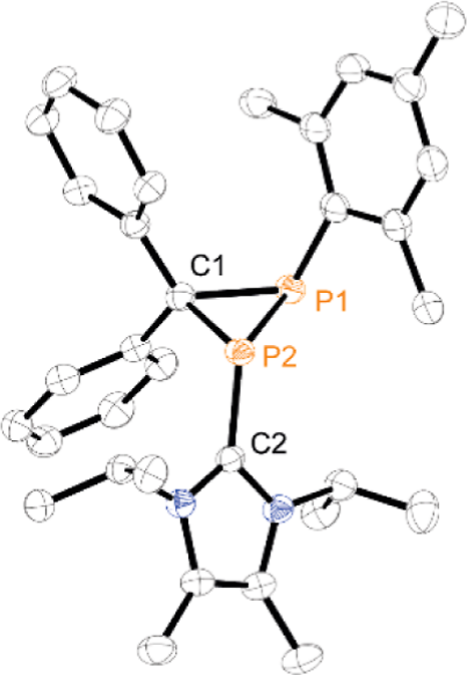
Molecular structure of **11a**^**+**^ in **11a**[OTf]; hydrogen atoms and the anion are omitted for clarity, and thermal ellipsoids are displayed at 50% probability selected bond lengths (Å) and angles (°): P1–C1 1.908(2), P1–P2 2.1905(7), P2–C1 1.887(2), P2–C2 1.852(2), P1–C1–P2 70.50(9), C1–P1–P2 54.30(7), P1–P2–C2 109.68(7), C1–P2–C2 106.65(9).

The observed high field-shifted resonances for both phosphorus nuclei are characteristic for phosphorus-containing three-membered ring {e.g., [(L_C_)_3_P_3_][OTf]_3_, (tBuP)_3_}.^16,41^ The molecular structures of **11a-b**^**+**^ in **11a**[OTf] (Figure 6) and **11b**[OTf]·*o*-C_6_H_4_F_2_ (Figure S86) reveal shortened P–P bond lengths [**11a**^**+**^: 2.1905(7) Å, **11b**^**+**^: 2.1817(4) Å] and acute P1–C1–P2 bond angles [**11a**^**+**^: 70.50(9)°, **11b**^**+**^: 70.82(15)°] in the range of other diphosphiranes^42^ and diphosphiranium cations [RP(R(CH_2_^*t*^Bu)P)((^*t*^Bu)HC)]^+^ (R = ^*t*^Bu, Ad).^43^

### Reactivity of Cationic Phosphaalkenes

**Scheme 4:**
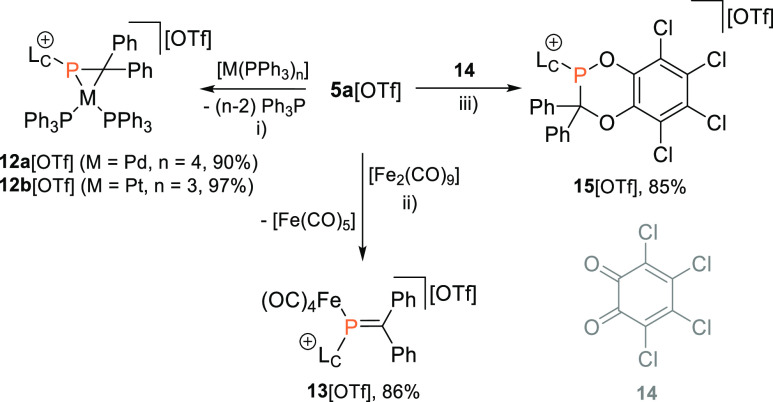
Reactions of Phosphaalkenes **5a**[OTf] with Low Oxidation State Transition Metal Complexes [M(Ph_3_P)_*n*_] (M = Pd: *n* = 4, M = Pt: *n* = 3) toward Metallaphosphiranes **12a**,**b**[OTf], with [Fe_2_(CO)_9_] toward Iron Complex **13**[OTf] and with 3,4,5,6-Tetrachloro-1,2-benzoquinone (**14**) toward **15**[OTf] Reagents and conditions: (i) +[M(PPh_3_)_*n*_], −(*n* – 2) Ph_3_P, C_6_H_5_F (**12a**[OTf]), toluene (**12b**[OTf]), rt, 1–4 h, 90–97%; (ii) +[Fe_2_(CO)_9_], −[Fe(CO)_5_], THF, rt, 16 h, 86%; (iii) +14, C_6_H_5_F, rt, 1 h, 85%.

Motivated by the successful isolation of **5a-e**[OTf], we further probed the reactivity of **5a**[OTf], as a model compound for cationic phosphaalkenes, due to its structural similarity to the well-studied MesP=CPh_2_, toward typical conversions of phosphaalkenes (Scheme 4). Treating **5a**[OTf] with [Pd(PPh_3_)_4_] or [Pt(PPh_3_)_3_] gave the metallaphosphiranes **12a**[OTf] and **12b**[OTf], respectively, under concomitant release of Ph_3_P as evidenced by ^31^P NMR spectroscopy. The η^2^-coordination of the phosphaalkenes was indicated by a strong high field shift [**12a**^**+**^: δ(^31^P_A_) = −5.6 ppm (br), **12b**^**+**^: δ(^31^P_A_) = −55.3 ppm] compared to the resonance of **5a**[OTf] and the modest coupling constant to the Pt atom in **12b**[OTf] [^1^*J*(P_A_Pt) = 564 Hz].^44^ The ^195^Pt NMR spectrum of **12b**[OTf] showed the expected doublet of doublet of doublet resonance at δ(^195^Pt) = −4822 ppm [^1^*J*(PtP) = 3571 Hz, ^1^*J*(PtP) = 3207 Hz, and ^1^*J*(PtP) = 563 Hz, Figure S102].

In general, the chemical shifts for **12a**,**b**^**+**^ are comparable to the reported values for some related η^2^-diphosphene complexes^16,45^ and the η^2^-phosphaalkene complex [Pt(Ph_3_P)_2_(η^2^-MesP=CPh_2_)].^46^ Notably, the latter phosphaalkene complex was structurally characterized as the η^1^-complex with the η^2^-complex only being observed by ^31^P NMR spectroscopy at −70 °C in solution. Remarkably, the ^31^P NMR spectra of solutions **12a-b**[OTf] in toluene-*d*_8_ did not show evidence for the formation of η^1^-complex upon heating to 100 °C. The latter might be a result of an increased π-acceptor ability in **12a**,**b**^**+**^ due to the presence of the imidazoliumyl-substituent, thereby favoring the η^2^-coordination mode.^47^ After workup, analytically pure red **12a**[OTf] and yellow **12b**[OTf] were obtained in excellent yields (90 and 97%, respectively).

**Figure 7:**
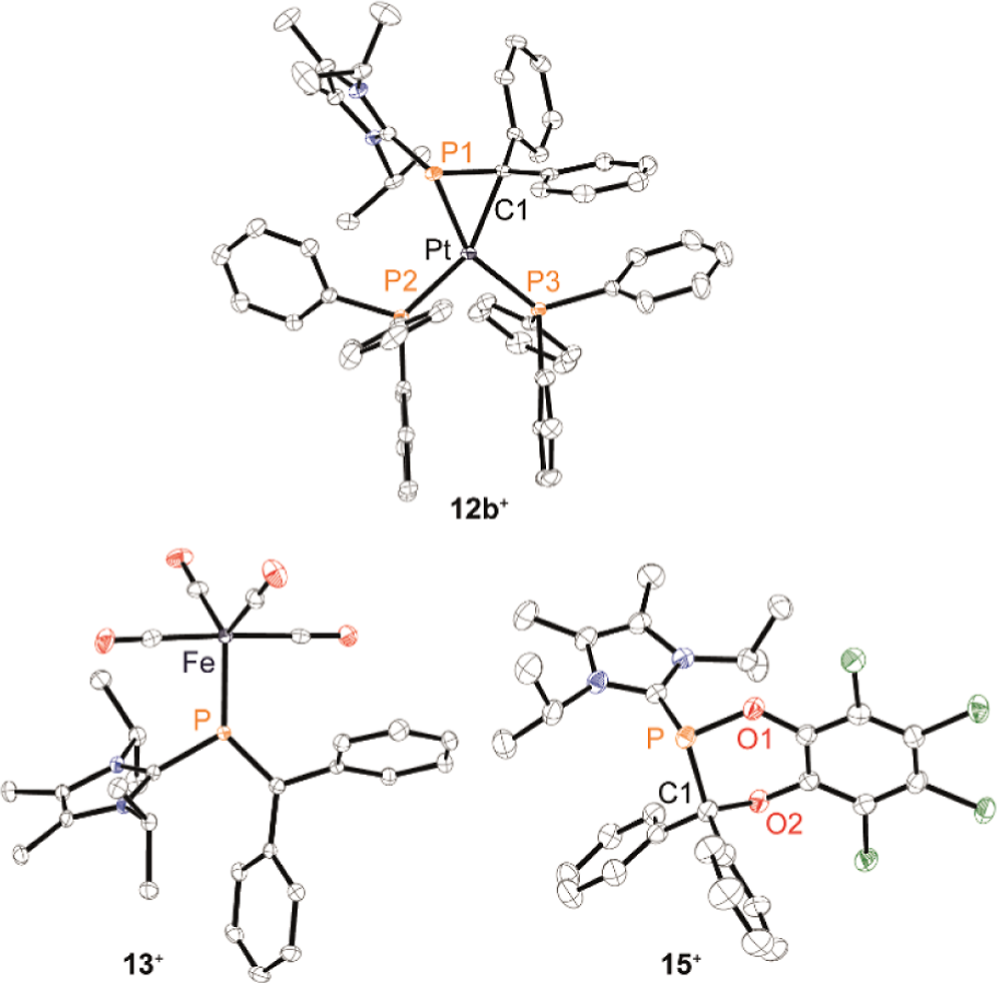
Molecular structures of metallaphosphirane **12b**^**+**^ in **12b**[OTf], iron complex **13**^**+**^ in **13**[OTf] (left) and of **15**^**+**^ in **15**[OTf]·0.5C_6_H_5_F·*n*-pentane (right); hydrogen atoms and anions are omitted for clarity and thermal ellipsoids are displayed at 50% probability; selected bond lengths (Å) and angles(°): **12b**^**+**^ (Pt): P1–C1 1.8260(18), Pt–P1 2.3127(5), Pt–C1 2.1718(16), Pt···P1–C1 2.0418(10), P1–Pt–C1 47.93(5); **13**^**+**^: P–C1 1.6846(18), P–Fe 2.1697(5), C–P–C 106.51(8), ⌀C≡Oax.: 1.1405, ⌀C≡Oeq.: 1.1435; **15**^**+**^: P–C1 1.897(3), P–O1 1.657(2), C1–O2 1.458(3); C1–P–C2 104.01(12)°.

Subsequently, single crystals of each were obtained and characterized by X-ray crystallography (Figures 7 and S96). Both complexes show the expected trigonal core, including the P–C bond of the [L_C_P=CPh_2_]^+^ ligand and the metal centers. Therein, the P–M–C bond angles are relatively acute [**12a**^**+**^ (M = Pd): 45.89(12)–46.46(9)°, **12b**^**+**^ (M = Pt): 47.93(5)°]. In line with the η^2^-coordination mode, the P=C bond in both metallaphosphiranes is elongated to the extent of a P–C single bond [**12a**^**+**^: 1.782(4)–1.787(5) Å, **12b**^**+**^: 1.8260(18) Å] presumably due to electron-backdonation from the metal centers into the π*-orbitals of the P=C bond.^48^ Similar structural features have been observed for other transition-metal complexes involving η^2^-phosphaalkene or η^4^-phosphabutadiene ligands.^49^

We further reacted phosphaalkene **5a**[OTf] (1 equiv) with [Fe_2_(CO)_9_] (1 equiv) in THF, which led to a red precipitate after 16 h at room temperature. Analysis of a CD_3_CN solution of the red product by means of ^31^P NMR spectroscopy showed only one singlet resonance at δ(^31^P) = 154.5 ppm. The slight downfield shift compared to that for **5a**[OTf] [δ(^31^P) = 152.8 ppm] suggests η^1^-phosphaalkene complex **13**[OTf] (Scheme 4). Crystallographic analysis of single crystals confirmed that the phosphaalkene ligand binds in a η^1^-fashion in the equatorial position of the Fe^0^ center (Figure 7). The P–Fe–CO_eq_ [119.62(6)° and 122.44(7)°] and P–Fe–CO_ax_ angles [88.29(6)° and 89.69(6)°] are typical of [FeL(CO)_4_] complexes, where L is a π-acceptor.^50^ As a result, the bonding parameters of the phosphaalkenes ligand are only slightly affected compared to uncoordinated **5a**^**+**^.^51^

For instance, the P=C bond in **13**[OTf] is marginally shortened [1.6846(18) Å versus **5a**^**+**^: 1.703(3) Å] and the C–P–C bond angle is widened [106.51(8)° versus **5a**^**+**^: 104.55(16)°], which may indicate a strengthening of the double bond character. Compound **13**[OTf] could be isolated in 86% yield. The IR stretching frequencies of the CO ligands are found at 2073, 2013, 1993, and 1965 cm^–1^, which renders the ligand properties of **5a**^**+**^ similar to those observed in phosphites, according to Tolman analysis.^52^

To evaluate the potential of the P=C double bond in **5a**[OTf] to be involved in cycloaddition reactions, we performed its conversion with 3,4,5,6-tetrachloro-1,2-benzoquinone (**14**). Upon dropwise addition of the red solution of **14** in C_6_H_5_F to yellow **5a**[OTf], a colorless reaction mixture is obtained immediately. X-ray analysis of single crystals obtained by vapor diffusion of *n*-pentane into the reaction mixture confirms **15**[OTf] as the product resulting from a [4 + 2] cycloaddition reaction (Figure 7). While the C1–P–C2 bond angle is barely affected [104.01(12)°], the P–C bond length is significantly elongated [1.897(3) Å] as a result of the conversion, and the P atom takes on a trigonal pyramidal geometry. The loss of the double bond character is consistent with the high field shift in the ^31^P NMR spectrum of **15**^**+**^ [δ(^31^P) = 110.3 ppm], which has been observed in the reaction of related phosphaalkenes with the same and other ortho-quinones.^53^

### Replacement Reactions of Imidazoliumyl Substituent L_C_

**Figure 8:**
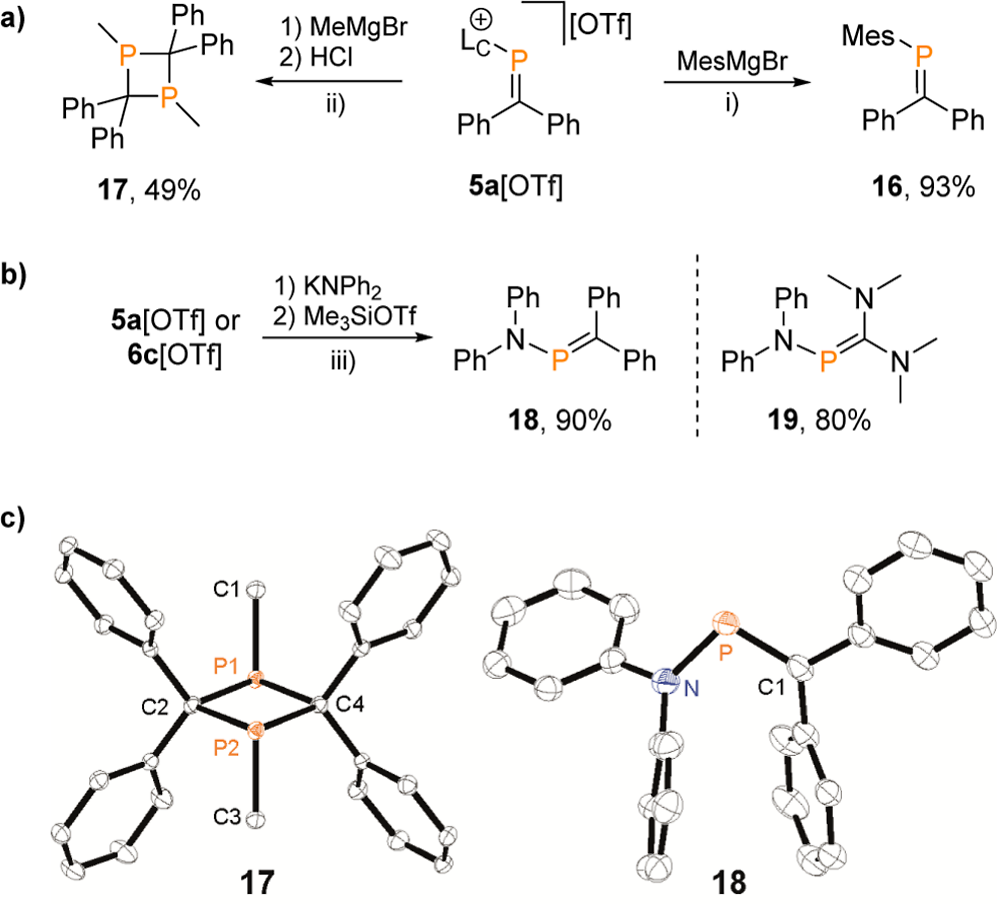
(a) Substitution reactions of the cationic imidazoliumyl substituent in **5a**[OTf] using Grignard reagents; reagents and conditions: (i) +MesMgBr (1 M in THF), −0.5 [Mg(OTf)_2_(THF)_4_], −0.5 [MgBr_2_(L_C_)_2_], THF, −78 °C, 30 min, 93%; (ii) +MeMgBr (1 M in ^*n*^Bu_2_O), THF, −78 °C, 30 min, quenched with 2 M HCl in Et_2_O, 49%; (b) reactions of **5a**[OTf] and **6c**[OTf] with KNPh_2_; reagents and conditions: (iii) +KNPh_2_, THF, rt, 30 min, quenched with Me_3_SiOTf, 90% (for **18**), 80% (for **19**); (c) molecular structures of 1,3-diphosphetane **17** and aminophosphaalkene **18**; hydrogen atoms are omitted for clarity, and thermal ellipsoids are displayed at 50% probability; selected bond lengths (Å) and angles(°): for **17**: P1–C1 1.8373(12), P1–C2 1.9085(11), P1–C4 1.9096(12), C1–P1–C2 103.44(5), C2–P1–C4 88.30(5), P1–C2–P2 91.70(5), C1–P1–P2–C3 180.00(9); **18**: P–C1 1.726(2), P–N 1.7132(17), C1–P–N 103.28(9).

We further assumed that, due to the cationic charge in synthesized **5a**-**f**^**+**^ and **6a**-**c**^**+**^, their interaction with nucleophiles may lead to substitution at the P atom. Based on this, we hypothesized that the reaction of **5a**[OTf] with MesMgBr could provide a practical route to obtaining MesP=CPh_2_ (**16**), a significant monomer for the creation of P-containing polymers^54^ that is typically synthesized via the phospha-Peterson reaction.^55^ As confirmed by ^31^P NMR spectroscopy, treating the cationic phosphaalkene **5a**[OTf] with 1 equiv of MesMgBr in THF at −78 °C resulted in its complete conversion to MesP=CPh_2_ (**16**) within 30 min (Figure 8a).

The product was conveniently obtained in 93% yield after extraction with *n*-hexane and subsequent recrystallization. One of the side products of the reaction was identified crystallographically as [MgBr_2_(L_C_)_2_].^56^

The successful conversion of **5a**[OTf] to MesP=CPh_2_ prompted us to expand our investigation to screening reactions with other nucleophiles that would result in the substitution of the imidazoliumyl substituent. Without adequate steric protection at the P atom, phosphaalkenes have a tendency to dimerize into either head-to-head (1,2-diphosphetanes) or head-to-tail (1,3-diphosphetane) dimers.^40,55,57^ Consequently, the ^31^P NMR spectrum of an aliquot removed from the reaction mixture of **5a**[OTf] with MeMgBr at −78 °C after quenching with HCl and warming to room temperature shows two new major resonances at δ(^31^P) = 35.6 ppm (70% integral ratio) and δ(^31^P) = −34.8 ppm (18% integral ratio), which we assigned to the head-to-tail and head-to-head dimers of MeP=CPh_2_, respectively. 1,3-Diphosphetane (**17**) was isolated from the reaction mixture in 49% yield (Figure 8a) and structurally characterized by single crystal X-ray analysis (Figure 8c). The nearly square planar P_2_C_2_ core of **17** features P–C bond distances of approximately 1.909 Å and bond angles C2–P1–C4 and P1–C2–P2 of 88.30(5)° and 91.70(5)°, respectively. The two methyl substituents are arranged in a trans position, forming a dihedral angle C1–P1–P2–C3 of 180.00(9)°.^57^

In some cases, thermodynamic equilibria between phosphaalkenes and their dimers have been reported.^40,57a,57c,57e^ Remarkably, no meaningful change within the ^1^H and ^31^P NMR spectra of isolated **17** in toluene-*d*_8_ was observed upon stepwise heating to 80 °C.

In a separate experiment, we investigated the reaction of two model compounds, **5a**[OTf] and **6c**[OTf], which are representative of cationic phosphaalkenes and phosphanides, respectively, toward KNPh_2_. The reaction was carried out by adding 1 equiv of KNPh_2_ to solutions of either **5a**[OTf] or **6c**[OTf] in THF at ambient temperature. After 30 min, the formation of free ^Me/iPr^NHC was observed, as confirmed by ^1^H and ^13^C NMR spectroscopic analysis of a sample removed from the reaction mixture. The ^31^P NMR spectra displayed low-field shifted resonances in both cases, indicating the exchange of the L_C_-substituent with an amino group, resulting in the formation of two new examples of aminophosphaalkenes [**18**: δ(^31^P) = 231.0 ppm; and **19**: δ(^31^P) = 106.1 ppm; Figure 8b]. Since both resulting compounds had similar solubility to free ^Me/iPr^NHC, 1 equiv of Me_3_SiOTf was added to the respective reaction mixture, resulting in the formation of [L_C_SiMe_3_][OTf], which is a precursor in the synthesis of **3**[OTf].^58^ Subsequently, **18** and **19** were isolated via extraction with *n*-hexane, with yields of 90 and 80%, respectively. Single crystals of both compounds were obtained through recrystallization from *n*-hexane or *n*-pentane and subjected to X-ray analysis.

In the molecular structures of **18** [P–C1 1.7132(17) Å, Figure 8 bottom], the P–C1 bond length is slightly elongated compared to a typical P=C double bond. This elongation is attributed to the donating effect of the P-amino substituent. The P–C1 bond length in C-amino substituted **19** [P–C1 1.754(1) Å, Figure S123] is even further elongated. The P–N bonds in both compounds [**18**: P–N 1.726(2) Å; **19**: P–N 1.7501(12) Å] are comparable with other structurally related compounds.^59^

## Conclusions

In summary, we showed the synthesis of cationic imidazoliumyl(phosphonio)-phosphanides **1a-d**^**+**^ via the nucleophilic fragmentation of tetracationic tetraphosphetane **2**[OTf]_4_ with tertiary phosphanes R_3_P (R = Ph, Me, Et, Cy). We tested their ability to undergo the hitherto unknown transfer of a cationic phosphinidene, that is, [L_C_–P]^+^. Employing *in situ* generated **1a**^**+**^ or isolated **1d**[OTf] in phospha-Wittig-type reactions with thiocarbonyls allowed the isolation and characterization of a series of novel cationic phosphaalkenes **5a-f**^**+**^ as well as phosphanides **6a-d**^**+**^ bearing a wide variety of substituents as their triflate salts. As evidenced spectroscopically and by DFT calculations [RI-BP86-D3/def2-TZVP (acetonitrile) level of theory], the mechanism of the formation of phosphaalkenes proceeds via the intermediary three-membered thiophosphiranes as a result of a [L_C_–P]^+^ transfer from **1a**^**+**^ onto the C=S double bond. Although calculations show a similar pathway for the formation of phosphanides, energy barriers are found to be significantly higher. Furthermore, when *in situ* generated **1a**^**+**^ is reacted with phosphaalkenes that are isolobal to thioketones, [L_C_–P]^+^ transfer is also observed, leading to the isolation of heteroleptic diphosphiranes **11a**,**b**[OTf].

In order to evaluate the reactivity of the formed cationic phosphaalkenes, we subjected **5a**[OTf] to reactions with low oxidation state transition-metal complexes [Pd(PPh_3_)_4_], [Pt(PPh_3_)_3_], and [Fe_2_(CO)_9_]. While the conversion with the latter gave iron complex **13**[OTf], in which the phosphaalkenes are in an equatorial position and have a η^1^-coordination mode, metallaphosphiranes **12a,b**[OTf] are formed in the reaction with the former two complexes, including **5a**^**+**^ in a η^2^-coordination mode. Lastly, we showed the potential to use the P=C double bond in **5a**[OTf] for cycloaddition reactions by its conversion with ortho-quinone **14** giving **15**[OTf].

We furthermore exemplified the possibility of exchanging the transferred L_C_-substituent in **5a**[OTf] by reacting it with MesMgBr. This reaction allowed for the convenient and high-yield synthesis of MesP=CPh_2_, a compound that is typically obtained through reactions involving malodorous primary phosphines and silylphosphines. Moreover, our work enables access to unprecedented 1,3-diphosphetane **17** as well as aminophosphaalkenes **18** and **19** through the conversions of **5a**[OTf] and **6c**[OTf] with MeMgBr or KNPh_2_. These conversions demonstrate the versatility of [L_C_–P]^+^ as a P_1_ building block.

## Supporting Information Available

Experimental and characterization details for all new compounds, including spectroscopic data and NMR spectra, molecular structures, crystallographic data, and computational details (PDF)
