# Supplementary material for: Cationic Phosphinidene as a Versatile P1 Building Block: [LC–P]+ Transfer from Phosphonio–Phosphanides [LC–P–PR3]+ and Subsequent LC Replacement Reactions (LC = N-Heterocyclic Carbene)
Source: J Am Chem Soc. 2023 Apr 27;145(18):10364–75. doi: 10.1021/jacs.3c02256 (PMC10177976; doi:10.1021/jacs.3c02256)
Supplement: Supplementary file 1 — ja3c02256_si_001.pdf [file ja3c02256_si_001.pdf]

Supporting Information

Cationic Phosphinidene as a Versatile P_1 Building Block: $[L_C\text{-}P]^+$ Transfer from Phosphonio-Phosphanides $[L_C–P–PR_3]^+$ and Subsequent L_C Replacement Reactions (L_C = N-Heterocyclic Carbene)

Philipp Royla,[a] Kai Schwedtmann,[a] Zeyu Han,[b] Jannis Fidelius,[a] Derek P. Gates,[b,*] Rosa M. Gomila,[c] Antonio Frontera,[c] Jan J. Weigand[a,*]

[a] *Chair of Inorganic Molecular Chemistry, Faculty of Chemistry and Food Chemistry, Technische Universität Dresden, 01069 Dresden, Germany*

[b] *Chemistry Department, The University of British Columbia, V6T 1Z1 Vancouver, Canada*

[c] *Department of Chemistry, Universitat de Illes Balears, 07122 Palma de Mallorca, Spain*

Content

S1. General remarks. 2

S2. Synthetic procedures, spectroscopic data and additional remarks. 3

S2.1 Preparation of $2[OTf]_4$ 3

S2.2 Reaction of 1d[OTf] with thiobenzophenone 3

S2.3 Reaction of 3[OTf] with Ph_3P, Zn and thiobenzophenone 4

S2.4 Reaction of $2[OTf]_4$ with thiobenzophenone 4

S2.5 Reaction of 5a[OTf] with S_8 6

S2.6 Reactions of 3[OTf] with R_3P (R = Me, Et, Cy, Ph) 7

S2.7 Reaction of 3[OTf] with 2eq. of Zn or Ph_3P and thiobenzophenone 8

S2.8 Reaction of $2[OTf]_4$ with ditopic phosphanes 8

S2.9 Preparation of $[(L_C)P–P(Me)_3][OTf]$ (1b[OTf]) 9

S2.10 Preparation of $[(L_C)P–P(Et)_3][OTf]$ (1c[OTf]) 11

S2.11 Preparation of $[(L_C)P–P(Cy)_3][OTf]$ (1d[OTf]) 13

S2.12 Preparation of $[(L_C)P–P(Ph)_3]^+$ ($1a^+$) 15

S2.13 Preparation of $[(L_C)P–P(Ph_2CH_2PPh_2)][OTf]$ (1e[OTf]) 15

S2.14 Reaction of 1b[OTf] with 4-methoxybenzaldehyde 17

S2.15 General procedures of the one-pot reaction for the formation of 5a-e[OTf] and 6a-d[OTf] 17

S2.16 Preparation of $[(L_C)P{=}C(Ph)_2][OTf]$ (5a[OTf]) 19

S2.17 Preparation of $[(L_C)P{=}C(4,4'\text{-}Cl\text{-}Ph)_2][OTf]$ (5b[OTf]) 21

S2.18 Preparation of $[(L_C)P{=}C(Ph\text{-}p\text{-}N(Me)_2)_2][OTf]$ (5c[OTf]) 23

S2.19 Preparation of [(*E*/*Z*)-(L_C)P=C(Ph)-2-thiophenyl][OTf] (5d[OTf]) 25
S2.20 Preparation of [(L_C)P=C(C_9H_{16})][OTf] (5e[OTf]) 27
S2.21 Preparation of [(L_C)P–C(H)N(Me)$_2$][OTf] (6a[OTf]) 29
S2.22 Preparation of [(L_C)P–C(Ph)N(Me)$_2$][OTf] (6b[OTf]) 31
S2.23 Preparation of [(L_C)P–C(N(Me)$_2$)$_2$][OTf] (6c[OTf]) 33
S2.24 Reaction of 2[OTf]$_4$ with 10 eq. of Ph_3P and 1,3-diisopropyl-4,5-dimethyl-1,3-dihydro-2H-imidazole-2-thione 35
S2.25 Reaction of 2[OTf]$_4$ with 4 eq. of Ph_3P and excess of *O*-methyl benzothioate 35
S2.26 Preparation of [(L_C)P(Fe(CO)$_4$)C(Ph)$_2$][OTf] (13[OTf]) 36
S2.27 Preparation of [(L_C)P–P(Mes)–C(Ph)$_2$][OTf] (11a[OTf]) 38
S2.28 Preparation of [(L_C)P–P(Ph)–C(Ph)$_2$][OTf] (11b[OTf]) 40
S2.29 Preparation of [Pd(Ph_3P)$_2$-η^2-((L_C)P=C(Ph)$_2$)][OTf] (12a[OTf]) 43
S2.30 Preparation of [Pt(Ph_3P)$_2$-η^2-((L_C)P=C(Ph)$_2$)][OTf] (12b[OTf]) 46
S2.31 Preparation of [(L_C)P–($C_6Cl_4O_2$)–C(Ph)$_2$][OTf] (15[OTf]) 48
S2.32 Preparation of MesP=C(Ph)$_2$ (16) 50
S2.33 Preparation of 1,3-dimethyl-2,2,4,4-tetraphenyl-1,3-diphosphetane 17 52
S2.34 Preparation of (Ph_2N)P=C(Ph)$_2$ (18) 54
S2.35 Preparation of (Ph_2N)P=C(NMe_2)$_2$ (19) 55
S3 Single Crystal X-ray Diffraction Data 57
S3.1 General remarks 57
S3.2 Refinement details 58
S4. Computational data 67
S5. References 85

S1. General remarks.

All manipulations were performed in a Glovebox or using Schlenk techniques under an atmosphere of purified argon or nitrogen. Dry, oxygen-free solvents were distilled either from CaH_2 or from potassium. Deuterated solvents were purchased from Merck, Deutero or Eurisotop. All solvents were stored over molecular sieves (4 Å: CH_2Cl_2, C_6H_5F, *n*-pentane, *n*-hexane, THF, THF-d_8, toluene-d_8, Et_2O, CD_2Cl_2; 3 Å: CD_3CN, CH_3CN). All glassware was oven-dried at 160 °C prior to use. Anhydrous deuterated acetonitrile (CD_3CN), dichloromethane (CD_2Cl_2) were purchased from Sigma-Aldrich. Reagents **3**[OTf][1], **2**[OTf]$_4$[2], **9**[3], **10**[4] as well as thiobenzophenone, thiophenylphenylthioketone, *O*-methylbenzothioate[5] 4,4′-bis(chloro)thiobenzophenone, (1*R*,4*R*)-thiocampher[6], *N,N*-dimethylbenzothioamide[7], 1,3-diisopropyl-4,5-dimethyl-1,3-dihydro-2H-imidazole-2-thione[8] and [Pt(Ph_3P)$_3$][9], MesMgBr (1M in THF)[10] and $KNPh_2$[11] were prepared according to procedures given by literature. Reagents 4,4′-bis-(dimethylamino)-thiobenzophenone, *N*,*N*,*N*,*N*-tetramethylthiourea, [$Fe_2(CO)_9$], [Pd(Ph_3P)$_4$] and MeMgBr (1M in *n*Bu_2O) were purchased from Sigma Aldrich and used as received. *N*,*N*-dimethylthioformamid was purchased from Sigma Aldrich and recondensed in an inert atmosphere and stored over 4 Å molecular sieve. Ph_3P, Cy_3P, bis(diphenylphosphino)methan (dppm), bis(diphenylphosphino)ethan (dppe) and 3,4,5,6-tetrachloro-1,2-benzoquinone were purchased

from Sigma Aldrich or Acros Organics and sublimed prior to use. Me_3P and Et_3P were distilled in an inert atmosphere prior to use. NMR spectra were measured on a Bruker AVANCE III HD Nanobay 400 MHz UltraShield (^{1}H: 400.13 MHz, ^{13}C: 100.61 MHz, ^{31}P: 161.98 MHz, ^{19}F: 376.50 MHz, ^{195}Pt: 86.01 MHz), or on a Bruker AVANCE III HDX, 500 MHz Ascend (^{1}H: 500.13 MHz, ^{13}C: 125.75 MHz, ^{31}P: 202.45 MHz, ^{19}F: 470.59 MHz). Reported numbers assigning atoms in the ^{13}C spectra were indirectly deduced from the cross-peaks in 2D correlation experiments (HMBC, HSQC). Chemical shifts are referenced to $\delta(Me_4Si)$ = 0.00 ppm (^{1}H, ^{13}C, externally), $\delta(CFCl_3)$ = 0.00 ppm (externally) and $\delta(H_3PO_4$, 85%) = 0.00 ppm (externally). Unless stated otherwise, all NMR spectra were measured at 300 K. Chemical shifts (δ) are reported in ppm. Coupling constants (J) are reported in Hz. The designation of the spin systems is performed by convention. The furthest downfield resonance is denoted by the latest letter in the alphabet and the furthest upfield by the earliest letter. Melting points were recorded on an electrothermal melting point apparatus (Büchi Switzerland, Melting point M-560) in sealed capillaries under Nitrogen atmosphere and are uncorrected. Infrared (IR) and Raman spectra were recorded at ambient temperature using a Bruker Vertex 70 instrument equipped with a RAM II module (Nd: YAG laser, 1064 nm). The Raman intensities are reported in percent relative to the most intense peak and are given in parenthesis. An ATR unit (diamond) was used for recording IR spectra. The intensities are reported relative to the most intense peak and are given in parenthesis using the following abbreviations: vw = very weak, w = weak, m = medium, s = strong, vs = very strong. Elemental analyses were performed on a Vario MICRO cube Elemental Analyzer by Elementar Analysatorsysteme GmbH in CHNS modus.

S2. Synthetic procedures, spectroscopic data and additional remarks.

S2.1 Preparation of 2[OTf]$_4$

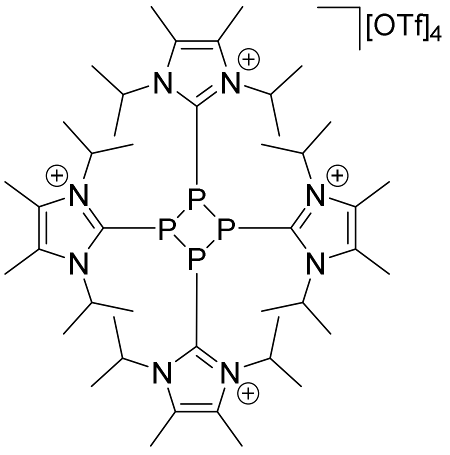

To a solution of **3**[OTf] (69.0 g, 0.16 mol, 1.0 eq.) in C_6H_5F (350 ml) a solution of 1,4-bis(trimethylsilyl)-1,4-dihydropyrazine (**4**, 37.0 g, 0.16 mol, 1.0 eq.) in C_6H_5F (150 ml) was added while stirring at room temperature. During the addition the reaction solution turned into a red suspension, which was stirred for 40 h leading to the precipitation of an off-white solid. After Filtration, washing the precipitate with C_6H_5F (3x30 ml) and drying *in vacuo* the product was afforded as an air and moisture sensitive colorless solid.

Yield: 50.84 g (88%); spectroscopic data matched literature values.[2]

S2.2 Reaction of 1d[OTf] with thiobenzophenone

To a solution of **1d**[OTf] (50 mg, 0.08 mmol, 1.0 eq.) in 1 ml CD_3CN a solution of thiobenzophenone (16 mg, 0.08 mmol, 1.0 eq.) 1 ml CD_3CN was added. The blue reaction mixture was stirred at room temperature for 10 min until an aliquot was analyzed by means of multinuclear NMR spectroscopy. The ^{31}P NMR spectrum of the reaction mixture (**Figure S1**) evidences the formation of transient thiophosphirane **7a**[OTf] even when isolated phosphonio-phosphanides, such as **1d**[OTf] are used.

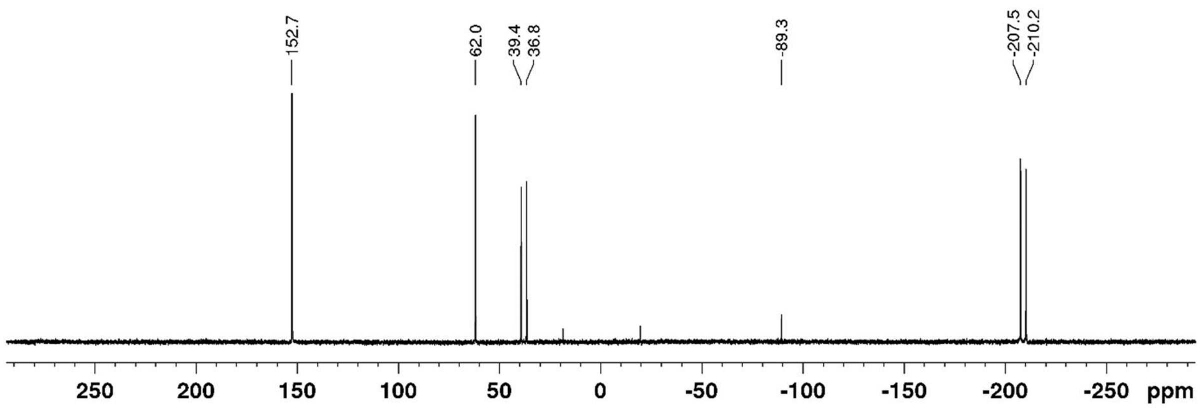

Figure S1. ^{31}P NMR spectrum of the supernatant in the reaction of **1d**[OTf] with thiobenzophenone after 10 min in CH_3CN at room temperature (C_6D_6 cap., 300 K).

S2.3 Reaction of 3[OTf] with Ph_3P, Zn and thiobenzophenone

To a mixture of **3**[OTf] (50 mg, 0.12 mmol, 1.0 eq), triphenylphosphane Ph_3P (34 mg, 0.13 mmol, 1.1 eq) and solid zinc dust (9 mg, 0.13 mmol, 1.1 eq) thiobenzophenone (25 mg, 0.13 mmol, 1.1 eq) in 2 ml THF (or CH_3CN) was added. The blue suspension was stirred at room temperature for 3 h (1 h) and the supernatant was analyzed by means of ^{31}P-NMR spectroscopy (**Figure S2**).

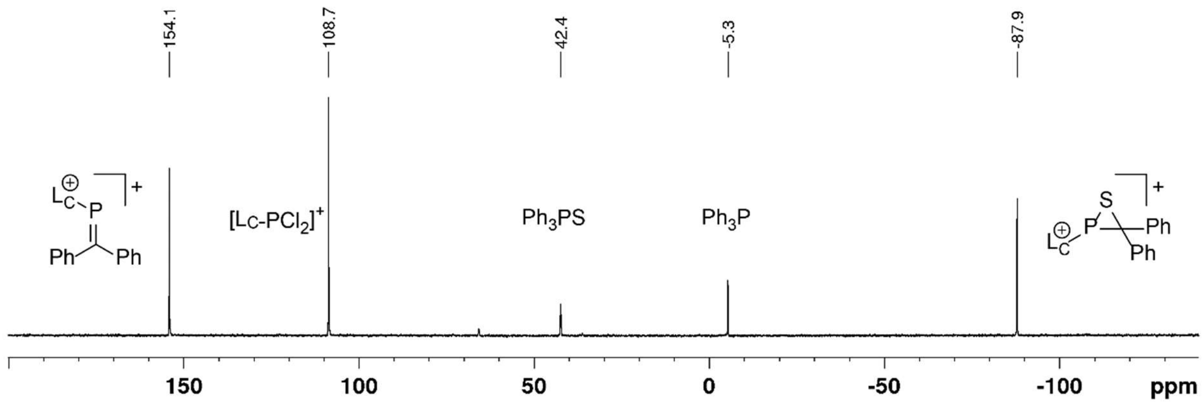

Figure S2. ^{31}P NMR spectrum of the supernatant in the reaction of **3**[OTf] with thiobenzophenone, Ph_3P and zinc dust after 3 h (1 h) in THF (CH_3CN) at room temperature (C_6D_6 cap., 300 K).

S2.4 Reaction of 2[OTf]$_4$ with thiobenzophenone

A solution of thiobenzophenone (138 mg, 0.69 mmol, 20 eq.) in 1 ml CH_3CN was added to a solution of **2**[OTf]$_4$ (50 mg, 0.03 mmol, 1 eq.) in 1 ml CH_3CN. The blue solution was stirred at room temperature for 16 h and analyzed by means of ^{31}P-NMR spectroscopy (**Figure S3**). Stirring of the reaction mixture for longer (12 d) at room temperature does not lead to significant changes within the product distribution. The product distribution can be rationalized by the reaction sequence in **Scheme S1**.

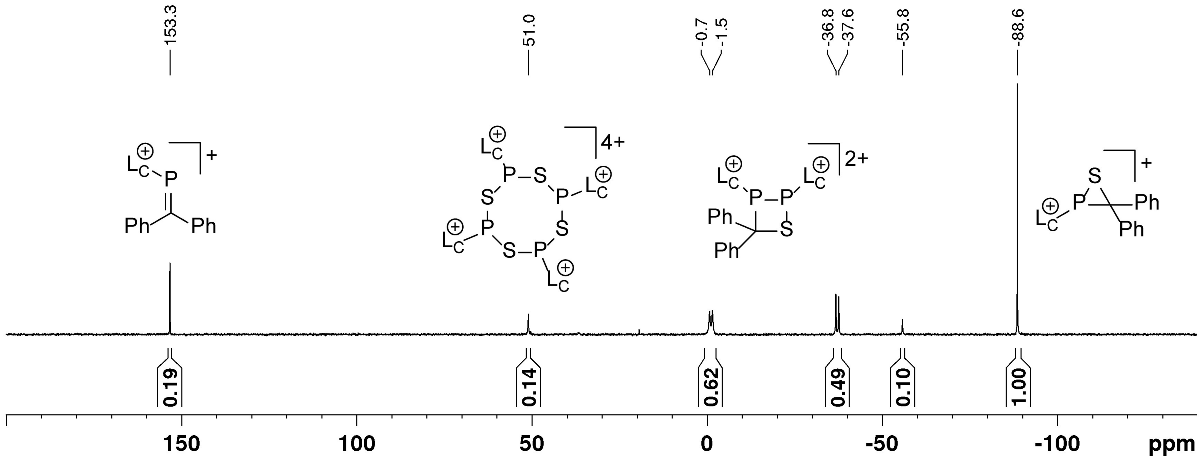

Figure S3. ^{31}P NMR spectrum of the reaction of **2**[OTf] with thiobenzophenone after 16 h in CH_3CN at room temperature (C_6D_6 cap., 300 K).

Scheme S1. Proposed reaction sequence in the conversion of 2[OTf]4 with thiobenzophenone in the absence of a reductant/S-scavenging agent

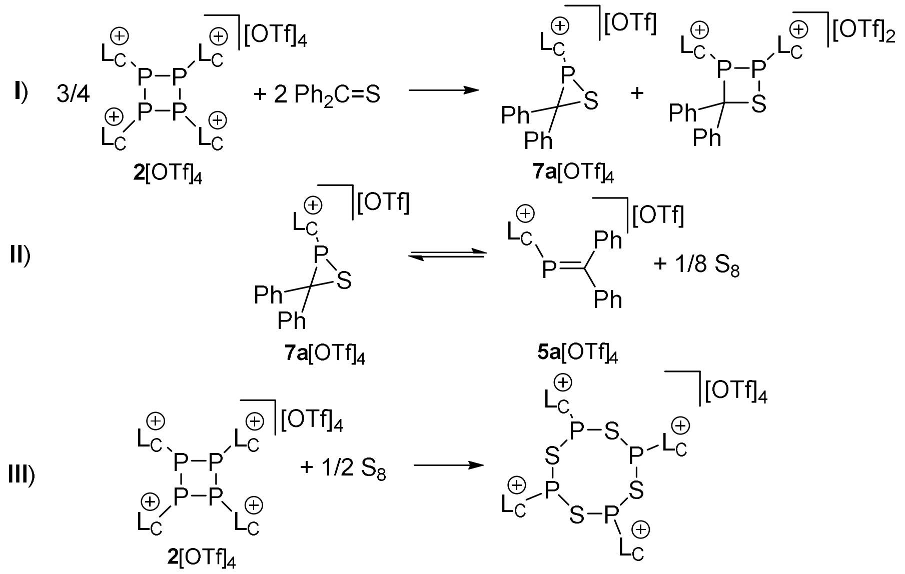

S2.5 Reaction of 5a[OTf] with S_8

To a solution of **5a**[OTf] (30 mg, 0.06 mmol, 1 eq.) in 2 ml of THF was added elemental sulfur (2 mg, 0.06 mmol, 1.1 eq.) as a solid. The mixture was heated to 80°C in a microwave reactor for 2 h and analyzed by means of ^{31}P NMR spectroscopy (**Figure S4**).

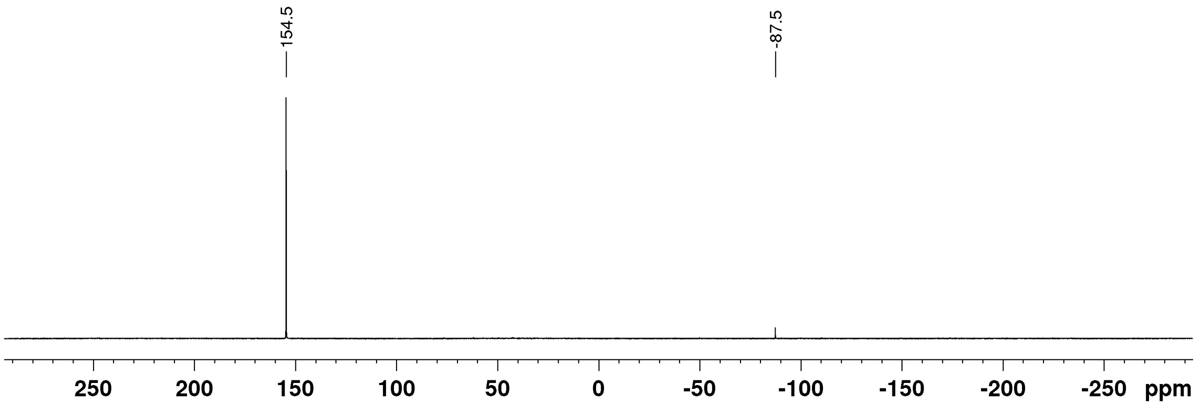

Figure S4. ^{31}P NMR spectrum of the reaction of **5a**[OTf] with elemental sulfur for 2 h at 80°C in a microwave reactor in CH_3CN (C_6D_6 cap., 300 K).

Addition of another 2/8 equivalents of S_8 to the reaction mixture and subsequent microwave irradiation for 16 h at 80°C leads to a blue reaction solution indicative of the formation of thiobenzophenone. The ^{31}P NMR spectrum of the reaction solution (**Figure S5**) shows **5a**[OTf] ($\delta(^{31}P)$ = 154.4 ppm) and multiple other unidentified products.

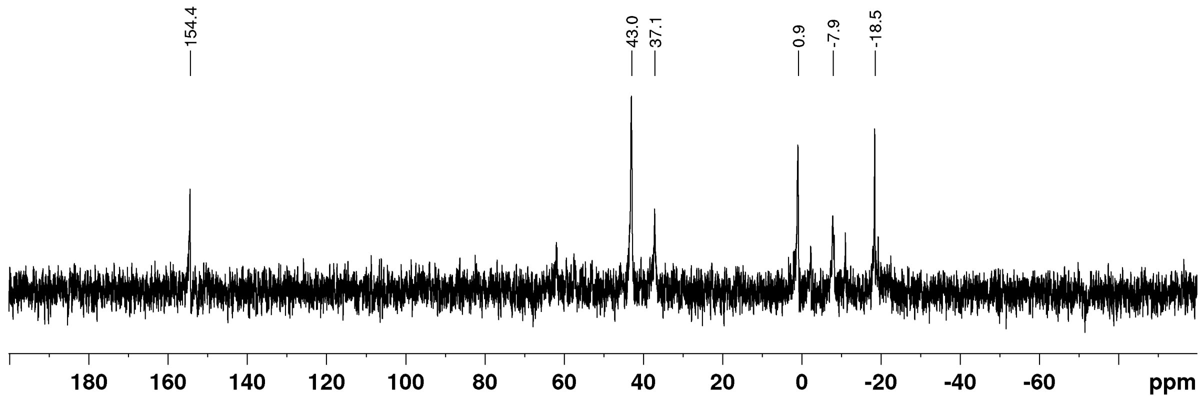

Figure S5. ^{31}P NMR spectrum of the reaction of **5a**[OTf] with 3/8 S_8 for 16 h at 80°C in a microwave reactor in CH_3CN (C_6D_6 cap., 300 K).

S2.6 Reactions of 3[OTf] with R_3P (R = Me, Et, Cy, Ph)

General procedure: To a solution of **3**[OTf] (50 mg, 0.12 mmol, 1 eq.) in 2 ml CH_3CN a tertiary phosphane R_3P (2.1 eq., R = Me, Et, Cy , Ph) was added and the mixture was allowed to stir at room temperature for 3-16 h after which an aliquot of the liquid phase was analyzed by means of ^{31}P NMR spectroscopy (**Figure S6**).

R = Me, Et, Cy: Addition of R_3P (R = Me, Et, Cy) results in a pale-yellow reaction mixture immediately. The ^{31}P NMR spectra after 3 h confirm the formation of **18**[OTf][12], **1b-d**[OTf] and [R_3PCl]Cl (R = Me, Et, Cy)[13] (**Scheme S2, I**).

R = Ph: Addition of Ph_3P results in a pale-yellow reaction mixture after 16 h. The ^{31}P NMR spectra confirm the formation of **18**[OTf] and [Ph_3PCl][OTf][13] (**Scheme S2**, **II**). Heating of the reaction mixture at 80°C for 4 h in a microwave reactor does not lead to further reduction of **18**[OTf], however the formation of some PCl_3 is observed ($\delta(^{31}P)$ = 220.3 ppm).

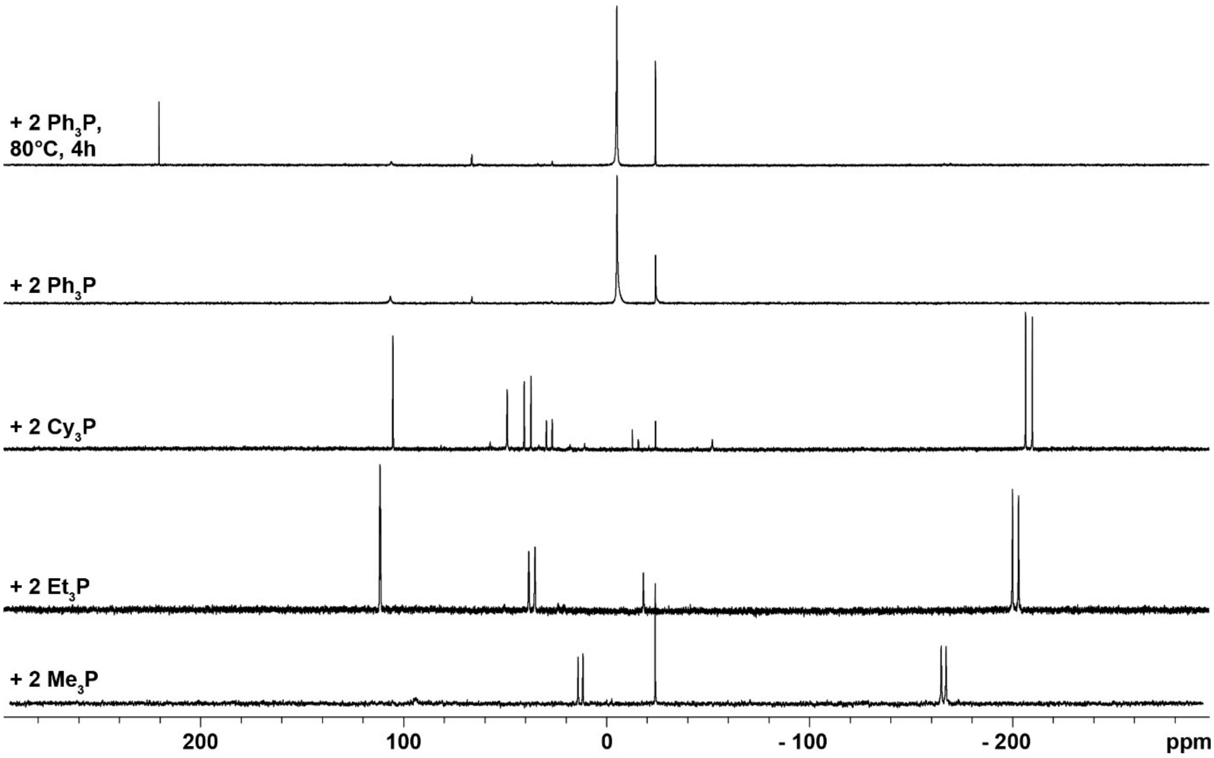

Figure S6. ^{31}P NMR spectra of the reactions of **3**[OTf] with 2 eq. of a tertiary phosphane R_3P (C_6D_6 cap., 300 K).

Scheme S2. Reactions of 3[OTf] with tertiary phosphanes

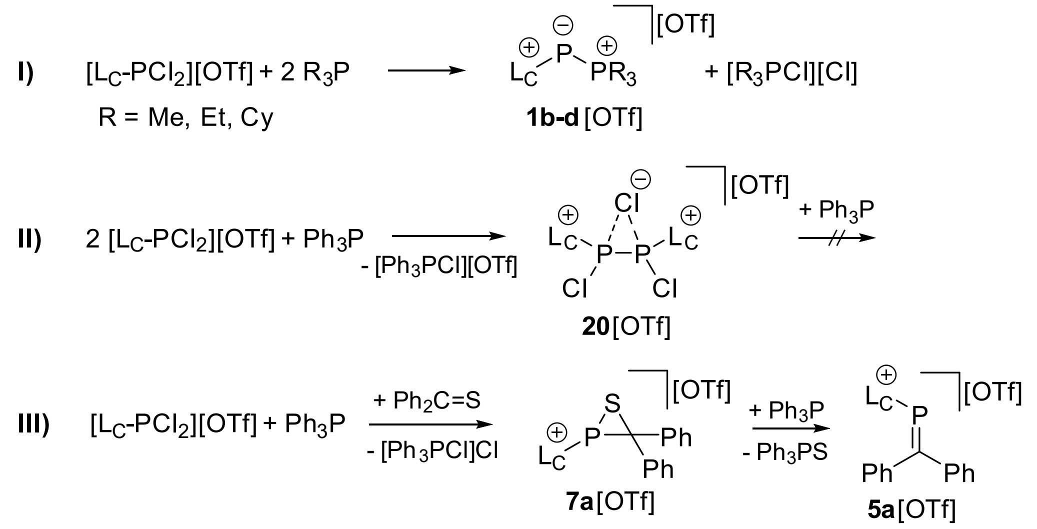

S2.7 Reaction of 3[OTf] with 2eq. of Zn or Ph_3P and thiobenzophenone

A solution of **3**[OTf] (50 mg, 0.12 mmol, 1.0 eq.) and thiobenzophenone (23.0 mg, 0.12 mmol, 1.0 eq.) in 2 ml CH_3CN was added to zinc dust (17 mg, 0.24 mmol, 2.2 eq.) and stirred at room temperature for 3 d. The ^{31}P NMR spectrum of the resulting yellow supernatant shows two major resonances, that can be assigned to thiophosphirane **7a**[OTf] as well as phosphaalkene **5a**[OTf] (**Figure S7**).

When **3**[OTf] (50 mg, 0.12 mmol, 1.0 eq.) is reacted with triphenylphosphane (67 mg, 0.26 mmol, 2.0 eq.) and thiobenzophenone (23.0 mg, 0.12 mmol, 1.0 eq.) in 2 ml CH_3CN at room temperature, the reaction mixture remains a clear blue solution after 1 d of stirring. The ^{31}P NMR spectrum shows complete conversion of **3**[OTf] to **20**[OTf], **5a**[OTf], Ph_3PS and $[Ph_3PCl]Cl$.

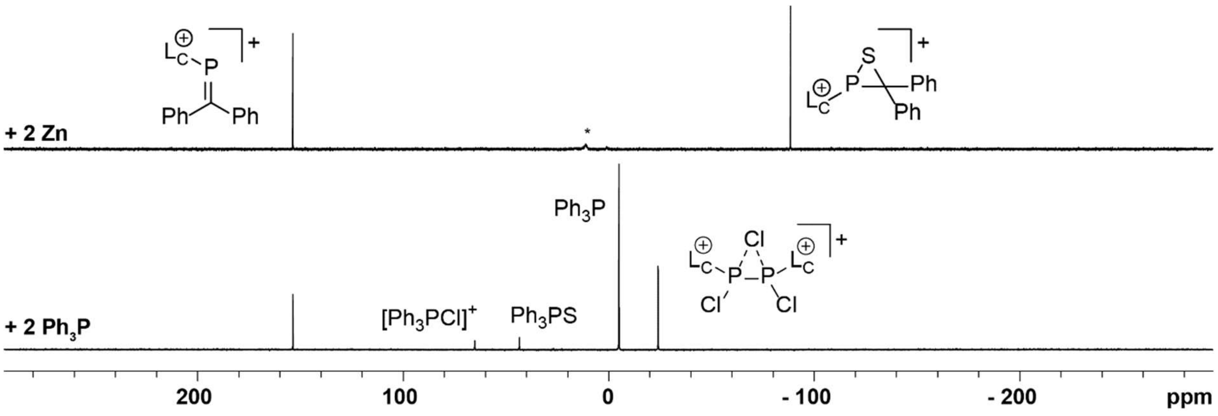

Figure S7. ^{31}P NMR spectra of the reactions of **3**[OTf] with zinc dust or Ph_3P in the presence of thiobenzophenone at room temperature (C_6D_6 cap., 300 K).

S2.8 Reaction of $2[OTf]_4$ with ditopic phosphanes

For the formation of **1e**[OTf] see **S2.12**

Formation of $\mathbf{23}^+$ and $\mathbf{6d}^+$: To a solution of **2**$[OTf]_4$ (50.0 mg, 0.03 mmol, 1.0 eq.) in CH_2Cl_2 (1 ml) a solution of bis(diphenylphosphino)ethan **22** (30.0 mg, 0.07 mmol, 2.1 eq.) in CH_2Cl_2 (1 ml) was added while stirring. The resulting orange reaction mixture was allowed to stir for 2 d at room temperature and consequently analyzed by means of multinuclear NMR spectroscopy (**Figure S8**) revealing resonances, that could be assigned to $\mathbf{23}^+$ and $\mathbf{6d}^+$.[14]

Scheme S3. Reactions of 2[OTf]$_4$ with ditopic phosphanes. Left: not observed formation of bis(phosphonio)phosphanides. Right: Formation of phosphonio-phosphanide 1e[OTf] (top) and substituent migration in the reaction of 2[OTf]$_4$ with dppe 22 to give triphosphenium cation 23$^+$ and di(imidazoliumyl)phosphanide[a] 6d$^+$.

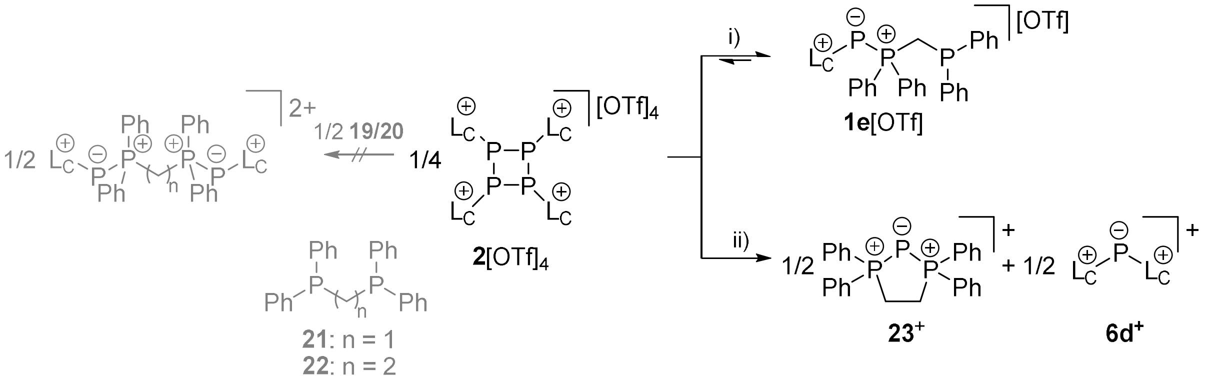

[a]i) + **21**, CH_2Cl_2, rt, 4 d, 90%; ii) + **22**, CH_2Cl_2, rt, 2 d, isolated yield not determined.

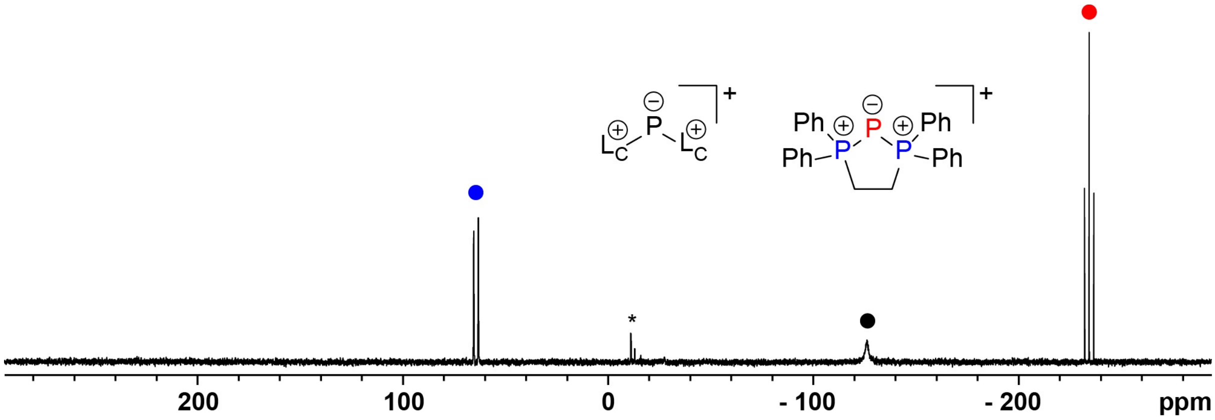

Figure S8. ^{31}P NMR spectrum of the reaction mixture of **2**[OTf]$_4$ with 2 eq. **22** in CH_3CN (C_6D_6 cap., 300 K).

S2.9 Preparation of [(L$_C$)P–P(Me)$_3$][OTf] (1b[OTf])

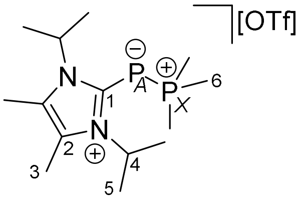

To a solution of **2**[OTf]$_4$ (750 mg, 0.52 mmol, 1.0 eq.) in CH_3CN (5 ml) cold (-30°C) Me_3P (241 µl, 178 mg, 2.3 mmol, 4.5 eq.) was added dropwise while stirring. The resulting deep red solution was stirred for 4 h at room temperature giving a misty suspension, which was filtered and evaporated to dryness. Subsequent washing of the residue with Et_2O (3x4 mL) and consecutive drying *in vacuo* afforded the product as an air and moisture sensitive off-white solid. Crystals suitable for X-ray diffraction analysis were obtained by diffusion of Et_2O into a saturated CH_3CN solution at -30°C.

Yield: 837 mg (92%); **m.p.**: 149-151 °C; **Raman** (80 mW, in cm^{-1}): 2982 (51), 2938 (45), 2914 (75), 1625 (29), 1450 (33), 1432 (19), 1407 (33), 1396 (31), 1386 (15), 1351 (44), 1290 (100), 1148 (14), 1084 (12), 1033 (61), 886 (16), 792 (15), 763 (18), 754 (32), 743 (17), 684 (24), 584 (15), 573 (15), 490 (42), 459 (17), 394 (15), 347 (25), 311 (24), 279 (24), 239 (22); **IR** (ATR, in cm^{-1}): 2981 (vw), 2940 (vw), 2361 (vw), 1624 (vw), 1468 (vw), 1444 (vw), 1422 (vw), 1387 (w), 1374 (w), 1336 (vw), 1308 (vw), 1289 (w), 1274 (s), 1257 (vs), 1221 (m), 1148 (s), 1114 (w), 1031 (vs), 965 (s), 907 (vw), 869 (vw), 857 (vw), 754 (w), 742 (vw), 708 (vw), 683 (w), 636 (vs), 571 (w), 547 (vw), 516 (m), 488 (w), 466 (vw), 415 (vw); **^{1}H NMR** (CD_3CN, 300 K, in ppm): δ = 1.5 (12H, d, $^3J_{HH}$ = 7 Hz, H-5), 1.6 (9H, d, $^3J_{HP}$ = 12 Hz, H-6), 2.3 (6H, s, H-3), 5.9 (2H, m, H-4); **^{13}C{^{1}H} NMR** (CD_3CN, 300 K, in ppm): δ = 11.0 (2C, s, C-3), 16.4 (3C, dd, $^1J_{CP}$ = 45 Hz, $^2J_{CP}$ = 11 Hz, C-6), 21.2

(4C, s, C-5), 54.0 (2C, d, $^3J_{CP}$ = 8 Hz, C-4), 122.2 (1C, q, $^1J_{CF}$ = 321 Hz, OTf), 130.2 (2C, s, C-2), 148.9 (1C, dd, $^1J_{CP}$ = 94 Hz, $^2J_{CP}$ = 8 Hz, C-1); **^{19}F{^{1}H} NMR** (CD$_3$CN, 300 K, in ppm): δ = -79.3 (3F, s, OTf); **^{31}P{^{1}H} NMR** (CD$_3$CN, 300 K, in ppm): AX spin system: δ = -167.0 (1P, d, $^1J_{PP}$ = -472 Hz, P$_A$), 12.0 (1P, d, $^1J_{PP}$ = -472 Hz, P$_X$); **elemental analysis**: calcd. for $C_{15}H_{29}F_3N_2O_3P_2S$: C: 41.28, H: 6.70, N: 6.42, S: 7.35; found: C: 41.19, H: 6.75, N: 6.51, S: 7.34.

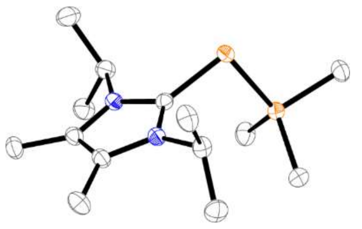

Figure S9. Molecular structure of **1b**$^+$ in **1b**[OTf]; hydrogen atoms and the anion are omitted for clarity and thermal ellipsoids are displayed at 50% probability.

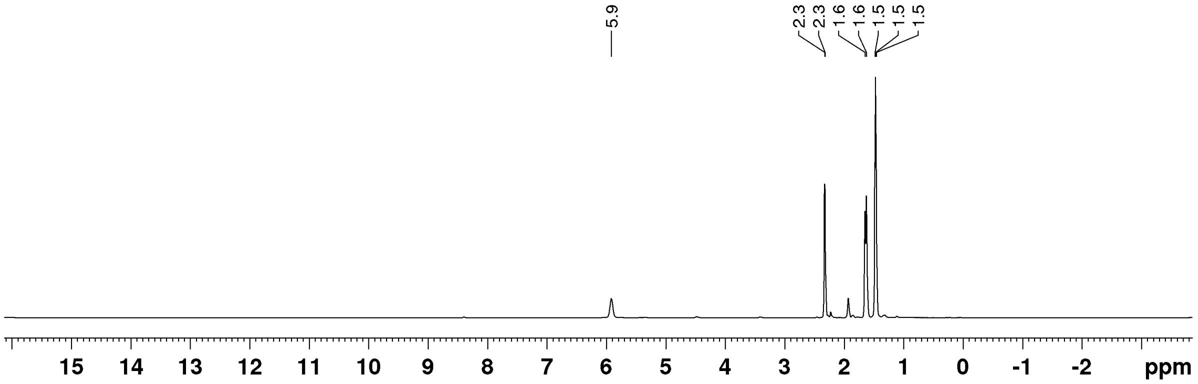

Figure S10. ^{1}H NMR spectrum of **1b**[OTf] (300 K, CD$_3$CN).

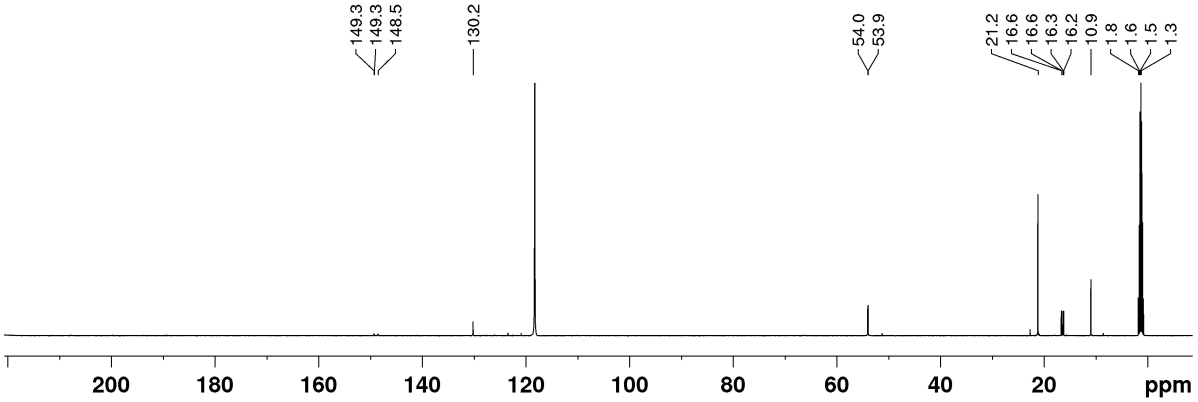

Figure S11. ^{13}C{^{1}H} NMR spectrum of **1b**[OTf] (300 K, CD$_3$CN).

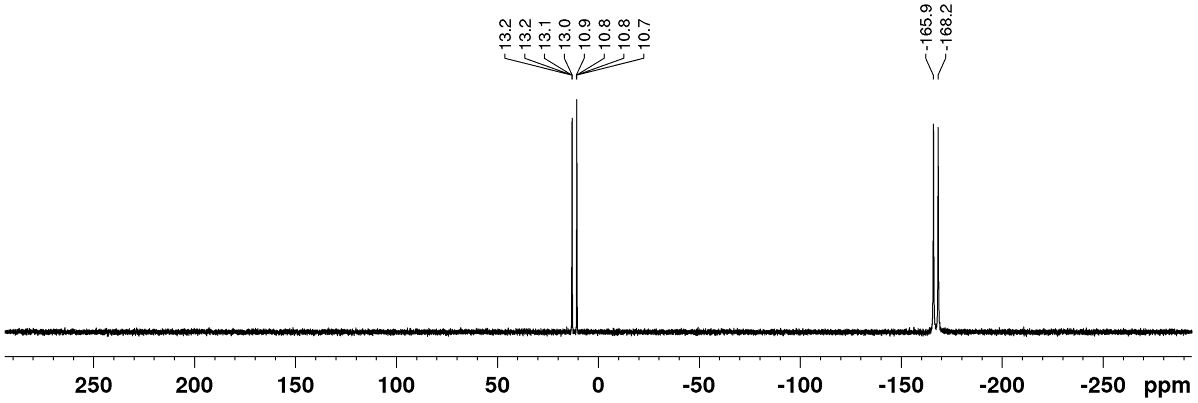

Figure S12. ^{31}P NMR spectrum of **1b**[OTf] (300 K, CD_3CN).

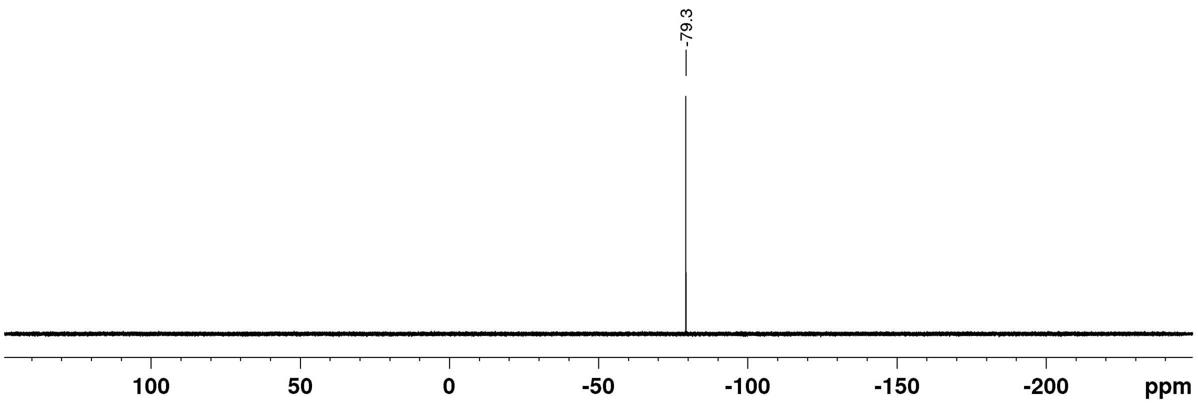

Figure S13. ^{19}F NMR spectrum of **1b**[OTf] (300 K, CD_3CN).

S2.10 Preparation of [(L_C)P–P(Et)$_3$][OTf] (1c[OTf])

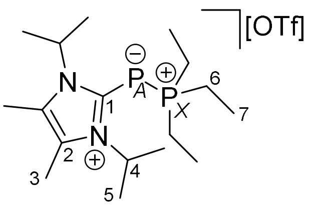

To a solution of **2**[OTf]$_4$ (1000 mg, 0.69 mmol, 1.0 eq.) in CH_3CN (5 ml) Et_3P (429 µl, 344 mg, 2.9 mmol, 4.2 eq.) was added dropwise while stirring. The resulting deep red solution was stirred for 16 h at room temperature giving a misty suspension, which was filtered and evaporated to dryness. Subsequent washing of the residue with Et_2O (3x4 mL) and consecutive drying *in vacuo* afforded the product as an air and moisture sensitive off-white solid. Crystals suitable for X-ray diffraction analysis were obtained by diffusion of Et_2O into a saturated CH_3CN solution at –30°C.

Yield: 1170 mg (88%); **m.p.**: 101-103 °C; **Raman** (80 mW, in cm^{-1}): 2986 (54), 2946 (96), 2918 (68), 2881 (39), 2730 (7), 1623 (28), 1449 (29), 1404 (30), 1350 (35), 1288 (100), 1150 (15), 1031 (46), 884 (12), 791 (13), 753 (19), 681 (12), 572 (14), 493 (100), 412 (13), 386 (16), 345 (29), 335 (36), 311 (23), 271 (24); **IR** (ATR, in cm^{-1}): 2975 (vw), 2942 (vw), 2881 (vw), 1622 (vw), 1453 (vw), 1419 (vw), 1371 (w), 1262 (vs), 1220 (m), 1156 (m), 1139 (s), 1114 (w), 1028 (vs), 976 (vw), 906 (w), 774 (m), 752 (m), 719 (w), 680 (vw), 658 (w), 635 (vs), 571 (m), 547 (w), 516 (m), 493 (w), 474 (w), 411 (w); **^{1}H NMR** (CD_3CN, 300 K, in ppm): δ = 1.17 (9H, dt, $^3J_{HP}$ = 17.6 Hz, $^3J_{HH}$ = 7.6 Hz, H7), 1.51 (12H, d, $^3J_{HH}$ = 7.1 Hz, H5), 1.89 (6H, dqd, $^2J_{HP}$ = 11.2 Hz, $^3J_{HH}$ = 7.6 Hz, $^3J_{HP}$ = 1.6 Hz, H6), 2.36 (6H, s, H3), 5.95 (2H, m, H4); **^{13}C{^{1}H} NMR** (CD_3CN, 300 K, in ppm): δ = 7.2 (3C, dd, $^1J_{CP}$ = 5 Hz, $^1J_{CP}$ = 4 Hz, 7C), 18.8 (3C, dd, $^1J_{CP}$ = 40 Hz, $^1J_{CP}$ = 9 Hz, 6C), 54.6 (2C, dd, $^3J_{CP}$ = 8 Hz, 4C), 122.6 (1C, q, $^1J_{CF}$ = 321 Hz, Otf), 149.6 (1C, dd, $^1J_{CP}$ = 98 Hz, $^2J_{CP}$ = 8 Hz, 1C); **^{19}F{^{1}H} NMR** (CD_3CN, 300 K, in ppm): δ = -79.3 (3F, s, OTf); **^{31}P{^{1}H} NMR** (CD_3CN, 300 K, in ppm): δ = -202.0 (1P, d, $^1J_{PP}$ = 492 Hz, P_A), 36.0 (1P, d, $^1J_{PP}$ = 492 Hz, P_X); **elemental analysis**: calcd. for $C_{18}H_{35}F_3N_2O_3P_2S$: C: 45.18, H: 7.37, N: 5.85, S: 6.70; found: C: 45.03, H: 7.44, N: 6.00, S: 6.62.

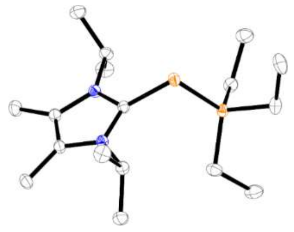

Figure S14. Molecular structure of **1c**$^+$ in **1c**[OTf]; hydrogen atoms and the anion are omitted for clarity and thermal ellipsoids are displayed at 50% probability.

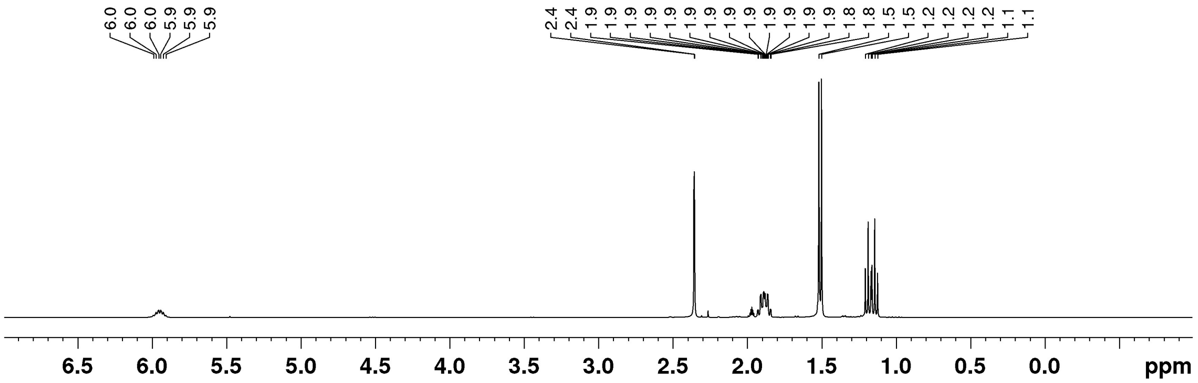

Figure S15. ^1H NMR spectrum of **1c**[OTf] (300 K, CD_3CN).

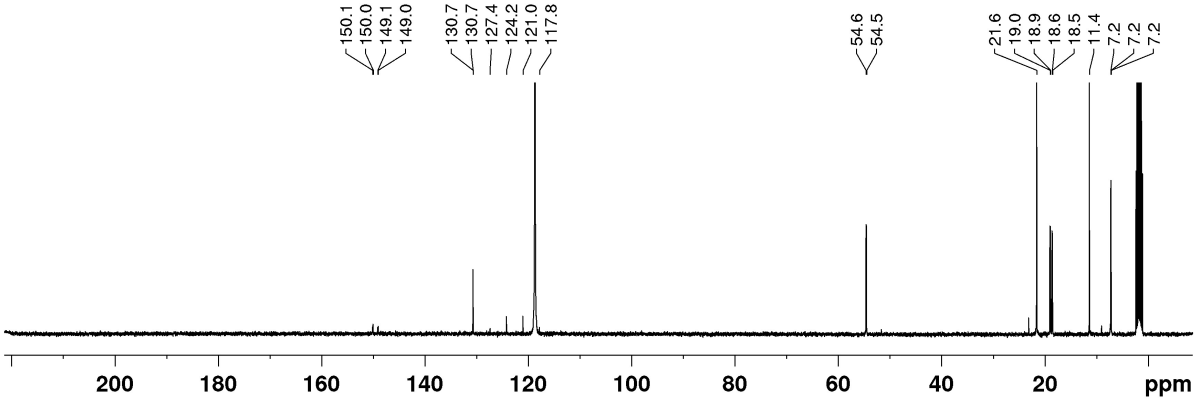

Figure S16. $^{13}C\{^1H\}$ NMR spectrum of **1c**[OTf] (300 K, CD_3CN).

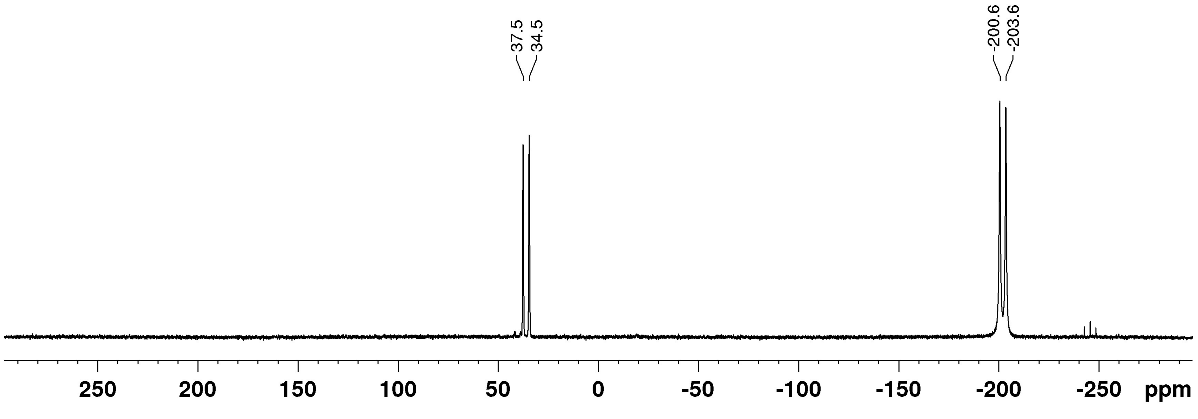

Figure S17. ^{31}P NMR spectrum of **1c**[OTf] (300 K, CD_3CN).

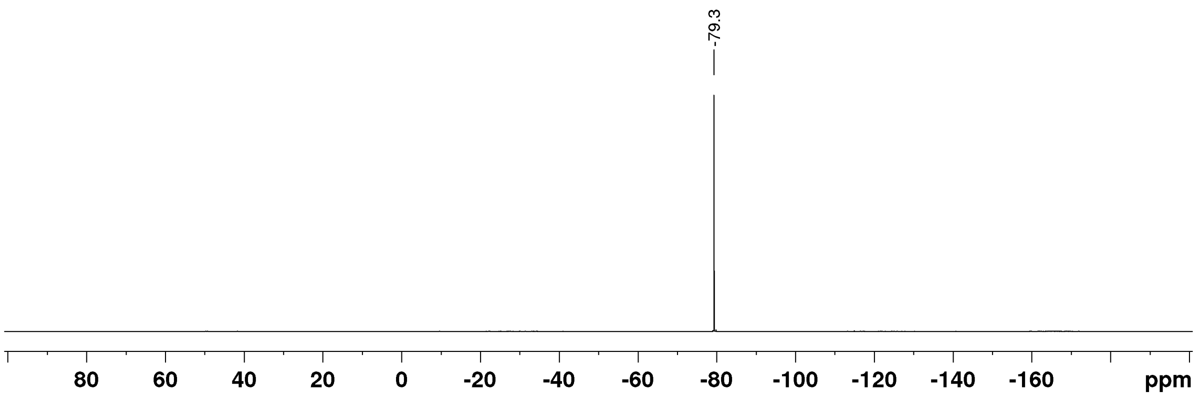

Figure S2. ^{19}F NMR spectrum of **1c**[OTf] (300 K, CD_3CN).

S2.11 Preparation of [(L_C)P–P(Cy)$_3$][OTf] (1d[OTf])

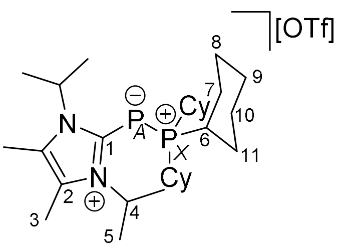

To a solution of **2**[OTf]$_4$ (250.0 mg, 0.17 mmol, 1.0 eq.) in CH_3CN (5 ml) freshly sublimed Cy_3P (200.0 mg, 0.71 mmol, 4.05 eq.) was added as a solid while stirring. The resulting suspension was stirred for 16 h at room temperature giving a clear orange colored solution, which was evaporated to dryness. Subsequent washing of the residue with Et_2O (3x4 mL) and consecutive drying *in vacuo* afforded the product as an air and moisture sensitive off-white solid. Crystals suitable for X-ray diffraction analysis were obtained by diffusion of Et_2O into a saturated CH_3CN solution at –30°C.

Yield: 412.3 mg (93%); **m.p.**: 165-167 °C; **Raman** (100 mW, in cm^{-1}): 2938 (100), 2896 (55), 2858 (67), 1623 (50), 1446 (66), 1402 (54), 1342 (58), 1286 (74), 1032 (61), 885 (37), 850 (38), 816 (41), 788 (37), 751 (38); **IR** (ATR, in cm^{-1}): 2982 (vw), 2927 (w), 2853 (w), 1624 (vw), 1445 (w), 1375 (w), 1342 (vw), 1263 (vs), 1221 (m), 1176 (w), 1137 (s), 1047 (vw), 1030 (vs), 1003 (w), 907 (w), 887 (w), 846 (w), 820 (vw), 789 (vw), 751 (w), 737 (w), 704 (vw), 635 (vs), 571 (w), 540 (m), 516 (m), 494 (w), 462 (w), 437 (vw), 423 (vw); **^{1}H NMR** (CD_3CN, 300 K, in ppm): δ = 1.23-1.37 (9H, m, $H8_{ax}$&$H9_{ax}$&$H10_{ax}$), 1.40-1.50 (6H, m, $H7_{ax}$&$H11_{ax}$), 1.54 (12H, d, $^3J_{HH}$ = 6.9 Hz, H5), 1.70-1.76 (3H, m, $H9_{eq}$), 1.83-1.90 (6H, m, $H8_{eq}$&$H10_{eq}$), 1.91-1.98 (6H, m, $H7_{eq}$&$H11_{eq}$), 2.14-2.24 (3H, m, $H6_{ax}$), 2.36 (6H, s, H3), 5.98 (2H, s (br), H4); **^{13}C{^{1}H} NMR** (CD_3CN, 300 K, in ppm): δ = 11.1 (2C, s, C3), 21.5 (4C, s, C5), 26.5 (3C, d, $^4J_{CP}$ = 1 Hz, C9), 27.7 (6C, d, $^3J_{CP}$ = 11 Hz, C8&C10), 28.5 (6C, pseudo-t, J_{CP} = 4 Hz, C7&C11), 36.5 (3C, dd, $^1J_{CP}$ = 31 Hz, $^2J_{CP}$ = 7 Hz, C6), 54.4 (2C, s, C4), 122.2 (1C, q, $^1J_{CF}$ = 321 Hz, OTf), 130.5 (2C, s, C2), 150.8 (1C, dd, $^1J_{CP}$ = 110 Hz, $^2J_{CP}$ = 11 Hz, C1); **^{19}F{^{1}H} NMR** (CD_3CN, 300 K, in ppm): δ = -79.3 (3F, s, OTf); **^{31}P{^{1}H} NMR** (CD_3CN, 300 K, in ppm): δ = -208.8 (1P, d, $^1J_{PP}$ = 545 Hz, P_A), 38.1 (1P, d, $^1J_{PP}$ = 545 Hz, P_X); **elemental analysis**: calcd. for $C_{30}H_{53}F_3N_2O_3P_2S$: C: 56.23, H: 8.34, N: 4.37, S: 5.00; found: C: 56.00, H: 8.272, N: 4.29, S: 4.591.

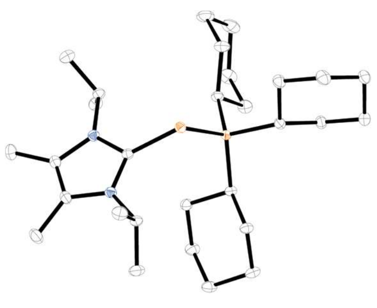

Figure S19. Molecular structure of **1d**$^+$ in **1d**[OTf]; hydrogen atoms and the anion are omitted for clarity and thermal ellipsoids are displayed at 50% probability.

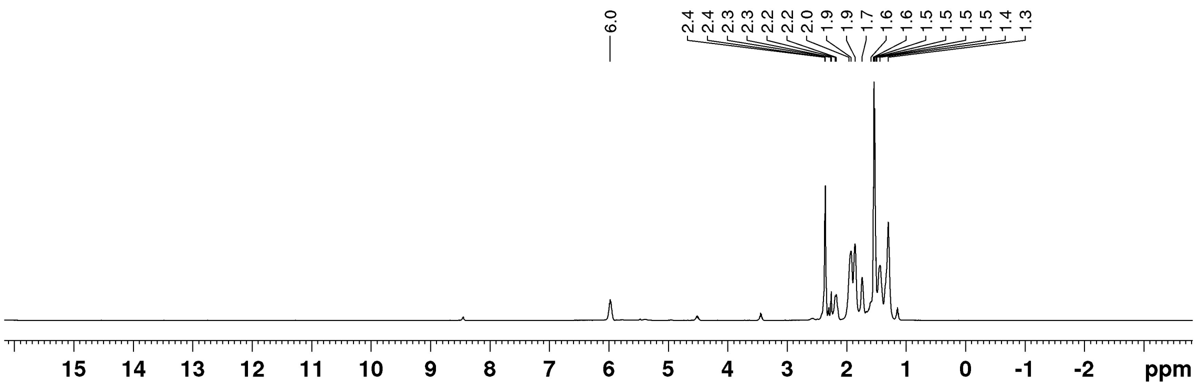

Figure S20. ^{1}H NMR spectrum of **1d**[OTf] (300 K, CD_3CN).

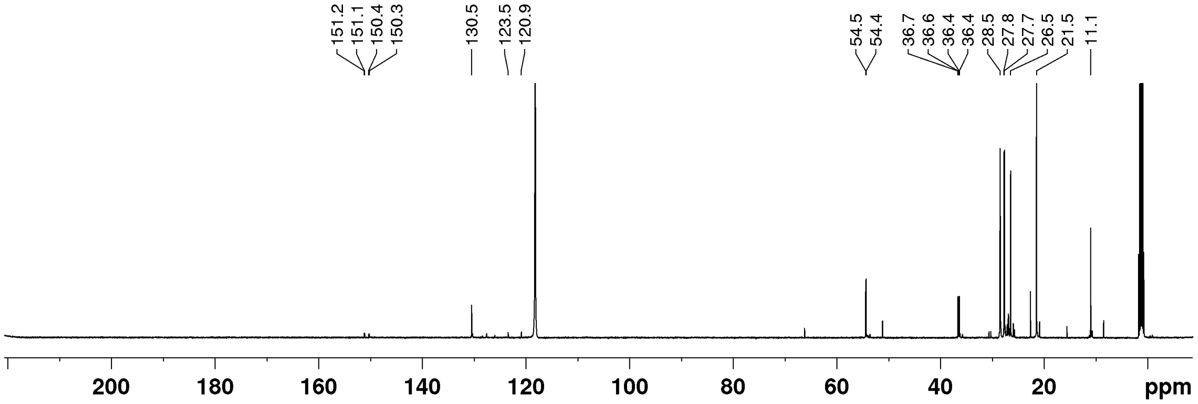

Figure S21. $^{13}C\{^{1}H\}$ NMR spectrum of **1d**[OTf] (300 K, CD_3CN).

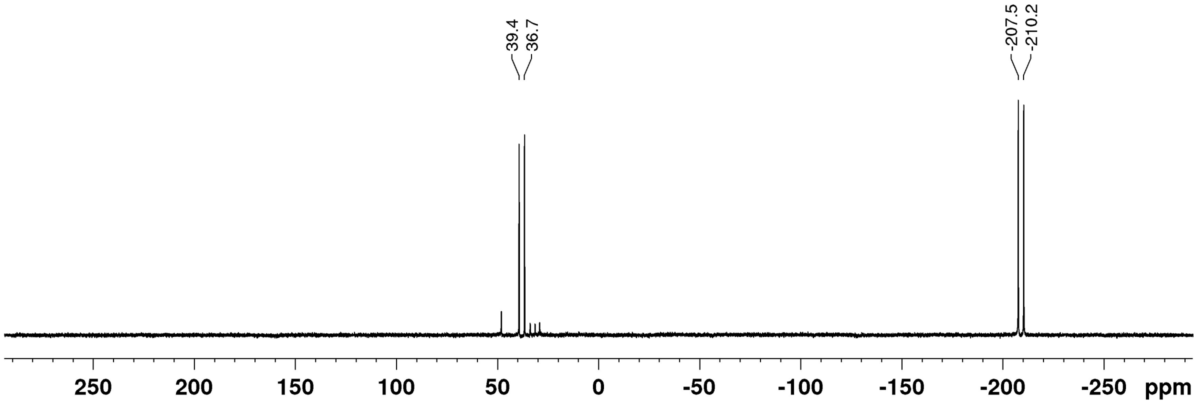

Figure S22. ^{31}P NMR spectrum of **1d**[OTf] (300 K, CD_3CN).

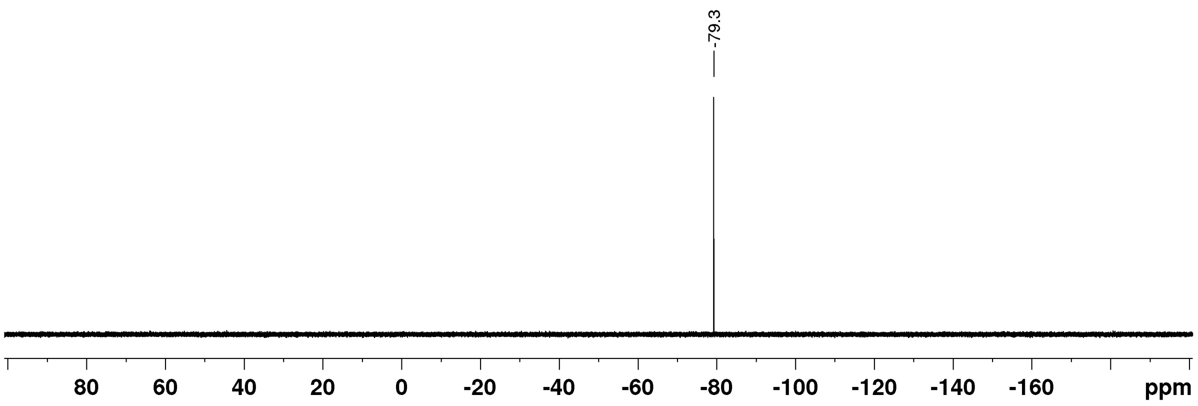

Figure S23. ^{19}F NMR spectrum of 1d[OTf] (300 K, CD_3CN).

S2.12 Preparation of $[(L_C)P{-}P(Ph)_3]^+$ (1a⁺)

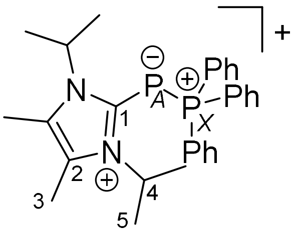

To a mixture of solid **2**[OTf]$_4$ (40 mg, 0.03 mmol, 1.0 eq.) and Ph_3P (29 mg, 0.11 mmol, 4.0 eq.) CD_3CN (1 ml) was added. The resulting yellow solution was stirred for 16 h at room temperature and subsequently investigated using multinuclear NMR experiments (see **Figure S24**).

$^{31}P\{^1H\}$ NMR (CD_3CN, 300 K, in ppm): δ = -168.7 (1P, d, $^1J_{PP}$ = 516 Hz, P_A), 31.3 (1P, d, $^1J_{PP}$ = 519 Hz, P_X).

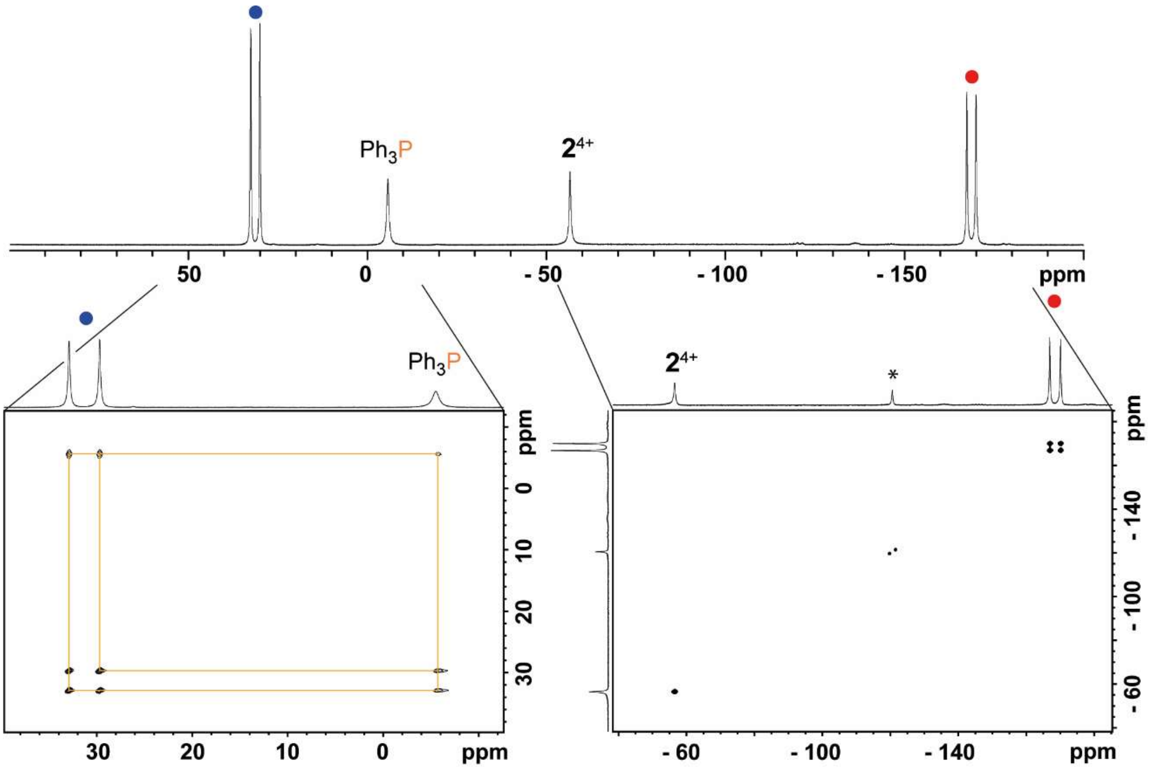

Figure S24. ^{31}P NMR spectrum of an aliquot of the reaction mixture of **2**[OTf]$_4$ with four equivalents Ph_3P in CD_3CN after 16 h at room temperature (top, CD_3CN, 300 K) and zoom in of a ^{31}P–^{31}P-EXSY NMR spectrum (bottom, CD_3CN, 300 K) showing spin polarisation exchange between the phosphonio moiety in **1a**⁺ and Ph_3P; no exchange between the phosphanide moiety and **2**[OTf]$_4$ is observed.

S2.13 Preparation of $[(L_C)P{-}P(Ph_2CH_2PPh_2)][OTf]$ (1e[OTf])

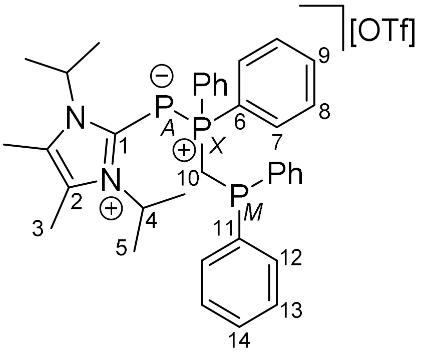

To a solution of **2**[OTf]$_4$ (250.0 mg, 0.17 mmol, 1.0 eq.) in CH_2Cl_2 (3 ml) a solution of bis(diphenylphosphino)methan (300 mg, 0.78 mmol, 4.5 eq.) in CH_2Cl_2 (2 ml) was added while stirring. The resulting slightly yellow reaction mixture was allowed to stir for 4 d at room temperature. Addition of Et_2O (10 ml) gives a precipitate, which was filtered off, washed with Et_2O (3x4 mL) and consecutively dried *in vacuo* to afford a pale-yellow powder (412.3 mg). Analysis by means of multinuclear NMR spectroscopy (**Figure S25-S28**) revealed resonances, that could be assigned to **1e**⁺ next to the starting materials. We attribute this to an underlying thermodynamical equilibrium similar as observed in the formation of **1a**⁺. Crystals suitable for X-ray diffraction analysis were obtained by diffusion of Et_2O into a saturated CH_3CN solution at –30°C (**Figure S29**).

Yield: 412.3 mg (93%); **^{19}F{^{1}H}-NMR** (CD_3CN, 300 K, in ppm): δ = -79.3 (3F, s, OTf); **^{31}P{^{1}H}-NMR** (CD_3CN, 300 K, in ppm): δ = -164.3 (1P, dd, $^{1}J_{PP}$ = -519 Hz, $^{3}J_{PP}$ = 54 Hz, P_A), -29.7 (1P, d, $^{2}J_{PP}$ = 69 Hz, $^{3}J_{PP}$ = 54 Hz, P_M), 38.1 (1P, d, $^{1}J_{PP}$ = -519 Hz, $^{2}J_{PP}$ = 69 Hz, P_X).

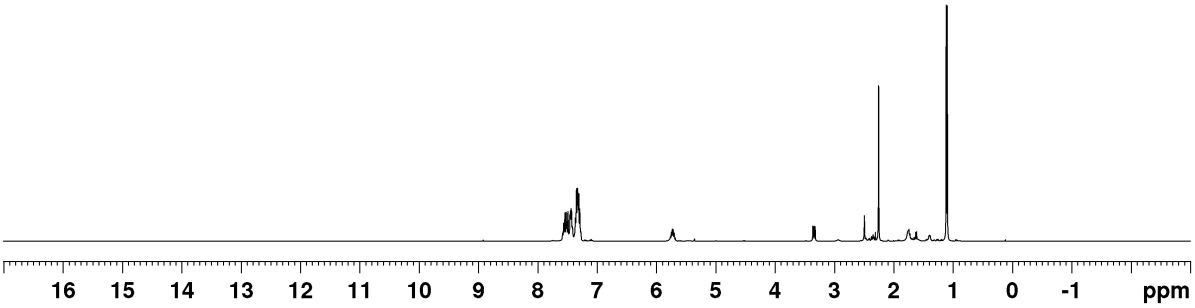

Figure S25. ^{1}H NMR spectrum of **1e**[OTf] (300 K, CD_3CN).

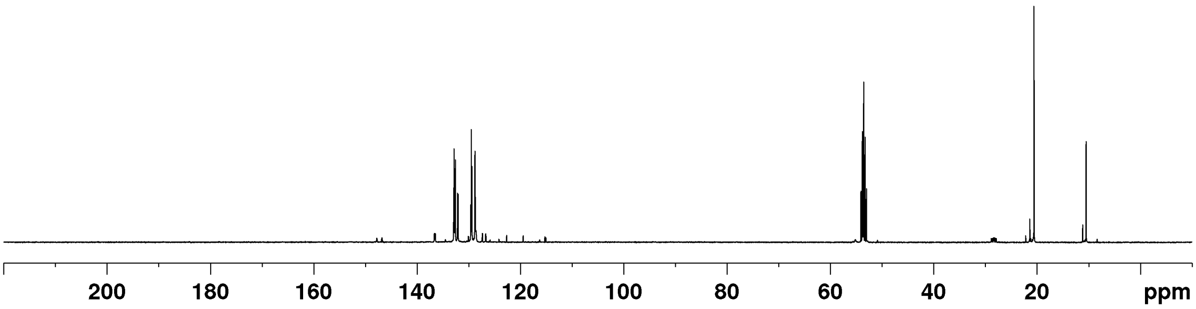

Figure S26. ^{13}C{^{1}H} NMR spectrum of **1e**[OTf] (300 K, CD_3CN).

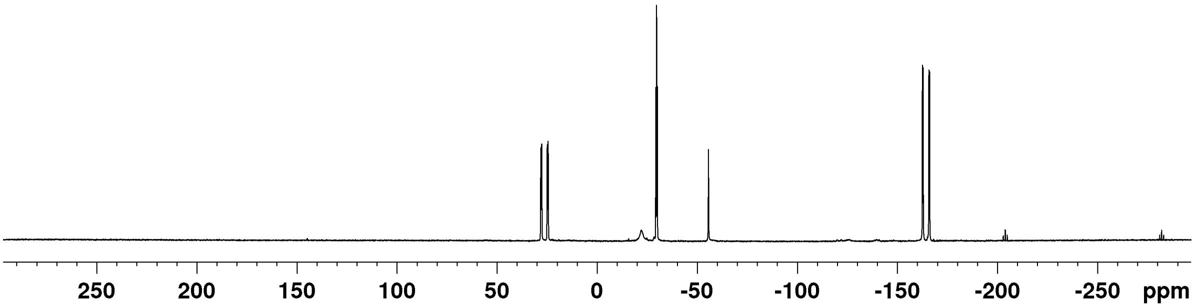

Figure S27. ^{31}P NMR spectrum of **1e**[OTf] (300 K, CD_3CN).

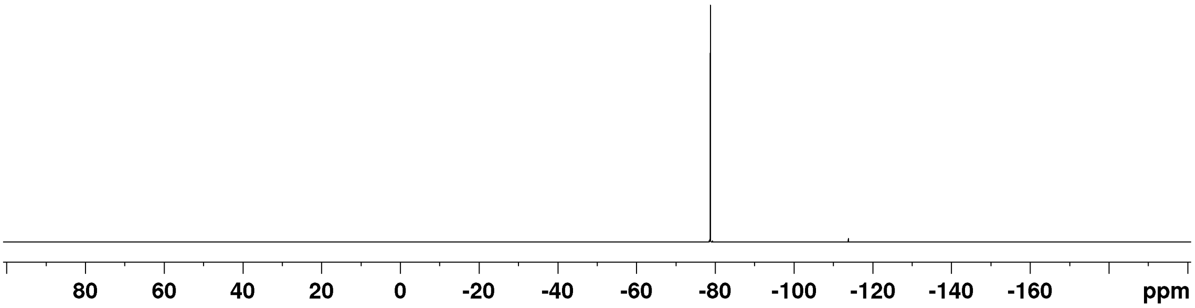

Figure S28. ^{19}F NMR spectrum of **1e**[OTf] (300 K, CD_3CN).

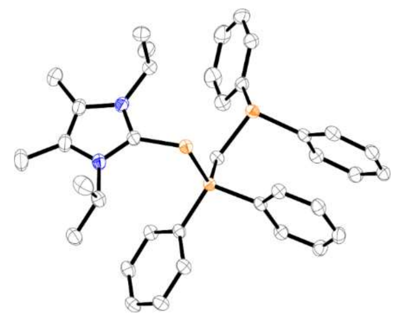

Figure S29. Molecular structure of **1e**$^{+}$ in **1e**[OTf]; hydrogen atoms and the anion are omitted for clarity and thermal ellipsoids are displayed at 50% probability.

S2.14 Reaction of 1b[OTf] with 4-methoxybenzaldehyde

A mixture of **1b**[OTf] (100 mg, 0.23 mmol, 1.0 eq.) and 4-methoxybenzaldehyde (156 mg, 1.2 mmol, 5 eq.) in THF was stirred at 70 °C in a microwave reactor for 24 h. Analysis of an aliquot of the mixture by ^{31}P NMR spectroscopy revealed the formation of O=PMe$_3$ [δ(^{31}P) = 36.2 ppm] and Me$_3$P [δ(^{31}P) = –61.5 ppm] (**Figure S30**). A third species was also formed [δ(^{31}P) = 178.3 ppm] in small amounts, which we assign to phosphaalkene [(L$_C$)P=CH(4-OMePh)]$^{+}$. The isolation of this compound has been unsuccessful thus far due to its low quantity.

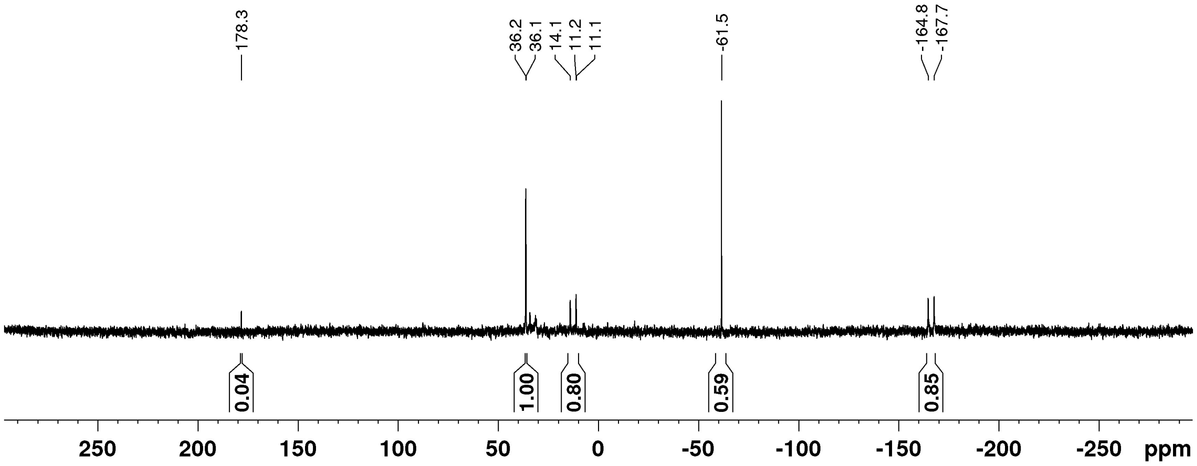

Figure S30. ^{31}P NMR spectrum of the reaction mixture of **1b**[OTf] and 4-methoxybenzaldehyde after 12 h at 70°C (300K, THF, C_6D_6 capillary).

S2.15 General procedures of the one-pot reaction for the formation of 5a-e[OTf] and 6a-d[OTf]

We further attempted a one-pot reaction of **3**[OTf] with thiocarbonyls in the presence of Ph$_3$P and zinc dust.[15]

Method: A mixture of **3**[OTf] (50 mg, 0.12 mmol, 1.0 eq; for **5a,b**[OTf]: 200 mg, 0.46 mmol, 1.0 eq), triphenylphosphane Ph$_3$P (31 mg, 0.12 mmol, 1.0 eq; for **5a,b**[OTf]: 122 mg, 0.46 mmol, 1.0 eq) and the respective thiocarbonyl (0.12 mmol, 1.0 eq; for **5a,b**[OTf]: 0.46 mmol, 1.0 eq) in CH$_3$CN (2 ml; for **5a,b**[OTf]: 4 ml) was added to solid zinc powder (8 mg, 0.12 mmol, 1.0 eq; for **5a,b**[OTf]: 30.4 mg, 0.46 mmol, 1.0 eq), stirred at room temperature for 16 h (for **5e**[OTf] and **6c**[OTf]: 80°C for 3 h in a microwave reactor) and analyzed by means of multinuclear NMR spectroscopy. Consequently, the volume of the mixture was reduced to ~1 mL *in vacuo*, filtered and 15 mL of Et$_2$O were added resulting in a precipitate, which was separated, washed three times with 4 mL of Et$_2$O and dried *in vacuo*. The resulting solids or oily residues were again investigated using multinuclear NMR spectroscopy (**Figure S31**).

Attempts to isolate the title compounds by precipitation from the reaction mixture with Et_2O and subsequent washing of the precipitate with Et_2O (3x4 ml) was only successful for **5a,b**[OTf] (90% and 88%, respectively). In other cases, viscous oils of impure products are obtained. Compounds **5f**[OTf] and **6d**[OTf] could not be synthesized using this one-pot method even at elevated temperatures. Crystals obtained from vapor diffusion of *n*-pentane in to a filtered THF solution of the crude product obtained in the attempted synthesis of **6d**[OTf] revealed the literature-known molecular structure of **24** (**Figure S32, Scheme S4**).[16] Next to this, the precipitation of **2**[OTf]$_4$ was observed during this conversion.

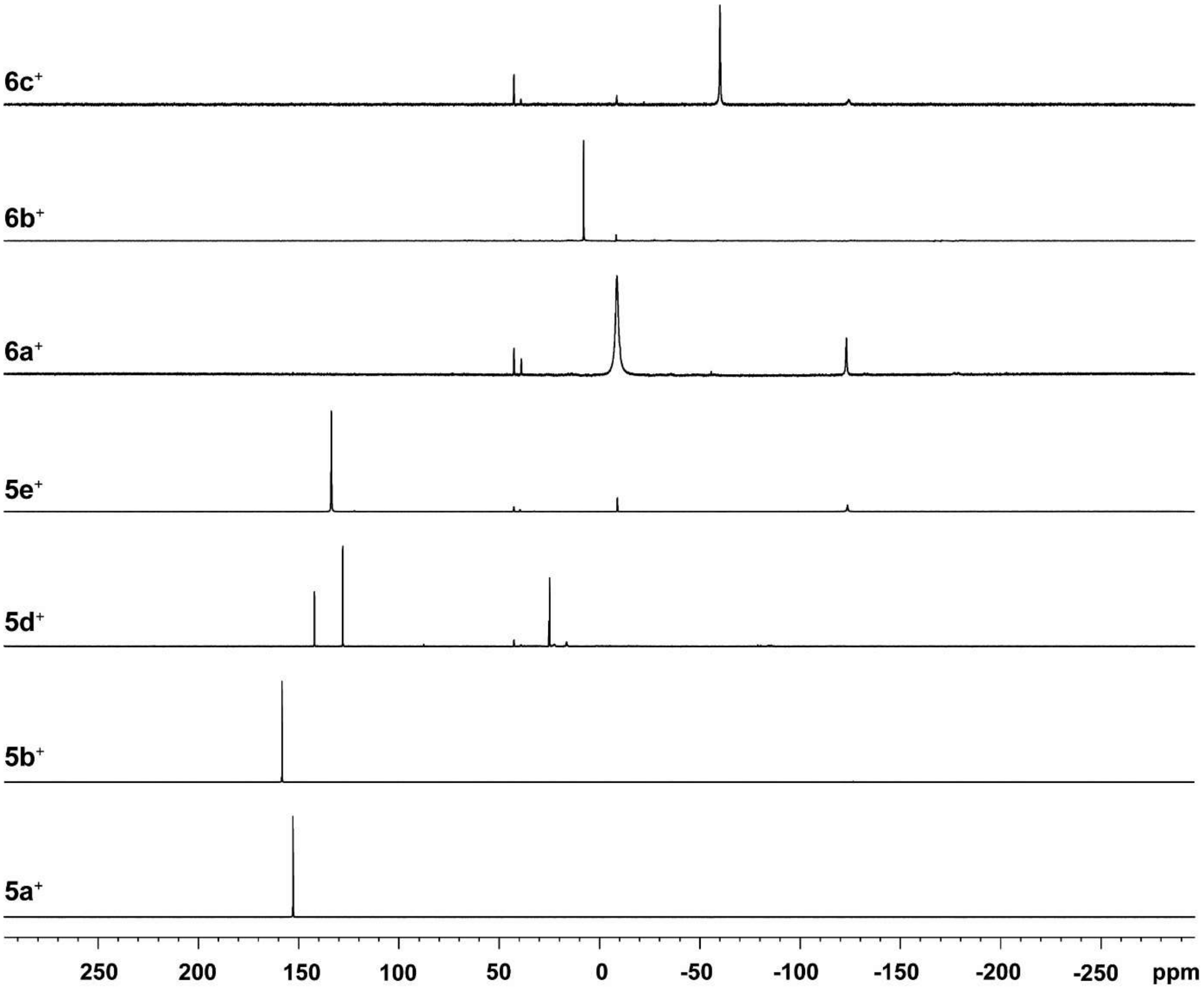

Figure S31. ^{31}P NMR spectra of residues obtained from the one pot reactions towards the formation of **5a,b,d,e**[OTf] and **6a-c**[OTf] (300 K, CD_3CN).

Scheme S4. Proposed reaction sequence for the reaction sequence of the one pot reaction of 3[OTf] with L$_C$=S in the presence of Ph$_3$P and Zn dust.

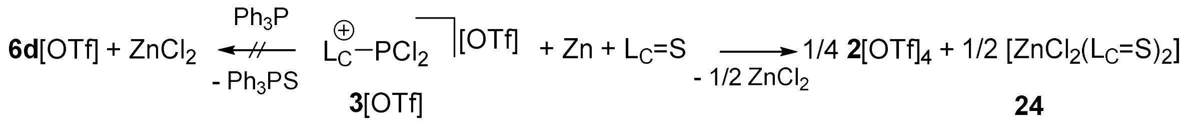

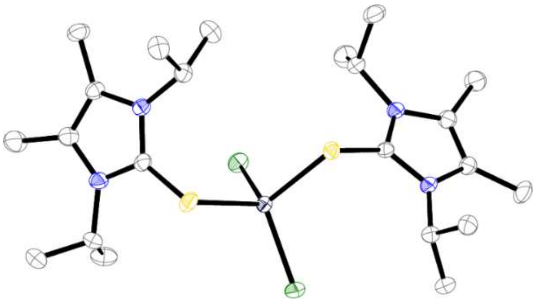

Figure S32. Molecular structure of **24**; hydrogen atoms and the anion are omitted for clarity and thermal ellipsoids are displayed at 50% probability.

S2.16 Preparation of [(L$_C$)P=C(Ph)$_2$][OTf] (5a[OTf])

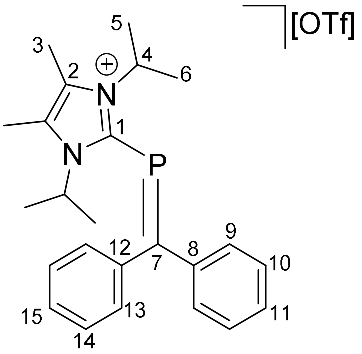

To a mixture of **2**[OTf]$_4$ (1000 mg, 0.69 mmol, 1.0 eq.) and Ph_3P (765 mg, 2.92 mmol, 4.2 eq.) in CH_3CN (5 ml) a solution of thiobenzophenone (578 mg, 2.92 mmol, 4.2 eq.) in CH_3CN (5 ml) was added and the mixture was stirred for 16 h at room temperature. The volume of the resulting yellow suspension was reduced to 2 ml *in vacuo* before it was filtered. Addition 15 mL of Et_2O of to the filtrate gives a yellow solid, which is again filtered off, washed three times with Et_2O (3x4 ml) and dried *in vacuo* to afford the product as an air and moisture sensitive yellow solid. Crystals suitable for X-ray diffraction analysis were obtained by diffusion of Et_2O into a saturated C_6H_5F solution at –30°C.

Yield: 1.332 g (91%); **m.p.**: 139-141 °C; **Raman** (100 mW, in cm^{-1}): 3066 (14), 2932 (15), 1622 (13), 1590 (76), 1486 (21), 1449 (21), 1433 (19), 1400 (19), 1285 (44), 1265 (36), 1218 (100), 1184 (22), 1036 (32), 998 (35), 688 (15), 513 (18), 408 (15); **IR** (ATR, in cm^{-1}): 3056 (vw), 2971 (vw), 2933 (vw), 2878 (vw), 1621 (vw), 1466 (vw), 1448 (w), 1399 (w), 1376 (w), 1265 (vs), 1220 (m), 1133 (s), 1110 (m), 1091 (w), 1031 (s), 1011 (w), 982 (vw), 938 (vw), 922 (vw), 904 (vw), 834 (vw), 786 (vw), 763 (m), 753 (m), 694 (s), 634 (vs), 599 (w), 571 (m), 516 (m), 469 (w), 457 (w), 444 (w), 414 (w); **^{1}H NMR** (CD_3CN, 300 K, in ppm): δ = 1.22 (6H, d, $^3J_{HH}$ = 6.4 Hz, H5), 1.52 (6H, d, $^3J_{HH}$ = 6.8 Hz, H6), 2.31 (6H, s, H3), 4.91 (2H, m, H4), 7.01 (2H, m, H13), 7.44 (1H, m, H15), 7.51 (4H, m, H10&H14), 7.55 (2H, m, H9), 7.63 (1H, m, H11); **^{13}C{^{1}H} NMR** (CD_3CN, 300 K, in ppm): δ = 10.8 (2C, s, C3), 20.8 (2C, s, C5), 21.7 (2C, s, C6), 54.7 (2C, d, $^3J_{CP}$ = 4 Hz, C4), 129.3 (2C, d, $^3J_{CP}$ = 20 Hz, C13), 129.9 (2C, s, C10&C14), 130.1 (2C, d, $^3J_{CP}$ = 6 Hz, C9), 131.2 (2C, s, C2), 131.5 (1C, s, C15), 143.0 (1C, d, $^2J_{CP}$ = 15 Hz, C12), 143.1 (1C, d, $^2J_{CP}$ = 30 Hz, C8), 145.4 (1C, d, $^1J_{CP}$ = 86 Hz, C1), 206.7 (1C, d, $^1J_{CP}$ = 46 Hz, C7); **^{19}F{^{1}H} NMR** (CD_3CN, 300 K, in ppm): δ = -79.2 (3F, s, OTf); **^{31}P{^{1}H} NMR** (CD_3CN, 300 K, in ppm): δ = 152.8 (1P, s, P); **elemental analysis**: calcd. for $C_{25}H_{30}F_3N_2O_3PS$: C: 57.03, H: 5.74, N: 5.32, S: 6.09; found: C: 56.98, H: 5.646, N: 5.33, S: 5.919.

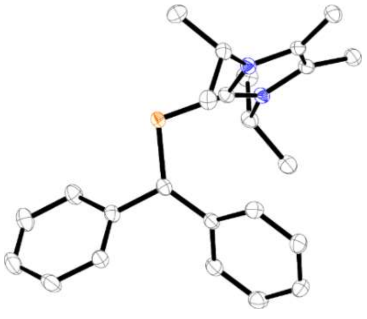

Figure S33. Molecular structure of **5a**$^+$ in **5a**[OTf]; hydrogen atoms and the anion are omitted for clarity and thermal ellipsoids are displayed at 50% probability.

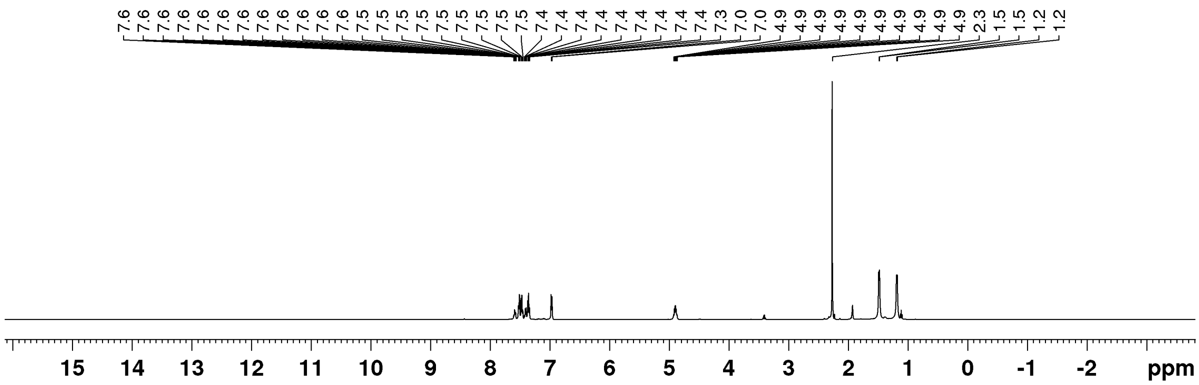

Figure S34. ^{1}H NMR spectrum of **5a**[OTf] (300 K, CD_3CN).

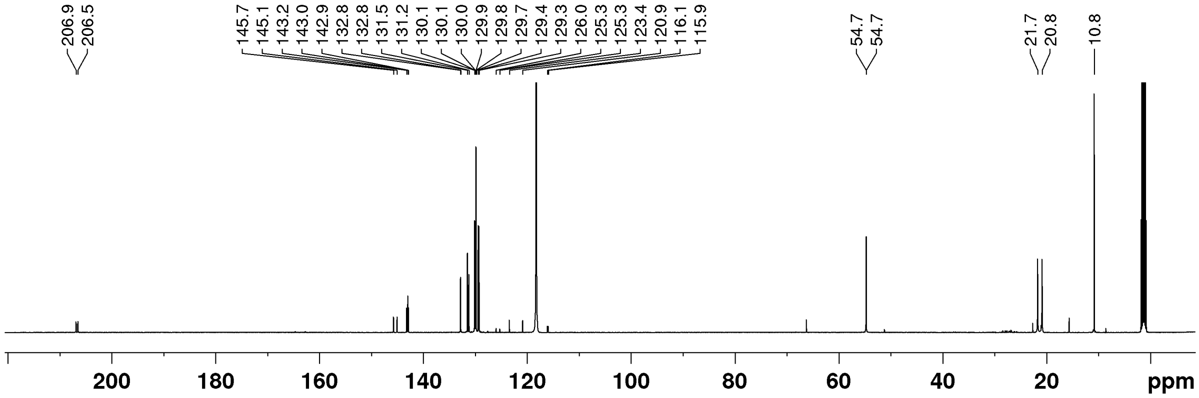

Figure S35. $^{13}C\{^{1}H\}$ NMR spectrum of **5a** [OTf] (300 K, CD_3CN).

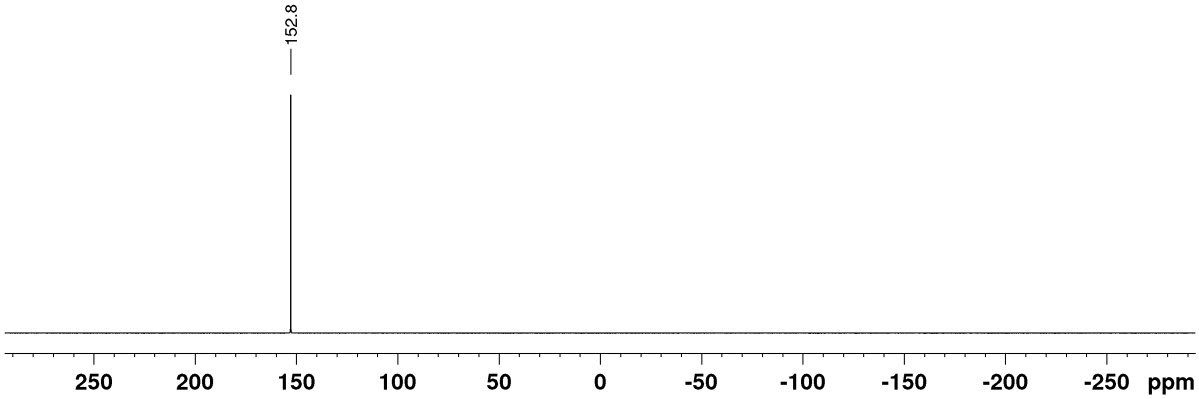

Figure S36. ^{31}P NMR spectrum of **5a** [OTf] (300 K, CD_3CN).

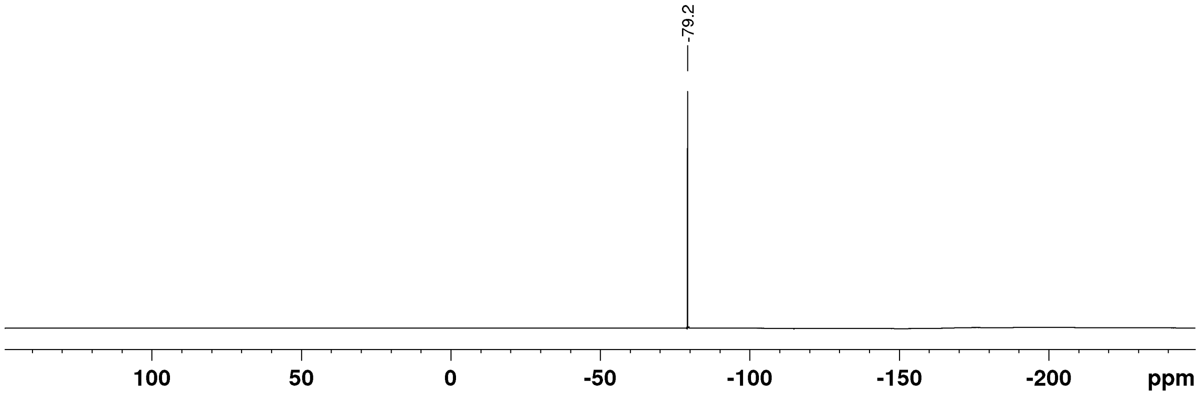

Figure S37. ^{19}F NMR spectrum of **5a** [OTf] (300 K, CD_3CN).

S2.17 Preparation of [(L$_C$)P=C(4,4'-Cl-Ph)$_2$][OTf] (5b[OTf])

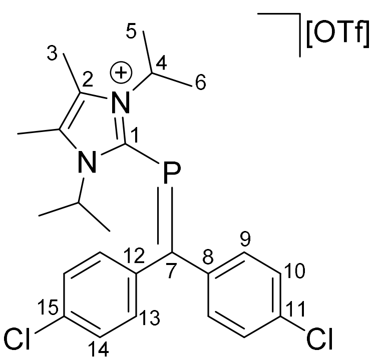

To a solution of **1d**[OTf] (250 mg, 0.39 mmol, 1.0 eq.) in THF (3 ml) a solution of 4,4'-dichlorothiobenzophenon (125 mg, 0.47 mmol, 1.2 eq.) in THF (5 ml) was added and the mixture was stirred for 16 h at room temperature. The resulting yellow precipitate was filtered off, washed thrice with cold (-30°C) THF (3x1 mL) and consequently extracted with C_6H_5F. Drying the extract *in vacuo* gives the analytically pure product as a yellow solid in good yields (124 mg, 53%). The yield can further be increased, when the THF mother liquor is cooled to -30°C after filtration affording yellow needles, which are filtered off, washed four times with Et_2O (4x2 mL) and dried *in vacuo* (61 mg, Σ = 185 mg, 80%).

The precipitate was washed four times with Et_2O and dried *in vacuo* to afford the product as an air and moisture sensitive yellow solid. Crystals suitable for X-ray diffraction analysis were obtained by layer diffusion of *n*-hexane into a saturated C_6H_5F solution at –30°C.

Yield: 185 mg (80%); **m.p.**: 222-224 °C; **Raman** (100 mW, in cm^{-1}): 3064 (5), 2991 (5), 2948 (10), 1614 (8), 1580 (100), 1486 (10), 1451 (9), 1423 (12), 1401 (11), 1373 (13), 1303 (20), 1288 (49), 1261 (14), 1214 (99), 1181 (30), 1152 (6), 1089 (34), 1031 (14), 1010 (6), 892 (5), 837 (6), 752 (5), 742 (16), 715 (5), 686 (7), 629 (7), 526 (9), 419 (10), 407 (10); **IR** (ATR, in cm^{-1}): 3083 (vw), 3054 (vw), 2989 (vw), 2922 (vw), 2850 (vw), 1614 (vw), 1579 (vw), 1485 (vw), 1447 (vw), 1418 (vw), 1399 (w), 1375 (vw), 1266 (vs), 1222 (m), 1178 (w), 1139 (s), 1113 (w), 1088 (m), 1030 (s), 1009 (w), 835 (w), 825 (w), 751 (w), 686 (vw), 635 (vs), 571 (w), 527 (w), 516 (w), 499 (vw), 477 (w), 419 (vw), 406 (vw); **^{1}H NMR** (CD_3CN, 300 K, in ppm): δ = 1.22 (6H, d, $^3J_{HH}$ = 6.8 Hz, H5), 1.49 (6H, d, $^3J_{HH}$ = 6.9 Hz, H6), 2.29 (6H, s, H3), 4.84 (2H, m, H4), 6.95 (2H, d, $^3J_{HH}$ = 8.5 Hz, H13), 7.39 (2H, d, $^3J_{HH}$ = 8.5 Hz, H14), 7.47-7.52 (4H, m, H9&H10); **^{13}C{^{1}H} NMR** (CD_3CN, 300 K, in ppm): δ = 10.8 (2C, s, C3), 20.9 (2C, s, C5), 21.7 (2C, s, C6), 54.8 (2C, d, $^3J_{CP}$ = 4 Hz, C4), 121.2 (1C, q, $^1J_{CF}$ = 321 Hz, OTf), 130.0 (2C, s, C10), 130.1 (2C, s, C14), 130.9 (2C, d, $^3J_{CP}$ = 20 Hz, C9), 131.6 (2C, s, C2), 131.7 (2C, d, $^3J_{CP}$ = 6 Hz, C13), 137.2 (1C, s, C15), 138.6 (1C, d, $^5J_{CP}$ = 5 Hz, C11), 141.2 (1C, d, $^2J_{CP}$ = 36 Hz, C12), 141.4 (1C, s, C8), 144.5 (1C, d, $^1J_{CP}$ = 86 Hz, C1), 203.0 (1C, d, $^1J_{CP}$ = 46 Hz, C7); **^{19}F{^{1}H} NMR** (CD_3CN, 300 K, in ppm): δ = -79.2 (3F, s, OTf); **^{31}P{^{1}H} NMR** (CD_3CN, 300 K, in ppm): δ = 158.2 (1P, s, P); **elemental analysis**: calcd. for $C_{25}H_{28}F_3N_2O_3PSCl_2$: C: 50.43, H: 4.74, N: 4.70, S: 5.38; found: C: 50.03, H: 4.956, N: 4.58, S: 6.064.

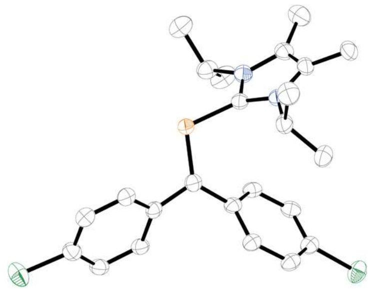

Figure S38. Molecular structure of **5b**$^+$ in **5b**[OTf]; hydrogen atoms and the anion are omitted for clarity and thermal ellipsoids are displayed at 50% probability.

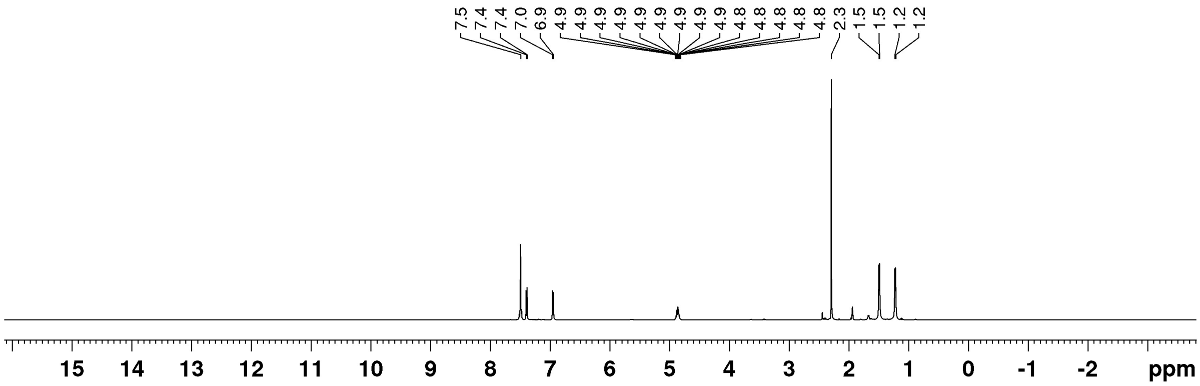

Figure S39. ^{1}H NMR spectrum of **5b**[OTf] (300 K, CD_3CN).

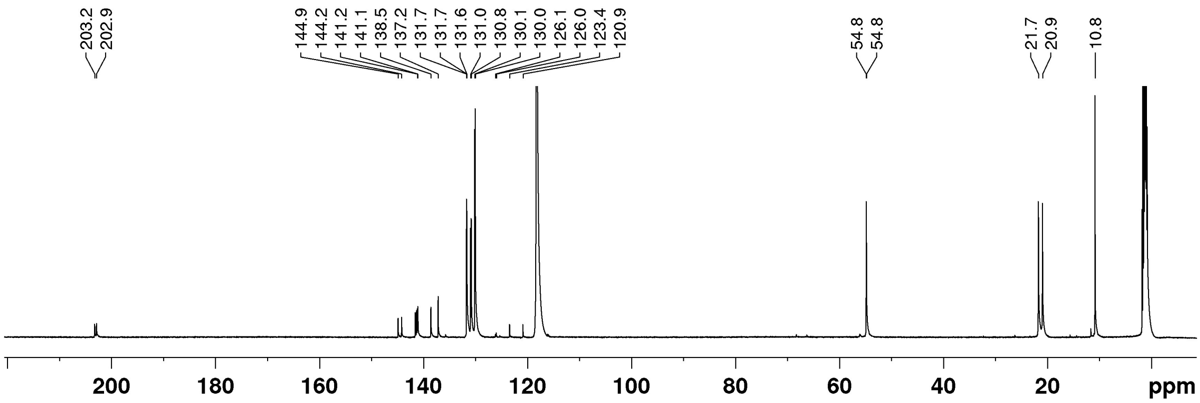

Figure S40. $^{13}C\{^{1}H\}$ NMR spectrum of **5b**[OTf] (300 K, CD_3CN).

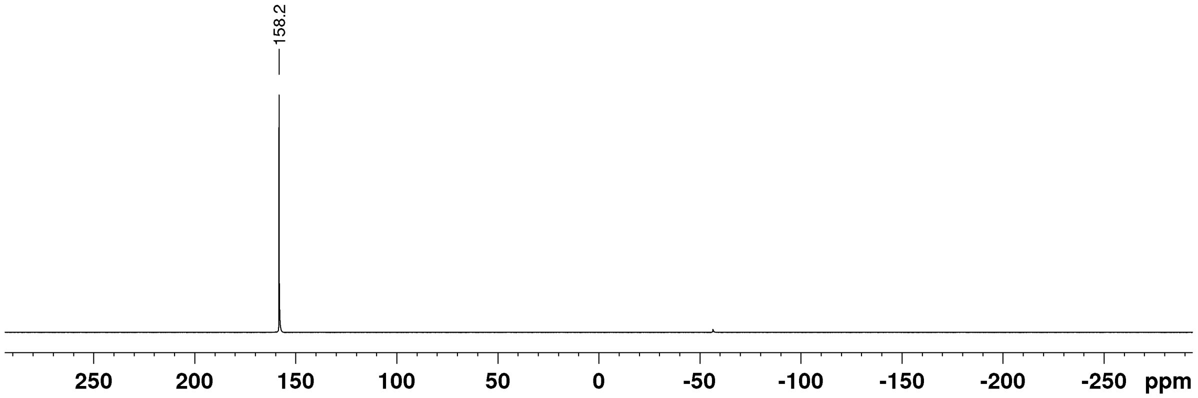

Figure S41. ^{31}P NMR spectrum of **5b**[OTf] (300 K, CD_3CN).

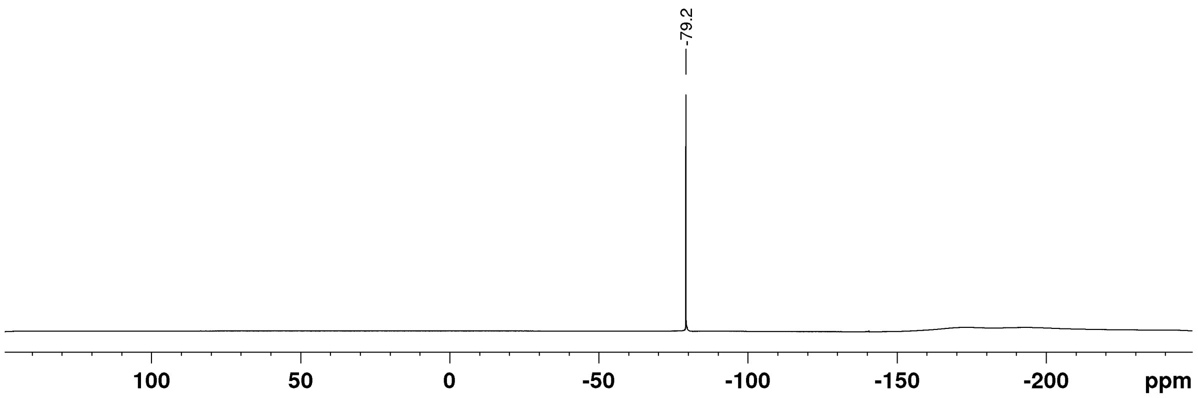

Figure S42. ^{19}F NMR spectrum of **5b**[OTf] (300 K, CD_3CN).

S2.18 Preparation of [(L_C)P=C(Ph-*p*-N(Me)$_2$)$_2$][OTf] (5c[OTf])

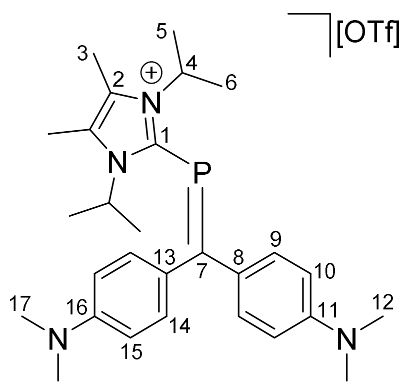

To a solution of **1d**[OTf] (250 mg, 0.39 mmol, 1.0 eq.) in THF (10 ml) a solution of Michler's Thion (126.6 mg, 0.47 mmol, 1.2 eq.) in THF (5 ml) was added and the mixture was stirred for 16 h at room temperature. The resulting red solution was evaporated to dryness *in vacuo,* washed three times with 4 mL of Et_2O and dried *in vacuo*. Twofold recrystallization from C_6H_5F /Et_2O at -30°C afforded the crystalline product as an air and moisture sensitive red solid. Crystals suitable for X-ray diffraction analysis were obtained by diffusion of Et_2O into a saturated C_6H_5F solution at –30°C.

Yield: 212.5 mg (89%); **m.p.**: 101-103 °C; **Raman** (100 mW, in cm^{-1}): 2938 (15), 1600 (100), 1524 (35), 1446 (20), 1418 (19), 1361 (27), 1307 (39), 1283 (34), 1238 (52), 1189 (89), 1017 (23), 762 (23), 726 (23), 599 (14), 536 (13), 426 (14); **IR** (ATR, in cm^{-1}): 2979 (vw), 2899 (vw), 2811 (vw), 2360 (vw), 1591 (vs), 1521 (w), 1445 (w), 1368 (m), 1306 (w), 1264 (vs), 1235 (m), 1221 (m), 1187 (s), 1171 (s), 1142 (s), 1082 (w), 1064 (w), 1030 (s), 1003 (w), 991 (w), 944 (m), 820 (m), 803 (w), 755 (m), 725 (w), 636 (vs), 596 (w), 571 (w), 540 (w), 516 (m), 412 (w); **^{1}H NMR** (CD_3CN, 300 K, in ppm): δ = 1.21 (6H, d, $^3J_{HH}$ = 6.9 Hz, H6), 1.46 (6H, d, $^3J_{HH}$ = 7.0 Hz, H5), 2.28 (6H, s, H3), 2.96 (6H, s, H17), 3.04 (6H, s, H12), 5.04 (2H, dsept, $^3J_{HH}$ = 6.9 Hz, $^4J_{HP}$ = 3.0 Hz, H4), 6.61 (2H, m, H15), 6.75 (2H, m, H10), 6.82 (2H, m, H14), 7.42 (2H, m, H9); **^{13}C{^{1}H} NMR** (CD_3CN, 300 K, in ppm): δ = 10.8 (2C, s, C3), 20.8 (2C, s, C6), 21.6 (2C, s, C5), 40.3 (2C, s, C17), 40.4 (2C, s, C12), 54.1 (2C, d, $^3J_{CP}$ = 5 Hz, C4), 112.2 (2C, s, C15), 112.5 (2C, s, C10), 122.2 (1C, q, $^1J_{CF}$ = 321 Hz, OTf), 130.1 (2C, s, C2), 130.6 (1C, d, $^2J_{CP}$ = 13 Hz, C13), 130.9 (1C, d, $^2J_{CP}$ = 32 Hz, C8), 131.5 (2C, d, $^3J_{CP}$ = 20 Hz, C9), 132.8 (2C, d, $^3J_{CP}$ = 4 Hz, C14), 149.1 (1C, d, $^1J_{CP}$ = 90 Hz, C1), 153.2 (1C, s, C16), 154.6 (1C, d, $^5J_{CP}$ = 2 Hz, C11), 204.0 (1C, d, $^1J_{CP}$ = 51 Hz, C7); **^{19}F{^{1}H} NMR** (CD_3CN, 300 K, in ppm): δ = -79.2 (3F, s, OTf); **^{31}P{^{1}H} NMR** (CD_3CN, 300 K, in ppm): δ = 99.4 (1P, s, P); **elemental analysis**: calcd. for $C_{29}H_{40}F_3N_4O_3PS \cdot 0.33\ C_6H_4F$: C: 57.22, H: 6.40, N: 8.61, S: 4.93; found: C: 57.03, H: 6.356, N: 8.47, S: 4.980.

Note: residual C_6H_5F from recrystallization could not be removed even after drying *in vacuo* for 24h. Both, NMR data and elemental analysis are consistent with a chemical formula for the solvate of $C_{29}H_{40}F_3N_4O_3PS \cdot 0.33\ C_6H_5F$.

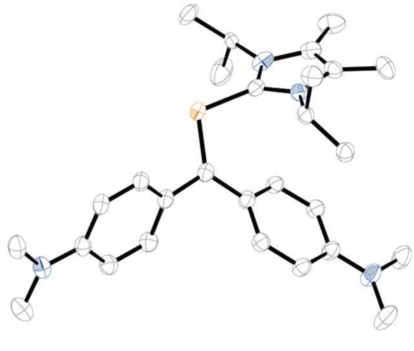

Figure S43. Molecular structure of **5c**$^+$ in **5c**[OTf] · $2C_6H_5F$; hydrogen atoms and the anion are omitted for clarity and thermal ellipsoids are displayed at 50% probability.

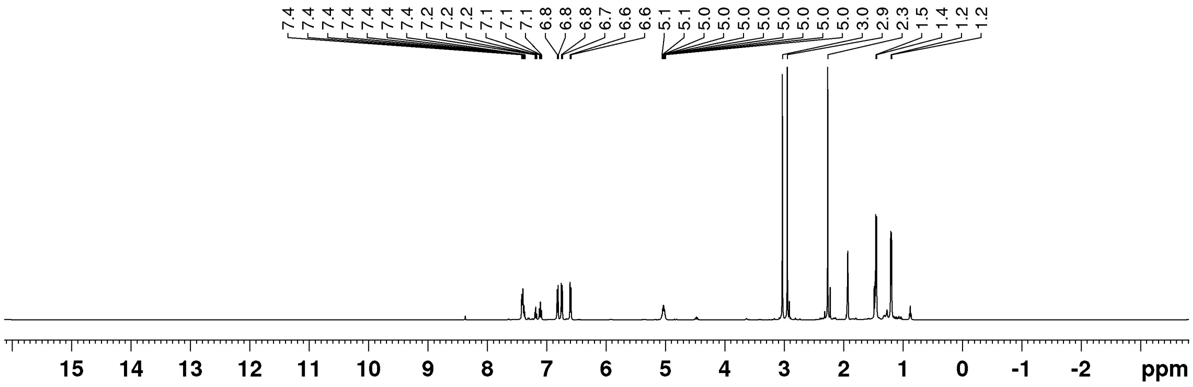

Figure S44. ^{1}H NMR spectrum of **5c**[OTf] (300 K, CD_3CN).

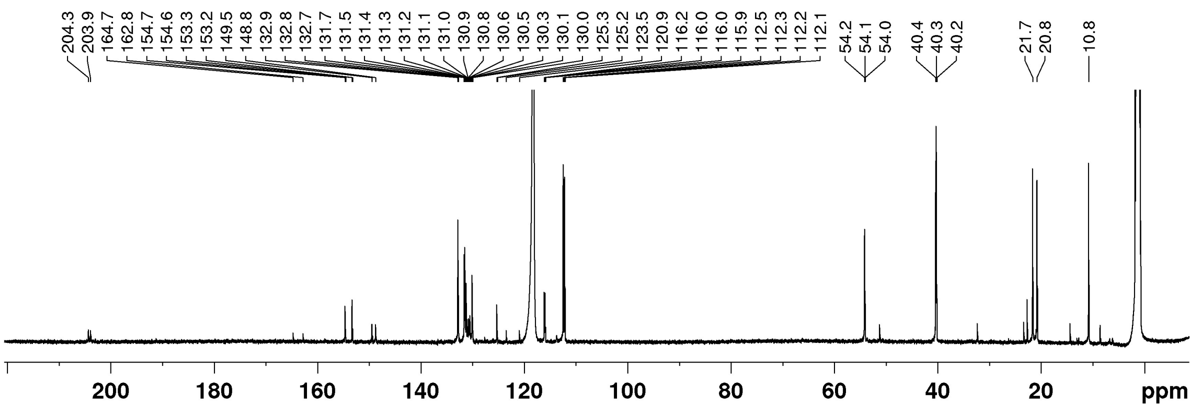

Figure S45. $^{13}C\{^{1}H\}$ NMR spectrum of **5c**[OTf] (300 K, CD_3CN).

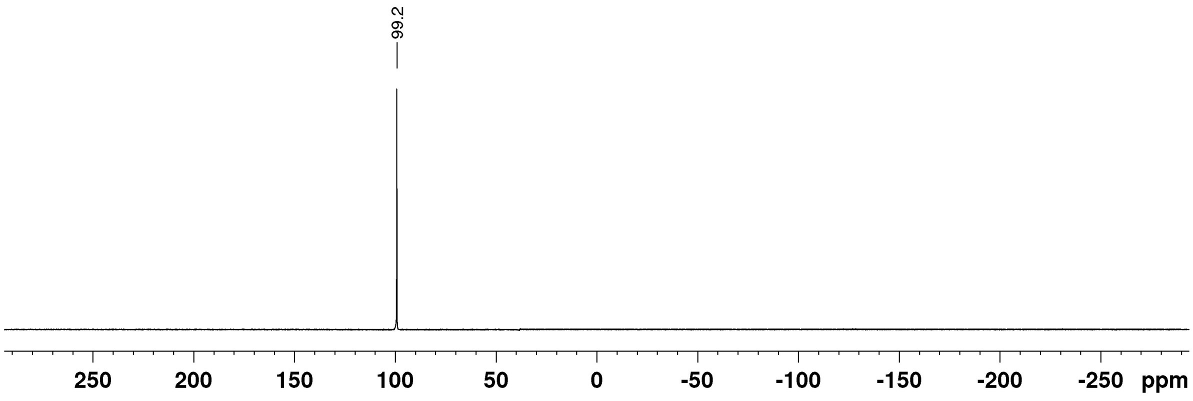

Figure S46. ^{31}P NMR spectrum of **5c**[OTf] (300 K, CD_3CN).

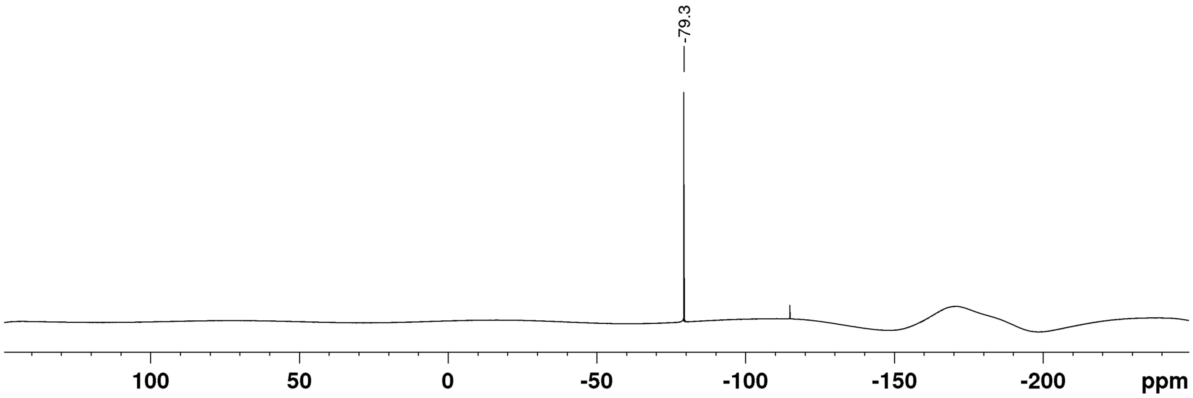

Figure S47. ^{19}F NMR spectrum of **5c**[OTf] (300 K, CD_3CN).

S2.19 Preparation of [(*E*/*Z*)-(L_C)P=C(Ph)-2-thiophenyl][OTf] (5d[OTf])

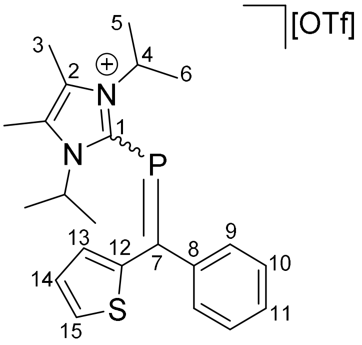

To a solution of **2**[OTf]$_4$ (150 mg, 0.1 mmol, 1.0 eq.) and Ph_3P (115 mg, 0.44 mmol, 4.2 eq.) in CH_3CN (4 ml) a deep blue solution of 2-thiophenyl(phenyl)thion (87 mg, 0.43 mmol, 4.1 eq.) in CH_3CN (1 ml) was added and the mixture was stirred for 16 h at room temperature. The resulting orange suspension was reduced to 2 mL *in vacuo* and filtered. Subsequent addition of 15 mL of Et_2O to the filtrate initially leads to the precipitation of a yellow oil, which turned into a yellow solid after vigorous stirring for 2 h, was filtered off, washed twice with 4 mL of Et_2O and dried *in vacuo* to afford the product as an air and moisture sensitive yellow solid. The ^{31}P NMR spectra of the obtained solid shows the presence of two resonances in an integral ratio of 1, which are assigned to the (E)- and (Z)- isomer. Crystals suitable for X-ray diffraction analysis were obtained by diffusion of Et_2O into a saturated C_6H_5F solution at –30°C.

Yield: 140 mg (63%); **m.p.**: 98-100 °C; **Raman** (100 mW, in cm^{-1}): 3072 (6), 2994 (5), 2969 (7), 2932 (10), 1623 (14), 1592 (34), 1504 (23), 1485 (14), 1449 (18), 1431 (21), 1407 (100), 1354 (53), 1317 (12), 1284 (36), 1267 (21), 1233 (20), 1215 (42), 1197 (22), 1190 (21), 1183 (21), 1113 (11), 1076 (16), 1057 (15), 1032 (23), 1002 (22), 987 (10), 961 (10), 889 (11), 842 (13), 788 (13), 752 (13), 739 (14), 672 (19), 623 (15), 613 (16), 561 (20), 506 (23), 406 (22), 376 (21), 346 (21), 310 (23), 152 (25), 93 (59); **IR** (ATR, in cm^{-1}): 3081 (vw), 2993 (vw), 2970 (vw), 2936 (vw), 2880 (vw), 1618 (w), 1465 (vw), 1446 (w), 1400 (m), 1376 (w), 1352 (w), 1262 (vs), 1134 (s), 1111 (m), 1056 (w), 1028 (vs), 1002 (w), 982 (w), 961 (vw), 939 (vw), 919 (vw), 904 (w), 862 (vw), 842 (w), 818 (w), 787 (vw), 756 (m), 696 (m), 671 (vw), 632 (vs), 567 (m), 512 (m), 454 (w), 411 (w); **^{1}H NMR** (CD_3CN, 300 K, in ppm): δ = 1.24 (6H, s(br), (E)-H5), 1.33 (6H, d, $^{3}J_{HH}$ = 7.0 Hz, (Z)-H5), 1.47 (6H, s(br), (E)-H6), 1.52 (6H, d, $^{3}J_{HH}$ = 7.0 Hz, (Z)-H6), 2.25 (3H, s, (Z)-H3), 2.40 (3H, s, (E)-H3), 4.96 (2H, m, (E)-H4), 4.96 (2H, m, (Z)-H4), 6.72 (1H, m, (Z)-H13), 7.09 (1H, dd, $^{3}J_{HH}$ = 5.5 Hz, $^{3}J_{HP}$ = 4.0 Hz, (E)-H13), 7.16 (1H, m, (E)-H9), 7.21 (1H, m, (Z)-H14), 7.34 (1H, *pseudo*-dt, $^{3}J_{HH}$ = 5.5 Hz, $^{3}J_{HH}$ = 5.4 Hz, $^{4}J_{HP}$ = 1.0 Hz, (E)-H14), 7.40 (2H, m, (E)-H10), 7.43 (2H, m, (E)-H11), 7.52 (2H, m, (Z)-H10), 7.62 (1H, m, (Z)-H9), 7.62 (2H, m, (Z)-H11), 7.71 (1H, ddd, $^{3}J_{HH}$ = 5.5 Hz, $^{4}J_{HP}$ = 2.2 Hz, $^{4}J_{HP}$ = 1.2 Hz, (Z)-H15), 7.74 (1H, *pseudo*-dt, $^{3}J_{HH}$ = 5.5 Hz, $^{4}J_{HH}$ = 1.3 Hz, $^{4}J_{HP}$ = 1.0 Hz, (E)-H15); **^{13}C{^{1}H} NMR** (CD_3CN, 300 K, in ppm): δ = 10.8 (2C, s, (Z)-C3), 10.8 (2C, s, (E)-C3), 20.9 (2C, s, (Z)-C5), 21.0 (2C, s, (E)-C5), 21.6 (2C, s, (E)-C6), 21.8 (2C, s, (Z)-C6), 54.8 (2C, d, $^{3}J_{CP}$ = 3 Hz, (Z)-C4), 54.9 (2C, d, $^{3}J_{CP}$ = 4 Hz, (E)-C4), 122.2 (1C, q, $^{1}J_{CF}$ = 321 Hz, OTf), 129.5 (1C, d, $^{3}J_{CP}$ = 4 Hz, (E)-C13), 129.7 (2C, s, (Z)-C10), 129.8 (2C, s, (E)-C9), 129.8 (2C, d, $^{4}J_{CP}$ = 6 Hz, (E)-C10), 130.3 (2C, d, $^{3}J_{CP}$ = 17 Hz, (Z)-C9), 130.3 (1C, d, $^{4}J_{CP}$ = 3 Hz, (Z)-C14), 131.2 (2C, s, (Z)-C2), 131.5 (1C, d, $^{4}J_{CP}$ = 22 Hz, (E)-C14), 131.6 (1C, s, (E)-C11), 132.0 (2C, s, (E)-C2), 132.4 (1C, d, $^{5}J_{CP}$ = 3 Hz, (Z)-C11), 133.4 (1C, d, $^{3}J_{CP}$ = 11 Hz, (Z)-C13), 133.5 (1C, d, $^{4}J_{CP}$ = 12 Hz, (E)-C15), 135.0 (1C, d, $^{4}J_{CP}$ = 6 Hz, (Z)-C15), 142.1 (1C, d, $^{2}J_{CP}$ = 13 Hz, (E)-C8), 142.3 (1C, d, $^{2}J_{CP}$ = 31 Hz, (Z)-C8), 144.3 (1C, d, $^{1}J_{CP}$ = 56 Hz, (E)-C1), 145.0 (1C, d, $^{1}J_{CP}$ = 50 Hz, (Z)-C1), 145.7 (1C, d, $^{2}J_{CP}$ = 19 Hz, (Z)-C12), 147.0 (1C, d, $^{2}J_{CP}$ = 35 Hz, (E)-C12), 193.9 (1C, d, $^{1}J_{CP}$ = 49 Hz, (Z)-C7), 196.6 (1C, d, $^{1}J_{CP}$ = 45 Hz, (E)-C7); **^{19}F{^{1}H} NMR** (CD_3CN, 300 K, in ppm): δ = -79.2 (3F, s, OTf); **^{31}P{^{1}H} NMR** (CD_3CN, 300 K, in ppm): δ = 127.9 (1P, s, (Z)-P), 142.1 (1P, s, (E)-P);

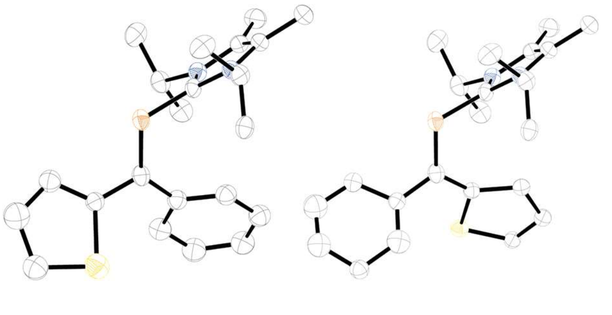

Figure S48. Molecular structure of *E*/*Z*-**5d**$^+$ in *E*/*Z*-**5d**[OTf]; hydrogen atoms and the anion are omitted for clarity and thermal ellipsoids are displayed at 50% probability.

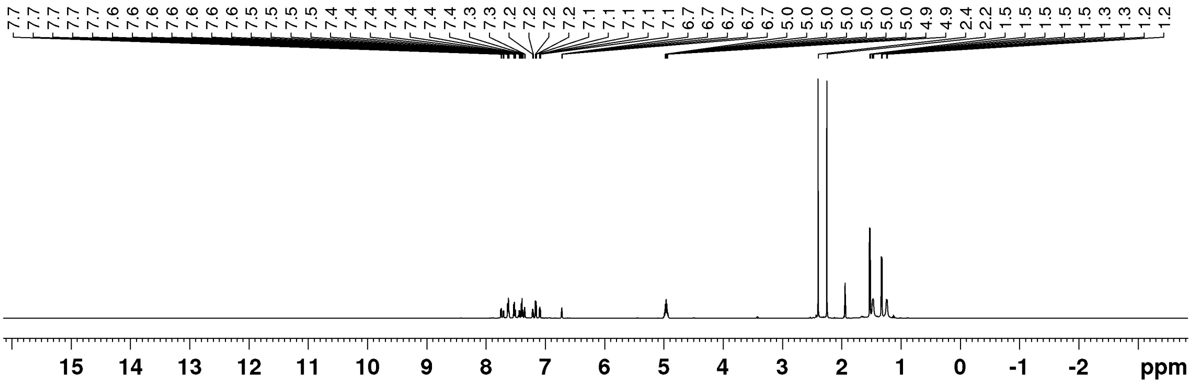

Figure S49. ^1H NMR spectrum of **5d**[OTf] (300 K, CD_3CN).

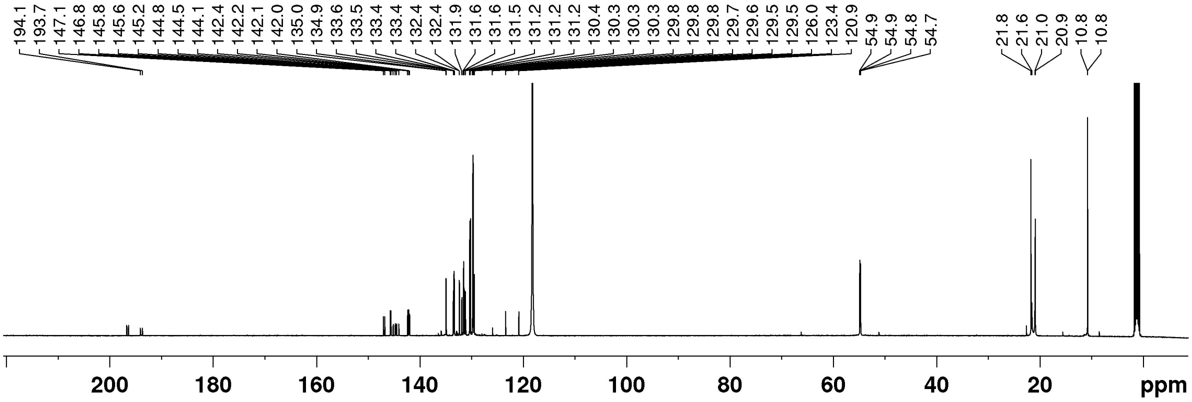

Figure S50. $^{13}C\{^1H\}$ NMR spectrum of **5d**[OTf] (300 K, CD_3CN).

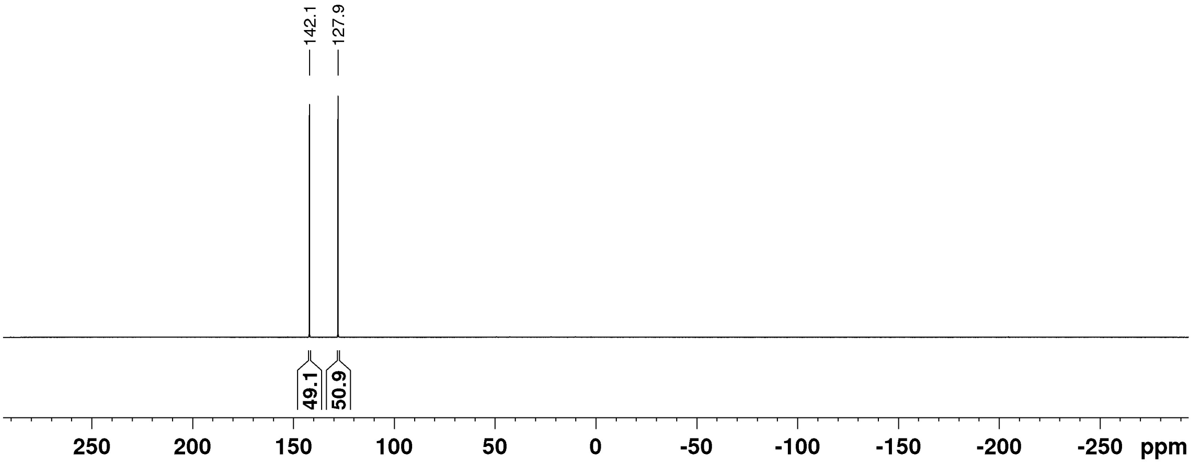

Figure S51. ^{31}P NMR spectrum of **5d**[OTf] (300 K, CD_3CN).

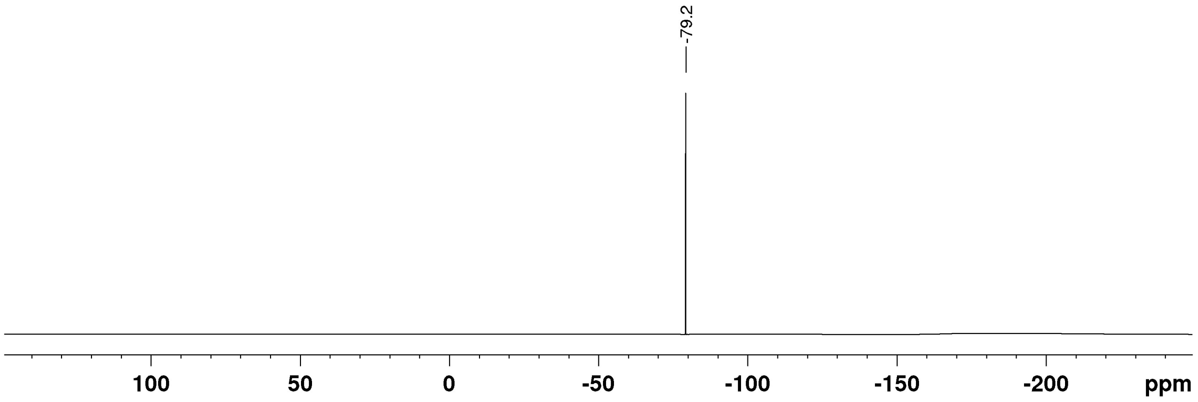

Figure S52. ^{19}F NMR spectrum of **5d**[OTf] (300 K, CD_3CN).

S2.20 Preparation of [(L$_C$)P=C(C_9H_{16})][OTf] (5e[OTf])

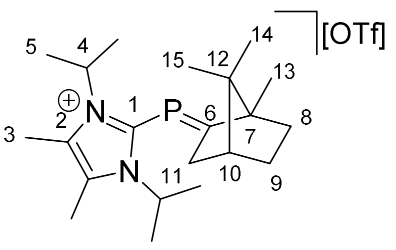

To a solution of **2**[OTf]$_4$ (200 mg, 0.14 mmol, 1.0 eq.) and Ph_3P (153 mg, 0.58 mmol, 4.2 eq.) in CH_3CN (4 ml) a solution of (1*R*)-(−)-Thiocampher (98 mg, 0.58 mmol, 4.2 eq.) in CH_3CN (1 ml) was added and the mixture was heated to 80°C for 3 h using a microwave reactor. The volume of the resulting yellow suspension was reduced to 2 ml *in vacuo* before it was filtered. Addition of 15 mL of Et_2O gives a slightly cloudy mixture, from which colorless crystals grow over the course of 16 h at -30°C. Their isolation and drying *in vacuo* affords the product as an air and moisture sensitive colorless solid. Crystals suitable for X-ray diffraction analysis were also obtained by diffusion of *n*-pentane into a saturated C_6H_5F solution at −30°C.

Yield: 213 mg (77%); **m.p.**: 124-126 °C; **Raman** (100 mW, in cm^{-1}): 2974 (63), 2948 (100), 2938 (90), 2874 (27), 2773 (7), 2727 (10), 1707 (6), 1631 (25), 1586 (6), 1526 (7), 1452 (55), 1419 (20), 1402 (33), 1385 (19), 1354 (17), 1343 (16), 1312 (23), 1294 (61), 1276 (25), 1263 (27), 1238 (14), 1222 (18), 1211 (27), 1192 (16), 1175 (17), 1160 (15), 1134 (12), 1113 (13), 1095 (18), 1078 (11), 1030 (54), 1013 (10), 997 (19), 985 (14), 949 (18), 909 (16), 891 (24), 856 (18), 835 (8), 790 (14), 753 (25), 712 (14), 691 (9), 671 (15), 642 (39), 595 (14); **IR** (ATR, in cm^{-1}): 2972 (vw), 2960 (vw), 2947 (vw), 2872 (vw), 1629 (vw), 1463 (vw), 1449 (vw), 1417 (vw), 1401 (vw), 1379 (vw), 1353 (vw), 1322 (vw), 1304 (vw), 1291 (vw), 1262 (vs), 1221 (w), 1172 (vw), 1148 (m), 1112 (w), 1091 (w), 1029 (s), 996 (vw), 947 (vw), 936 (vw), 904 (vw), 888 (vw), 851 (vw), 788 (vw), 762 (vw), 752 (vw), 710 (vw), 671 (vw), 636 (vs), 595 (vw), 571 (w), 546 (vw), 534 (vw), 516 (m), 493 (w), 436 (vw), 423 (vw); **^{1}H NMR** (CD_3CN, 300 K, in ppm): δ = 0.81 (3H, s, H15), 1.00 (3H, s, H14), 1.18 (1H, m, H9$_{ax}$), 1.31 (3H, s, H13), 1.41 (1H, m, H8$_{ax}$), 1.53 (12H, s(br), H5), 1.90 (1H, m, H9$_{eq}$), 1.97 (1H, m, H10), 2.02 (1H, m, H8$_{eq}$), 2.17 (1H, dd, $^1J_{HH}$ = 20.6 Hz, H11$_{ax}$), 2.34 (6H, s, H3), 2.63 (1H, m, H11$_{eq}$), 4.78 (2H, s(br), H4); **^{13}C{^{1}H} NMR** (CD_3CN, 300 K, in ppm): δ = 10.7 (2C, s(br), C3), 13.8 (1C, d, $^3J_{CP}$ = 20 Hz, C13), 19.1 (1C, d, $^4J_{CP}$ = 2 Hz, C15), 19.9 (1C, s, C14), 21.7 (4C, s(br), C5), 27.7 (1C, s, C9), 37.1 (1C, d, $^3J_{CP}$ = 10 Hz, C8), 46.2 (1C, s, C10), 46.5 (1C, d, $^2J_{CP}$ = 16 Hz, C11), 50.3 (1C, d, $^3J_{CP}$ = 5 Hz, C12), 54.4 (2C, s(br), C4), 62.3 (1C, d, $^1J_{CP}$ = 21 Hz, C7), 122.2 (1C, q, $^1J_{CF}$ = 321 Hz, OTf), 130.9 (2C, s(br), C2), 145.8 (1C, d, $^1J_{CP}$ = 87 Hz, C1), 233.5 (1C, d, $^1J_{CP}$ = 51 Hz, C6); **^{19}F{^{1}H} NMR** (CD_3CN, 300 K, in ppm): δ = -79.3 (3F, s, OTf); **^{31}P{^{1}H} NMR** (CD_3CN, 300 K, in ppm): δ = 133.6 (1P, s, P); **elemental analysis**: calcd. for $C_{22}H_{36}F_3N_2O_3PS$: C: 53.21, H: 7.31, N: 5.64, S: 6.46; found: C: 52.89, H: 7.086, N: 5.46, S: 6.495.

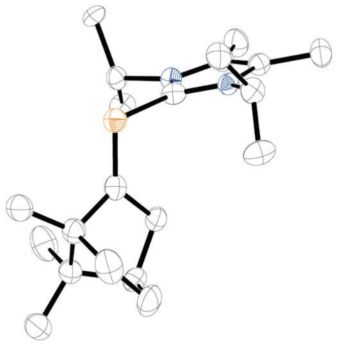

Figure S53. Molecular structure of **5c**$^+$ in **5c**[OTf]; hydrogen atoms and the anion are omitted for clarity and thermal ellipsoids are displayed at 50% probability.

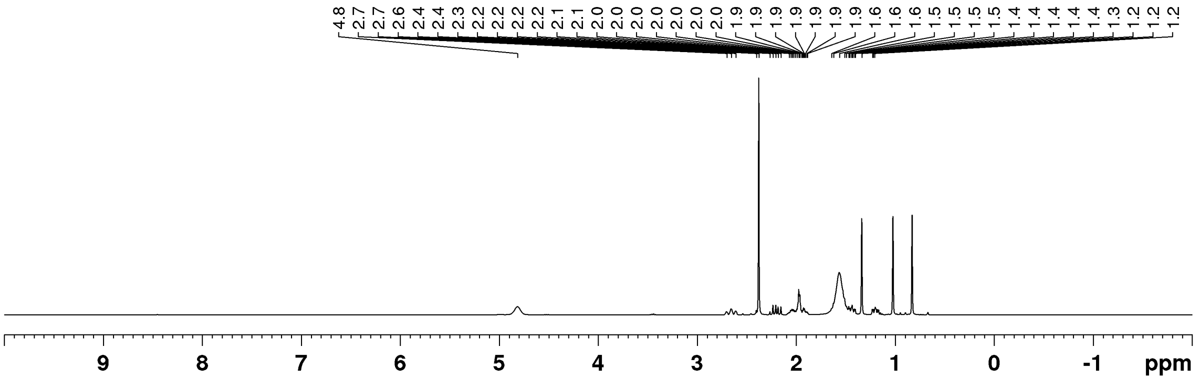

Figure S54. ^{1}H NMR spectrum of **5e**[OTf] (300 K, CD_3CN).

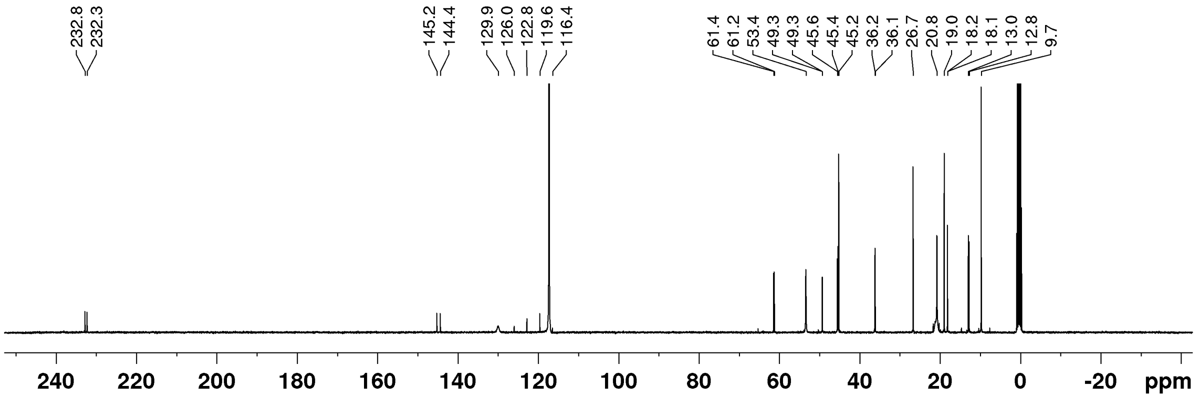

Figure S55. $^{13}C\{^{1}H\}$ NMR spectrum of **5e**[OTf] (300 K, CD_3CN).

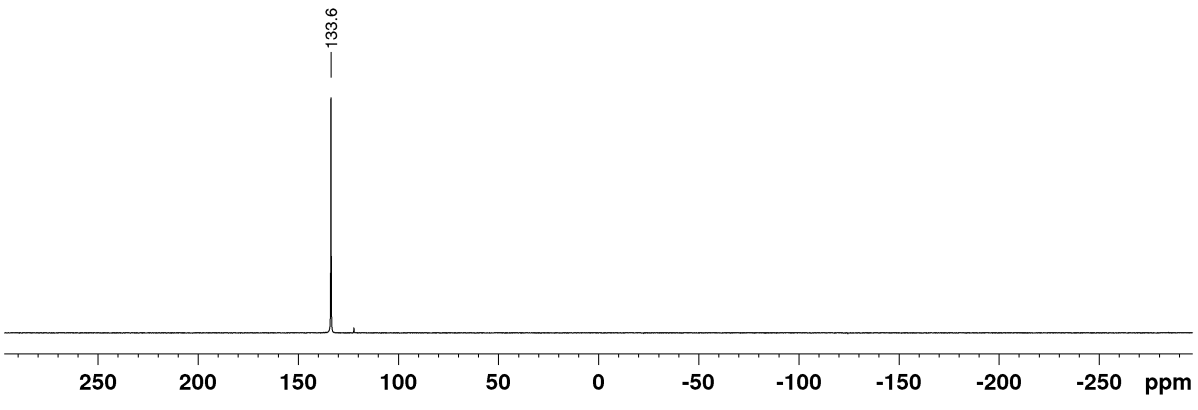

Figure S56. ^{31}P NMR spectrum of **5e**[OTf] (300 K, CD_3CN).

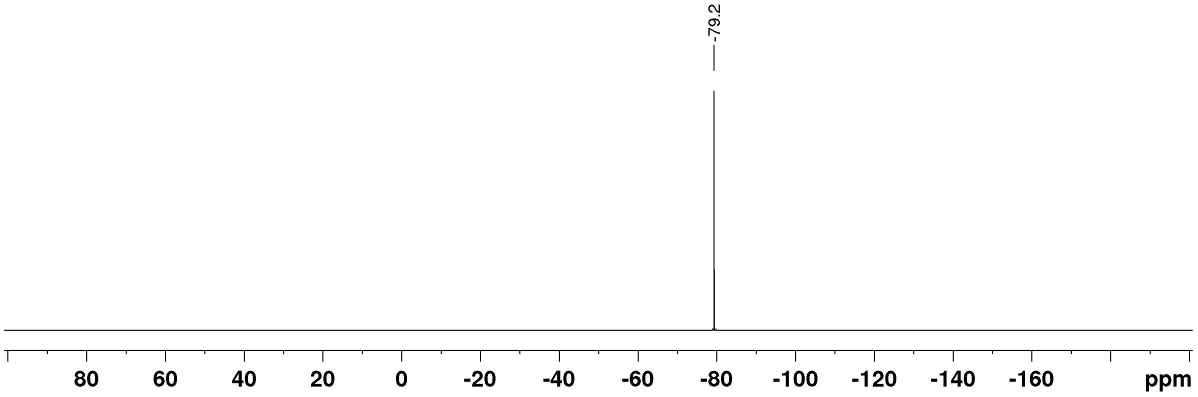

Figure S57. ^{19}F NMR spectrum of **5e**[OTf] (300 K, CD_3CN).

S2.21 Preparation of [(L_C)P–C(H)N(Me)$_2$][OTf] (6a[OTf])

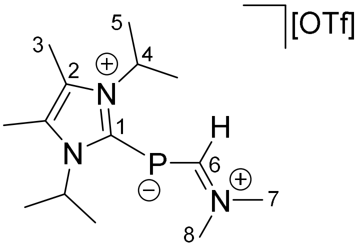

To a solution of **2**[OTf]$_4$ (500 mg, 0.34 mmol, 1.0 eq.) and Ph_3P (382 mg, 1.46 mmol, 4.2 eq.) in CH_3CN (4 ml) a solution of N,N-dimethylthioformamid (130 mg, 1.46 mmol, 4.2 eq.) in CH_3CN (1 ml) was added and the mixture was stirred for 16 h at room temperature. The resulting colorless suspension was filtered, and the filtrate evaporated to dryness *in vacuo*. The crude solid was subsequently washed three times with Et_2O (3x4 mL) and dried *in vacuo* to afford the product as an air and moisture sensitive colorless solid. Crystals suitable for X-ray diffraction analysis were obtained by diffusion of *n*-pentane into a saturated C_6H_5F solution at –30°C.

Yield: 502 mg (87%); **m.p.**: 127-129°C; **Raman** (100 mW, in cm^{-1}): 2984 (51), 2945 (100), 2886 (35), 2806 (13), 2739 (8), 1627 (32), 1545 (18), 1473 (27), 1448 (62), 1425 (56), 1419 (58), 1400 (29), 1390 (32), 1367 (50), 1340 (11), 1329 (17), 1304 (59), 1274 (10), 1224 (14), 1155 (13), 1139 (18), 1090 (9), 1032 (65), 908 (29), 892 (22), 829 (34), 788 (20), 752 (22), 585 (14), 572 (13), 549 (13), 506 (17), 461 (13), 417 (13), 394 (13), 346 (18), 310 (19), 277 (15), 235 (18), 208 (15), 165 (13), 81 (88); **IR** (ATR, in cm^{-1}): 2985 (vw), 2938 (vw), 2885 (vw), 1626 (vw), 1544 (m), 1468 (vw), 1443 (vw), 1409 (m), 1376 (w), 1329 (vw), 1302 (vw), 1260 (vs), 1221 (m), 1138 (vs), 1055 (vw), 1027 (vs), 978 (vw), 941 (vw), 825 (w), 788 (vw), 754 (w), 710 (vw), 630 (vs), 571 (w), 551 (vw), 513 (m), 458 (vw), 417 (vw); **^{1}H NMR** (CD_3CN, 300 K, in ppm): δ = 1.50 (12H, d, $^3J_{HH}$ = 7.1 Hz, H5), 2.34 (6H, s, H3), 3.03 (3H, d, $^4J_{HP}$ = 4.2 Hz, H7), 3.16 (3H, d, $^4J_{HP}$ = 3.5 Hz, H8), 5.36 (2H, s (br), H4); **^{13}C{^{1}H} NMR** (CD_3CN, 300 K, in ppm): δ = 10.7 (2C, s, C3), 21.1 (4C, s, C5), 40.3 (1C, d, $^3J_{CP}$ = 24 Hz, C7), 47.8 (1C, d, $^3J_{CP}$ = 2 Hz, C8), 53.9 (2C, d, $^3J_{CP}$ = 4 Hz, C4), 129.6 (2C, s, C2), 150.3 (1C, d, $^1J_{CP}$ = 77 Hz, C1), 191.7 (1C, d, $^1J_{CP}$ = 54 Hz, C6); **^{19}F{^{1}H} NMR** (CD_3CN, 300 K, in ppm): δ = -79.3 (3F, s, OTf); **^{31}P{^{1}H} NMR** (CD_3CN, 300 K, in ppm): δ = -8.8 (1P, s (br), P); **elemental analysis**: calcd. for $C_{15}H_{27}F_3N_3O_3PS$: C: 43.16, H: 6.52, N: 10.07, S: 7.68; found: C: 43.03, H: 6.222, N: 10.03, S: 7.662.

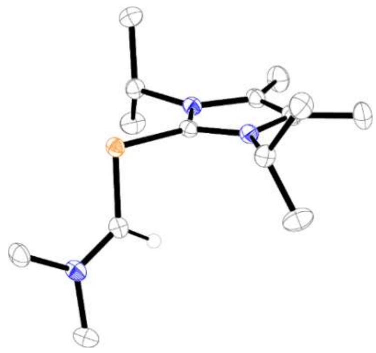

Figure S58. Molecular structure of **6a**$^+$ in **6a**[OTf]; hydrogen atoms and the anion are omitted for clarity and thermal ellipsoids are displayed at 50% probability.

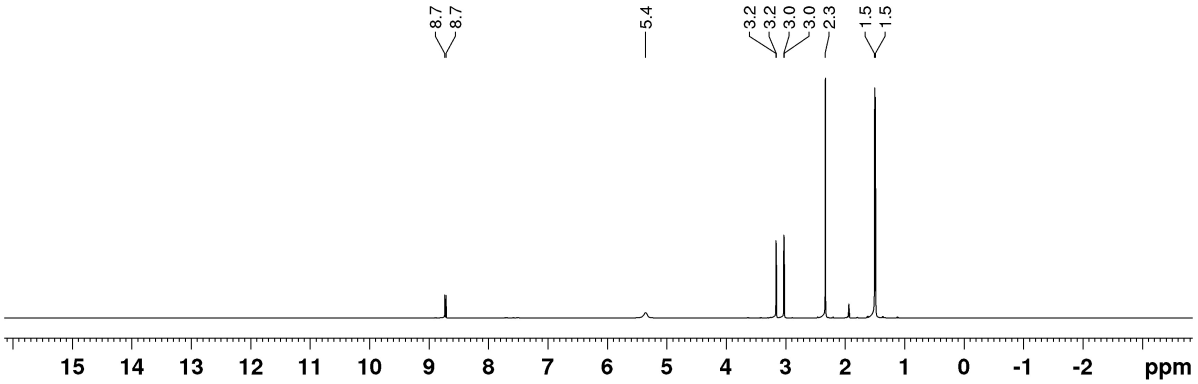

Figure S59. ^{1}H NMR spectrum of **6a**[OTf] (300 K, CD_3CN).

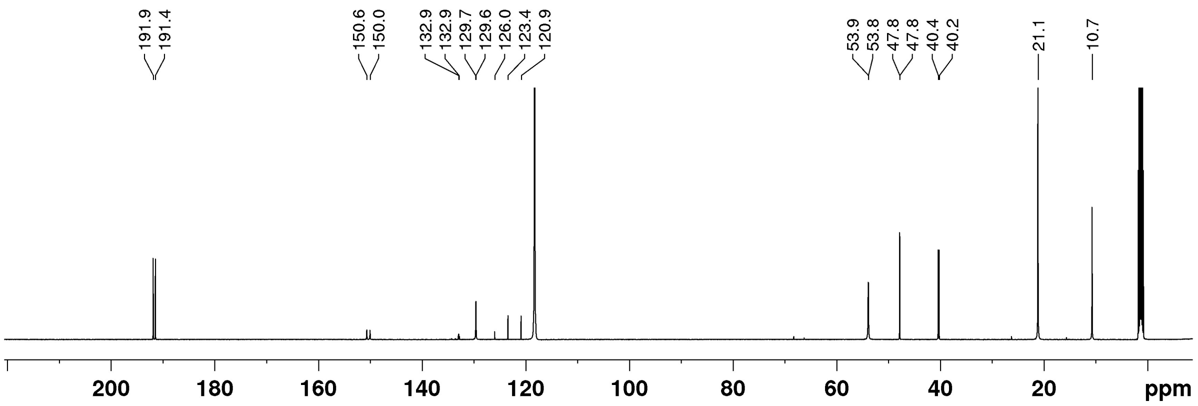

Figure S60. $^{13}C\{^{1}H\}$ NMR spectrum of **6a**[OTf] (300 K, CD_3CN).

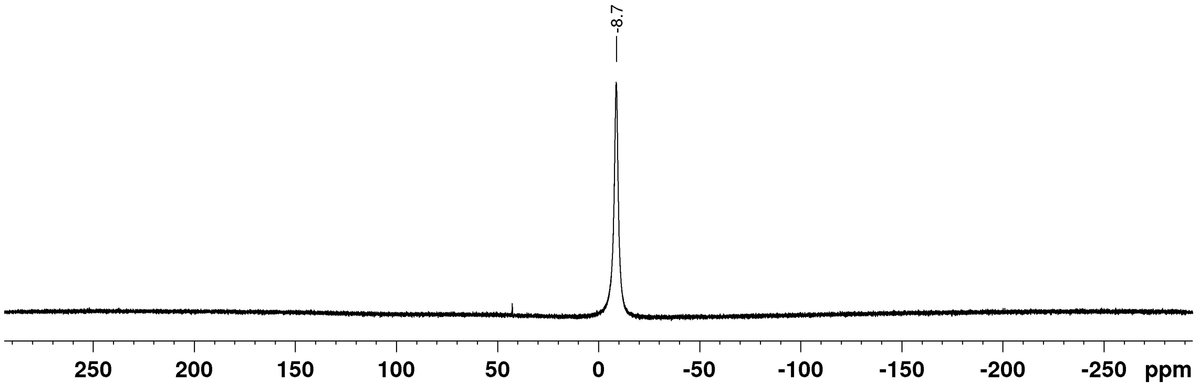

Figure S61. ^{31}P NMR spectrum of **6a**[OTf] (300 K, CD_3CN).

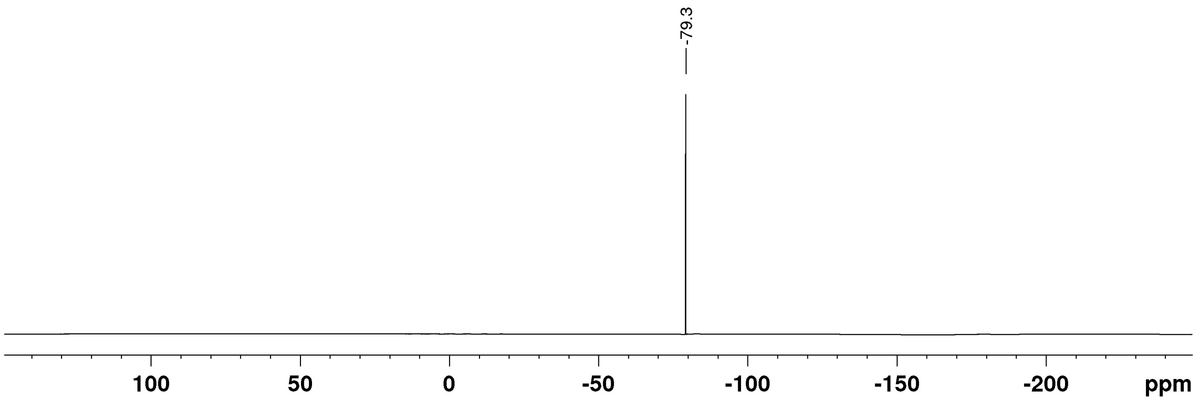

Figure S62. ^{19}F NMR spectrum of **6a**[OTf] (300 K, CD_3CN).

S2.22 Preparation of [(L$_C$)P–C(Ph)N(Me)$_2$][OTf] (6b[OTf])

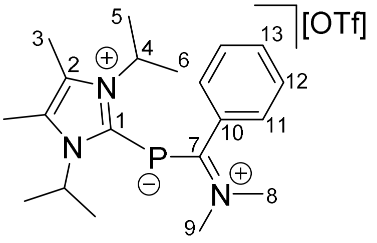

To a solution of **2**[OTf]$_4$ (150 mg, 0.10 mmol, 1.0 eq.) and Ph_3P (115 mg, 0.44 mmol, 4.2 eq.) in CH_3CN (4 ml) a solution of *N,N*-dimethylbenzothioamide (72 mg, 0.44 mmol, 4.2 eq.) in CH_3CN (1 ml) was added and the mixture was stirred for 16 h at room temperature. The resulting yellow suspension was reduced to 2 mL *in vacuo* and 10 mL of Et_2O were added giving a fine colorless precipitate. After filtration, washing with Et_2O (2x2 mL) and drying *in vacuo*, the product was obtained as an air and moisture sensitive colorless solid. Crystals suitable for X-ray diffraction analysis were obtained by diffusion of Et_2O into a saturated CH_3CN solution at –30°C.

Yield: 171 mg (83%); **m.p.**: 193-195°C; **Raman** (100 mW, in cm^{-1}): 3058 (27), 2988 (30), 2971 (38), 2937 (60), 2876 (18), 1613 (29), 1599 (39), 1575 (10), 1516 (12), 1452 (43), 1436 (34), 1407 (55), 1356 (59), 1331 (12), 1290 (61), 1225 (20), 1185 (13), 1146 (18), 1139 (20), 1085 (11), 1033 (52), 1003 (43), 987 (18), 888 (17), 787 (18), 752 (17), 674 (25), 635 (11), 602 (12), 578 (15), 544 (21), 514 (20), 473 (16), 448 (19), 388 (10), 347 (30), 312 (20), 299 (15), 270 (20), 207 (23), 185 (26), 159 (20), 125 (51), 98 (100); **IR** (ATR, in cm^{-1}): 3055 (vw), 2970 (vw), 2934 (vw), 2879 (vw), 1612 (vw), 1514 (w), 1490 (vw), 1463 (vw), 1440 (vw), 1397 (m), 1372 (w), 1264 (vs), 1223 (w), 1138 (vs), 1107 (w), 1086 (vw), 1061 (vw), 1032 (s), 1003 (vw), 981 (vw), 928 (vw), 905 (vw), 889 (vw), 758 (m), 708 (m), 674 (vw), 635 (vs), 571 (w), 543 (vw), 516 (w); **^{1}H NMR** (CD_3CN, 300 K, in ppm): δ = 1.34 (6H, d, $^3J_{HH}$ = 7.0 Hz, H5), 1.41 (6H, d, $^3J_{HH}$ = 7.1 Hz, H6), 2.14 (6H, s, H3), 2.90 (3H, d, $^4J_{HP}$ = 3.4 Hz, H8), 3.37 (3H, d, $^4J_{HP}$ = 5.7 Hz, H9), 5.39 (2H, m, H4), 7.05-7.10 (4H, m, H11), 7.27-7.39 (3H, m, H12&H13); **^{13}C{^{1}H} NMR** (CD_3CN, 300 K, in ppm): δ = 10.7 (2C, s, C3), 21.1 (2C, s, C5), 21.3 (2C, s, C6), 43.5 (1C, d, $^3J_{CP}$ = 35 Hz, C9), 44.5 (1C, d, $^3J_{CP}$ = 2 Hz, C8), 54.2 (2C, d, $^3J_{CP}$ = 6 Hz, C4), 127.5 (2C, d, $^3J_{CP}$ = 3 Hz, C11), 129.5 (2C, d, $^4J_{CP}$ = 2 Hz, C12), 129.6 (2C, s, C2), 130.1 (1C, s, C13), 139.1 (1C, d, $^2J_{CP}$ = 7 Hz, C10), 149.1 (1C, d, $^1J_{CP}$ = 79 Hz, C1), 202.6 (1C, d, $^1J_{CP}$ = 64 Hz, C7); **^{19}F{^{1}H} NMR** (CD_3CN, 300 K, in ppm): δ = -79.3 (3F, s, OTf); **^{31}P{^{1}H} NMR** (CD_3CN, 300 K, in ppm): δ = 7.9 (1P, s, P); **elemental analysis**: calcd. for $C_{21}H_{31}F_3N_3O_3PS$: C: 51.11, H: 6.33, N: 8.51, S: 6.50; found: C: 51.49, H: 6.679, N: 8.33, S: 6.543.

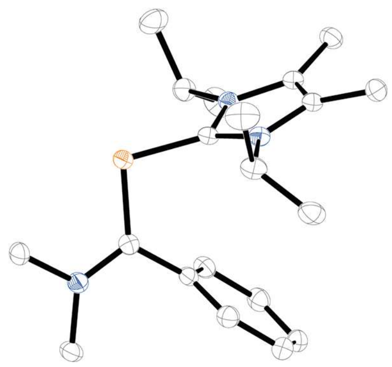

Figure S63. Molecular structure of **6b**$^+$ in **6b**[OTf]; hydrogen atoms and the anion are omitted for clarity and thermal ellipsoids are displayed at 50% probability.

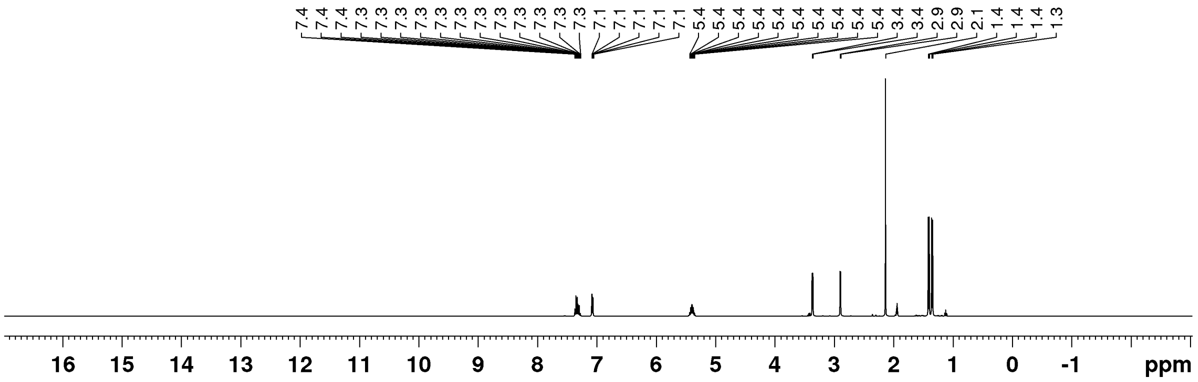

Figure S64. ^{1}H NMR spectrum of **6b**[OTf] (300 K, CD_3CN).

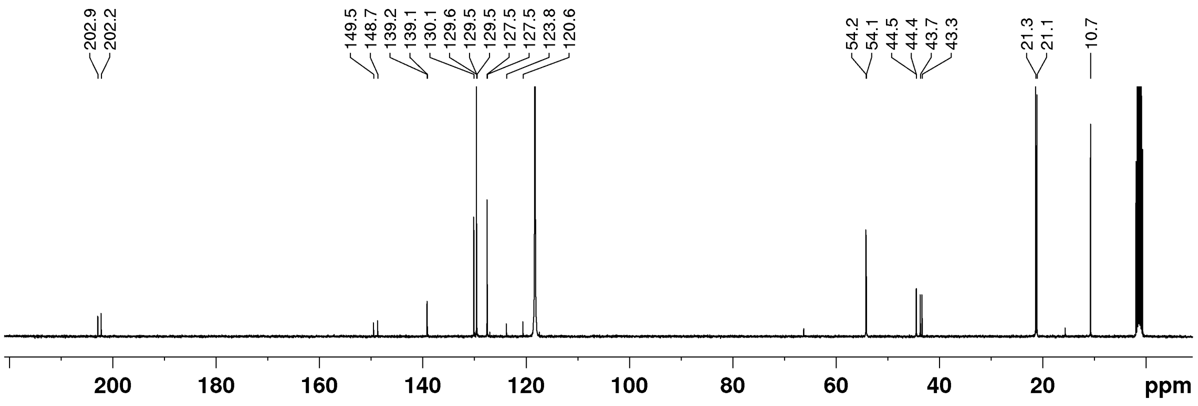

Figure S65. $^{13}C\{^{1}H\}$ NMR spectrum of **6b**[OTf] (300 K, CD_3CN).

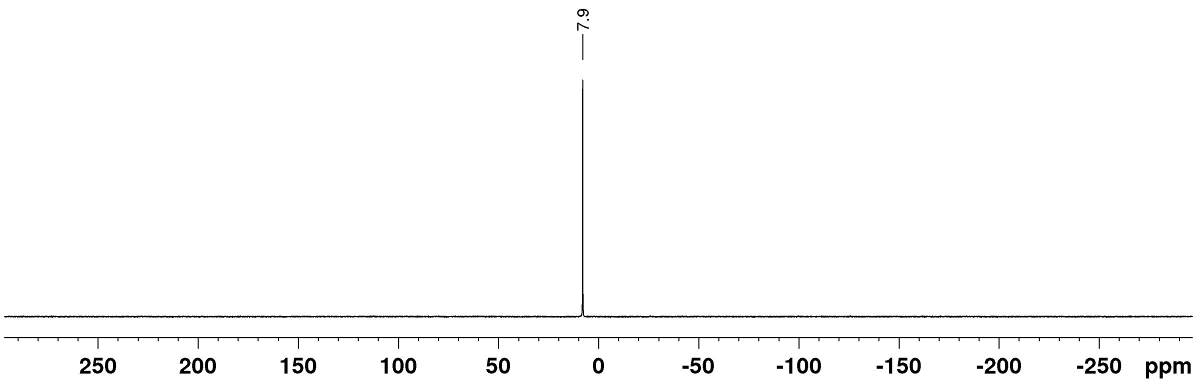

Figure S66. ^{31}P NMR spectrum of **6b**[OTf] (300 K, CD_3CN).

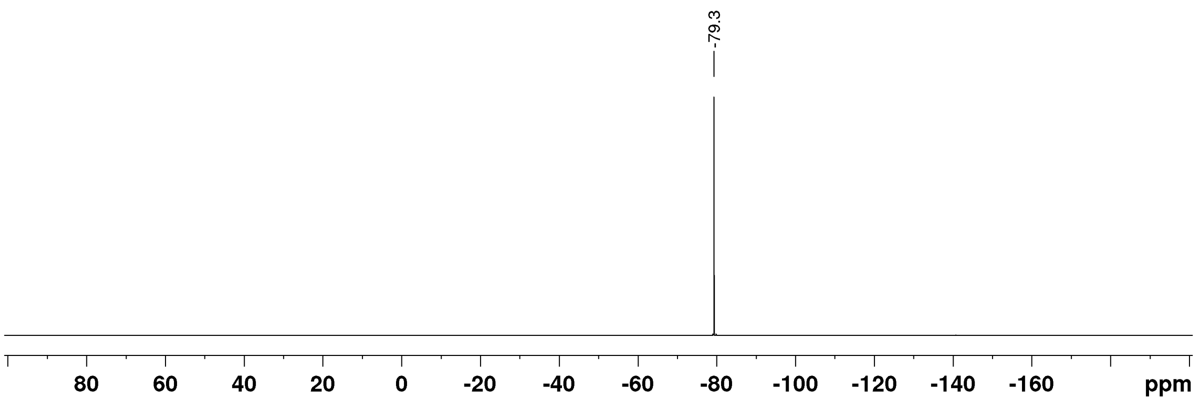

Figure S67. ^{19}F NMR spectrum of **6b**[OTf] (300 K, CD_3CN).

S2.23 Preparation of [(L$_C$)P–C(N(Me)$_2$)$_2$][OTf] (6c[OTf])

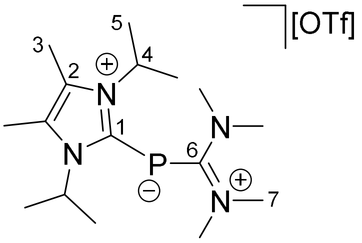

A solution of **2**[OTf]$_4$ (100 mg, 0.07 mmol, 1.0 eq.), Ph_3P (77 mg, 0.29 mmol, 4.2 eq.) and *N,N,N,N*-tetramethylthiourea (39 mg, 0.29 mmol, 4.2 eq.) in CH_3CN (4 ml) was heated to 80°C for 3 h using a microwave reactor. The resulting yellow suspension was filtered and the filtrate evaporated to dryness *in vacuo*. The crude solid was subsequently washed three times with Et_2O (3x2 mL) and recrystallized from a saturated CH_3CN/Et_2O solution at -30°C to afford the product as an air and moisture sensitive, colorless and crystalline solid. Crystals suitable for X-ray diffraction analysis were obtained by the same method.

Yield: 95 mg (76%); **m.p.**: 144-146°C; **Raman** (100 mW, in cm^{-1}): 2979 (43), 2946 (72), 2927 (65), 2863 (25), 2832 (10), 2812 (20), 1627 (31), 1502 (12), 1446 (59), 1419 (58), 1405 (100), 1385 (51), 1375 (63), 1340 (87), 1316 (24), 1294 (69), 1268 (17), 1225 (13), 1149 (28), 1097 (18), 1079 (21), 1065 (13), 1031 (61), 940 (11), 887 (17), 878 (16), 789 (31), 754 (25), 710 (14), 633 (20), 606 (29), 580 (14), 572 (17), 548 (17), 518 (10), 503 (14), 462 (31), 389 (21), 347 (27), 312 (28), 275 (21), 211 (17), 174 (23), 104 (46), 72 (75); **IR** (ATR, in cm^{-1}): 2971 (vw), 2940 (vw), 2907 (vw), 2834 (vw), 2811 (vw), 1626 (vw), 1529 (w), 1502 (vw), 1475 (w), 1463 (vw), 1370 (m), 1342 (vw), 1258 (vs), 1223 (m), 1140 (s), 1100 (m), 1077 (m), 1024 (vs), 980 (vw), 939 (vw), 905 (w), 875 (w), 789 (vw), 753 (w), 630 (vs), 570 (w), 548 (w), 512 (m), 460 (w); **^{1}H NMR** (CD_3CN, 300 K, in ppm): δ = 1.46 (12H, s (br), H5), 2.32 (6H, s, H3), 2.83 (6H, s (br), NMe_2), 3.00 (6H, s (br), NMe_2), 5.22 (2H, m, H4); **^{13}C{^{1}H} NMR** (CD_3CN, 300 K, in ppm): δ = 10.9 (2C, s, C3), 21.2 (4C, s (br), C5), 43.4 (4C, s (br), C7), 53.7 (2C, d, $^3J_{CP}$ = 8 Hz, C4), 129.5 (2C, d, $^3J_{CP}$ = 2 Hz, C2), 153.0 (1C, d, $^1J_{CP}$ = 89 Hz, C1), 197.5 (1C, d, $^1J_{CP}$ = 77 Hz, C6); **^{19}F{^{1}H} NMR** (CD_3CN, 300 K, in ppm): δ = -79.2 (3F, s, OTf); **^{31}P{^{1}H} NMR** (CD_3CN, 300 K, in ppm): δ = -60.2 (1P, s (br), P); **elemental analysis**: calcd. for $C_{17}H_{32}F_3N_4O_3PS$: C: 44.34, H: 7.00, N: 12.17, S: 6.96; found: C: 43.96, H: 6.687, N: 12.19, S: 6.727.

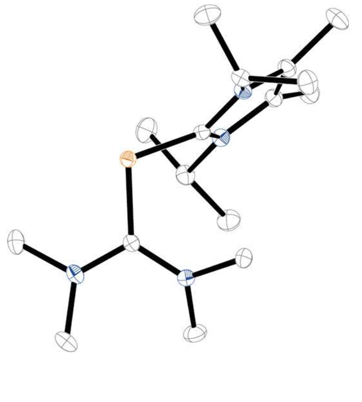

Figure S68. Molecular structure of **6c**$^+$ in **6c**[OTf]; hydrogen atoms and the anion are omitted for clarity and thermal ellipsoids are displayed at 50% probability.

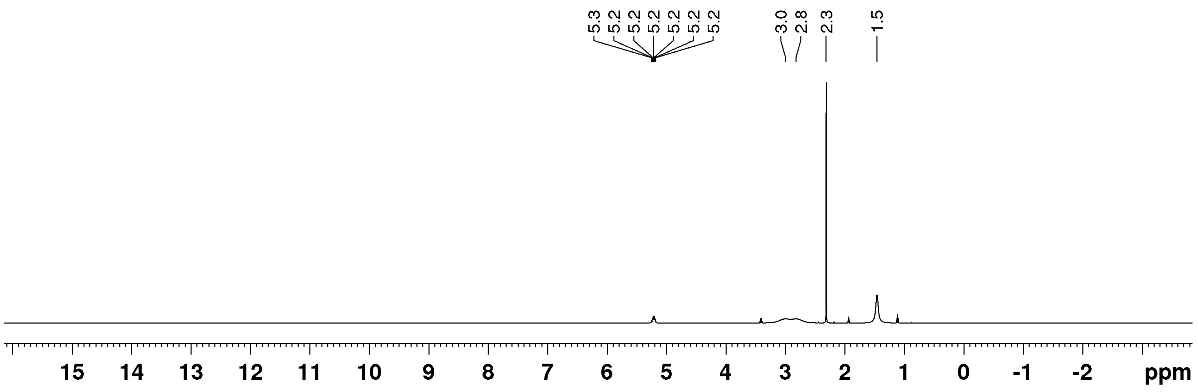

Figure S69. ^{1}H NMR spectrum of **6c**[OTf] (300 K, CD_3CN).

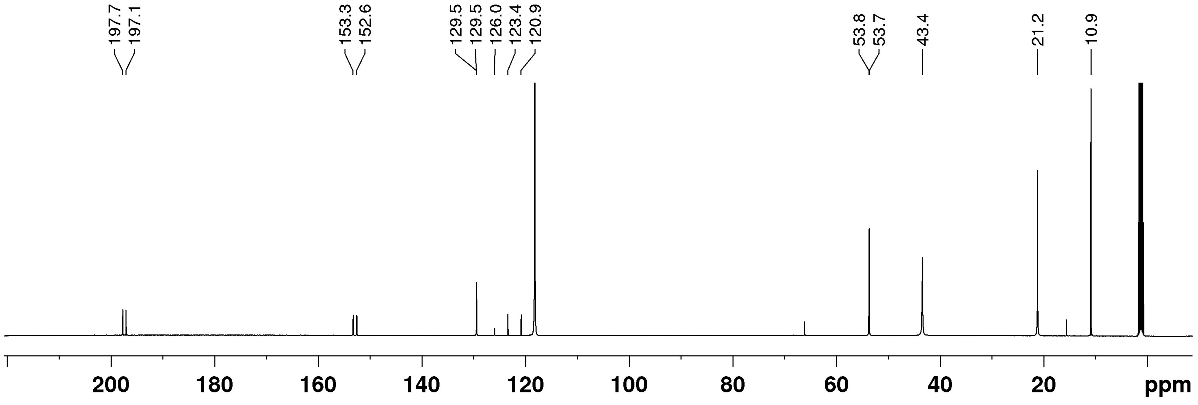

Figure S70. $^{13}C\{^{1}H\}$ NMR spectrum of **6c**[OTf] (300 K, CD_3CN).

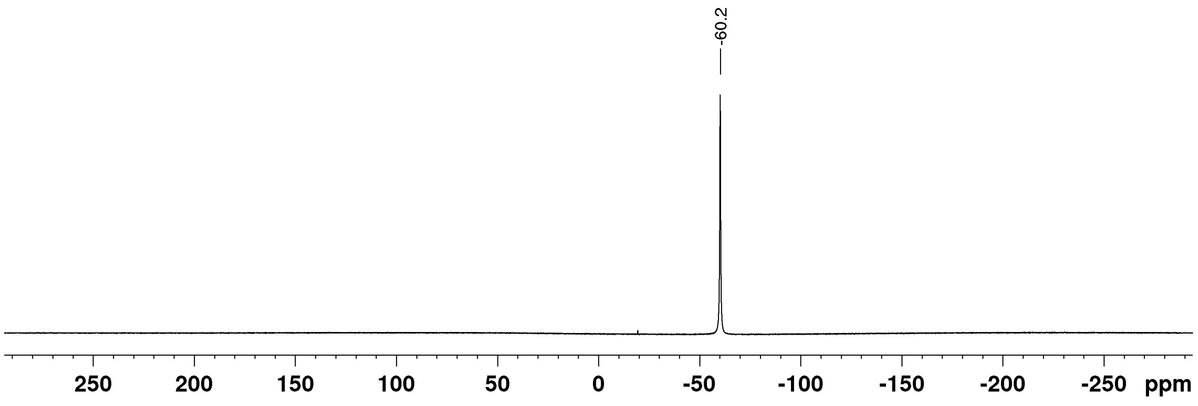

Figure S71. ^{31}P NMR spectrum of **6c**[OTf] (300 K, CD_3CN).

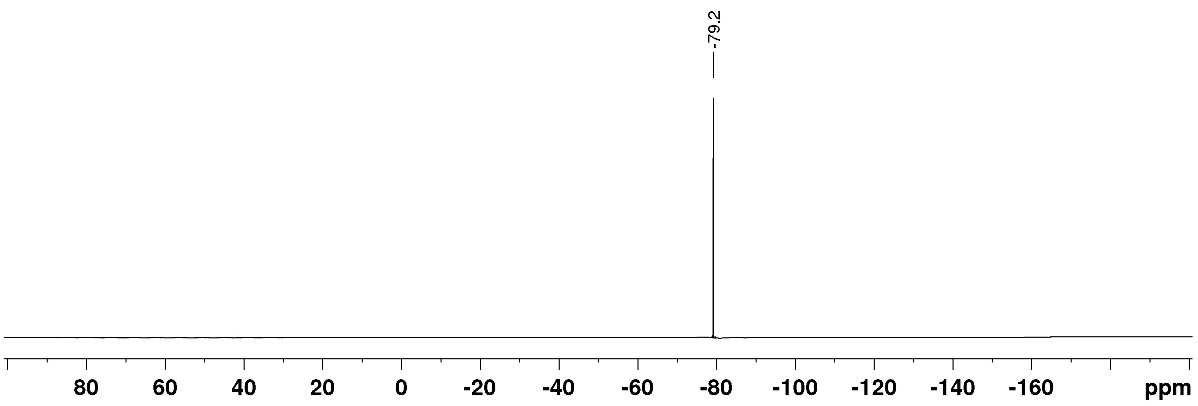

Figure S72. ^{19}F NMR spectrum of **6c**[OTf] (300 K, CD_3CN).

S2.24 Reaction of 2[OTf]$_4$ with 10 eq. of Ph_3P and 1,3-diisopropyl-4,5-dimethyl-1,3-dihydro-2H-imidazole-2-thione

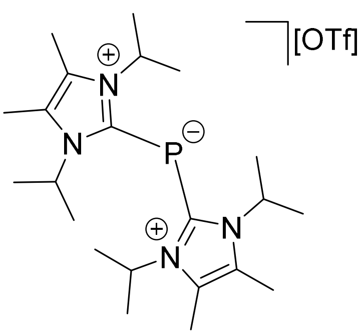

A solution of **2**[OTf]$_4$ (100 mg, 0.07 mmol, 1.0 eq.), Ph_3P (182 mg, 0.7 mmol, 10 eq.) and 1,3-diisopropyl-4,5-dimethyl-1,3-dihydro-2H-imidazole-2-thione (62 mg, 0.29 mmol, 4.2 eq.) in CD_3CN (4 ml) was heated to 80°C for 24 h using a microwave reactor. The resulting yellow suspension was filtered and analyzed by means of ^{31}P NMR spectroscopy (**Figure S73**).

$^{31}P\{^1H\}$ NMR (CD_3CN, 300 K, in ppm): δ = -125.9 (1P, s (br), P).

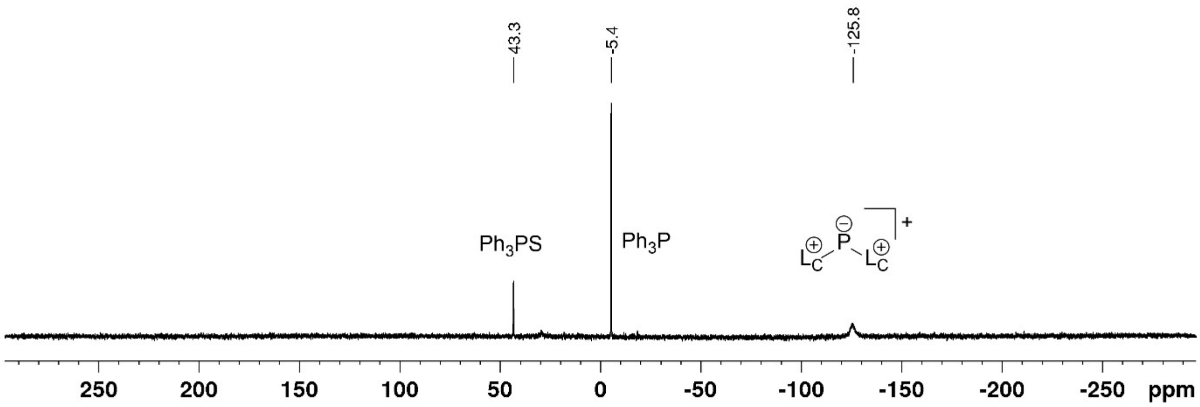

Figure S73. ^{31}P NMR spectrum of the reaction mixture after 24 h at 80°C in a microwave reactor (300 K, C_6D_6 cap.).

S2.25 Reaction of 2[OTf]$_4$ with 4 eq. of Ph_3P and excess of *O*-methyl benzothioate

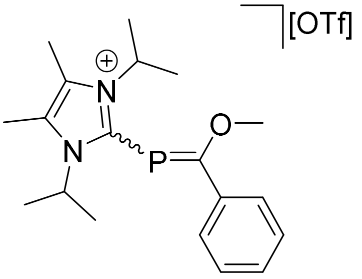

A solution of **2**[OTf]$_4$ (150 mg, 0.10 mmol, 1.0 eq.), Ph_3P (115 mg, 0.44 mmol, 4.2 eq.) and a large excess of *O*-methyl benzothioate (238 mg, 1.6 mmol, 15 eq.) in *o*-$C_6H_4F_2$ (4 ml) was heated to 100°C for 3 h using a microwave reactor. The resulting yellow solution was evaporated to dryness *in vacuo* and the crude residue was subsequently washed with *n*-hexane (1x2 mL), Et_2O (2x2 mL) and *n*-pentane (2x2 mL) and again dried *in vacuo* giving a yellow to orange sticky solid, which was investigated by means of multinuclear NMR spectroscopy (**Figure S74**). Attempts to recrystallize the target compound

from a saturated CH_3CN/Et_2O solution at -30°C affords mainly orange oils. Analysis of some crystalline material obtained via this method revealed one product to be *Z*-**5f**[OTf].

$^{31}P\{^1H\}$ NMR (CD_3CN, 300 K, in ppm): δ = 56.0 (1P, s, P, *Z*-isomer), δ = 42.6 (1P, s, P, *E*-isomer).

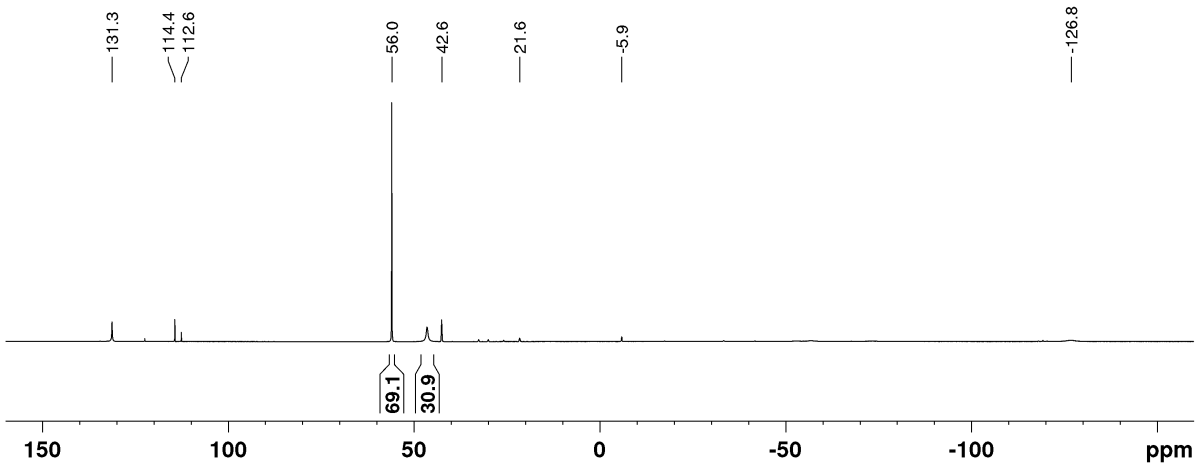

Figure S74. ^{31}P NMR spectrum of reaction mixture after irradiation in a microwave reactor at 100°C over 3 h in o-$C_6H_4F_2$ with 15-fold excess thioester. Resonances at a chemical shift of δ = 56.0 and δ = 42.6 are assigned to E-**5f**$^+$ and Z-**5f**$^+$, respectively.

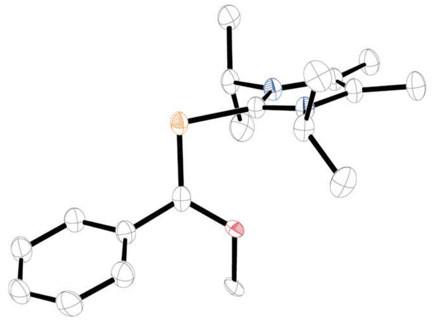

Figure S75. Molecular structure of *Z*-**5f**$^+$ in **5f**[OTf]; hydrogen atoms and the anion are omitted for clarity and thermal ellipsoids are displayed at 50% probability.

S2.26 Preparation of [(L$_C$)P(Fe(CO)$_4$)C(Ph)$_2$][OTf] (13[OTf])

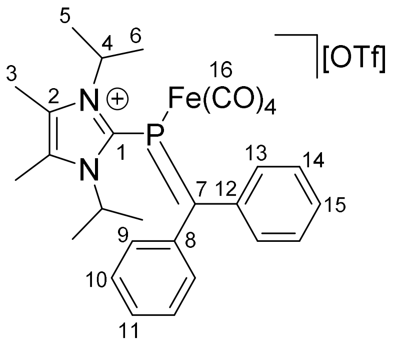

To a yellow solution of **5a**[OTf] (100 mg, 0.2 mmol, 1.0 eq.) in THF (2 mL) a suspension of $[Fe_2(CO)_9]$ (69.0 mg, 0.1 mmol, 1.0 eq.) in THF (2 mL) was added dropwise. After complete addition a dark brown solution is obtained and stirred at room temperature for 16 h. The occurring red precipitate is filtered off, washed twice with THF (2x2 mL) and dried *in vacuo* to afford the product as a red air and moisture sensitive powder. Vapor diffusion of Et_2O into a saturated solution of **13**[OTf] in CH_3CN gives the product as dark red blocks suitable for single crystal X-ray analysis.

Yield: 114 mg (86%); **m.p.**: 166-168 (decomp.); **Raman** (100 mW, in cm^{-1}): 3073 (9), 2997 (6), 2977 (5), 2948 (11), 2074 (51), 2012 (12), 1994 (22), 1967 (31), 1592 (87), 1488 (42), 1445 (17), 1408 (15), 1387 (10), 1366 (17), 1283 (54), 1267 (69), 1234 (100), 1183 (24), 1164 (11), 1150 (10), 1090 (8), 1047 (23), 1032 (13), 1023 (9), 1000 (32), 972 (8), 916 (10), 887 (8), 846 (14), 793 (10), 770 (10), 757 (15), 692 (11), 627 (11), 620 (10), 607 (8), 583 (13), 543 (8), 509 (7), 486 (17), 472 (13), 431 (51), 404 (15), 380 (9), 347 (10), 310 (9); **IR** (ATR, in cm^{-1}): 3073 (vw), 3003

(vw), 2946 (vw), 2073 (m), 2013 (m), 1993 (vs), 1965 (vs), 1927 (vw), 1612 (vw), 1488 (vw), 1463 (vw), 1443 (vw), 1412 (vw), 1393 (vw), 1376 (vw), 1265 (vs), 1221 (w), 1183 (vw), 1145 (s), 1111 (vw), 1090 (vw), 1031 (s), 999 (vw), 904 (vw), 894 (vw), 769 (w), 756 (w), 697 (w), 624 (vs), 597 (s), 572 (w), 554 (vw), 542 (w), 535 (w), 517 (w), 508 (w), 485 (w), 468 (vw), 455 (vw), 429 (w), 412 (vw); **^{1}H NMR** (CD_3CN, 300 K, in ppm): δ = 1.37 (6H, d, $^3J_{HH}$ = 6.9 Hz, H5), 1.61 (6H, d, $^3J_{HH}$ = 6.9 Hz, H6), 2.35 (6H, s, H3), 5.34 (2H, dsept, $^3J_{HH}$ = 6.8 Hz, $^4J_{HP}$ = 3.3 Hz, H4), 6.96-7.01 (2H, m, H9), 7.29-7.40 (3H, m, H10&H11), 7.52-7.60 (5H, m, H13-H15); **^{13}C{^{1}H} NMR** (CD_3CN, 300 K, in ppm): δ = 11.0 (2C, s, C3), 21.0 (2C, s, C5), 21.7 (2C, s, C6), 55.4 (2C, d, $^3J_{CP}$ = 3 Hz, C4), 129.7 (2C, d, $^3J_{CP}$ = 11 Hz, C9), 129.8 (2C, s, C10), 130.2 (2C, s, C14), 130.8 (1C, d, $^5J_{CP}$ = 3 Hz, C11), 131.2 (1C, d, $^5J_{CP}$ = 4 Hz, C15), 131.6 (2C, d, $^3J_{CP}$ = 16 Hz, C13), 132.1 (2C, s, C2), 141.9 (1C, d, $^2J_{CP}$ = 17 Hz, C12), 142.7 (1C, d, $^1J_{CP}$ = 2 Hz, C1), 143.6 (1C, d, $^2J_{CP}$ = 11 Hz, C8), 185.3 (1C, d, $^1J_{CP}$ = 50 Hz, C7), 213.0 (4C, d, $^2J_{CP}$ = 10 Hz, C16); **^{19}F{^{1}H} NMR** (CD_3CN, 300 K, in ppm): δ = -79.3 (3F, s, OTf); **^{31}P{^{1}H} NMR** (CD_3CN, 300 K, in ppm): δ = 154.5 (1P, s, P).

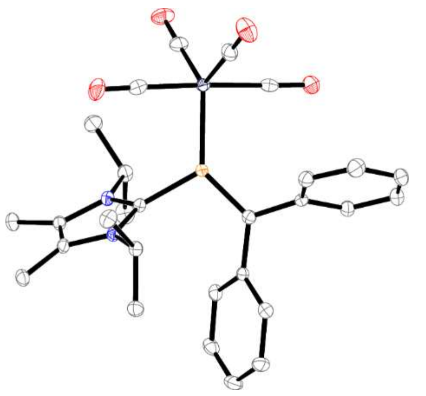

Figure S76. Molecular structure of **13**$^+$ in **13**[OTf]; hydrogen atoms and the anion are omitted for clarity and thermal ellipsoids are displayed at 50% probability.

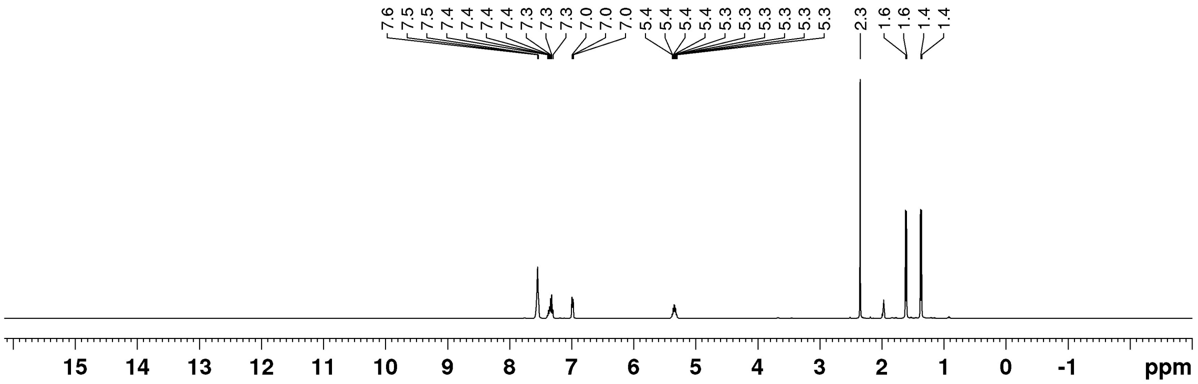

Figure S77. ^{1}H NMR spectrum of **13**[OTf] (300 K, CD_3CN).

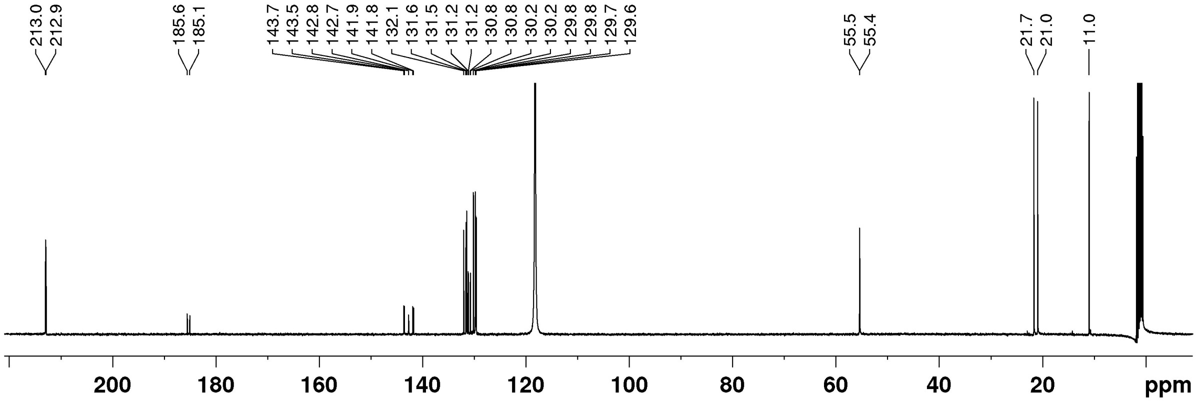

Figure S78. ^{13}C{^{1}H} NMR spectrum of **13**[OTf] (300 K, CD_3CN).

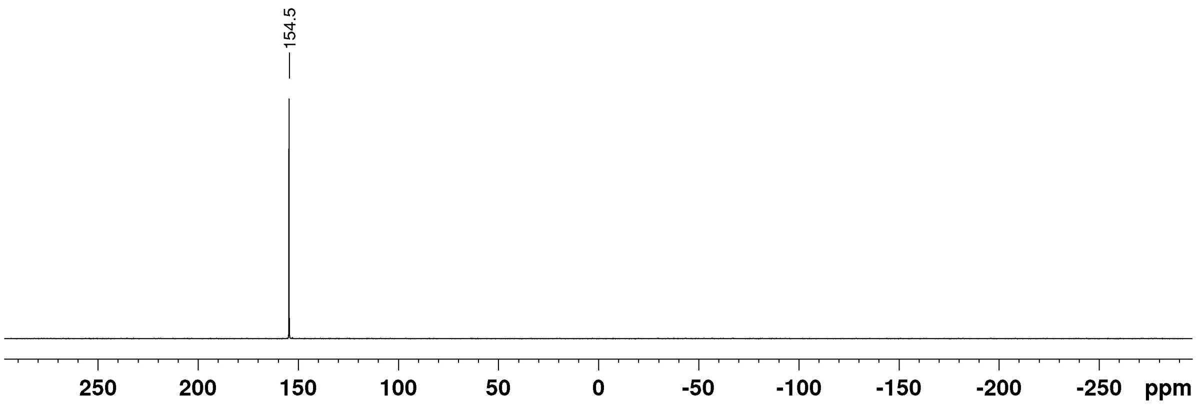

Figure S79. ^{31}P NMR spectrum of **13**[OTf] (300 K, CD_3CN).

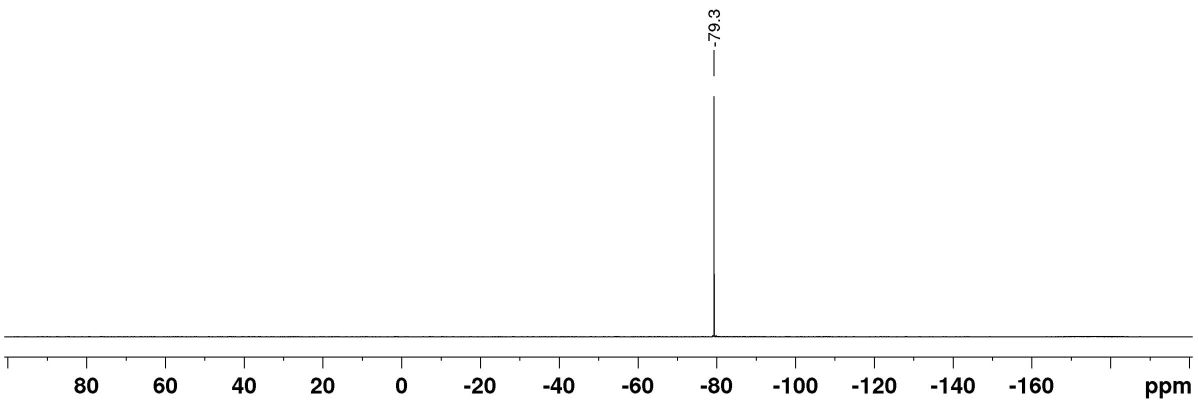

Figure S80. ^{19}F NMR spectrum of **13**[OTf] (300 K, CD_3CN).

S2.27 Preparation of [(L_C)P–P(Mes)–C(Ph)$_2$][OTf] (11a[OTf])

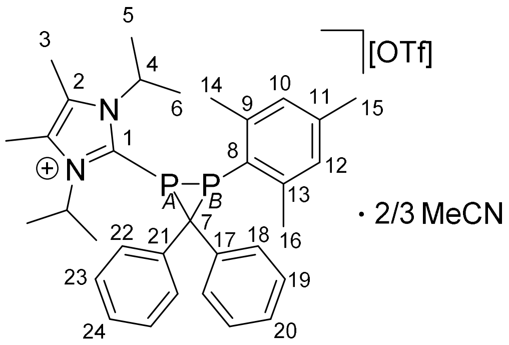

To a solution of **2**[OTf]$_4$ (100 mg, 7*10^{-2} mmol, 1.0 eq.) and Ph_3P (7 mg, 3*10^{-2} mmol, 0.4 eq.) in CH_3CN (2 mL) a solution of MesP=CPh$_2$ **9** (99 mg, 0.3 mmol, 4.5 eq.) in CH_3CN (1 mL) was added. The resulting colorless reaction mixture is stirred at room temperature for 2 h. Vapor diffusion of Et_2O into the reaction mixture gives the product as colorless blocks. Crystals suitable for single crystal X-ray analysis are obtained slow diffusion of Et_2O into a saturated solution of **11a**[OTf] in CH_2Cl_2.

Yield: 156 mg (83%); **m.p.**: 170-172 °C; **Raman** (100 mW, in cm^{-1}): 3059 (58), 2990 (38), 2940 (58), 2917 (48), 2734 (7), 2254 (17), 1620 (35), 1603 (67), 1595 (67), 1489 (16), 1447 (38), 1410 (46), 1385 (30), 1379 (30), 1362 (60), 1287 (93), 1227 (38), 1163 (89), 1089 (15), 1056 (30), 1031 (78), 1000 (100), 921 (11), 887 (26), 845 (10), 792 (19), 771 (12), 754 (21), 701 (9), 669 (35), 639 (14), 619 (24), 609 (19), 601 (21), 574 (39), 540 (16), 524 (34), 490 (34), 473 (31), 456 (15), 425 (11), 403 (14), 390 (17), 368 (18), 347 (25), 328 (29); **IR** (ATR, in cm^{-1}): 3013 (vw), 2985 (vw), 2938 (vw), 1619 (vw), 1601 (vw), 1574 (vw), 1488 (vw), 1445 (w), 1406 (w), 1377 (w), 1311 (vw), 1260 (vs), 1219 (w), 1173 (vw), 1145 (s), 1116 (w), 1088 (vw), 1078 (vw), 1029 (s), 1001 (vw), 984 (vw), 918 (vw), 906 (vw), 886 (vw), 858 (w), 790 (vw), 766 (w), 753 (w), 699 (m), 669 (vw), 636 (vs), 617 (w), 599 (w), 571 (w), 547 (w), 517 (m), 490 (vw), 469 (w), 416 (vw); **^{1}H NMR** (CD_3CN, 300 K, in ppm): δ = 1.38 (6H, d, $^3J_{HH}$ = 6.5 Hz, H5), 1.50 (6H, d, $^3J_{HH}$ = 6.9 Hz, H6), 1.96 (3H, s, CH_3CN), 2.19 (6H, s, H3), 2.37 (6H, s(br), H12), 5.47 (2H, m, H4), 6.68 (2H, s(br), H10), 6.69-7.02 (4H, m, H15&H16), 7.05-7.16 (1H, m, H17), 7.2-7.32 (3H, m, H20&21), 7.35-7.41 (2H, m,

H19); **^{13}C{^{1}H} NMR** (CD_3CN, 300 K, in ppm): δ = 11.2 (2C, s, C3), 20.9 (1C, s, C13), 21.4 (2C, s, C5), 21.6 (2C, s, C6), 24.1 (1C, s(br), C12), 54.7 (2C, m, C4), 61.1 (1C, dd, $^{1}J_{CP}$ = 53 Hz, $^{1}J_{CP}$ = 50 Hz, C7), 122.2 (1C, q, $^{1}J_{CF}$ = 321 Hz, OTf), 124.1 (1C, dd, $^{1}J_{CP}$ = 47 Hz, $^{2}J_{CP}$ = 11 Hz, C8), 127.9 (1C, s, C17), 128.4 (2C, s, C16), 128.8 (1C, s, C21), 130.0 (2C, s, C20), 130.1 (2C, d, $^{3}J_{CP}$ = 19 Hz, C15), 130.2 (2C, s, C10), 130.4 (2C, d, $^{3}J_{CP}$ = 18 Hz, C19), 131.9 (2C, s, C2), 139.5 (1C, dd, $^{2}J_{CP}$ = 19 Hz, $^{2}J_{CP}$ = 3 Hz, C14), 140.6 (1C, s, C11), 140.6 (1C, dd, $^{1}J_{CP}$ = 100 Hz, $^{2}J_{CP}$ = 10 Hz, C1), 140.9 (1C, dd, $^{2}J_{CP}$ = 19 Hz, $^{2}J_{CP}$ = 2 Hz, C18), 144.2 (2C, d, $^{2}J_{CP}$ = 13 Hz, C9); **^{19}F{^{1}H} NMR** (CD_3CN, 300 K, in ppm): δ = -79.3 (3F, s, OTf); **^{31}P{^{1}H} NMR** (CD_3CN, 300 K, in ppm): δ = -127.7 (1P, d, $^{1}J_{PP}$ = 146 Hz, P_A), -100.8 (1P, d, $^{1}J_{PP}$ = 146 Hz, P_B); **elemental analysis**: calcd. for $C_{34}H_{41}F_3N_2O_3PS \cdot 0.67\ CH_3CN$: C: 60.28, H: 6.16, N: 5.3, S: 4.55; found: C: 60.45, H: 6.296, N: 5.32, S: 4.591.

Note: residual CH_3CN from recrystallization could not be removed even after drying *in vacuo* for 24h. Both, NMR data and elemental analysis are consistent with a chemical formula for the solvate of $C_{34}H_{41}F_3N_2O_3PS \cdot 0.67\ CH_3CN$.

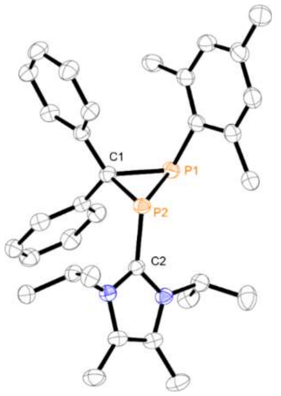

Figure S81. Molecular structure of **11a**$^{+}$ in **11a**[OTf]; hydrogen atoms and the anion are omitted for clarity and thermal ellipsoids are displayed at 50% probability.

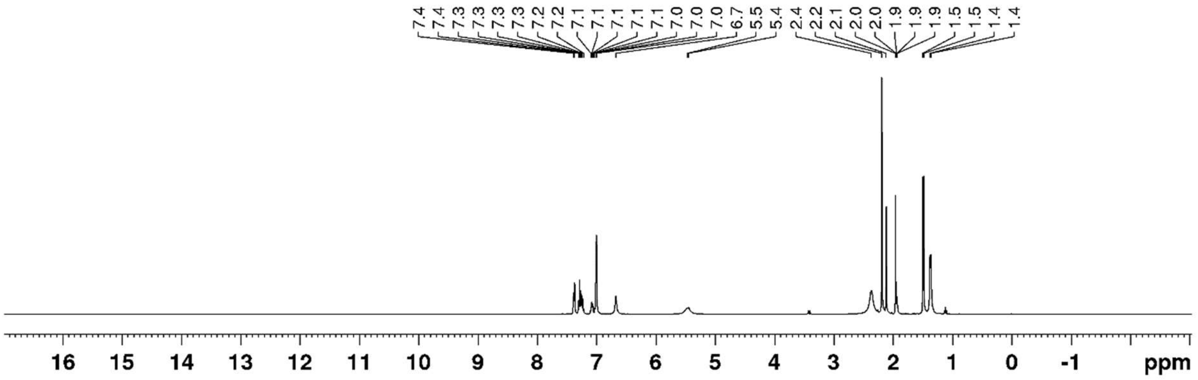

Figure S82. ^{1}H NMR spectrum of **11a**[OTf] (300 K, CD_3CN).

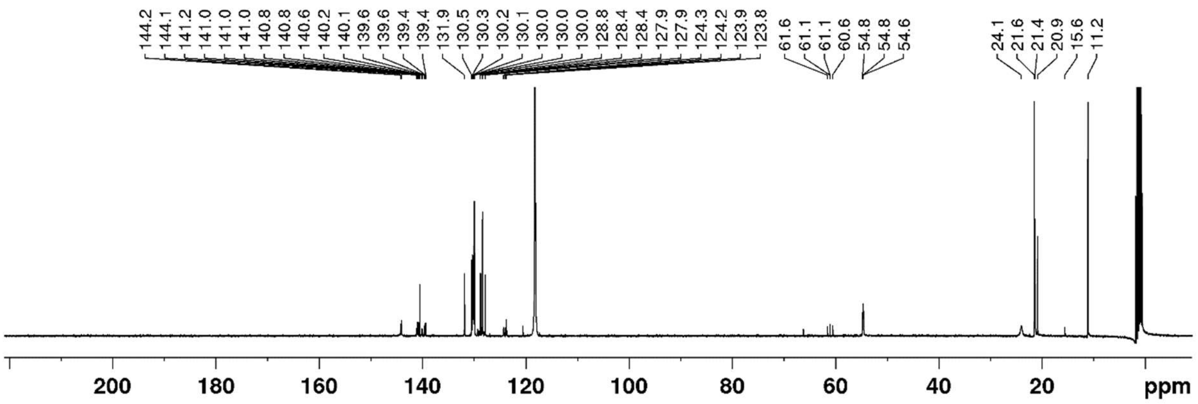

Figure S83. $^{13}C\{^1H\}$ NMR spectrum of **11a**[OTf] (300 K, CD_3CN).

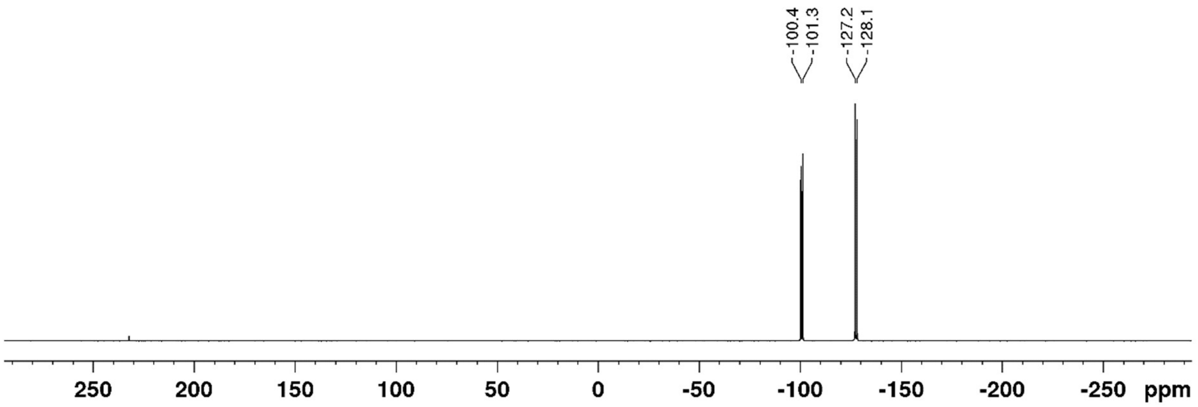

Figure S84. ^{31}P NMR spectrum of **11a**[OTf] (300 K, CD_3CN).

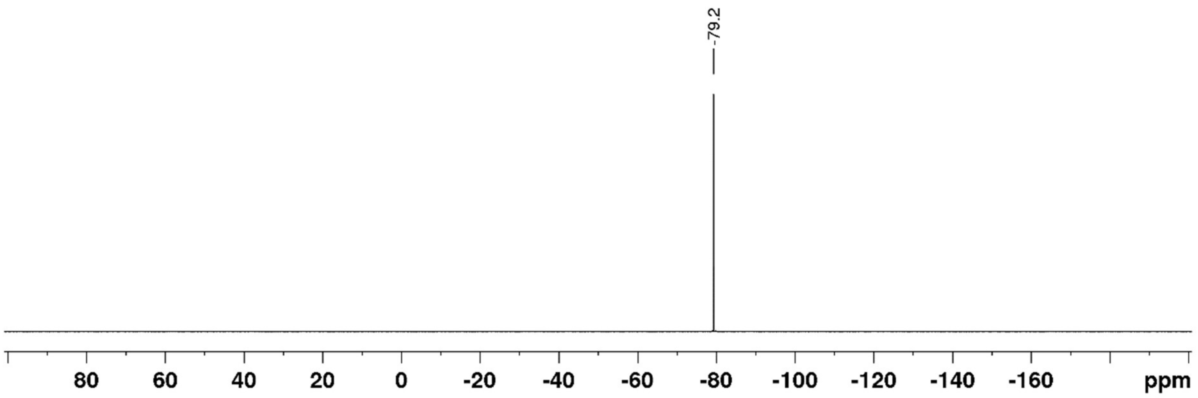

Figure S85. ^{19}F NMR spectrum of **11a**[OTf] (300 K, CD_3CN).

S2.28 Preparation of [(L_C)P–P(Ph)–C(Ph)$_2$][OTf] (11b[OTf])

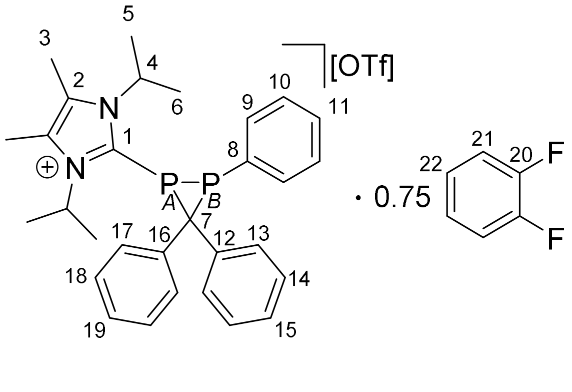

A solution of **2**[OTf]$_4$ (100 mg, 7*10^{-2} mmol, 1.0 eq.) and Ph_3P (7 mg, 3*10^{-2} mmol, 0.4 eq.) in CH_3CN (2 mL) was added to a suspension of hexaphenyl-1,2-diphosphetane **10** (86 mg, 0.15 mmol, 2.3 eq.) in CH_3CN (1 mL). Heating the mixture to 60°C in a microwave reactor for 2 h gives a pale-yellow solution, which is evaporated to dryness. The resulting yellow oil was dissolved in 2 mL of *o*-$C_6H_4F_2$, layered with *n*-hexane and stored at -30°C to give crystalline

product, which was separated, washed twice with *n*-hexane (2x4 mL) and dried *in vacuo*. Crystals suitable for single crystal X-ray analysis were obtained via the same method.

Yield: 158 mg (90%); **m.p.**: 109-111 °C; **Raman** (100 mW, in cm^{-1}): 3061 (23), 2976 (11), 2948 (18), 2871 (5), 1617 (14), 1593 (30), 1587 (31), 1493 (6), 1447 (14), 1416 (23), 1401 (14), 1363 (30), 1287 (30), 1229 (14), 1165 (34), 1091 (19), 1032 (34), 1001 (74), 898 (10), 886 (12), 804 (11), 790 (10), 767 (7), 753 (10), 700 (7), 668 (17), 644 (11), 619 (12), 611 (14), 584 (8), 572 (9), 545 (8), 527 (19), 490 (19), 451 (11), 433 (8), 401 (10), 342 (15), 324 (15), 312 (14), 297 (12), 267 (16), 251 (15), 218 (14), 160 (21), 132 (45); **IR** (ATR, in cm^{-1}): 3060 (vw), 2999 (vw), 2985 (vw), 2941 (vw), 2885 (vw), 1616 (vw), 1591 (vw), 1489 (w), 1459 (vw), 1444 (w), 1436 (w), 1410 (w), 1398 (w), 1378 (vw), 1337 (vw), 1264 (vs), 1216 (m), 1145 (s), 1114 (w), 1089 (w), 1066 (vw), 1030 (s), 999 (w), 978 (vw), 932 (vw), 904 (w), 886 (vw), 844 (vw), 803 (w), 789 (vw), 770 (m), 752 (w), 738 (w), 700 (m), 689 (m), 636 (vs), 608 (w), 571 (w), 543 (w), 516 (m), 502 (w), 489 (w), 473 (w), 428 (w), 414 (w), 405 (w); **^{1}H NMR** (CD_3CN, 300 K, in ppm): δ = 1.12 (6H, d, $^{3}J_{HH}$ = 5.2 Hz, H5), 1.65 (6H, d, $^{3}J_{HH}$ = 6.9 Hz, H6), 2.25 (6H, s, H3), 5.29 (2H, s(br), H4), 6.75-6.80 (2H, m, H17), 7.1-7.2 (9H, m, H10&H11&H14&H15&H17&H18&H19), 7.2-7.35 (6H, m, H9&H13&H21&H22); **^{13}C{^{1}H} NMR** (CD_3CN, 300 K, in ppm): δ = 11.2 (2C, s, C3), 21.1 (2C, s, C5), 21.4 (2C, s, C6), 55.3 (2C, m, C4), 61.3 (1C, dd, $^{1}J_{CP}$ = 54 Hz, $^{1}J_{CP}$ = 50 Hz, C7), 118.4 (2C, m, C22), 122.2 (1C, q, $^{1}J_{CF}$ = 321 Hz, OTf), 126.1 (2C, m, C21), 128.1 (1C, s, C19), 128.3 (2C, d, $^{3}J_{CP}$ = 15 Hz, C17), 128.8 (1C, s, C15), 129.2 (2C, d, $^{3}J_{CP}$ = 7 Hz, C10), 129.7 (4C, s, C14&C18), 130.2 (1C, dd, $^{1}J_{CP}$ = 41 Hz, $^{2}J_{CP}$ = 11 Hz, C8), 131.0 (1C, s, C11), 132.2 (2C, s, C2), 133.9 (2C, d, $^{3}J_{CP}$ = 16 Hz, C13), 136.0 (2C, dd, $^{2}J_{CP}$ = 20 Hz, $^{3}J_{CP}$ = 13 Hz, C9), 137.7 (1C, dd, $^{2}J_{CP}$ = 20 Hz, $^{2}J_{CP}$ = 4 Hz, C12), 140.7 (1C, dd, $^{1}J_{CP}$ = 90 Hz, $^{2}J_{CP}$ = 11 Hz, C1), 142.3 (1C, dd, $^{2}J_{CP}$ = 19 Hz, $^{2}J_{CP}$ = 3 Hz, C16), 151.2 (2C, dd, $^{1}J_{CF}$ = 247 Hz, $^{2}J_{CF}$ = 14 Hz, C20); **^{19}F{^{1}H} NMR** (CD_3CN, 300 K, in ppm): δ = -140.7 (1.5F, s, *o*-$C_6H_4F_2$), -79.2 (3F, s, OTf); **^{31}P{^{1}H} NMR** (CD_3CN, 300 K, in ppm): δ = -139.5 (1P, d, $^{1}J_{PP}$ = 133 Hz, P_A), -107.9 (1P, d, $^{1}J_{PP}$ = 133 Hz, P_B); **elemental analysis**: calcd. for $C_{31}H_{35}F_3N_2O_3P_2S \cdot 0.75$ *o*-$C_6H_4F_2$: C: 59.2, H: 5.32, N: 3.89, S: 4.45; found: C: 59.32, H: 5.225, N: 3.97, S: 4.384.

Note: residual *o*-$C_6H_4F_2$ from recrystallization could not be removed even after drying *in vacuo* for 24h. Both, NMR data and elemental analysis are consistent with a chemical formula for the solvate of $C_{31}H_{35}F_3N_2O_3P_2S \cdot 0.75$ *o*-$C_6H_4F_2$.

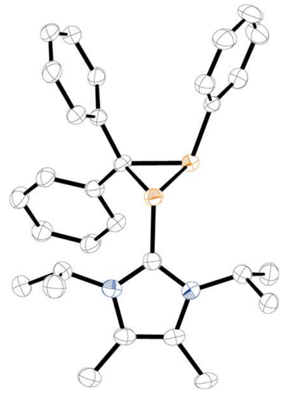

Figure S86. Molecular structure of **11b**$^{+}$ in **11b**[OTf] · *o*-$C_6H_4F_2$; hydrogen atoms and the anion are omitted for clarity and thermal ellipsoids are displayed at 50% probability.

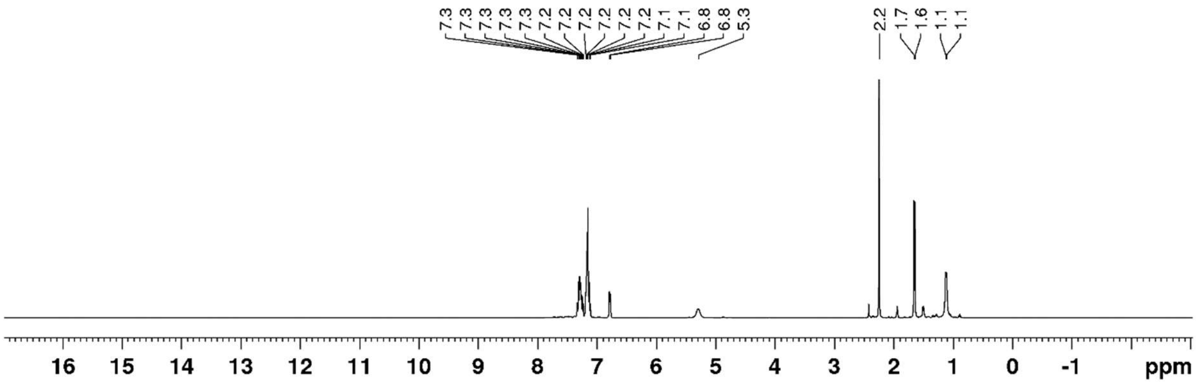

Figure S87. ^{1}H NMR spectrum of **11b**[OTf] (300 K, CD_3CN).

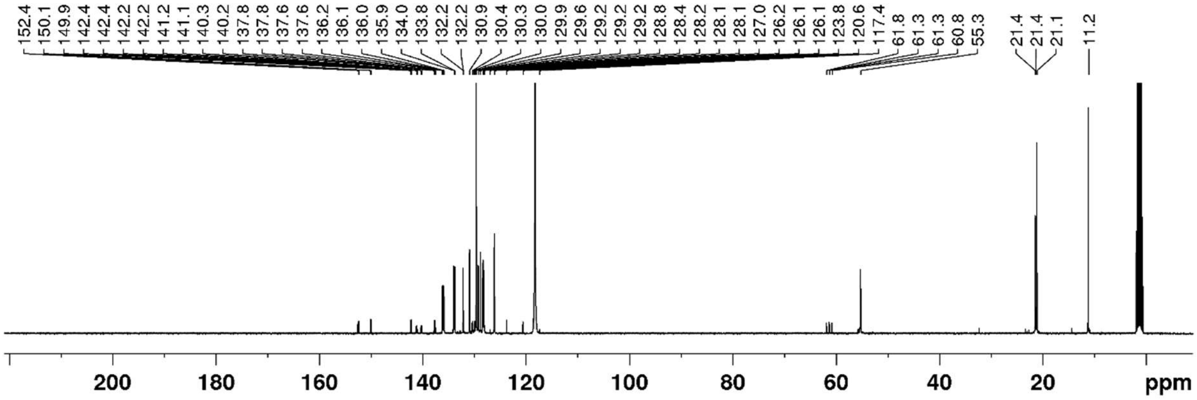

Figure S88. $^{13}C\{^{1}H\}$ NMR spectrum of **11b**[OTf] (300 K, CD_3CN).

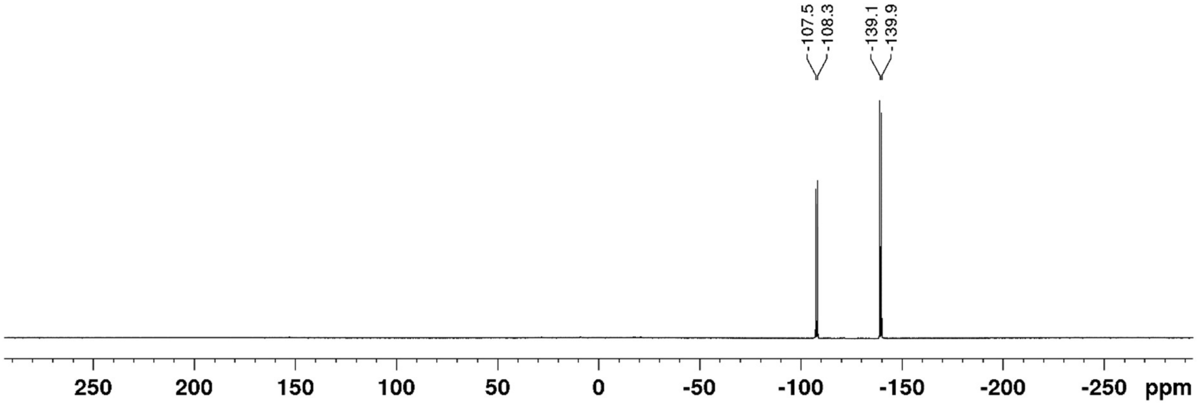

Figure S89. ^{31}P NMR spectrum of **11b**[OTf] (300 K, CD_3CN).

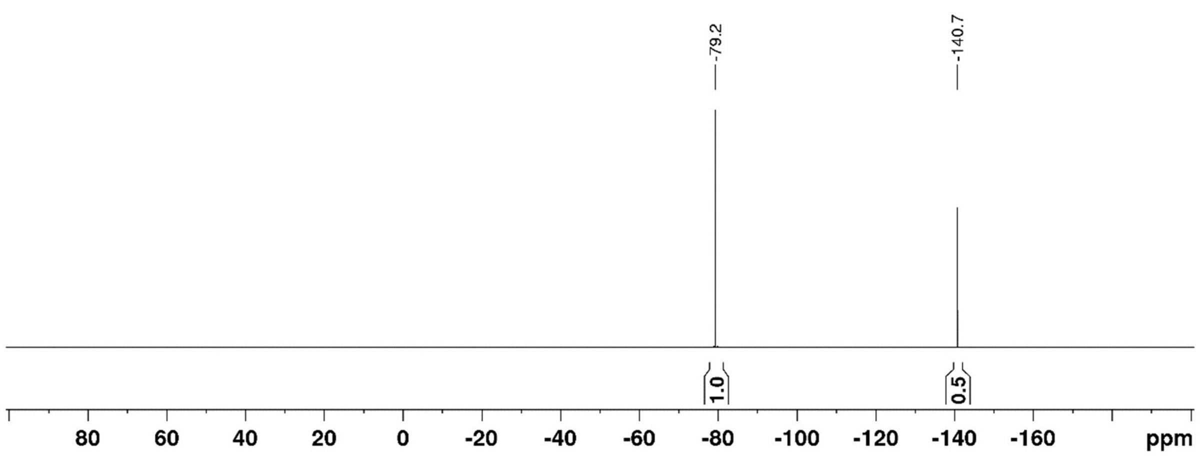

Figure S90. ^{19}F NMR spectrum of **11b**[OTf] (300 K, CD_3CN).

S2.29 Preparation of [Pd(Ph_3P)$_2$-η^2-((L_C)P=C(Ph)$_2$)][OTf] (12a[OTf])

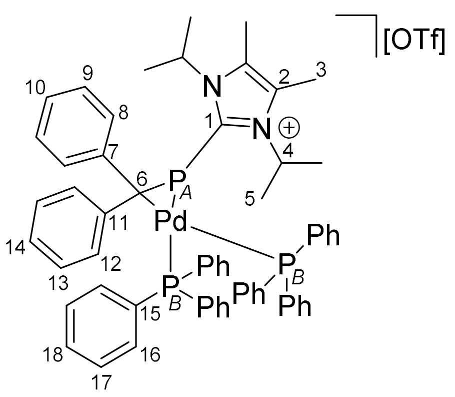

To a solution of **5a**[OTf] (75 mg, 0.14 mmol, 1.0 eq.) in C_6H_5F (2 ml) a suspension of [Pd(PPh_3)$_4$] (165 mg, 0.14 mmol, 1.0 eq.) in C_6H_5F (1 ml) was added and the mixture was stirred for 4 h at room temperature. Removal of all volatiles results in a dark red oil, which was dissolved in benzene (3 mL). Addition of a 1:1 mixture of Et_2O and *n*-hexane results in the precipitation of a dark brown solid, which was filtered off, washed three times with a mixture of Et_2O and *n*-hexane in a 1:1 ration and dried *in vacuo*. Crystals suitable for X-ray diffraction analysis were obtained by vapor diffusion of *n*-pentane into a saturated solution of **12a**[OTf] in toluene at room temperature over two months.

Yield: 149 mg (90%); **m.p.**: 98-100 °C; **Raman** (100 mW, in cm^{-1}): sample shows fluorescence. **IR** (ATR, in cm^{-1}): 3051 (w), 2986 (w), 2936 (w), 2870 (w), 1622 (w), 1586 (w), 1571 (w), 1480 (w), 1434 (m), 1391 (w), 1374 (w), 1265 (vs), 1220 (m), 1184 (w), 1141 (s), 1112 (w), 1092 (m), 1030 (s), 998 (w), 904 (w), 847 (w), 742 (m), 693 (vs), 636 (vs), 598 (w), 583 (w), 571 (w), 514 (vs), 488 (s), 448 (m), 426 (m), 414 (m); **^{1}H NMR** (THF-d_8, 300 K, in ppm): δ = 1.24 (12H, s(br), H5), 2.23 (6H, s, H3), 5.68 (2H, s(br), H4), 6.97-7.02 (2H, m, H12), 7.05-7.13 (28H, m, H9&H13&H16&H17), 7.12-7.15 (2H, m, H8), 7.21-7.26 (8H, m, H10&H14&H18); **^{13}C{^{1}H} NMR** (THF-d_8, 300 K, in ppm): δ = 10.7 (2C, s, C3), 21.4 (4C, s, C5), 54.0 (2C, d, $^3J_{CP}$ = 6 Hz, C4), 104.4 (1C, m, C6), 122.6 (1C, q, $^1J_{CF}$ = 324 Hz, OTf), 126.7 (1C, s, C14), 126.8 (1C, s, C10), 127.9 (2C, d, $^3J_{CP}$ = 24 Hz, C12), 128.4 (2C, s, C13), 128.8 (2C, s, C9), 128.9 (12C, d, $^3J_{CP}$ = 9 Hz, C17), 130.0 (2C, s, C2), 130.3 (6C, s, C18), 131.9 (2C, s, C8), 134.5 (6C, d, $^1J_{CP}$ = 28 Hz, C15), 134.6 (12C, d, $^2J_{CP}$ = 13 Hz, C16), 145.3 (3C, d, $^1J_{CP}$ = 29 Hz, C7), 145.6 (1C, m, C11), 148.3 (1C, d, $^1J_{CP}$ = 101 Hz, C1); **^{19}F{^{1}H} NMR** (THF-d_8, 300 K, in ppm): δ = -78.7 (3F, s, OTf); **^{31}P{^{1}H} NMR** (THF-d_8, 300 K, in ppm): δ = -5.6 (1P, s(br), P_A), 19.4 (2P, s(br), P_B); **elemental analysis**: calcd. for $C_{61}H_{60}F_3N_2O_3P_3SPd$: C: 63.29, H: 5.22, N: 2.42, S: 2.77; found: C: 63.61, H: 5.441, N: 2.39, S: 2.356.

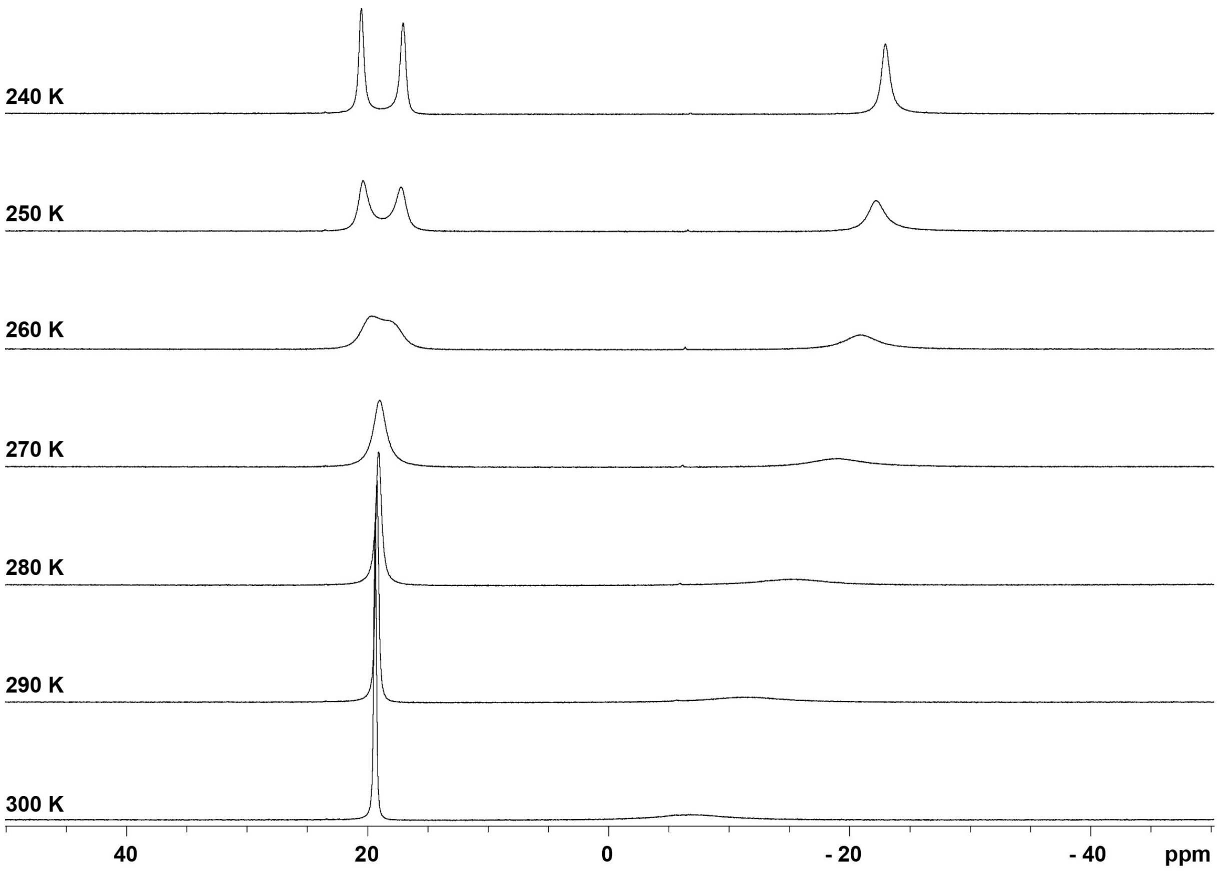

Figure S91. VT-^{31}P NMR spectra of **12a**[OTf] (300-240K, THF-d_8).

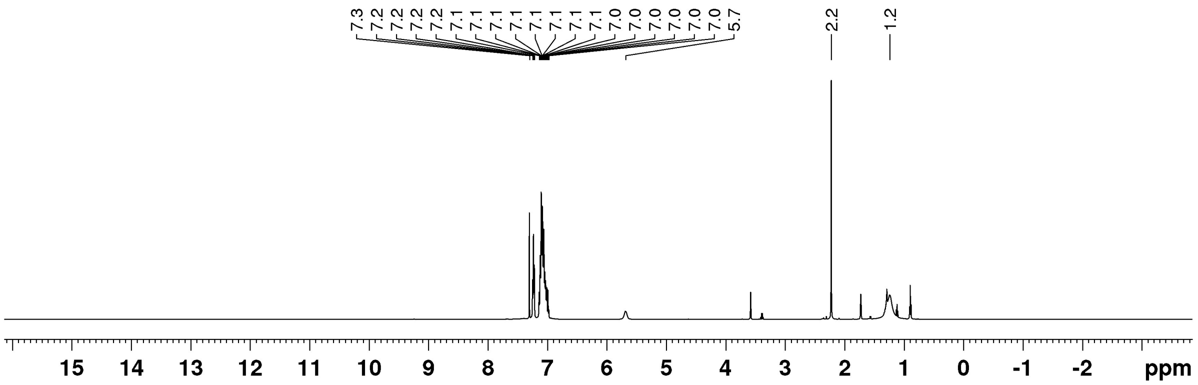

Figure S92. ^{1}H NMR spectrum of **12a**[OTf] (300 K, THF-d_8).

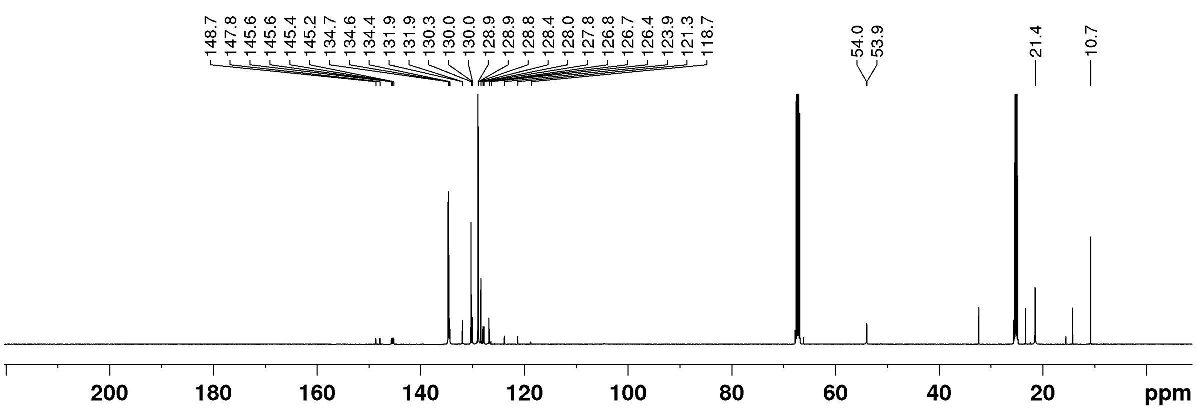

Figure S93. $^{13}C\{^{1}H\}$ NMR spectrum of **12a**[OTf] (300 K, THF-d_8).

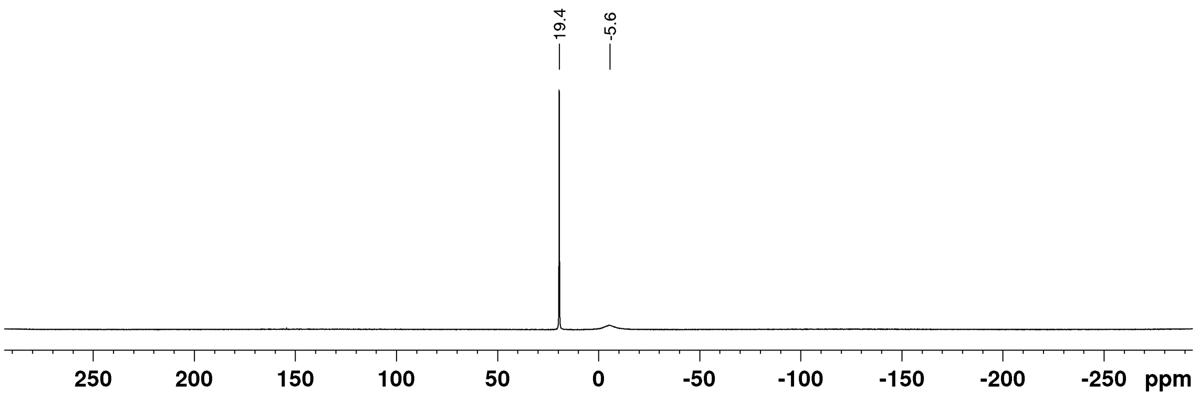

Figure S94. ^{31}P NMR spectrum of **12a**[OTf] (300 K, THF-d_8).

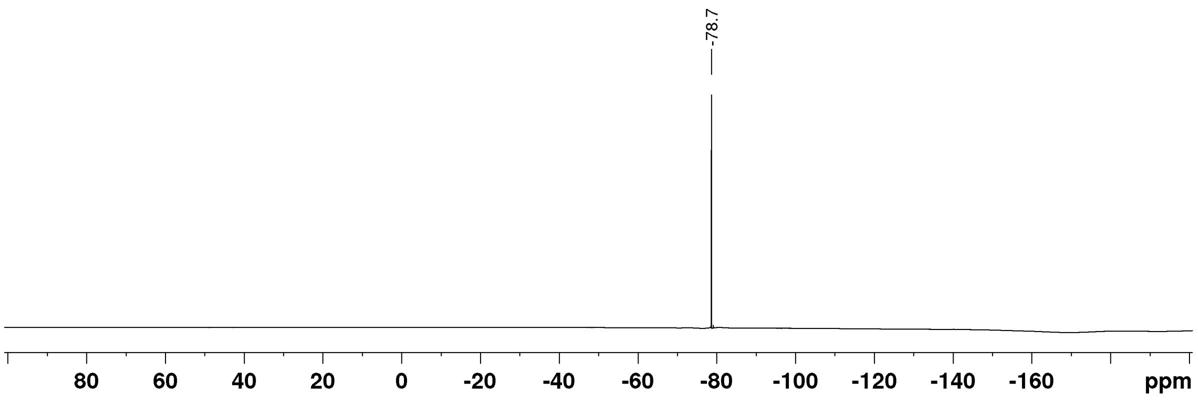

Figure S95. ^{19}F NMR spectrum of **12a**[OTf] (300 K, THF-d_8).

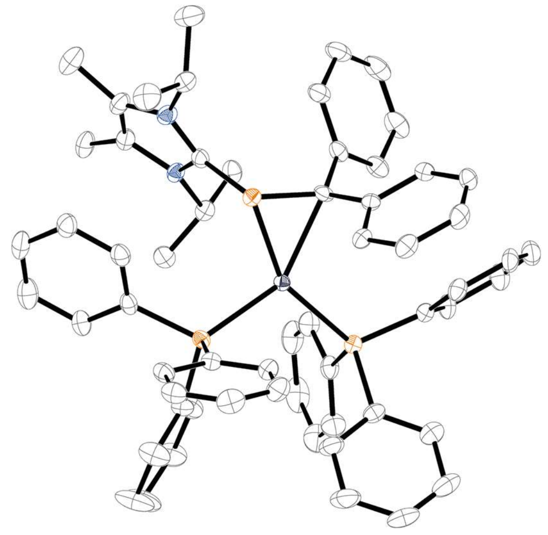

Figure S96. Molecular structure of **12a**$^+$ in **12a**[OTf]; hydrogen atoms and the anion are omitted for clarity and thermal ellipsoids are displayed at 50% probability.

S2.30 Preparation of $[Pt(Ph_3P)_2$-η^2-$((L_C)P{=}C(Ph)_2)][OTf]$ (12b[OTf])

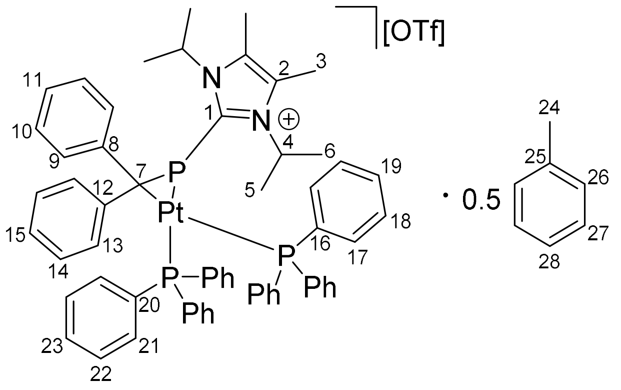

To a suspension of **5a**[OTf] (200 mg, 0.38 mmol, 1.0 eq.) in toluene (4 ml) solid $[Pt(PPh_3)_3]$ (473 mg, 0.38 mmol, 1.0 eq.) was added and the mixture was stirred for 1 h at room temperature. Addition of Et_2O (10 mL) to the the now orange solution results in precipitation of a yellow solid, which was filtered off, washed three times with Et_2O and *n*-pentane (3x4 mL each) and dried *in vacuo*. Crystals suitable for X-ray diffraction analysis were obtained by vapor diffusion of *n*-pentane into a saturated solution of **12b**[OTf] in toluene at room temperature over several weeks.

Yield: 472 mg (97%); **m.p.**: 203-205 °C; **Raman** (100 mW, in cm^{-1}): 3061 (32), 3052 (41), 3004 (10), 2985 (10), 2945 (16), 1624 (13), 1587 (81), 1485 (12), 1447 (15), 1419 (20), 1406 (10), 1387 (7), 1347 (28), 1312 (9), 1291 (22), 1274 (8), 1243 (23), 1222 (8), 1169 (46), 1097 (25), 1085 (17), 1031 (43), 1002 (100), 936 (7), 905 (10), 784 (8), 772 (9), 753 (11), 704 (7), 670 (8), 629 (12), 620 (13), 603 (22), 595 (13), 511 (7), 466 (10), 449 (7), 422 (9), 359 (7), 348 (8), 324 (10), 311 (9), 280 (10), 268 (11), 256 (18), 223 (17); **IR** (ATR, in cm^{-1}): 3050 (w), 2981 (w), 2939 (w), 1623 (w), 1587 (w), 1575 (w), 1482 (w), 1464 (w), 1434 (m), 1419 (w), 1391 (w), 1378 (w), 1366 (w), 1348 (w), 1264 (vs), 1221 (w), 1213 (w), 1181 (w), 1161 (m), 1141 (m), 1111 (w), 1097 (m), 1084 (w), 1029 (s), 999 (w), 978 (w), 931 (w), 903 (w), 881 (w), 848 (w), 771 (w), 742 (m), 733 (w), 694 (vs), 636 (vs), 602 (w), 594 (w), 571 (w), 535 (m), 519 (vs), 510 (vs), 494 (s), 464 (m), 428 (w), 416 (w); **^{1}H NMR** (CD_2Cl_2, 300 K, in ppm): δ = 0.89 (6H, s(br), H5), 1.53 (6H, d, $^3J_{HH}$ = 6.9 Hz, H6), 2.14 (6H, s, H3), 2.35 (1.5H, s, H24), 5.83 (2H, s(br), H4), 6.73 (2H, m, H9), 6.87 (6H, m, H17), 6.96 (1H, m, H15), 7.01 (6H, m, H18), 7.06-7.12 (4H, m, H10&H14), 7.09 (2H, m, H13), 7.15-7.30 (1H, m, H26), 7.15-7.30 (0.5H, m, H28), 7.16 (6H, m, H22), 7.17 (1H, m, H11), 7.22-7.29 (3H, m, H23), 7.24 (1H, m, H27), 7.27 (6H, m, H21); **^{13}C{^{1}H} NMR** (CD_2Cl_2, 300 K, in ppm): δ = 11.0 (2C, s, C3), 21.6 (0.5C, s, C24), 21.6 (2C, s, C6), 21.8 (2C, s, C5), 53.2 (2C, m, C4), 78.3 (1C, ddd, $^1J_{CP}$ = 68 Hz, $^2J_{CP}$ = 51 Hz, $^2J_{CP}$ = 4 Hz, C7), 121.5 (1C, q, $^1J_{CF}$ = 321 Hz, OTf), 125.6 (1C, s, C11), 126.0 (1C, s, C15), 126.3 (0.5C, m, C28), 127.8 (2C, s, C9), 128.0-135.0 (1C, m, C27), 128.2 (6C, m, C18), 128.4 (3C, m, C16), 128.5 (3C, m, C20), 128.5 (6C, m, C22), 128.7 (2C, s, C2), 129.3 (1C, m, C26), 130.4 (3C, s, C19), 130.5 (3C, s, C23), 131.6-132.9 (6C, m, C8&C10&C12&C14), 132.6 (2C, m, C13), 134.3 (6C, m, C17), 134.4 (6C, m, C21), 138.4 (0.5C, s, C25), 146.7 (1C, ddd, $^1J_{CP}$ = 114 Hz, $^3J_{CP}$ = 6 Hz, C1); **^{19}F{^{1}H} NMR** (CD_2Cl_2, 300 K, in ppm): δ = -78.8 (3F, s, OTf); **^{31}P{^{1}H} NMR** (CD_2Cl_2, 300 K, in ppm): δ = -55.3 (1P, dd, $^1J_{PPt}$ = 564 Hz, $^2J_{PP}$ = 65 Hz, $^2J_{PP}$ = 13 Hz, P_A), 17.8 (1P, s(br), $^1J_{PPt}$ = 3216 Hz, P_X), 20.6 (1P, d, $^1J_{PPt}$ = 3575 Hz, $^2J_{PP}$ = 13 Hz, P_Y); **^{195}Pt NMR** (CD_2Cl_2, 300 K, in ppm): δ = -4822.0 (1Pt, ddd, $^1J_{PtP}$ = 3571 Hz, $^1J_{PtP}$ = 3207 Hz, $^1J_{PtP}$ = 563 Hz, Pt); **elemental analysis**: calcd. for $C_{61}H_{60}F_3N_2O_3P_3SPt \cdot 0.5\ C_7H_8$: C: 59.95, H: 4.99, N: 2.17, S: 2.48; found: C: 59.70, H: 5.058, N: 2.14, S: 2.202.

Note: residual toluene from recrystallization could not be removed even after drying *in vacuo* for 24h. Both, NMR data and elemental analysis are consistent with a chemical formula for the solvate of $C_{61}H_{60}F_3N_2O_3P_3SPt \cdot 0.5\ C_7H_8$.

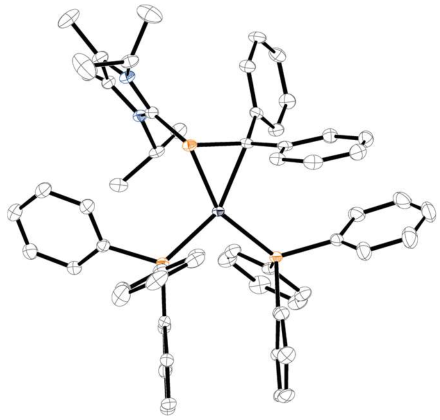

Figure S97. Molecular structure of **12b**$^+$ in **12b**[OTf]; hydrogen atoms and the anion are omitted for clarity and thermal ellipsoids are displayed at 50% probability.

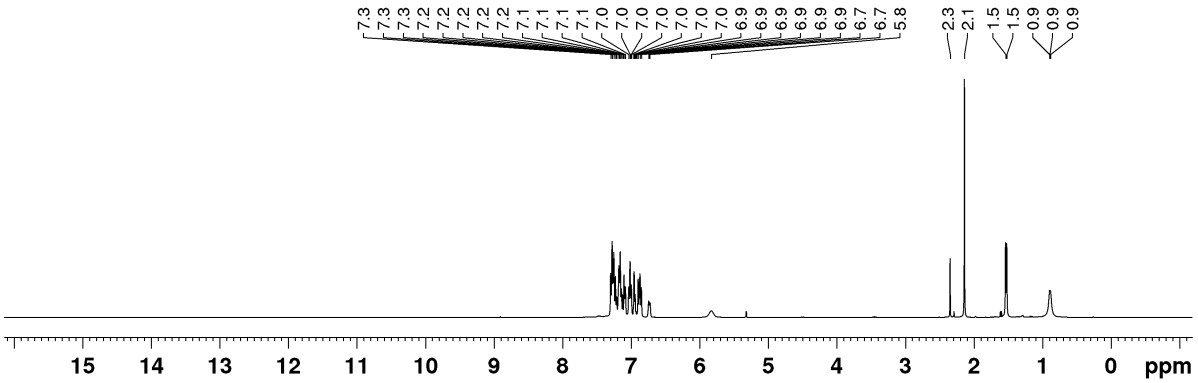

Figure S98. ^{1}H NMR spectrum of **12b**[OTf] (300 K, CD_2Cl_2).

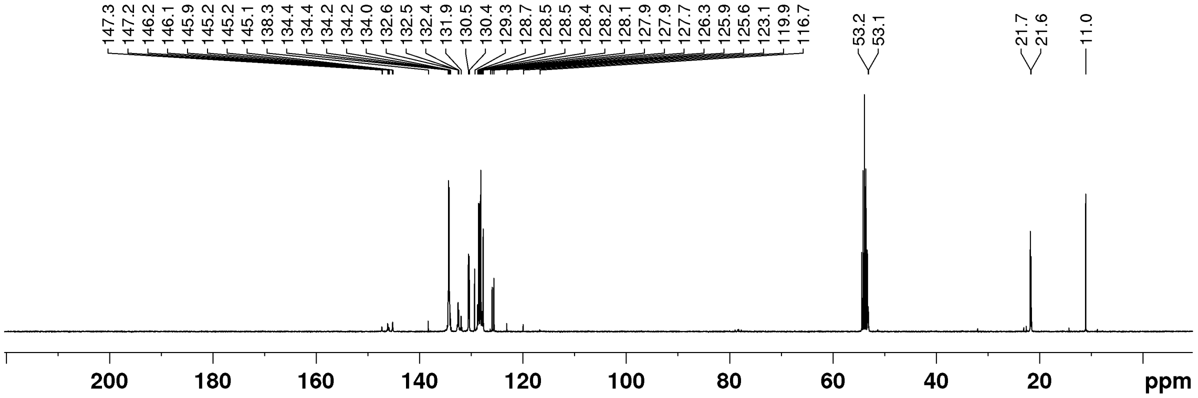

Figure S99. ^{13}C{^{1}H} NMR spectrum of **12b**[OTf] (300 K, CD_2Cl_2).

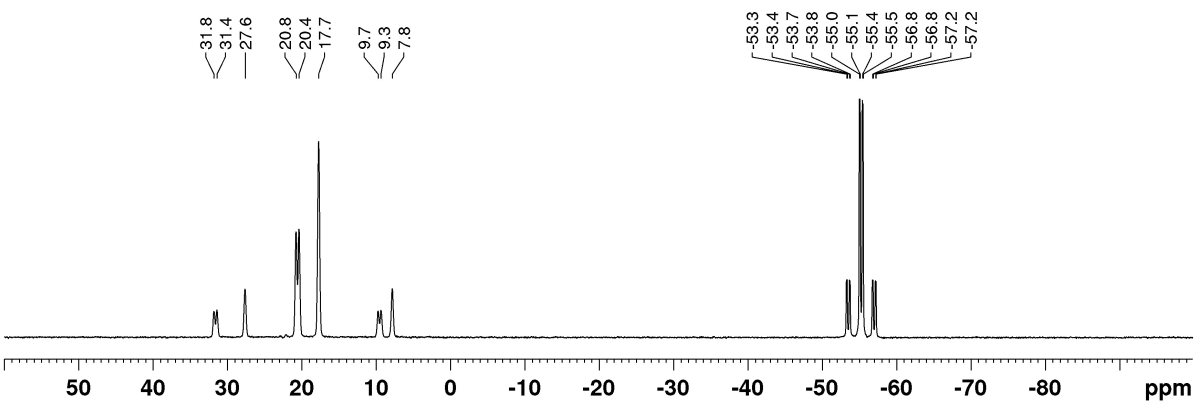

Figure S100. ^{31}P NMR spectrum of **12b**[OTf] (300 K, CD_2Cl_2).

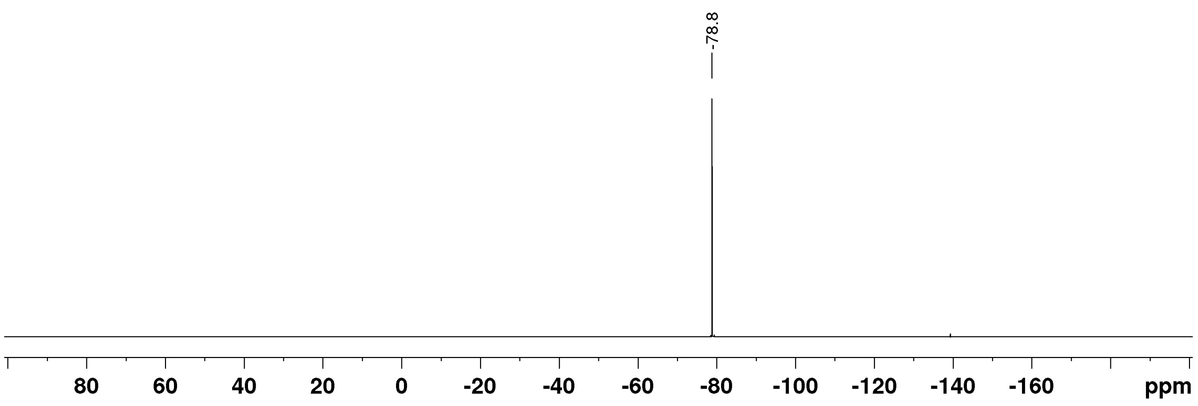

Figure S101. ^{19}F NMR spectrum of **12b**[OTf] (300 K, CD_2Cl_2).

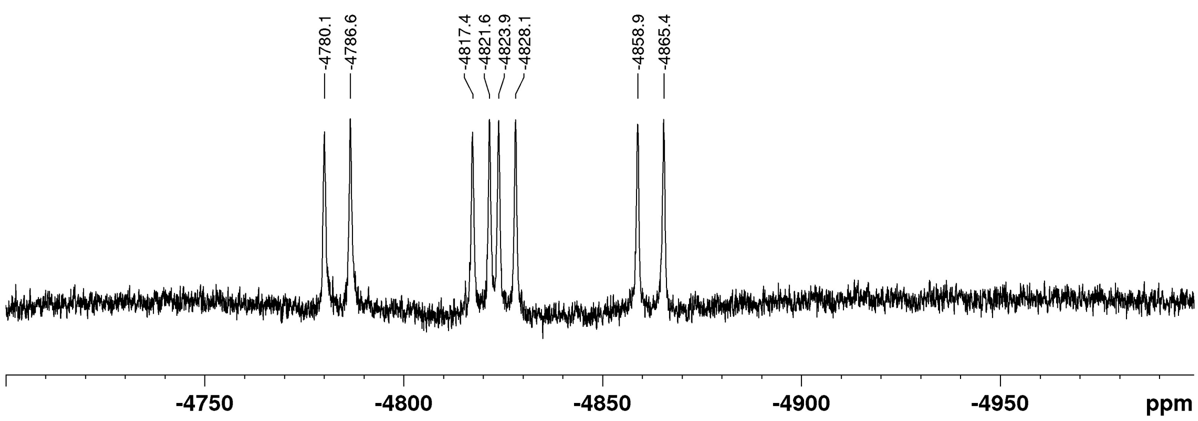

Figure S102. ^{195}Pt NMR spectrum of **12b**[OTf] (300 K, CD_2Cl_2).

S2.31 Preparation of [(L_C)P–($C_6Cl_4O_2$)–C(Ph)$_2$][OTf] (15[OTf])

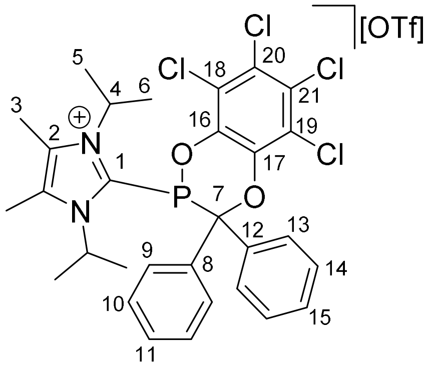

To a yellow solution of **5a**[OTf] (50 mg, 0.1 mmol, 1.0 eq.) in C_6H_5F (1 mL) a deep red solution of 3,4,5,6-tetrachloro-*o*-benzochinone (24 mg, 0.1 mmol, 1.0 eq.) in C_6H_5F (1 mL) was added dropwise. After complete addition a nearly colorless solution is obtained and stirred at room temperature for 1 h. Vapor diffusion of *n*-pentane into the reaction mixture gives the product as colorless blocks in near quantitative yield. Crystals suitable for single crystal X-ray analysis are obtained via the same method.

Yield: 69 mg (95%); **m.p.**: 227-229 °C; **Raman** (100 mW, in cm^{-1}): 3072 (44), 3029 (14), 2997 (25), 2982 (32), 2941 (49), 2877 (14), 1612 (43), 1598 (47), 1587 (35), 1555 (49), 1448 (35), 1411 (48), 1388 (36), 1355 (44), 1323 (23), 1302 (25), 1269 (100), 1219 (33), 1194 (35), 1176 (41), 1162 (38), 1136 (27), 1087 (24), 1033 (66), 1021 (33), 1003 (82), 953 (26), 940 (23), 907 (23), 886 (31), 792 (32), 754 (37), 721 (24), 708 (24), 671 (39), 639 (25), 620 (34), 606 (25), 588 (32), 571 (34), 546 (33), 508 (34), 486 (33), 461 (32), 424 (46), 401 (33); **IR** (ATR, in cm^{-1}): 2978 (vw), 2938 (vw), 1611 (vw), 1586 (vw), 1552 (vw), 1493 (vw), 1464 (vw), 1449 (vw), 1405 (m), 1387 (w), 1355 (vw), 1339 (vw), 1259 (vs), 1219 (m), 1201 (w), 1145 (m), 1119 (w), 1086 (w), 1030 (m), 1016 (m), 995 (m), 951 (w), 938 (w), 905 (w), 885 (vw), 841 (m), 791 (m), 761 (w), 750 (m), 707 (m), 696 (m), 670 (w), 656 (w), 636 (vs), 568 (m), 545 (w), 517 (m), 484 (w), 474 (w), 459 (w), 421 (m); **^{1}H NMR** (CD_3CN, 300 K, in ppm): δ = 0.99 (6H, d, $^{3}J_{HH}$ = 6.9 Hz, H5), 1.52 (6H, d, $^{3}J_{HH}$ = 6.9 Hz, H6), 2.29 (6H, s, H3), 4.56 (2H, dsept, $^{3}J_{HH}$ = 7.1 Hz, $^{4}J_{HP}$ = 7.2 Hz, H4), 7.27 (2H, m, H13), 7.73 (2H, m, H9), 7.32-7.50 (6H, m, $H_{arom.}$); **^{13}C{^{1}H} NMR** (CD_3CN, 300 K, in ppm): δ = 11.5 (2C, s, C3), 20.7 (4C, d, $^{4}J_{CP}$ = 4 Hz, C5&C6), 54.4 (2C, d, $^{3}J_{CP}$ = 18 Hz, C4), 89.2 (1C, d, $^{1}J_{CP}$ = 35 Hz, C7), 122.2 (1C, q, $^{1}J_{CF}$ = 321 Hz, OTf), 127.0 (1C, d, $^{3}J_{CP}$ = 4 Hz, C13), 128.9 (2C, d, $^{3}J_{CP}$ = 20 Hz, C9), 130.3 (1C, s, C15), 130.5 (2C, d, $^{4}J_{CP}$ = 2 Hz, C14), 130.6 (2C, s, C10), 131.1 (1C, s, C11), 134.2 (2C, s, C2), 138.0 (2C, d, $^{2}J_{CP}$ = 6 Hz, C8&C12), 138.2 (1C, d, $^{1}J_{CP}$ = 90 Hz, C1), 139.2 (2C, s, C20&C21), 139.3 (1C, s, C18), 139.4 (1C, s, C19), 141.1 (1C, s, C16), 141.2 (1C, s, C17); **^{19}F{^{1}H} NMR** (CD_3CN, 300 K, in ppm): δ = -79.3 (3F, s, OTf); **^{31}P{^{1}H} NMR** (CD_3CN, 300 K, in ppm): δ = 110.3 (1P, s, P); **elemental analysis**: calcd. for $C_{31}H_{30}Cl_4F_3N_2O_5PS$: C: 48.20, H: 3.91, N: 3.63, S: 4.15; found: C: 47.96, H: 3.735, N: 3.91, S: 4.319.

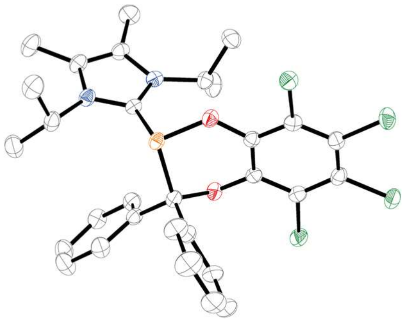

Figure S103. Molecular structure of **15**$^{+}$ in **15**[OTf] · 0.5 C_6H_5F · *n*-pentane; hydrogen atoms and the anion are omitted for clarity and thermal ellipsoids are displayed at 50% probability.

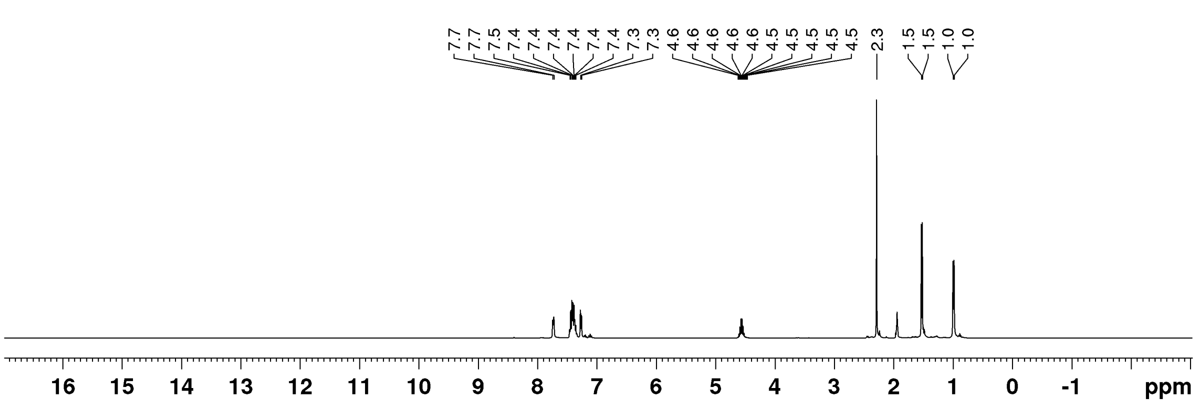

Figure S104. ^{1}H NMR spectrum of **15**[OTf] (300 K, CD_3CN).

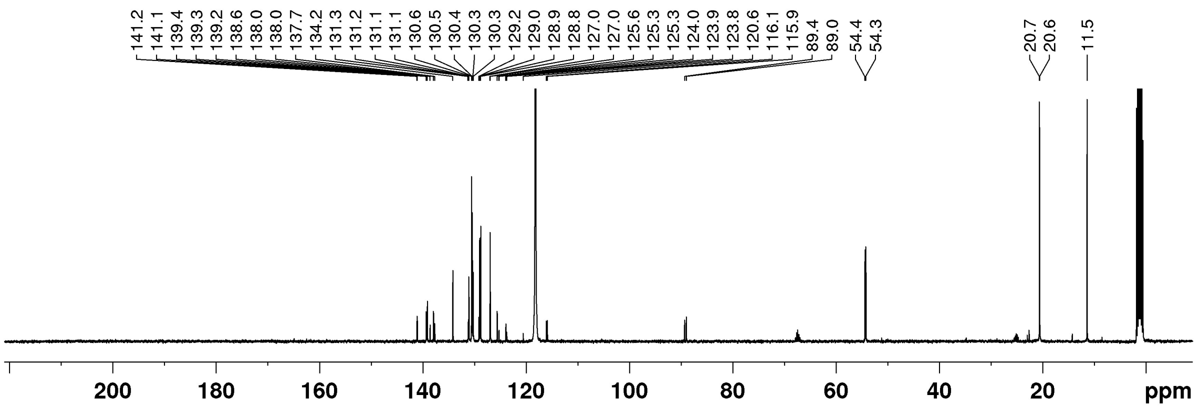

Figure S105. $^{13}C\{^{1}H\}$ NMR spectrum of **15**[OTf] (300 K, CD_3CN).

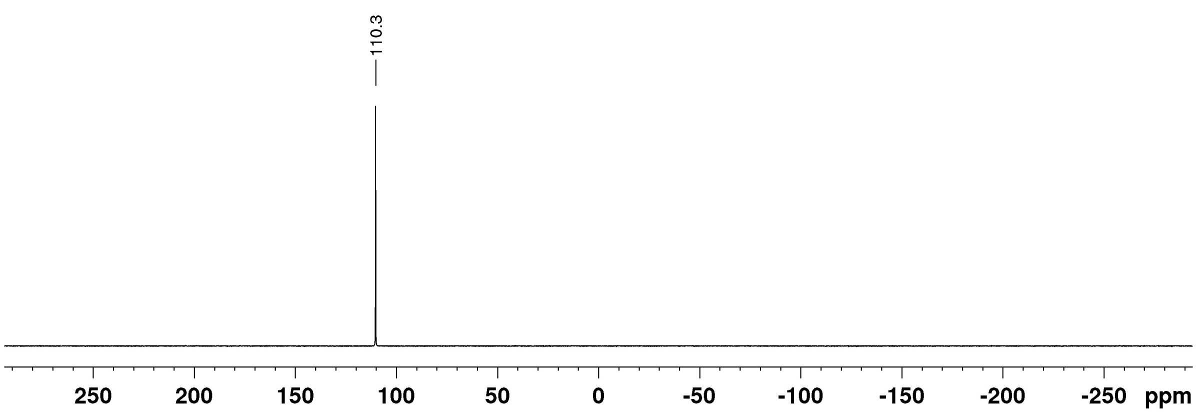

Figure S106. ^{31}P NMR spectrum of **15**[OTf] (300 K, CD_3CN).

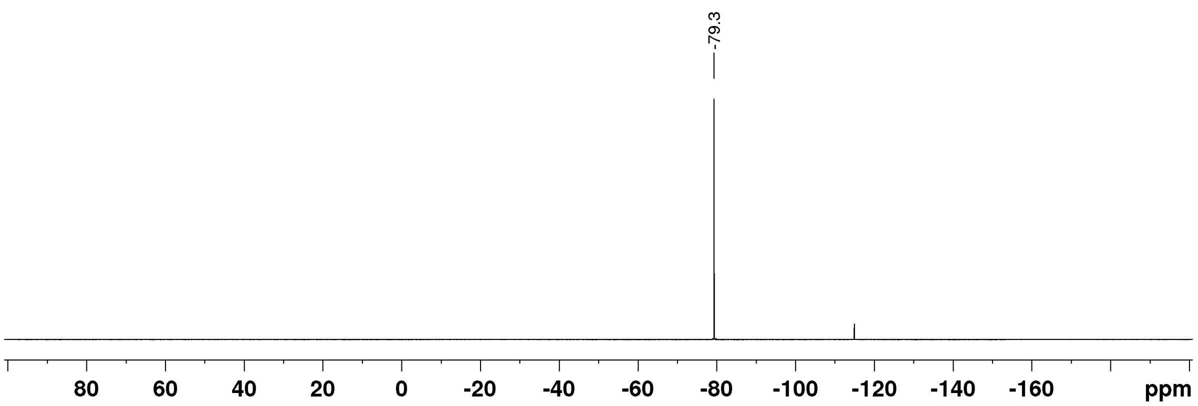

Figure S107. ^{19}F NMR spectrum of **15**[OTf] (300 K, CD_3CN).

S2.32 Preparation of MesP=C(Ph)$_2$ (16)

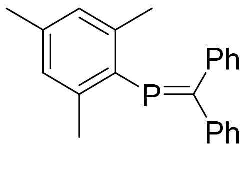

To a yellow solution of **5a**[OTf] (500 mg, 0.95 mmol, 1.0 eq.) in THF (15 mL) a solution of MesMgBr in THF (1 M, 1 mL, 0.1 mmol, 1.05 eq.) was added dropwise at -78°C. Upon addition the reaction mixture immediately changed to a dark red mixture and was stirred at -78°C for 30 min. Subsequently the mixture was allowed to warm up to room temperature, all volatiles were removed *in vacuo* and the resulting brown solid residue was extracted twice with *n*-hexane (2x10 mL). The volume of the extract was subsequently reduced to 2 ml *in vacuo* and storage at -30°C for 16 h yielded near colorless needles, which were separated by decantation and dried *in vacuo* to give the product as an off-white solid.

Yield: 279 mg (93%).

Spectroscopic characterization of the obtained product matched literature reported values.[17] One side product of this reaction was identified crystallographically as $[MgBr_2(NHC)_2]$[18] and was not isolated.

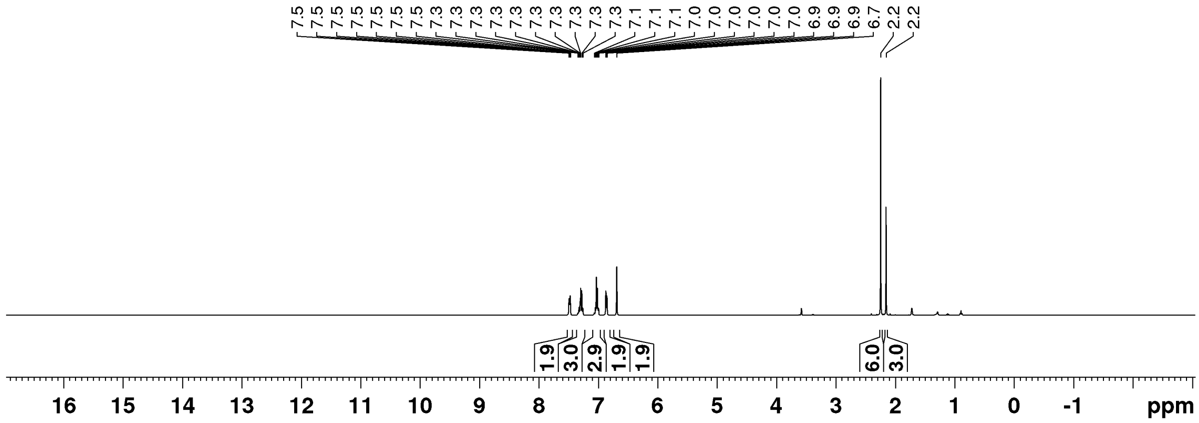

Figure S108. ^{1}H NMR spectrum of **16** (300 K, THF-d_8).

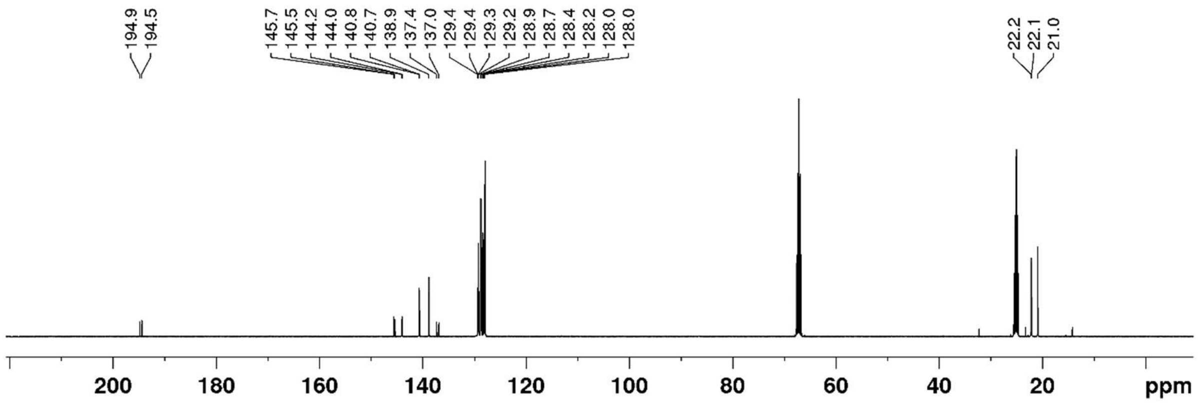

Figure S109. $^{13}C\{^1H\}$ NMR spectrum of **16** (300 K, THF-d_8).

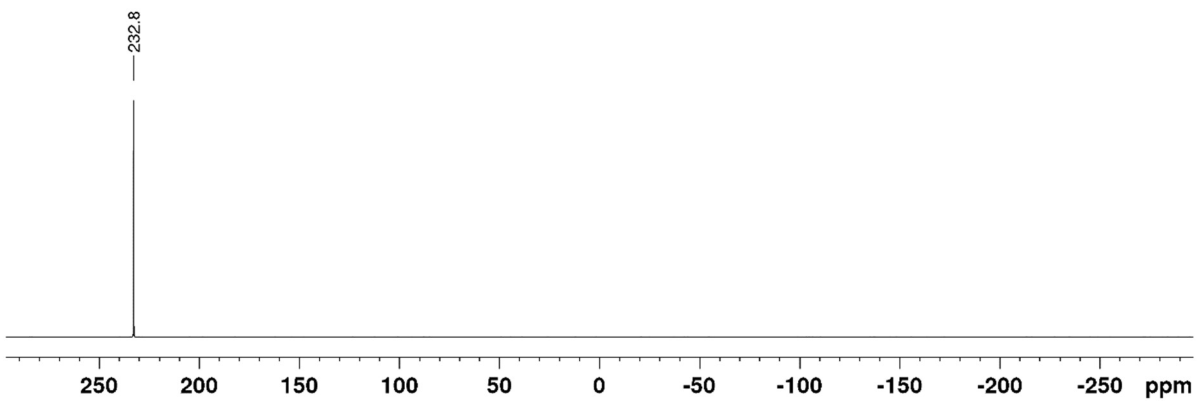

Figure S110. ^{31}P NMR spectrum of **16** (300 K, THF-d_8).

S2.33 Preparation of 1,3-dimethyl-2,2,4,4-tetraphenyl-1,3-diphosphetane 17

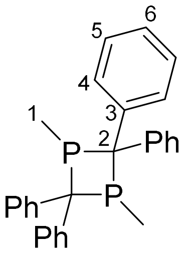

To a yellow solution of **5a**[OTf] (500 mg, 0.95 mmol, 1.0 eq.) in THF (20 mL) a solution of 1 M MeMgBr (950 µl, 0.95 mmol, 1.0 eq.) in ^{n}Bu$_2$O was added dropwise at -78°C. The resulting dark red reaction mixture was allowed to stir at -78°C for 30 min and then warmed to room temperature. Cooling the reaction mixture to -20°C and addition of 2 M HCl in Et$_2$O (500 µl, 1.0 mmol, 1.05 eq.) gave a pale yellow suspension with a colorless precipitate, which was filtered off at room temperature (^{31}P NMR spectrum of an aliquot removed from the filtrate shown in **Figure S111**). After removal of all volatiles *in vacuo* from the filtrate, the solid residue was suspended in toluene (10 ml), filtered and evaporate to dryness *in vacuo*. The remaining solid was then stirred over Et$_2$O (5 ml) for 16 h, filtered, washed with Et$_2$O (3x2 ml) and dried *in vacuo* to obtain the product as a colorless solid.

Note: No additional resonance within the ^{31}P NMR spectrum can be observed upon heating a solution of **17** in toluene-d$_8$ to 80°C.

Yield: 98 mg (49%); **m.p.**: 227-229 °C; **Raman** (100 mW, in cm^{-1}): 3058 (34), 3045 (24), 3017 (8), 2977 (8), 2968 (12), 2903 (28), 1592 (46), 1579 (15), 1489 (5), 1446 (7), 1409 (10), 1283 (5), 1198 (18), 1181 (28), 1167 (51), 1034 (32), 1001 (91), 890 (12), 805 (18), 733 (8), 702 (6), 658 (20), 619 (10), 600 (5), 407 (6), 398 (11), 354 (10), 330 (9), 283 (10), 256 (15), 240 (9), 198 (15), 167 (8), 128 (46), 101 (100), 89 (94); **IR** (ATR, in cm^{-1}): 3074 (vw), 3058 (vw), 3014 (vw), 2992 (vw), 2966 (vw), 2901 (vw), 1590 (w), 1574 (w), 1486 (w), 1439 (w), 1408 (w), 1313 (vw), 1286 (vw), 1244 (vw), 1198 (vw), 1180 (w), 1157 (w), 1111 (vw), 1078 (w), 1031 (w), 999 (vw), 967 (vw), 920 (w), 908 (w), 881 (w), 857 (w), 833 (vw), 802 (m), 754 (w), 729 (m), 696 (vs), 653 (m), 603 (m), 517 (m), 491 (s), 440 (w), 425 (w), 405 (w); **^{1}H NMR** (500.13 MHz, toluene-d$_8$, 300 K, in ppm): δ = 0.49 (6H, d, $^{2}J_{HP}$ = 6.6 Hz, H1), 6.88 (4H, m, H6), 7.07 (8H, m, H5), 7.56 (8H, m, H4); **^{13}C{^{1}H} NMR** (125.76 MHz, toluene-d$_8$, 300 K, in ppm): δ = 11.4 (2C, m, C1), 32.3 (2C, t, $^{1}J_{CP}$ = 8 Hz, C2), 125.4 (4C, m, C6), 128.1 (8C, m, C5), 131.6 (8C, m, C4), 145.1 (4C, m, C3); **^{31}P{^{1}H} NMR** (202.46 MHz, toluene-d$_8$, 300 K, in ppm): δ = 35.9 (2P, s, P).

Note: Although multinuclear NMR analysis of isolated **17** (^{1}H, ^{13}C{^{1}H}, ^{31}P and ^{19}F NMR spectroscopy) shows no evidence of impurities, we were not able to obtain an elemental analysis consistent with calculated values.

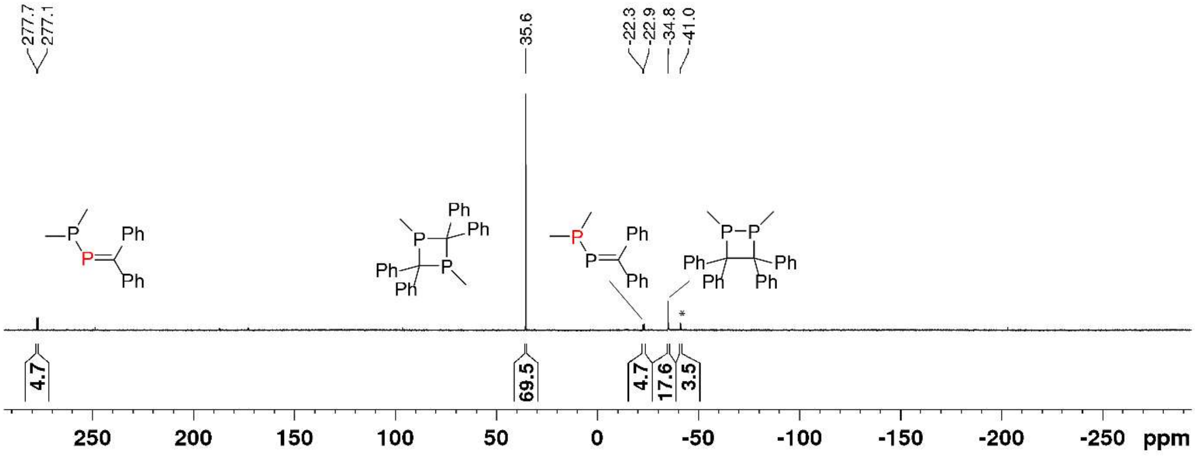

Figure S111. ^{31}P NMR spectrum of an aliquot removed from the filtrate after HCl quenching in the preparation of **17** (300 K, C$_6$D$_6$ cap.).

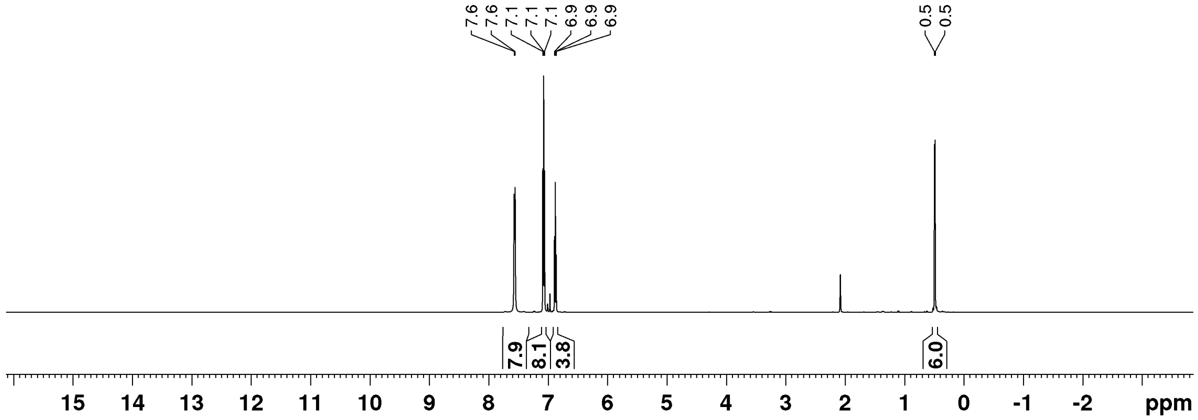

Figure S112. ^{1}H NMR spectrum of **17** (300 K, toluene-d_8).

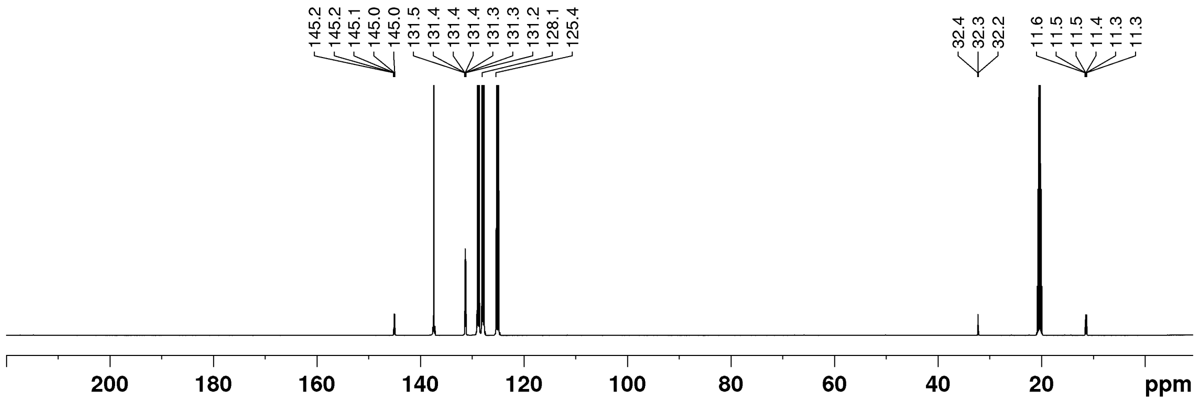

Figure S113. $^{13}C\{^{1}H\}$ NMR spectrum of **17** (300 K, toluene-d_8).

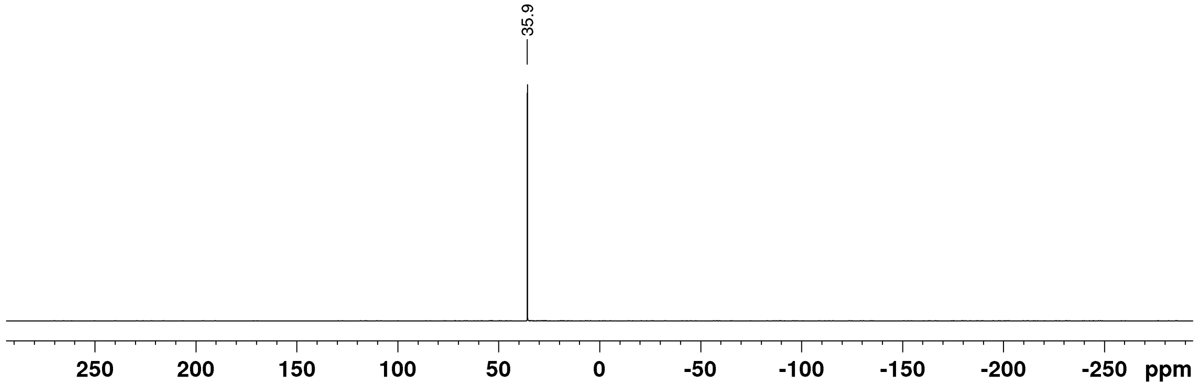

Figure S114. ^{31}P NMR spectrum of **17** (300 K, toluene-d_8).

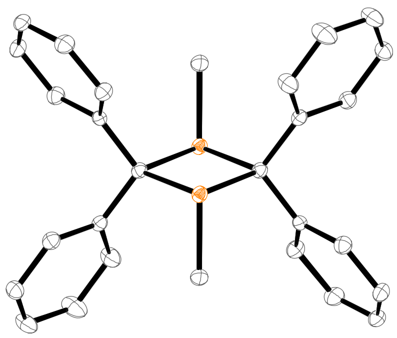

Figure S115. Molecular structure of **17**; hydrogen atoms are omitted for clarity and thermal ellipsoids are displayed at 50% probability.

S2.34 Preparation of $(Ph_2N)P{=}C(Ph)_2$ (18)

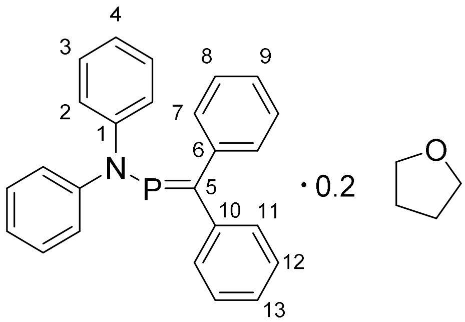

To a yellow solution of **5a**[OTf] (200 mg, 0.38 mmol, 1.0 eq.) in THF (4 mL) a solution of $KNPh_2$ (83 mg, 0.40 mmol, 1.05 eq.) in THF (1 mL) was added dropwise at room temperature. The dark red mixture was stirred at room temperature for 30 min after which Me_3SiOTf (88 mg, 72 µl 0.40 mmol, 1.05 eq.) was added dropwise using a microsyringe. The resulting yellow mixture was evaporated to dryness *in vacuo* and extracted with *n*-hexane (3x5 ml). After removal of all volatiles from the bright yellow extract a crude product (95%, purity as determined by ^1H NMR spectroscopy) was obtained, which was recrystallized from *n*-pentane at -30°C to give the product as yellow crystals.

Yield: 135 mg (crude solid, 95% purity); 125 mg (crystals, 90% yield); **m.p.**: 116-118 °C; **Raman** (100 mW, in cm^{-1}): 3055 (15), 1589 (76), 1485 (32), 1446 (5), 1281 (45), 1218 (100), 1183 (35), 1155 (8), 1041 (21), 1029 (12), 1015 (9), 998 (46), 758 (9), 688 (10), 588 (6), 513 (7), 386 (8), 319 (6), 230 (6), 202 (6), 101 (31), 77 (26); **IR** (ATR, in cm^{-1}): 3051 (w), 3023 (w), 2922 (w), 2853 (w), 1949 (w), 1876 (vw), 1807 (vw), 1585 (m), 1482 (s), 1454 (w), 1443 (m), 1324 (w), 1301 (w), 1268 (s), 1199 (s), 1178 (m), 1154 (w), 1081 (w), 1072 (w), 1027 (m), 995 (w), 948 (s), 917 (w), 905 (w), 878 (w), 830 (w), 751 (vs), 689 (vs), 618 (w), 588 (m), 522 (vs), 446 (w), 431 (m), 411 (m); ^{1}**H NMR** (500.13 MHz, THF-d_8, 300 K, in ppm): δ = 1.63 (0.8H, m, THF), 3.41 (0.8H, m, THF), 6.85 (2H, m, H7), 6.93 (2H, m, H4), 6.99 (4H, m, H2), 7.02 (2H, m, H8), 7.03 (1H, m, H9), 7.09 (4H, m, H3), 7.27 (4H, m, H12), 7.31 (1H, m, H13), 7.34 (2H, m, H11); ^{13}**C{^{1}H} NMR** (125.76 MHz, THF-d_8, 300 K, in ppm): δ = 124.0 (2C, s, C4), 124.7 (4C, d, $^3J_{CP}$ = 5 Hz, C2), 127.6 (1C, s, C9), 127.6 (2C, d, $^3J_{CP}$ = 19 Hz, C11), 128.6 (2C, s, C8), 128.9 (2C, s, C12), 129.1 (1C, d, $^5J_{CP}$ = 3 Hz, C13), 129.4 (4C, s, C3), 130.0 (2C, d, $^3J_{CP}$ = 6 Hz, C7), 142.4 (1C, d, $^2J_{CP}$ = 29 Hz, C10), 142.6 (1C, d, $^2J_{CP}$ = 11 Hz, C6), 148.6 (2C, d, $^2J_{CP}$ = 3 Hz, C1), 180.5 (1C, d, $^1J_{CP}$ = 48 Hz, C5), ^{31}**P{^{1}H} NMR** (202.46 MHz, THF-d_8, 300 K, in ppm): δ = 231.0 (1P, s, P); **elemental analysis**: calcd. for $C_{25}H_{20}NP \cdot 0.2\ C_4H_8O$: C: 81.58, H: 5.73, N: 3.69, S: 0.00; found: C: 81.28, H: 6.060, N: 3.41, S: 0.096.

Note: residual THF from the reaction could not be removed even after drying *in vacuo* for 24 h. Both, NMR data and elemental analysis are consistent with a chemical formula for the solvate of $C_{25}H_{20}NP \cdot 0.2\ C_4H_8O$.

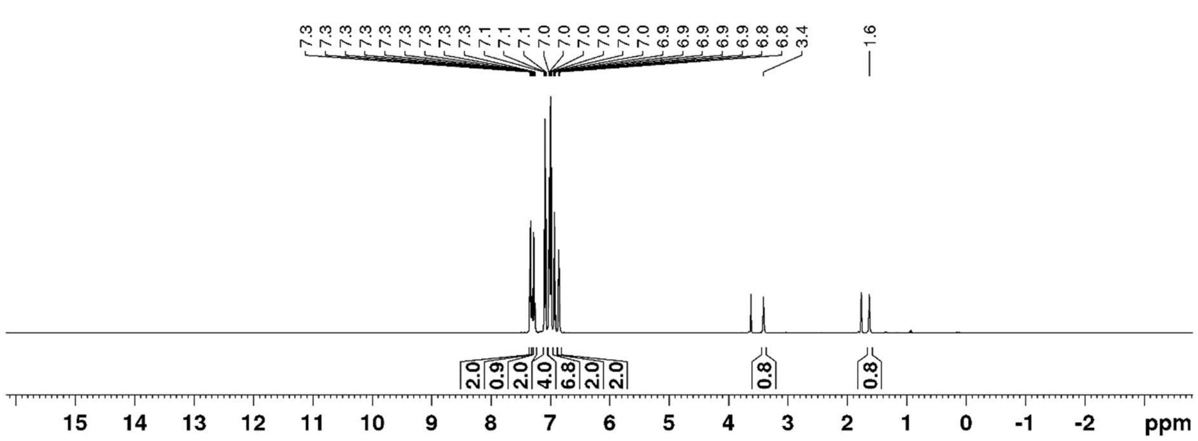

Figure S116. ^1H NMR spectrum of **18** (300 K, THF-d_8).

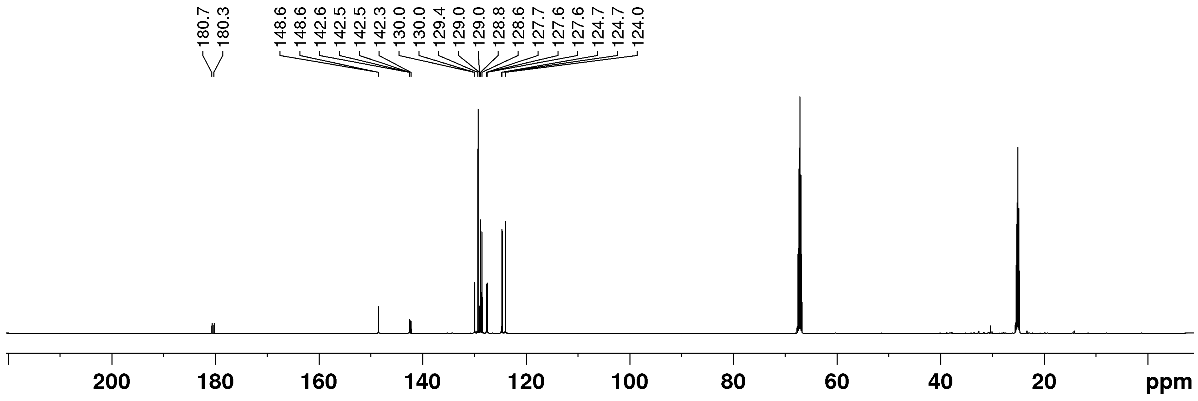

Figure S117. $^{13}C\{^{1}H\}$ NMR spectrum of **18** (300 K, THF-d$_8$).

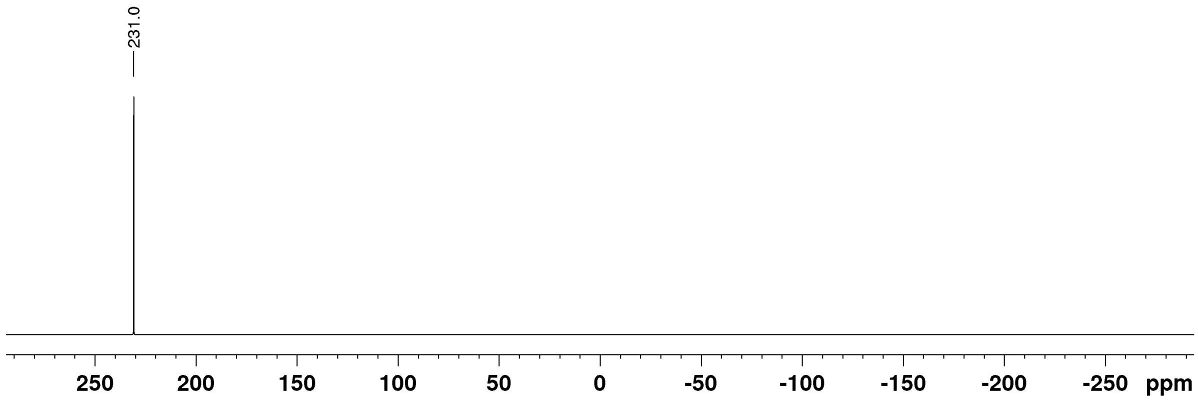

Figure S118. ^{31}P NMR spectrum of **18** (300 K, THF-d$_8$).

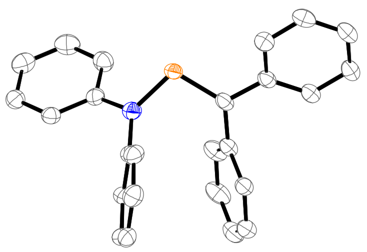

Figure S119. Molecular structure of **18**; hydrogen atoms are omitted for clarity and thermal ellipsoids are displayed at 50% probability.

S2.35 Preparation of $(Ph_2N)P{=}C(NMe_2)_2$ (19)

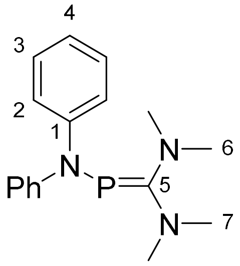

To a yellow solution of **6c**[OTf] (250 mg, 0.54 mmol, 1.0 eq.) in THF (4 mL) a solution of $KNPh_2$ (118 mg, 0.57 mmol, 1.05 eq.) in THF (1 mL) was added dropwise at room temperature. The dark red mixture was stirred at room temperature for 30 min after which Me_3SiOTf (127 mg, 103 µl 0.57 mmol, 1.05 eq.) was added dropwise using a microsyringe. The resulting yellow mixture was evaporated to dryness *in vacuo* and extracted with *n*-hexane (3x5 ml). The volume of the combined extracts was reduced to ~2 ml *in vacuo* and the yellow solution was subsequently stored at -30°C resulting in the formation of analytically pure yellow crystals, which were also suitable for single crystal X-ray diffraction. Removal of the supernatant and drying the crystals *in vacuo* affords the product as a yellow solid.

Yield: 135 mg (80%); **m.p.**: 77-79 °C; **Raman** (100 mW, in cm^{-1}): 3058 (66), 3029 (17), 3006 (20), 2934 (43), 2879 (20), 2845 (22), 2792 (23), 1595 (61), 1585 (81), 1483 (21), 1452 (21), 1406 (9), 1371 (7), 1331 (9), 1290 (7), 1268 (27), 1200 (20), 1179 (20), 1168 (26), 1158 (15), 1133 (10),

1110 (14), 1085 (19), 1056 (9), 1035 (44), 1024 (24), 1003 (63), 992 (100), 975 (10), 940 (12), 897 (9), 878 (11), 851 (6), 768 (14), 708 (12), 684 (15), 637 (38), 609 (37), 591 (29), 538 (11), 521 (7), 501 (7), 452 (29), 414 (7), 398 (10), 380 (14); **IR** (ATR, in cm^{-1}): 3000 (w), 2988 (w), 2923 (m), 2869 (w), 2851 (w), 2790 (w), 1960 (vw), 1932 (vw), 1593 (m), 1580 (s), 1482 (vs), 1447 (m), 1426 (m), 1402 (w), 1369 (s), 1329 (s), 1265 (vs), 1196 (s), 1177 (m), 1156 (m), 1126 (m), 1107 (s), 1084 (vs), 1070 (s), 1054 (s), 1032 (m), 1023 (m), 1004 (w), 991 (m), 973 (w), 958 (w), 934 (s), 893 (m), 876 (s), 838 (m), 764 (w), 749 (vs), 698 (vs), 683 (s), 635 (m), 617 (w), 589 (m), 532 (s), 517 (s), 497 (m), 450 (m); **^{1}H NMR** (400.13 MHz, THF-d_8, 300 K, in ppm): δ = 2.61 (6H, d, $^4J_{HP}$ = 1.0 Hz, H6), 2.85 (6H, d, $^4J_{HP}$ = 3.4 Hz, H7), 6.79 (2H, m, H4), 7.10 (4H, m, H3), 7.28 (4H, m, H2); **^{13}C{^{1}H} NMR** (100.61 MHz, THF-d_8, 300 K, in ppm): δ = 41.3 (2C, d, $^3J_{CP}$ = 18 Hz, C7), 41.3 (2C, s, C6), 121.2 (2C, s, C4), 123.4 (4C, d, $^3J_{CP}$ = 5 Hz, C2), 129.0 (4C, s, C3), 151.1 (2C, d, $^2J_{CP}$ = 2 Hz, C1), 200.8 (1C, d, $^1J_{CP}$ = 90 Hz, C5); **^{31}P{^{1}H} NMR** (161.98 MHz, THF-d_8, 300 K, in ppm): δ = 106.1 (1P, s, P); **elemental analysis**: calcd. for $C_{17}H_{22}N_3P$: C: 68.20, H: 7.41, N: 14.0, S: 0.00; found: C: 68.46, H: 7.308, N: 13.56, S: 0.054.

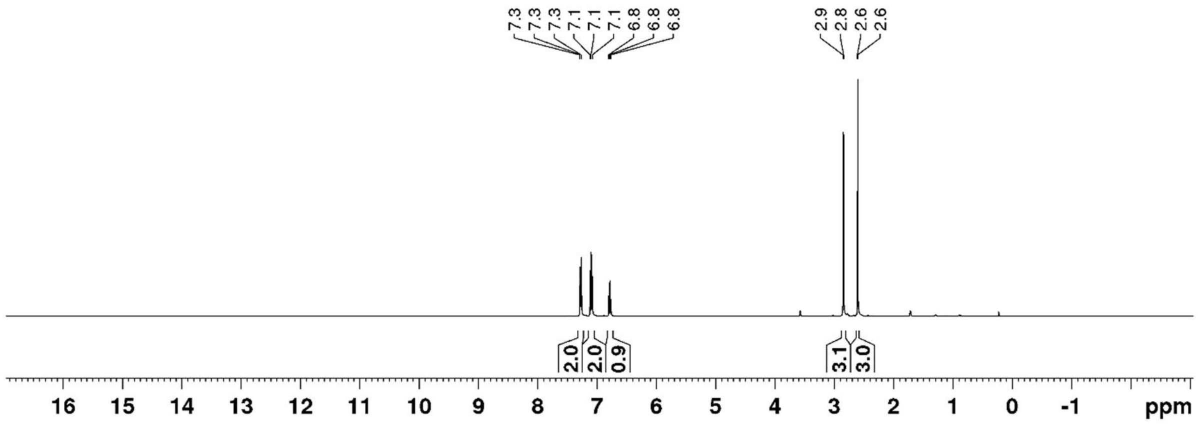

Figure S120. ^{1}H NMR spectrum of **19** (300 K, THF-d_8).

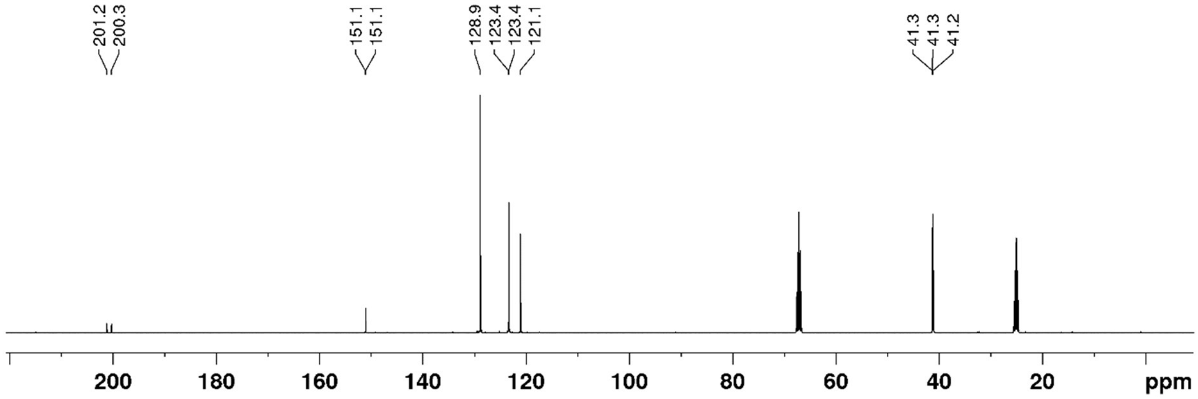

Figure S121. ^{13}C{^{1}H} NMR spectrum of **19** (300 K, THF-d_8).

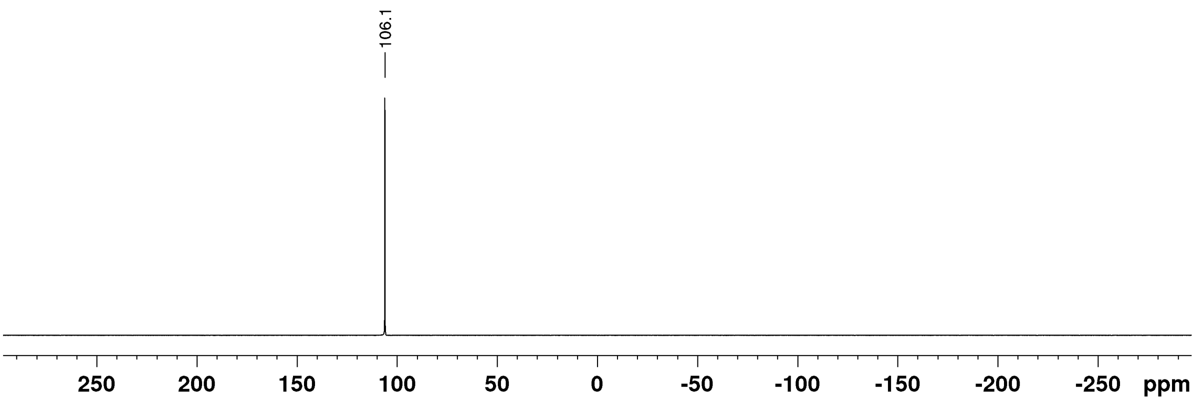

Figure S122. ^{31}P NMR spectrum of **19** (300 K, THF-d_8).

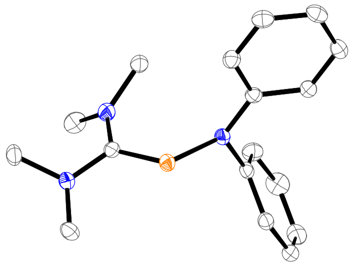

Figure S123. Molecular structure of **19**; hydrogen atoms are omitted for clarity and thermal ellipsoids are displayed at 50% probability.

S3 Single Crystal X-ray Diffraction Data

S3.1 General remarks

Suitable single crystals were coated with Paratone-N oil, mounted using a nylon loop and frozen in a cold nitrogen stream. Crystals were measured at 100 K on a Rigaku Oxford Diffraction SuperNova system using Cu K_{α} radiation (λ = 1.54184 Å) generated by a Nova micro-focus X-ray source. Reflections were collected with an Atlas S2 detector. Data reduction and absorption correction was performed with CrysaAlisPro[19] software. Using Olex2[20], the structures were solved with SHELXS/T[21] by direct methods and refined with SHELXL[22] by least-square minimization against F^2 using first isotropic and later anisotropic thermal parameters for all non-hydrogen atoms. Hydrogen atoms bonded to carbon atoms were added to the structure models on calculated positions using the riding model. All other hydrogen atoms were localized in the difference Fourier map. Images of the structures were produced with Olex2[20] software. All structures have been deposited with the Cambridge Crystallographic Data Centre (CCDC) and can be accessed free of charge under the numbers CCDC 2220645-2220663 and 2243526-2243528.

S3.2 Refinement details

Table S1. Crystallographic data of **1b-d**[OTf].

	**1b**[OTf]	**1c**[OTf]	**1d**[OTf]
Empirical formula	$C_{15}H_{29}F_3N_2O_3P_2S$	$C_{18}H_{35}F_3N_2O_3P_2S$	$C_{30}H_{53}F_3N_2O_3P_2S$
Formula weight	436.40	478.48	640.74
Temperature/K	100.01(10)	104.3(7)	100.00(10)
Crystal system	monoclinic	monoclinic	monoclinic
Space group	$P2_1/c$	$P2_1/n$	C2/c
a/Å	8.33228(12)	13.27022(14)	18.38970(10)
b/Å	27.5583(3)	14.08162(11)	15.07000(10)
c/Å	9.66095(16)	13.28589(13)	24.1300(2)
α/°	90	90	90
β/°	104.9924(15)	108.0219(11)	96.8550(10)
γ/°	90	90	90
Volume/Å^3	2142.87(5)	2360.88(4)	6639.41(8)
Z	4	4	8
ρ_{calc}g/cm^3	1.353	1.346	1.282
μ/mm^{-1}	3.145	2.901	2.195
F(000)	920.0	1016.0	2752.0
Crystal size/mm^3	0.215 × 0.062 × 0.056	0.132 × 0.105 × 0.015	0.733 × 0.192 × 0.143
Radiation	CuKα (λ = 1.54184)	CuKα (λ = 1.54184)	CuKα (λ = 1.54184)
2Θ range for data collection/°	6.414 to 153.422	8.23 to 153.542	7.38 to 153.368
Index ranges	-10 ≤ h ≤ 9, -34 ≤ k ≤ 24, -11 ≤ l ≤ 12	-16 ≤ h ≤ 16, -17 ≤ k ≤ 11, -16 ≤ l ≤ 16	-18 ≤ h ≤ 22, -18 ≤ k ≤ 18, -30 ≤ l ≤ 30
Reflections collected	19445	23382	32046
Independent reflections	4486 [R_{int} = 0.0257, R_{sigma} = 0.0201]	4946 [R_{int} = 0.0193, R_{sigma} = 0.0144]	6916 [R_{int} = 0.0212, R_{sigma} = 0.0175]
Data/restraints/parameters	4486/0/244	4946/0/271	6916/0/376
Goodness-of-fit on F^2	1.044	1.071	1.062
Final R indexes [I>=2σ (I)]	R_1 = 0.0291, wR_2 = 0.0755	R_1 = 0.0264, wR_2 = 0.0689	R_1 = 0.0301, wR_2 = 0.0760
Final R indexes [all data]	R_1 = 0.0309, wR_2 = 0.0768	R_1 = 0.0272, wR_2 = 0.0696	R_1 = 0.0305, wR_2 = 0.0763
Largest diff. peak/hole/e Å^{-3}	0.36/-0.45	0.32/-0.34	0.34/-0.37
CCDC	2220645	2220646	2220647

Table S2. Crystallographic data of **1e**[OTf] and **5a,b**[OTf].

	**1e**[OTf]·CH_3CN	**5a**[OTf]	**5b**[OTf]
Empirical formula	$C_{39}H_{45}F_3N_3O_3P_3S$	$C_{25}H_{30}F_3N_2O_3PS$	$C_{25}H_{28}Cl_2F_3N_2O_3PS$
Formula weight	785.75	526.54	595.42
Temperature/K	100.01(10)	100.00(10)	99.95(13)
Crystal system	monoclinic	monoclinic	orthorhombic
Space group	$P2_1/c$	$P2_1$	$Pna2_1$
a/Å	15.9741(2)	7.91250(10)	22.5395(3)
b/Å	9.48620(10)	21.2964(2)	15.9815(2)
c/Å	26.2844(3)	15.05710(10)	7.59410(10)
α/°	90	90	90
β/°	90.6500(10)	95.5400(10)	90
γ/°	90	90	90
Volume/$Å^3$	3982.71(8)	2525.39(4)	2735.51(6)
Z	4	4	4
ρ_{calc}g/cm^3	1.310	1.385	1.446
μ/mm^{-1}	2.317	2.197	3.853
F(000)	1648.0	1104.0	1232.0
Crystal size/mm^3	0.189 × 0.087 × 0.043	0.268 × 0.146 × 0.053	0.117 × 0.075 × 0.059
Radiation	CuKα (λ = 1.54184)	Cu Kα (λ = 1.54184)	Cu Kα (λ = 1.54184)
2Θ range for data collection/°	5.532 to 153.776	5.898 to 153.67	6.78 to 153.66
Index ranges	-20 ≤ h ≤ 20, -11 ≤ k ≤ 11, -33 ≤ l ≤ 32	-9 ≤ h ≤ 9, -26 ≤ k ≤ 26, -15 ≤ l ≤ 18	-26 ≤ h ≤ 28, -17 ≤ k ≤ 20, -9 ≤ l ≤ 9
Reflections collected	47503	29760	32109
Independent reflections	8332 [R_{int} = 0.0337, R_{sigma} = 0.0232]	10304 [R_{int} = 0.0412, R_{sigma} = 0.0470]	5651 [R_{int} = 0.0502, R_{sigma} = 0.0301]
Data/restraints/parameters	8332/0/476	10304/1/644	5651/1/340
Goodness-of-fit on F^2	1.046	1.039	1.072
Final R indexes [I>=2σ (I)]	R_1 = 0.0342, wR_2 = 0.0861	R_1 = 0.0371, wR_2 = 0.0910	R_1 = 0.0481, wR_2 = 0.1301
Final R indexes [all data]	R_1 = 0.0382, wR_2 = 0.0891	R_1 = 0.0415, wR_2 = 0.0945	R_1 = 0.0488, wR_2 = 0.1311
Largest diff. peak/hole/e $Å^{-3}$	0.34/-0.45	0.28/-0.34	0.45/-0.37
Flack parameter		0.494(16)	-0.008(14)
CCDC	2220648	2220649	2220650

Table S3. Crystallographic data of **5c-e**[OTf].

	**5c**[OTf] · $2C_6H_5F$	**5d**[OTf]	**5e**[OTf]
Empirical formula	$C_{41}H_{50}F_5N_4O_3PS$	$C_{23}H_{28}F_3N_2O_3PS_2$	$C_{22}H_{36}F_3N_2O_3PS$
Formula weight	804.88	532.56	496.56
Temperature/K	100.01(10)	100.01(10)	100.00(10)
Crystal system	monoclinic	monoclinic	orthorhombic
Space group	$P2_1/c$	$P2_1/n$	$P2_12_12_1$
a/Å	14.4167(2)	7.85770(10)	10.6947(5)
b/Å	29.5643(3)	21.2672(3)	10.7725(6)
c/Å	9.57180(10)	14.9565(2)	21.9056(12)
α/°	90	90	90
β/°	94.9260(10)	94.7180(10)	90
γ/°	90	90	90
Volume/Å^3	4064.62(8)	2490.93(6)	2523.7(2)
Z	4	4	4
ρ_{calc}g/cm^3	1.315	1.420	1.307
μ/mm^{-1}	1.640	2.996	2.156
F(000)	1696.0	1112.0	1056.0
Crystal size/mm^3	0.141 × 0.101 × 0.089	0.267 × 0.072 × 0.048	0.345 × 0.128 × 0.047
Radiation	Cu Kα (λ = 1.54184)	Cu Kα (λ = 1.54184)	Cu Kα (λ = 1.54184)
2Θ range for data collection/°	5.978 to 149	7.242 to 136.484	8.072 to 136.458
Index ranges	-17 ≤ h ≤ 18, -30 ≤ k ≤ 36, -11 ≤ l ≤ 11	-9 ≤ h ≤ 9, -24 ≤ k ≤ 25, -18 ≤ l ≤ 16	-8 ≤ h ≤ 12, -12 ≤ k ≤ 12, -26 ≤ l ≤ 25
Reflections collected	27264	26810	20676
Independent reflections	8282 [R_{int} = 0.0470, R_{sigma} = 0.0420]	4560 [R_{int} = 0.0419, R_{sigma} = 0.0259]	4604 [R_{int} = 0.0655, R_{sigma} = 0.0503]
Data/restraints/parameters	8282/396/576	4560/1253/529	4604/438/431
Goodness-of-fit on F^2	1.038	1.195	1.031
Final R indexes [I>=2σ (I)]	R_1 = 0.0560, wR_2 = 0.1524	R_1 = 0.0668, wR_2 = 0.1455	R_1 = 0.0396, wR_2 = 0.0934
Final R indexes [all data]	R_1 = 0.0651, wR_2 = 0.1642	R_1 = 0.0701, wR_2 = 0.1473	R_1 = 0.0513, wR_2 = 0.1005
Largest diff. peak/hole/e Å^{-3}	0.90/-0.40	1.00/-0.49	0.22/-0.27
Flack parameter			-0.03(2)
CCDC	2220651	2220652	2220653

Table S4. Crystallographic data of **5f**[OTf] and **6a,b**[OTf].

	**5f**[OTf]	**6a**[OTf]	**6b**[OTf]
Empirical formula	$C_{20}H_{28}F_3N_2O_4PS$	$C_{15}H_{27}F_3N_3O_3PS$	$C_{21}H_{31}F_3N_3O_3PS$
Formula weight	480.47	417.42	493.52
Temperature/K	100.01(10)	99.99(10)	100.01(10)
Crystal system	triclinic	monoclinic	monoclinic
Space group	P-1	$P2_1/c$	Cc
a/Å	9.1520(4)	8.27030(10)	8.5928(2)
b/Å	10.6177(4)	15.8553(3)	16.3219(3)
c/Å	12.1696(5)	15.9960(3)	17.1998(4)
α/°	98.302(3)	90	90
β/°	93.334(4)	104.553(2)	93.076(2)
γ/°	95.037(4)	90	90
Volume/$Å^3$	1162.63(8)	2030.23(6)	2408.81(9)
Z	2	4	4
$\rho_{calc}g/cm^3$	1.372	1.366	1.361
μ/mm^{-1}	2.361	2.589	2.272
F(000)	504.0	880.0	1040.0
Crystal size/mm^3	0.211 × 0.196 × 0.14	0.304 × 0.203 × 0.18	0.136 × 0.088 × 0.037
Radiation	Cu Kα (λ = 1.54184)	Cu Kα (λ = 1.54184)	Cu Kα (λ = 1.54184)
2Θ range for data collection/°	7.36 to 136.476	7.982 to 154.232	10.302 to 153.35
Index ranges	-5 ≤ h ≤ 11, -12 ≤ k ≤ 12, -14 ≤ l ≤ 14	-10 ≤ h ≤ 10, -19 ≤ k ≤ 19, -15 ≤ l ≤ 19	-10 ≤ h ≤ 10, -20 ≤ k ≤ 20, -20 ≤ l ≤ 21
Reflections collected	11457	22934	8635
Independent reflections	4246 [R_{int} = 0.0237, R_{sigma} = 0.0277]	4242 [R_{int} = 0.0364, R_{sigma} = 0.0239]	8635 [R_{int} = ?, R_{sigma} = 0.0257]
Data/restraints/parameters	4246/42/307	4242/0/243	8635/2/298
Goodness-of-fit on F^2	1.037	1.038	1.079
Final R indexes [I>=2σ (I)]	R_1 = 0.0432, wR_2 = 0.1119	R_1 = 0.0326, wR_2 = 0.0860	R_1 = 0.0356, wR_2 = 0.0973
Final R indexes [all data]	R_1 = 0.0452, wR_2 = 0.1137	R_1 = 0.0340, wR_2 = 0.0874	R_1 = 0.0373, wR_2 = 0.0981
Largest diff. peak/hole/e $Å^{-3}$	0.73/-0.44	0.38/-0.36	0.25/-0.21
Flack parameter			0.495(11)
CCDC	2220654	2220655	2220656

Table S5. Crystallographic data of **6c**[OTf] and **11a,b**[OTf].

	**6c**[OTf]	**11a**[OTf]	**11b**[OTf] · *o*-$C_6H_4F_2$
Empirical formula	$C_{17}H_{32}F_3N_4O_3PS$	$C_{34}H_{41}F_3N_2O_3P_2S$	$C_{37}H_{39}F_5N_2O_3P_2S$
Formula weight	460.49	676.69	748.70
Temperature/K	100.01(10)	99.98(17)	100.00(10)
Crystal system	monoclinic	triclinic	monoclinic
Space group	$P2_1/c$	P-1	$P2_1$
a/Å	10.95670(10)	10.4527(4)	9.4289(4)
b/Å	8.79670(10)	10.6996(4)	14.2320(5)
c/Å	23.2287(2)	17.3037(6)	13.9072(4)
α/°	90	97.938(3)	90
β/°	91.6850(10)	97.919(3)	104.312(3)
γ/°	90	116.521(4)	90
Volume/$Å^3$	2237.88(4)	1671.01(12)	1808.32(11)
Z	4	2	2
ρ_{calc}g/cm^3	1.367	1.345	1.375
μ/mm^{-1}	2.413	2.222	2.193
F(000)	976.0	712.0	780.0
Crystal size/mm^3	0.24 × 0.094 × 0.084	0.653 × 0.297 × 0.1	0.246 × 0.14 × 0.055
Radiation	Cu Kα (λ = 1.54184)	Cu Kα (λ = 1.54184)	Cu Kα (λ = 1.54184)
2Θ range for data collection/°	7.616 to 153.506	5.292 to 152.968	6.56 to 136.45
Index ranges	-13 ≤ h ≤ 13, -10 ≤ k ≤ 10, -28 ≤ l ≤ 29	-12 ≤ h ≤ 13, -13 ≤ k ≤ 12, -21 ≤ l ≤ 21	-11 ≤ h ≤ 11, -17 ≤ k ≤ 14, -16 ≤ l ≤ 16
Reflections collected	22039	15881	16114
Independent reflections	4666 [R_{int} = 0.0241, R_{sigma} = 0.0185]	6927 [R_{int} = 0.0376, R_{sigma} = 0.0431]	5293 [R_{int} = 0.0520, R_{sigma} = 0.0604]
Data/restraints/parameters	4666/36/300	6927/0/415	5293/1/457
Goodness-of-fit on F^2	1.055	1.040	1.032
Final R indexes [I>=2σ (I)]	R_1 = 0.0301, wR_2 = 0.0807	R_1 = 0.0485, wR_2 = 0.1275	R_1 = 0.0466, wR_2 = 0.1131
Final R indexes [all data]	R_1 = 0.0318, wR_2 = 0.0823	R_1 = 0.0549, wR_2 = 0.1321	R_1 = 0.0514, wR_2 = 0.1166
Largest diff. peak/hole/e Å^{-3}	0.54/-0.38	0.71/-0.44	0.51/-0.41
Flack parameter			0.01(2)
CCDC	2220657	2220658	2220659

Table S6. Crystallographic data of **12a,b**[OTf] and **13**[OTf].

	**12a**[OTf]	**12b**[OTf]	**13**[OTf]
Empirical formula	$C_{61}H_{60}F_3N_2O_3P_3PdS$	$C_{61}H_{60}F_3N_2O_3P_3PtS$	$C_{29}H_{30}F_3FeN_2O_7PS$
Formula weight	1157.48	1246.17	694.43
Temperature/K	100.02(10)	100.01(10)	100.01(10)
Crystal system	monoclinic	monoclinic	orthorhombic
Space group	$P2_1$	C2/c	$Pna2_1$
a/Å	17.36850(10)	35.4994(2)	17.50191(7)
b/Å	34.4641(2)	13.44480(10)	10.56688(3)
c/Å	18.28810(10)	23.60750(10)	16.61932(7)
α/°	90	90	90
β/°	94.78	99.6580(10)	90
γ/°	90	90	90
Volume/Å^3	10909.00(11)	11107.74(12)	3073.59(2)
Z	8	8	4
ρ_{calc}g/cm^3	1.410	1.490	1.501
μ/mm^{-1}	4.406	6.358	5.654
F(000)	4784.0	5040.0	1432.0
Crystal size/mm^3	0.166 × 0.132 × 0.075	0.152 × 0.085 × 0.04	0.29 × 0.178 × 0.13
Radiation	Cu Kα (λ = 1.54184)	Cu Kα (λ = 1.54184)	Cu Kα (λ = 1.54184)
2Θ range for data collection/°	4.848 to 153.598	5.05 to 153.404	9.778 to 153.372
Index ranges	-21 ≤ h ≤ 21, -43 ≤ k ≤ 41, -23 ≤ l ≤ 18	-43 ≤ h ≤ 21, -16 ≤ k ≤ 16, -29 ≤ l ≤ 29	-21 ≤ h ≤ 21, -13 ≤ k ≤ 10, -20 ≤ l ≤ 20
Reflections collected	140581	44323	35901
Independent reflections	43921 [R_{int} = 0.0443, R_{sigma} = 0.0480]	11445 [R_{int} = 0.0243, R_{sigma} = 0.0206]	6149 [R_{int} = 0.0267, R_{sigma} = 0.0183]
Data/restraints/parameters	43921/259/2763	11445/0/673	6149/1/403
Goodness-of-fit on F^2	1.025	1.024	1.033
Final R indexes [I>=2σ (I)]	R_1 = 0.0346, wR_2 = 0.0823	R_1 = 0.0196, wR_2 = 0.0466	R_1 = 0.0191, wR_2 = 0.0492
Final R indexes [all data]	R_1 = 0.0365, wR_2 = 0.0834	R_1 = 0.0222, wR_2 = 0.0479	R_1 = 0.0193, wR_2 = 0.0494
Largest diff. peak/hole/e Å^{-3}	1.14/-0.50	0.33/-0.75	0.17/-0.27
Flack parameter	0.437(3)		-0.0087(12)
CCDC	2220660	2220661	2220662

Table S7. Crystallographic data of **15**[OTf], **17**, **18**.

	**15**[OTf] · $0.5C_6H_4F_2{\cdot}C_5H_{12}$	**17**	**18**
Empirical formula	$C_{34}H_{32.5}Cl_4F_{3.5}N_2O_5PS$	$C_{28}H_{26}P_2$	$C_{25}H_{20}NP$
Formula weight	820.45	424.43	365.39
Temperature/K	100.00(10)	100.00(10)	100.01(10)
Crystal system	triclinic	triclinic	monoclinic
Space group	P-1	P-1	P21/n
a/Å	8.8478(2)	7.7279(3)	12.7425(5)
b/Å	19.0050(4)	8.7761(4)	9.7245(3)
c/Å	24.8005(5)	9.3032(4)	16.0159(5)
α/°	67.922(2)	68.160(4)	90
β/°	86.234(2)	69.116(4)	103.956(3)
γ/°	77.930(2)	83.180(3)	90
Volume/Å^3	3778.66(15)	547.12(5)	1926.02(12)
Z	4	1	4
ρ_{calc}g/cm^3	1.442	1.288	1.26
μ/mm^{-1}	4.287	1.882	1.311
F(000)	1684.0	224	768
Crystal size/mm^3	0.196 × 0.132 × 0.054	0.318 × 0.168 × 0.068	0.197 × 0.149 × 0.033
Radiation	Cu Kα (λ = 1.54184)	Cu Kα (λ = 1.54184)	Cu Kα (λ = 1.54184)
2Θ range for data collection/°	5.12 to 153.606	10.862 to 153.462	7.99 to 153.586
Index ranges	-11 ≤ h ≤ 10, -23 ≤ k ≤ 19, -31 ≤ l ≤ 31	-9 ≤ h ≤ 9, -10 ≤ k ≤ 10, -8 ≤ l ≤ 11	-15 ≤ h ≤ 15, -12 ≤ k ≤ 12, -19 ≤ l ≤ 19
Reflections collected	42821	5149	4225
Independent reflections	15510 [R_{int} = 0.0489, R_{sigma} = 0.0572]	2266 [R_{int} = 0.0186, R_{sigma} = 0.0226]	4225 [Rint = ?, Rsigma = 0.0358]
Data/restraints/parameters	15510/84/974	2266/0/137	4225/0/255
Goodness-of-fit on F^2	1.044	1.044	1.046
Final R indexes [I>=2σ (I)]	R_1 = 0.0510, wR_2 = 0.1285	R_1 = 0.0291, wR_2 = 0.0744	R1 = 0.0483, wR2 = 0.1206
Final R indexes [all data]	R_1 = 0.0640, wR_2 = 0.1361	R_1 = 0.0303, wR_2 = 0.0754	R1 = 0.0603, wR2 = 0.1255
Largest diff. peak/hole/e Å^{-3}	0.67/-0.39	0.29/-0.24	0.23/-0.32
CCDC	2220663	2243526	2243527

Table S8. Crystallographic data of **19**.

	**19**
Empirical formula	$C_{17}H_{22}N_3P$
Formula weight	299.34
Temperature/K	100.01(10)
Crystal system	monoclinic
Space group	P21/n
a/Å	11.76800(10)
b/Å	9.49050(10)
c/Å	15.2118(2)
α/°	90
β/°	108.1480(10)
γ/°	90
Volume/Å^3	1614.40(3)
Z	4
ρ_{calc}g/cm^3	1.232
μ/mm^{-1}	1.472
F(000)	640
Crystal size/mm^3	0.404 × 0.285 × 0.266
Radiation	Cu Kα (λ = 1.54184)
2Θ range for data collection/°	8.354 to 152.974
Index ranges	-14 ≤ h ≤ 14, -10 ≤ k ≤ 11, -17 ≤ l ≤ 18
Reflections collected	16399
Independent reflections	3366 [Rint = 0.0208, Rsigma = 0.0137]
Data/restraints/parameters	3366/0/194
Goodness-of-fit on F^2	1.026
Final R indexes [I>=2σ (I)]	R1 = 0.0314, wR2 = 0.0780
Final R indexes [all data]	R1 = 0.0321, wR2 = 0.0785
Largest diff. peak/hole/e Å^{-3}	0.25/-0.32
CCDC	2243528

In the structure refinements of **5c**[OTf]·2C_6H_5F, **5d**[OTf] and **5e**[OTf] SADI, SIMU, RIGU and/or FLAT restraints were applied to the disordered C_6H_5F solvent molecule, the substituents at the *C*-terminus and the (L_C)P-substituent, respectively. Occurring disorders in the structure refinement of **5f**[OTf], **6c**[OTf], **12a**[OTf], **18** and **23** could be refined without restraints or constraints. **5a**[OTf] and **6b**[OTf] were refined as inversion twins in the ratio 50:50. **18** was refined as a twin using the twin law Twin Law (1 0 1) with a BASF value of 0.18. In the refinement of the structure of **15**[OTf] the PLATON/SQUEEZE[23] extension in Olex2 was used for a solvent mask of *n*-pentane.

S4. Computational data

The geometries and energies of all systems included in this study were fully optimized at the RI-BP86-D3/def2-TZVP level of theory. The calculations have been performed by using the program TURBOMOLE version 7.0.[24] For the calculations we have used the BP86[25] functional with the D3 correction for dispersion.[26] In order to reproduce solvent effects, we have used the conductor-like screening model COSMO,[27] which is a variant of the dielectric continuum solvation models. The minimum nature of the complexes and compounds have been confirmed by doing frequency calculations. The transition states were initially located using the NEB tool of ORCA 4.2[28] and re-optimized using Turbomole 7.0.[24] They only present one negative frequency that corresponds to the movement of atoms connecting the intermediates. The NMR calculations were computed using the GIAO method[29] at the B3LYP-RIJK/def2-TZVPP/def2-JK level of theory using the ORCA 4.2[28] calculation package. We have used acetonitrile as solvent using CPCM continuum model.[30]

Table S9: Frontier orbital energy levels of 1a-c$^+$ and DmpP–PMe$_3$ at the RI-BP86-D3/def2-TZVP level of theory

	HOMO (eV)	LUMO (eV)	LUMO+1(eV)	GAP(HOMO-LUMO)
**1a$^+$**	-7.453	-4.981	-4.790	2.472
**1b$^+$**	-7.787	-4.996	-3.953	2.791
**1c$^+$**	-7.654	-4.894	-3.741	2.760
DmpP–PMe$_3$	-3.852	-1.409	-1.239	2.443

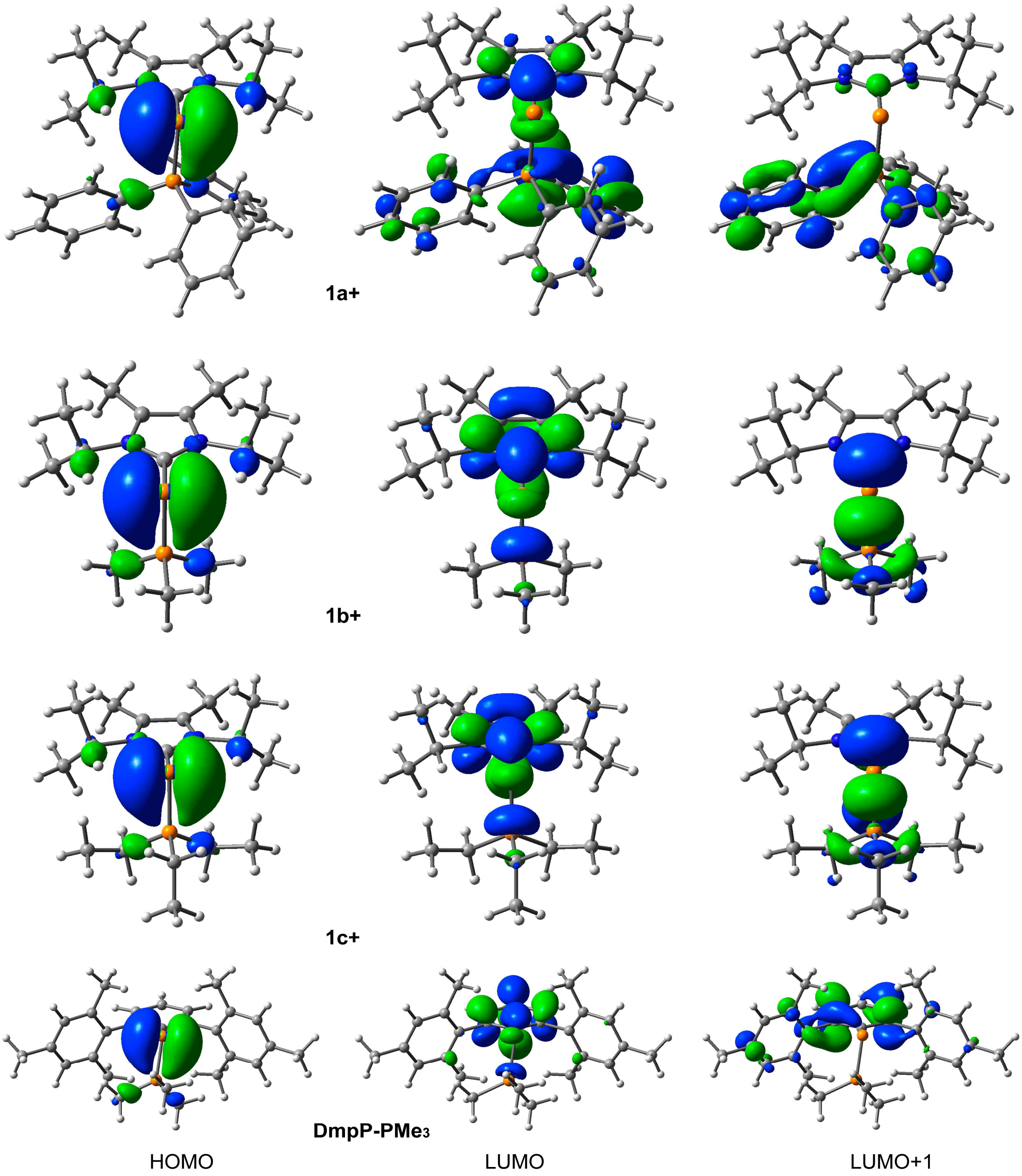

Figure S124. HOMOs, LUMOs and LUMO+1s of compounds **1a-c**$^+$ and DmpP-PMe$_3$ using the 0.045 a.u. isosurface.

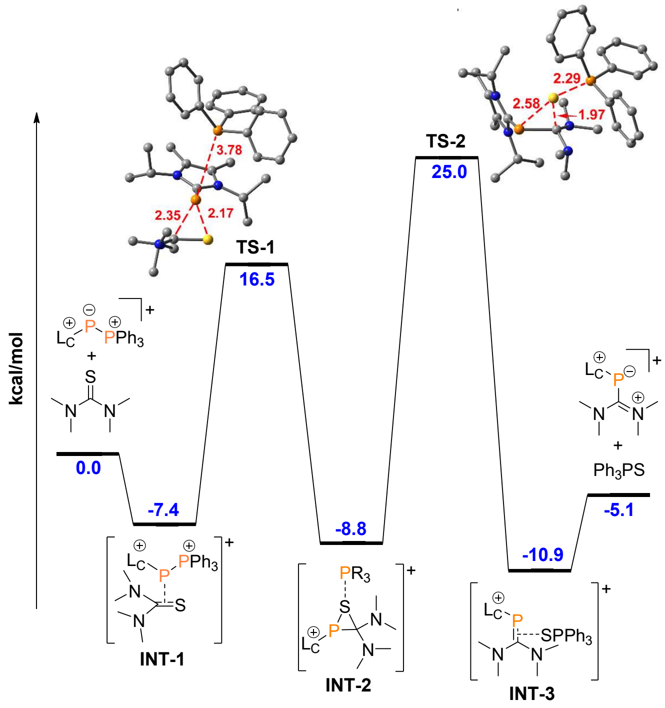

Figure S125. Left: Reaction profile for the conversion of **1a**+ to **6c**+ at the RI-BP86-D3/def2-TZVP (acetonitrile) level of theory; Optimized geometries of the transition states (TS) with distances in Å.

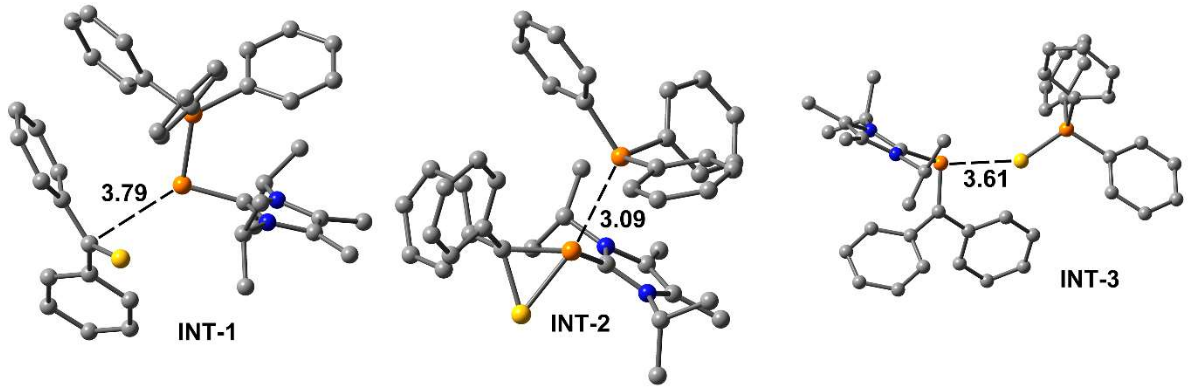

Figure S126. The three intermediates corresponding to the reaction profile for the conversion of **1a**+ to **5a**+. H-atoms omitted for clarity. Distances in Å.

Cartesian coordinates

[LcP-PPh3]+ (1a+)

P	0.3131998	-0.2612934	0.5438602
P	-1.3403982	-1.6052127	0.2968079
C	-0.6385209	-3.2570848	0.0105385
C	0.5104336	-3.3954468	-0.7872187
C	-1.2673829	-4.3970730	0.5373490
C	1.0223233	-4.6659492	-1.0531560
H	1.0002550	-2.5080655	-1.1906551
C	-0.7486302	-5.6632429	0.2646392
H	-2.1538477	-4.2968487	1.1638156
C	0.3939072	-5.7990149	-0.5291577
H	1.9167110	-4.7698092	-1.6678992
H	-1.2368589	-6.5462636	0.6777995
H	0.7977234	-6.7904970	-0.7363052
C	-2.4438945	-1.7811668	1.7464712
C	-3.8294686	-1.9703258	1.6250521
C	-1.8519523	-1.7743416	3.0197220
C	-4.6143488	-2.1257620	2.7708522
H	-4.2986881	-1.9989017	0.6415774

C	-2.6386042	-1.9422560	4.1584073
H	-0.7729804	-1.6304923	3.1127001
C	-4.0222780	-2.1089314	4.0357152
H	-5.6911659	-2.2661406	2.6716003
H	-2.1717529	-1.9414021	5.1440181
H	-4.6378129	-2.2313758	4.9273635
C	-2.4395545	-1.2053743	-1.1040726
C	-3.3149574	-0.1076956	-1.0177417
C	-2.4032500	-1.9690948	-2.2803442
C	-4.1494393	0.2096392	-2.0890926
H	-3.3591909	0.4901204	-0.1075260
C	-3.2423697	-1.6461416	-3.3496905
H	-1.7311758	-2.8242084	-2.3572361
C	-4.1182117	-0.5614874	-3.2558906
H	-4.8318235	1.0563361	-2.0093420
H	-3.2152035	-2.2508433	-4.2564634
H	-4.7760926	-0.3169230	-4.0902585
C	-0.6613505	1.2339943	0.1382709
C	-1.4789251	3.0042089	-1.0019032
C	-1.9405721	3.0904023	0.2958418
N	-0.6990339	1.8587474	-1.0781600
N	-1.4201247	1.9996118	0.9816516
C	-2.8563035	4.1112631	0.8801356
H	-2.9315335	4.9728350	0.2073916
H	-3.8711876	3.7123594	1.0203245
H	-2.5037931	4.4788898	1.8507067
C	-1.7588232	3.9239705	-2.1403937
H	-2.1932251	4.8576324	-1.7665277
H	-0.8478080	4.1815422	-2.6947847
H	-2.4728868	3.4839573	-2.8510942
C	0.0722907	1.3621051	-2.2519622
H	0.3574794	0.3484431	-1.9316249
C	1.3482669	2.1842366	-2.4319332
H	1.1327712	3.2304981	-2.6878508
H	1.9568719	2.1667396	-1.5182462
H	1.9436083	1.7562480	-3.2496439
C	-0.7700625	1.2438006	-3.5198496
H	-1.7464160	0.7907903	-3.3102789
H	-0.9226218	2.2061885	-4.0224533
H	-0.2401303	0.5893367	-4.2243496
C	-1.5479911	1.6992087	2.4337545
H	-1.0544263	0.7200531	2.5165049
C	-3.0006591	1.5403922	2.8776012
H	-3.5524327	0.8680970	2.2073228
H	-3.0114573	1.0796644	3.8736465
H	-3.5324408	2.4967308	2.9460398
C	-0.7456599	2.6971834	3.2688967
H	0.2977829	2.7363375	2.9300253
H	-1.1643279	3.7109283	3.2322152
H	-0.7532543	2.3731010	4.3179309

$[L_CP\text{-}PMe_3]^+$ ($1b^+$)

P	0.1951884	-0.2363144	0.5021892
P	-1.4181735	-1.5819465	0.1712308
C	-0.7542451	1.2880409	0.1268396
C	-1.4205601	3.1799442	-0.9190016
C	-1.8033735	3.2730466	0.4036650
N	-0.7826325	1.9547325	-1.0694818
N	-1.3883471	2.1027411	1.0271890
C	-2.5437225	4.3816845	1.0708480
H	-2.5622383	5.2614245	0.4183705
H	-3.5862096	4.1061865	1.2838797
H	-2.0746445	4.6820359	2.0159962
C	-1.6494115	4.1648256	-2.0145222
H	-1.9653038	5.1245694	-1.5913672
H	-0.7409012	4.3467234	-2.6019687
H	-2.4372766	3.8326714	-2.7051770
C	-0.0972344	1.4373768	-2.2865389
H	0.1347084	0.3988957	-2.0039129
C	1.2293467	2.1659491	-2.5035351
H	1.0861648	3.2284396	-2.7399185
H	1.8654042	2.0873333	-1.6122642
H	1.7617290	1.7047250	-3.3458214
C	-0.9986108	1.4229138	-3.5207662
H	-1.9973475	1.0282119	-3.2919715
H	-1.1117708	2.4136835	-3.9753934

H	-0.5428140	0.7696761	-4.2767245
C	-1.4689307	1.7745355	2.4778470
H	-1.1670464	0.7163036	2.4989502
C	-2.8849283	1.8963733	3.0406554
H	-3.6330373	1.4666538	2.3608417
H	-2.9337010	1.3438486	3.9880032
H	-3.1688409	2.9333426	3.2542728
C	-0.4254262	2.5698841	3.2629878
H	0.5809313	2.3885237	2.8632717
H	-0.6233935	3.6494089	3.2391864
H	-0.4398844	2.2495885	4.3130950
C	-2.2044815	-1.5219177	-1.4760298
H	-2.6530192	-0.5303714	-1.6178678
H	-1.4467031	-1.6868974	-2.2511095
H	-2.9912827	-2.2850672	-1.5565378
C	-2.8359256	-1.4754582	1.3192336
H	-2.4792431	-1.5938276	2.3497617
H	-3.3076880	-0.4900691	1.2156597
H	-3.5780139	-2.2532989	1.0913967
C	-0.7393595	-3.2569808	0.3649869
H	-0.3091164	-3.3640848	1.3687721
H	-1.5381927	-3.9979098	0.2299113
H	0.0461050	-3.4244610	-0.3821115

[L_CP-PEt$_3$]$^+$ (1c$^+$)

P	0.2343110	-0.1971041	0.4114016
P	-1.4073835	-1.5294449	0.1221712
C	-0.7172593	1.3447758	0.1100498
C	-1.4472560	3.2493346	-0.8723152
C	-1.7657473	3.3196280	0.4677153
N	-0.8061194	2.0327421	-1.0717942
N	-1.3096834	2.1443772	1.0525330
C	-2.4880573	4.4084565	1.1854293
H	-2.5499710	5.2967703	0.5473335
H	-3.5154149	4.1148314	1.4440042
H	-1.9791056	4.7034314	2.1113399
C	-1.7457601	4.2423097	-1.9435192
H	-2.0643662	5.1893192	-1.4944346
H	-0.8695582	4.4521886	-2.5693086
H	-2.5561299	3.8984925	-2.6018295
C	-0.1561764	1.5555718	-2.3221834
H	0.1072283	0.5182022	-2.0674020
C	1.1469250	2.3158396	-2.5700079
H	0.9753836	3.3809957	-2.7727810
H	1.8170115	2.2274427	-1.7048232
H	1.6563749	1.8863602	-3.4427571
C	-1.0995887	1.5432494	-3.5242566
H	-2.0837488	1.1306545	-3.2647568
H	-1.2447061	2.5376695	-3.9616027
H	-0.6623191	0.9053080	-4.3034065
C	-1.2946226	1.8085472	2.5018433
H	-0.9690078	0.7580696	2.4948921
C	-2.6740163	1.8948263	3.1544411
H	-3.4520726	1.4443812	2.5234386
H	-2.6465520	1.3412732	4.1016470
H	-2.9705444	2.9243396	3.3866676
C	-0.2205333	2.6199975	3.2272450
H	0.7617519	2.4665137	2.7612548
H	-0.4429448	3.6950028	3.2305958
H	-0.1599456	2.2874538	4.2719385
C	-2.2299666	-1.4539462	-1.5240178
H	-2.5255054	-0.4010757	-1.6530982
H	-3.1613986	-2.0370551	-1.4604029
C	-2.8051612	-1.3749240	1.3114508
H	-3.1536821	-0.3343235	1.2219941
H	-3.6252614	-2.0134802	0.9475684
C	-0.7266026	-3.2207415	0.3120899
H	-0.2433539	-3.2520907	1.2997667
H	0.0769446	-3.3156804	-0.4330131
C	-2.4333623	-1.7204706	2.7556821
H	-3.2488953	-1.4440501	3.4364029
H	-2.2518008	-2.7966028	2.8760479
H	-1.5246032	-1.1934680	3.0782463
C	-1.7644169	-4.3395096	0.1615075
H	-1.2750284	-5.3158641	0.2746229
H	-2.5525256	-4.2772234	0.9243966

H	-2.2459627	-4.3255621	-0.8254686
C	-1.3513956	-1.9352534	-2.6817547
H	-1.8160735	-1.6831218	-3.6437900
H	-0.3551164	-1.4735461	-2.6529773
H	-1.2144571	-3.0241750	-2.6552568

Ph_2CS

C	-0.7059824	0.8906055	0.0088542
C	-1.4666892	2.1529555	0.1617154
C	-2.6746088	2.3510954	-0.5386489
C	-0.9787555	3.2008586	0.9670562
C	-3.3609359	3.5616338	-0.4468187
H	-3.0581950	1.5575528	-1.1798290
C	-1.6779682	4.3990219	1.0757593
H	-0.0433558	3.0505805	1.5073078
C	-2.8695564	4.5873919	0.3652302
H	-4.2823036	3.7042243	-1.0123946
H	-1.2936888	5.1923317	1.7187649
H	-3.4136760	5.5295803	0.4457867
C	-1.4773513	-0.3645752	-0.1514587
C	-2.6947245	-0.5500652	0.5359164
C	-0.9907175	-1.4177687	-0.9500532
C	-3.3914953	-1.7545150	0.4379310
H	-3.0778353	0.2476697	1.1717780
C	-1.6999645	-2.6099499	-1.0651652
H	-0.0481814	-1.2772555	-1.4802450
C	-2.9010840	-2.7858097	-0.3672420
H	-4.3202418	-1.8875439	0.9935898
H	-1.3161812	-3.4071943	-1.7028043
H	-3.4531304	-3.7229848	-0.4525355
S	0.9499024	0.8825337	0.0169982

TS-1 (Figure 5 main text)

P	1.038268300	-1.427860500	-0.471858100
P	-1.060688600	-2.532891600	0.740095500
C	0.194642500	0.190913500	-0.161202100
C	-1.039968200	2.043069300	-0.517224400
C	-0.543512800	2.106952400	0.768074200
N	-0.577580200	0.855430000	-1.069752900
N	0.208209100	0.956016000	0.969341500
C	-0.787526700	3.158339300	1.795398500
H	-1.205395900	4.052789400	1.320835200
H	-1.502573100	2.812477100	2.555822200
H	0.133246500	3.458951400	2.309235400
C	-1.881090100	3.045260600	-1.229946200
H	-2.278205900	3.774574400	-0.515206900
H	-1.297137900	3.592519800	-1.982907700
H	-2.736245600	2.582062900	-1.736869700
C	-0.900584000	0.310432100	-2.420549500
H	-0.210540100	-0.542513700	-2.507631200
C	-0.605314300	1.300473500	-3.549281600
H	-1.425184500	2.013519200	-3.698044800
H	0.324155200	1.852555700	-3.378530600
H	-0.494689900	0.736845900	-4.485155500
C	-2.333402400	-0.218526200	-2.472168200
H	-2.520871600	-0.963691900	-1.692487300
H	-3.072479000	0.588275100	-2.377039000
H	-2.496974400	-0.708722600	-3.440897200
C	0.961268800	0.577731700	2.199158800
H	1.262273900	-0.459210200	1.989334500
C	0.088221000	0.579968900	3.455457400
H	-0.897098600	0.139336500	3.271284400
H	0.589990200	-0.020941600	4.225087900
H	-0.044153900	1.587928600	3.865558900
C	2.223959700	1.425547700	2.361104000
H	2.871735900	1.349701800	1.480607400
H	1.983862400	2.481356500	2.541919700
H	2.788307100	1.061459200	3.230214700
C	2.796545100	-0.530586600	-1.982547700
S	3.175004700	-0.830001300	-0.313070900
C	2.890209400	-1.664543000	-2.939461500
C	1.980124400	-1.778007100	-4.011534200
C	3.855999100	-2.674447700	-2.784112800
C	2.032039100	-2.858319800	-4.887943000
H	1.216171600	-1.011513900	-4.146430200
C	3.915203700	-3.751941600	-3.669028400

H	4.566563600	-2.608392500	-1.960075000
C	3.003081000	-3.852298600	-4.721820700
H	1.310692900	-2.929025700	-5.703205400
H	4.681670300	-4.516009100	-3.534955600
H	3.047906900	-4.697165000	-5.409545000
C	2.685401100	0.857095300	-2.470682200
C	2.401927500	1.941637300	-1.609653300
C	2.945356100	1.163068200	-3.827131300
C	2.330275100	3.246962100	-2.082268100
H	2.242902400	1.750556400	-0.550391700
C	2.888084800	2.475618900	-4.296400000
H	3.223306800	0.366893100	-4.515300200
C	2.566031800	3.526069200	-3.434613000
H	2.100891800	4.056818600	-1.388192000
H	3.105931700	2.676928700	-5.345928600
H	2.517948600	4.550319900	-3.804690200
C	-2.089846300	-3.205971200	-0.601319200
C	-1.433628400	-3.684533700	-1.752608800
C	-3.494288600	-3.191004600	-0.562206400
C	-2.175900600	-4.128983000	-2.845831800
H	-0.343767200	-3.701513700	-1.798025200
C	-4.229533200	-3.633651000	-1.663806700
H	-4.012123000	-2.826991700	0.325639300
C	-3.573950100	-4.097391300	-2.807534500
H	-1.659498700	-4.498774100	-3.732404100
H	-5.319313700	-3.621005200	-1.624690300
H	-4.151755800	-4.440364700	-3.666377300
C	-0.360378400	-3.965108700	1.611438200
C	0.861740900	-3.809243100	2.285462300
C	-1.015090900	-5.207304100	1.637741100
C	1.416021200	-4.879224100	2.989678600
H	1.387615700	-2.853162100	2.246923700
C	-0.454592000	-6.275416500	2.339486200
H	-1.958797100	-5.336608800	1.105924700
C	0.758202800	-6.112114900	3.016511000
H	2.367071200	-4.754340500	3.508118200
H	-0.964992800	-7.238953100	2.355960200
H	1.195321600	-6.950584300	3.559659700
C	-2.198716500	-1.718166700	1.902369200
C	-2.815067800	-0.520977500	1.489304600
C	-2.389245000	-2.168793800	3.218563300
C	-3.616197100	0.201358800	2.371352900
H	-2.661817200	-0.150833600	0.474144800
C	-3.184912200	-1.434231700	4.102277900
H	-1.912616000	-3.091043800	3.552584100
C	-3.799094700	-0.251247000	3.683556200
H	-4.096117300	1.122000700	2.036959000
H	-3.327740600	-1.792025400	5.122436400
H	-4.420052200	0.317128500	4.376488800

TS-2 (Figure 5 main text)

P	-0.207169100	-2.107527800	0.274061700
P	3.539774400	0.254011500	-1.897033400
C	-1.135249300	-0.618040400	0.793948100
C	-2.855452200	0.798646800	1.177005500
C	-1.864163100	1.086549700	2.090656800
N	-2.391511700	-0.251351600	0.395401700
N	-0.814868200	0.210699900	1.833673200
C	-1.866719800	2.128252400	3.157308300
H	-2.886909600	2.496518000	3.314997700
H	-1.507555300	1.733932200	4.116019400
H	-1.240294900	2.992136900	2.893136000
C	-4.175717000	1.470092400	1.011018200
H	-4.368227900	2.128141500	1.866017500
H	-4.205078300	2.087876800	0.102664400
H	-5.001865300	0.750205700	0.954551200
C	-3.151569600	-1.013053900	-0.631761500
H	-2.371272800	-1.620279700	-1.109939000
C	-3.777797500	-0.120666700	-1.700803200
H	-4.714535000	0.343539500	-1.369820400
H	-3.079458200	0.661367300	-2.021918100
H	-4.009569200	-0.739822900	-2.577056400
C	-4.141354500	-1.961855400	0.044651400
H	-3.618700200	-2.637274000	0.735322500
H	-4.916121700	-1.418777700	0.602364300
H	-4.643276000	-2.569844800	-0.719970500

C	0.472613300	0.103409500	2.564729400
H	1.069834700	-0.544694400	1.905947000
C	0.284573500	-0.619873100	3.897953300
H	-0.162162300	-1.610372900	3.740489100
H	1.262590800	-0.758203400	4.378315000
H	-0.352901000	-0.053435900	4.590394400
C	1.186327800	1.450614500	2.689825700
H	1.083703900	2.036246000	1.766797100
H	0.815969100	2.052717800	3.528414300
H	2.255300500	1.268683900	2.863678400
C	0.442581500	-1.610427300	-1.396066700
S	2.200470900	-0.937061500	-0.654568700
C	5.209892100	0.102211600	-1.220493900
C	5.660035100	-1.160529500	-0.794366300
C	6.054322100	1.221265800	-1.127283300
C	6.955024200	-1.299936700	-0.295697100
H	4.992335800	-2.022077900	-0.844134100
C	7.344793700	1.070884000	-0.618804200
H	5.704093900	2.202497900	-1.449028000
C	7.796337300	-0.186496000	-0.206461300
H	7.304250300	-2.278957500	0.033255900
H	8.000264800	1.939178900	-0.546074500
H	8.805499500	-0.297912900	0.191271200
C	3.065638000	2.002431900	-1.834580500
C	2.704173000	2.528468200	-0.583895400
C	3.044878600	2.824890100	-2.970141400
C	2.308812200	3.859416800	-0.472748500
H	2.725449100	1.883920300	0.295320200
C	2.638672300	4.156268100	-2.853945400
H	3.338182300	2.429854100	-3.942323900
C	2.265758600	4.672958100	-1.610829200
H	2.033536600	4.264341800	0.501875100
H	2.616552700	4.791436000	-3.739828100
H	1.948069400	5.712737300	-1.526334500
C	3.527542100	-0.276634600	-3.619188000
C	4.576970700	-1.050924900	-4.141964600
C	2.374795700	-0.040330200	-4.389973000
C	4.462154900	-1.594258400	-5.421815200
H	5.474115200	-1.233612000	-3.550058900
C	2.271435300	-0.589645200	-5.665602700
H	1.556411000	0.557583800	-3.990580900
C	3.308728600	-1.372826900	-6.180438900
H	5.278090800	-2.192956300	-5.827493200
H	1.370283900	-0.411653300	-6.252710000
H	3.220494300	-1.808573400	-7.175961100
C	-0.176499400	-0.474091700	-2.185248200
C	-0.145890600	0.850520600	-1.708729700
C	-0.759207000	-0.704077200	-3.443193000
C	-0.622918500	1.908129900	-2.479126900
H	0.315073700	1.058755600	-0.744646500
C	-1.255077500	0.354974300	-4.210308100
H	-0.794586300	-1.715547600	-3.844507600
C	-1.174564900	1.667777100	-3.743349700
H	-0.540023600	2.926784100	-2.097696100
H	-1.689328500	0.147878200	-5.189220600
H	-1.537278100	2.495014800	-4.353932600
C	0.667972300	-2.878068200	-2.214726500
C	1.858074000	-3.157082200	-2.896818800
C	-0.402729200	-3.779868000	-2.366235900
C	1.975568100	-4.278135900	-3.719234800
H	2.713629200	-2.500044000	-2.775502300
C	-0.289318300	-4.901817900	-3.189298400
H	-1.334419400	-3.594207100	-1.831742600
C	0.900661800	-5.155377600	-3.874438300
H	2.918549200	-4.464468700	-4.234602300
H	-1.135208200	-5.583266400	-3.286509000
H	0.992583400	-6.034881900	-4.512430600

INT-I (Figure 5 main text)

P	-1.2690521	0.2494568	-0.0516812
P	-2.3123548	-1.3225929	0.9721803
C	-2.4185846	-2.7295214	-0.1780877
C	-2.6819760	-2.4987746	-1.5383146
C	-2.2926062	-4.0438937	0.2968788
C	-2.8259435	-3.5777042	-2.4119545
H	-2.7644783	-1.4750045	-1.9060621

C	-2.4396721	-5.1170267	-0.5826354
H	-2.0673622	-4.2284791	1.3472202
C	-2.7089222	-4.8867769	-1.9345990
H	-3.0272469	-3.3953583	-3.4680177
H	-2.3381552	-6.1366111	-0.2100912
H	-2.8213219	-5.7283097	-2.6190463
C	-1.4988327	-1.9634929	2.4737513
C	-2.2146595	-2.4470249	3.5818080
C	-0.0975455	-1.9888032	2.4812330
C	-1.5200529	-2.9317862	4.6924552
H	-3.3046052	-2.4407052	3.5830579
C	0.5895992	-2.4849328	3.5863855
H	0.4582914	-1.5994994	1.6297971
C	-0.1211093	-2.9496715	4.6970397
H	-2.0744917	-3.2973532	5.5574855
H	1.6800230	-2.4823488	3.5744192
H	0.4136250	-3.3250203	5.5704090
C	-4.0145890	-0.9112569	1.4882169
C	-4.2142984	-0.0230048	2.5605379
C	-5.1249517	-1.4607501	0.8304470
C	-5.5069822	0.3005287	2.9715292
H	-3.3604907	0.4028023	3.0882775
C	-6.4175282	-1.1359018	1.2496726
H	-4.9819710	-2.1543540	0.0017808
C	-6.6115670	-0.2594131	2.3202845
H	-5.6519590	0.9830954	3.8094438
H	-7.2752908	-1.5762798	0.7405887
H	-7.6217733	-0.0132152	2.6484557
C	-1.9891894	1.6006138	0.9486851
C	-3.3053603	3.3297516	1.5676681
C	-2.2978516	3.2107086	2.5032131
N	-3.0992448	2.3344774	0.6241817
N	-1.4940375	2.1522609	2.0994326
C	-2.0646709	4.0534574	3.7114553
H	-2.9752236	4.6103395	3.9595659
H	-1.7994931	3.4507695	4.5876052
H	-1.2593159	4.7837758	3.5498611
C	-4.4207109	4.3177697	1.5409061
H	-4.2358027	5.1080122	2.2768932
H	-4.5173195	4.8004599	0.5598565
H	-5.3850398	3.8489512	1.7816827
C	-3.8606396	2.1043359	-0.6343556
H	-3.5154140	1.1046896	-0.9376600
C	-3.4159084	3.1064256	-1.7001915
H	-3.6574936	4.1400387	-1.4162350
H	-2.3339167	3.0341299	-1.8746598
H	-3.9325996	2.8891304	-2.6447272
C	-5.3728075	2.0506223	-0.4302131
H	-5.6346793	1.4682834	0.4608410
H	-5.8293500	3.0447151	-0.3565333
H	-5.8195091	1.5515197	-1.3001278
C	-0.2275163	1.6917641	2.7338553
H	0.1394268	0.9345788	2.0218657
C	-0.4624754	1.0039077	4.0771531
H	-1.2216643	0.2157935	4.0012142
H	0.4737511	0.5226136	4.3880105
H	-0.7545461	1.7091641	4.8654735
C	0.7987973	2.8229559	2.8120712
H	0.8153421	3.4108230	1.8847812
H	0.6116404	3.5005684	3.6538878
H	1.7953540	2.3830704	2.9448185
C	2.4527275	-0.4974843	0.0800492
C	2.3486317	0.4632097	-1.0351948
C	2.6961832	0.0842067	-2.3481310
C	1.9220752	1.7869304	-0.8091399
C	2.6217370	1.0015006	-3.3959319
H	3.0555737	-0.9273071	-2.5374462
C	1.8241072	2.6927206	-1.8589778
H	1.6651991	2.0800236	0.2079574
C	2.1760873	2.3038082	-3.1577478
H	2.9169960	0.6992379	-4.4010997
H	1.4790793	3.7100469	-1.6692530
H	2.1094907	3.0175113	-3.9797034
C	2.1499148	-1.9190210	-0.1914469
C	1.1360863	-2.2707952	-1.1084633
C	2.7881341	-2.9480461	0.5310542

C	0.7559222	-3.6012644	-1.2711961
H	0.6182142	-1.4885298	-1.6628387
C	2.4209119	-4.2773743	0.3490509
H	3.5779469	-2.6810720	1.2333444
C	1.3953106	-4.6078806	-0.5451442
H	-0.0527247	-3.8501273	-1.9579899
H	2.9367263	-5.0621687	0.9032997
H	1.0989804	-5.6490436	-0.6781901
S	2.8609216	-0.0063307	1.6154751

INT-II (Figure 5 main text)

P	-2.34004545592729	-1.61253299682511	-0.03215577847922
P	-5.14937600590042	-2.24722208033634	-0.19551757755629
C	-6.54924885091714	-1.16590116313660	0.26185513362171
C	-6.38013629370673	-0.30016077648390	1.35698176574532
C	-7.74399154835038	-1.10847872536609	-0.47228457916339
C	-7.38112324216507	0.60375498362537	1.70841615085607
H	-5.46202951324018	-0.34387293843079	1.94319675129925
C	-8.74030168476186	-0.19192983667446	-0.12566381923668
H	-7.89490415402237	-1.78072989681933	-1.31686982479843
C	-8.56212513203396	0.66791729472546	0.96064033555189
H	-7.24317354862269	1.25870896035088	2.56992894046167
H	-9.66490678642347	-0.15843841073220	-0.70296397104616
H	-9.34415305749855	1.37797235177035	1.23146438211991
C	-5.73922696616611	-3.17928064015513	-1.64435602838218
C	-6.02070425324240	-4.55237636457496	-1.60077207782051
C	-5.82831316488786	-2.49520969396865	-2.87079211831832
C	-6.37974552375954	-5.23001391487510	-2.76844296621838
H	-5.95311898884944	-5.09090028341332	-0.65508737694341
C	-6.19894040047143	-3.17404556805918	-4.03067349548090
H	-5.60752174371322	-1.42690956544546	-2.91460599360980
C	-6.46779270249965	-4.54571161107428	-3.98293068105713
H	-6.59752992304036	-6.29773214366984	-2.72605100801181
H	-6.27275071190438	-2.63472166974723	-4.97590155687689
H	-6.74726926754834	-5.07852884679381	-4.89192908562912
C	-5.04775743425421	-3.45709523350670	1.16484524328982
C	-3.95108428271271	-4.33966419892047	1.18075741781407
C	-5.98599364896967	-3.51147024689135	2.20805977580150
C	-3.80024319399819	-5.25546712574846	2.22158194962171
H	-3.21785734325468	-4.31854705360445	0.37270053534512
C	-5.82049468624649	-4.41967872548141	3.25584249761154
H	-6.84908954146552	-2.84508474490212	2.20236201053636
C	-4.72765276874169	-5.28987049050315	3.26718852036801
H	-2.95053922178646	-5.93935829760155	2.21827314876387
H	-6.55537474314554	-4.45227488408679	4.06157985826649
H	-4.60186676965638	-5.99863716973481	4.08669913516037
C	-2.96404949315216	0.05182548255387	0.50295753834016
C	-4.05686170931191	1.98373827840123	0.88033538205002
C	-3.58912808033538	1.52656096803777	2.09310325563312
N	-3.66385073291068	1.05575112407525	-0.08056340632483
N	-2.91605460839145	0.33341630252532	1.83806257018504
C	-3.80799064531050	2.11984671188714	3.44059860230865
H	-4.19515542973517	3.13931350376731	3.33349618147976
H	-4.54232949768726	1.54585131720856	4.02444823495904
H	-2.88559095725622	2.17937999461382	4.02939335566262
C	-4.89893121150689	3.17857201353147	0.60567853908051
H	-4.90202461508266	3.83955457315330	1.48019453406860
H	-4.53346407901449	3.75934368591483	-0.24996392957684
H	-5.93989518664155	2.88719567897830	0.40189340111130
C	-3.84017323068547	1.16383208835143	-1.54847756107850
H	-3.49979002811467	0.19065591636860	-1.91717294170154
C	-2.91346362959343	2.24171384349983	-2.10411563780752
H	-3.23517581875902	3.24722165676477	-1.80105706859963
H	-1.88194710225828	2.08294005371004	-1.76866878369433
H	-2.92348279969708	2.19604872812684	-3.20002094727112
C	-5.29490463317816	1.34519956707590	-1.97417614237131
H	-5.96786179173647	0.67981639762386	-1.42140945447755
H	-5.64100226014146	2.37788994178614	-1.85109316495552
H	-5.36802615289479	1.10384549683866	-3.04342661071575
C	-2.21871190286698	-0.53726959168003	2.82694235743366
H	-1.83873768839968	-1.36958016691450	2.21623788094517
C	-3.19478596476983	-1.13675134631479	3.83455982608058
H	-3.99795659735531	-1.68814119210465	3.32571775020174
H	-2.65305377936414	-1.85213419624874	4.46769996530776
H	-3.64037049846343	-0.38257324852019	4.49486534009782
C	-1.01017897683288	0.16998801736271	3.43616178743252

H -0.37451625441963 0.59631772260920 2.64921083228454
H -1.29210248350401 0.96641339381917 4.13686110498924
H -0.41453924775352 -0.56442077654009 3.99450435454460
C -1.40167377841025 -1.64690161002394 -1.66073280743575
C -1.54981938096896 -0.79706174816771 -2.89230015361439
C -2.60312987804312 -1.04239192215534 -3.79191852108651
C -0.58023021643349 0.15291122781185 -3.23339349419933
C -2.69659481520752 -0.32808961424464 -4.98479175799769
H -3.34447297067427 -1.80699962995336 -3.56089209535124
C -0.66168471661402 0.85242958888102 -4.44089411979656
H 0.24758560689152 0.33221609706143 -2.54618846183479
C -1.72246799610385 0.62101629675754 -5.31716425050636
H -3.52077900103971 -0.52933542929502 -5.67064216087972
H 0.11150724678391 1.57825624084046 -4.69554491923975
H -1.78495852874731 1.16438284199890 -6.26020725411448
C -1.24135004199960 -3.12225992548528 -2.01213083366804
C -2.36806711250856 -3.92714872971556 -2.22271322883878
C 0.03306435714619 -3.67801804203283 -2.17621893529980
C -2.22983754796277 -5.26636546602188 -2.59182554484649
H -3.36547811422345 -3.50681298881582 -2.10556278748364
C 0.17285847193814 -5.01288171750934 -2.55705579807834
H 0.91485764388054 -3.06374769182909 -1.99124298747867
C -0.95643512331953 -5.81214162828068 -2.76322865048290
H -3.12146165514870 -5.87531814537572 -2.74421286469609
H 1.17038261238284 -5.43394327583587 -2.68667615949681
H -0.84106409196619 -6.85672563178232 -3.05340863620982
S -0.23709802864254 -1.07711282999802 -0.35204813659208

INT-III (Figure 5 main text)

P -4.0211899 -2.7238196 0.2091628
P -8.6117002 -4.6071085 -0.6673283
C -10.1987769 -3.8077064 -0.2513430
C -10.1738707 -2.7429424 0.6645722
C -11.4165426 -4.2208796 -0.8072020
C -11.3584291 -2.1046402 1.0253257
H -9.2210443 -2.4198644 1.0883444
C -12.6012753 -3.5704570 -0.4480689
H -11.4424525 -5.0463666 -1.5193019
C -12.5745502 -2.5172732 0.4677767
H -11.3368220 -1.2829971 1.7424455
H -13.5468017 -3.8912085 -0.8862962
H -13.5007430 -2.0148622 0.7492541
C -9.0063991 -5.8990066 -1.8980908
C -9.4751015 -7.1643113 -1.5111906
C -8.8835318 -5.5873227 -3.2599557
C -9.8244009 -8.1042884 -2.4830329
H -9.5620846 -7.4207060 -0.4549454
C -9.2377159 -6.5299327 -4.2260545
H -8.5001523 -4.6085264 -3.5521235
C -9.7100658 -7.7874897 -3.8393202
H -10.1837095 -9.0879516 -2.1786639
H -9.1416951 -6.2822690 -5.2838480
H -9.9844395 -8.5237627 -4.5956280
C -8.1324109 -5.4992891 0.8535483
C -6.7800930 -5.7849117 1.0894345
C -9.1078997 -5.9485199 1.7580471
C -6.4087954 -6.5258159 2.2120254
H -6.0214665 -5.4118789 0.4007556
C -8.7304631 -6.6899174 2.8799019
H -10.1598590 -5.7131546 1.5893314
C -7.3828870 -6.9826454 3.1055586
H -5.3554157 -6.7471035 2.3896308
H -9.4916270 -7.0361522 3.5798165
H -7.0909769 -7.5631694 3.9815818
C -4.0610700 -0.9217234 0.6243670
C -4.5119711 1.2772007 0.7551449
C -3.6697457 0.9608535 1.7999768
N -4.7521602 0.0992853 0.0546151
N -3.4080278 -0.4014998 1.7029815
C -3.1013978 1.8709804 2.8346171
H -3.6720112 2.8061953 2.8593399
H -3.1438579 1.4327884 3.8391692
H -2.0537360 2.1285993 2.6229750
C -5.0412288 2.6182428 0.3796136
H -4.8742918 3.3263923 1.1989894
H -4.5322257 3.0124912 -0.5107764

H	-6.1172171	2.5974597	0.1711394
C	-5.6899562	-0.0855393	-1.0902535
H	-5.5138023	-1.1242173	-1.4009638
C	-5.3547782	0.8300263	-2.2675705
H	-5.7366114	1.8481524	-2.1252893
H	-4.2738316	0.8722965	-2.4463549
H	-5.8301415	0.4237951	-3.1689479
C	-7.1381045	0.0311316	-0.6143188
H	-7.3325314	-0.6620896	0.2130417
H	-7.3888778	1.0536339	-0.3022615
H	-7.8070833	-0.2517500	-1.4363647
C	-2.5702752	-1.2368561	2.6075838
H	-2.4952166	-2.1944519	2.0651658
C	-3.2940712	-1.4959187	3.9283421
H	-4.2813989	-1.9437012	3.7522044
H	-2.7031440	-2.1924634	4.5374588
H	-3.4287124	-0.5743385	4.5098491
C	-1.1529957	-0.6878333	2.7737694
H	-0.7174081	-0.3997472	1.8078803
H	-1.1124526	0.1740448	3.4497442
H	-0.5213423	-1.4743292	3.2068029
C	-3.2092252	-2.7328261	-1.3104552
C	-2.6581501	-1.5753105	-2.0400059
C	-2.9244807	-1.4568431	-3.4198887
C	-1.8776063	-0.5798076	-1.4226381
C	-2.4573853	-0.3623150	-4.1451411
H	-3.5179394	-2.2269823	-3.9123183
C	-1.4023917	0.5092408	-2.1516197
H	-1.6125929	-0.6851946	-0.3712513
C	-1.6976502	0.6271605	-3.5139564
H	-2.6871913	-0.2808300	-5.2077869
H	-0.7839286	1.2611868	-1.6600538
H	-1.3257495	1.4798584	-4.0828301
C	-3.0673860	-4.0578054	-1.9430731
C	-4.0823893	-5.0328868	-1.8592792
C	-1.8678618	-4.3855202	-2.6134342
C	-3.8941106	-6.3007765	-2.4042734
H	-5.0492024	-4.7603137	-1.4311587
C	-1.6816830	-5.6575066	-3.1468249
H	-1.0754057	-3.6411971	-2.6908323
C	-2.6912880	-6.6213517	-3.0406037
H	-4.6989815	-7.0346885	-2.3475776
H	-0.7444002	-5.9011890	-3.6479472
H	-2.5444134	-7.6144211	-3.4666585
S	-7.2510356	-3.3229453	-1.2994304

$(Me_2N)_2CS$

C	-0.5106264	-0.5239420	-0.8308670
S	-0.2793463	-0.1221099	-2.4431962
N	0.3219054	-1.3908015	-0.1484144
N	-1.5496728	-0.0150664	-0.0740729
C	0.7068797	-1.1344018	1.2400135
H	0.4915307	-1.9975375	1.8900335
H	1.7896241	-0.9287520	1.2882519
H	0.1755117	-0.2584530	1.6245621
C	1.2783986	-2.2049403	-0.8861827
H	2.1772138	-1.6279025	-1.1652411
H	1.5769558	-3.0499101	-0.2482000
H	0.8202204	-2.5819611	-1.8063033
C	-2.2748057	-0.8571630	0.8784969
H	-3.3134482	-0.9924530	0.5321107
H	-1.8065072	-1.8442394	0.9450804
H	-2.3032508	-0.4058157	1.8830865
C	-2.3260020	1.1094480	-0.5791484
H	-2.8433854	1.5803326	0.2695294
H	-1.6634404	1.8390144	-1.0565713
H	-3.0728293	0.7886044	-1.3256733

Int-I (Figure S125)

P	0.1257760	0.3091736	0.5513368
P	-1.8912481	-0.2977162	0.1252952
C	-1.8876092	-2.1112252	0.1421757
C	-0.8610193	-2.7993786	-0.5249961
C	-2.8829010	-2.8251311	0.8241312
C	-0.8327544	-4.1927328	-0.5051052
H	-0.0773898	-2.2391378	-1.0368582

C	-2.8400072	-4.2191924	0.8483823
H	-3.6739584	-2.2966608	1.3551538
C	-1.8169478	-4.9031141	0.1876396
H	-0.0318304	-4.7249072	-1.0193638
H	-3.6043169	-4.7723854	1.3944401
H	-1.7849001	-5.9926651	0.2155581
C	-3.1642338	0.2319797	1.3277179
C	-4.4425097	0.6651977	0.9413336
C	-2.8358450	0.1572510	2.6916739
C	-5.3694548	1.0519881	1.9132229
H	-4.7208821	0.7008815	-0.1113359
C	-3.7716059	0.5304785	3.6550351
H	-1.8596796	-0.2324226	2.9950379
C	-5.0347319	0.9911075	3.2679390
H	-6.3585266	1.3949096	1.6077881
H	-3.5133129	0.4551301	4.7119359
H	-5.7619920	1.2917815	4.0230131
C	-2.5018693	0.2717521	-1.5008394
C	-2.8124802	1.6304257	-1.6943396
C	-2.6545255	-0.6258040	-2.5684177
C	-3.2802154	2.0770040	-2.9295876
H	-2.7100487	2.3401223	-0.8734786
C	-3.1195437	-0.1717422	-3.8053301
H	-2.4275377	-1.6832799	-2.4307334
C	-3.4374065	1.1761457	-3.9881575
H	-3.5297034	3.1302219	-3.0637396
H	-3.2434602	-0.8791521	-4.6256766
H	-3.8085875	1.5245967	-4.9522420
C	-0.0633293	1.9776125	-0.1746182
C	0.1348347	3.7374580	-1.5792565
C	-0.4195858	4.1990081	-0.4028639
N	0.3416926	2.3737260	-1.4198069
N	-0.5257688	3.1056894	0.4485094
C	-0.8678568	5.5824017	-0.0757305
H	-0.4637445	6.2887927	-0.8098101
H	-1.9633417	5.6710032	-0.0999226
H	-0.5280465	5.9041986	0.9158429
C	0.4444859	4.5072164	-2.8170486
H	0.4099778	5.5824476	-2.6077147
H	1.4449549	4.2775143	-3.2041062
H	-0.2812106	4.2989997	-3.6158291
C	0.9694816	1.4271230	-2.3812984
H	0.7317583	0.4500770	-1.9314871
C	2.4885379	1.5976379	-2.3742045
H	2.7953020	2.5829215	-2.7504040
H	2.8900811	1.4715579	-1.3596187
H	2.9429961	0.8360385	-3.0218945
C	0.3491460	1.4774459	-3.7752905
H	-0.7452822	1.5032980	-3.7229156
H	0.7000954	2.3297500	-4.3694172
H	0.6379789	0.5648737	-4.3135900
C	-0.9804431	3.0964085	1.8654593
H	-0.9952913	2.0219235	2.1010582
C	-2.3966466	3.6379185	2.0379546
H	-3.0854207	3.1885379	1.3108941
H	-2.7558382	3.3545902	3.0355362
H	-2.4501852	4.7304645	1.9591971
C	0.0586111	3.7542729	2.7729832
H	1.0383142	3.2739786	2.6471347
H	0.1670811	4.8297704	2.5817468
H	-0.2505659	3.6314671	3.8194991
C	1.3943870	-1.5603149	3.1756022
S	-0.1536802	-2.2182472	3.2928134
N	2.3626458	-2.1070141	2.3693242
N	1.7833498	-0.4379734	3.8761749
C	3.3431841	-1.2800335	1.6668346
H	4.3766204	-1.5506513	1.9349218
H	3.2174395	-1.4183702	0.5805918
H	3.1765295	-0.2229355	1.8961235
C	2.1829711	-3.4380696	1.8041332
H	1.5504920	-3.4090940	0.9017252
H	3.1720448	-3.8395334	1.5426824
H	1.7006991	-4.0961638	2.5340199
C	3.1024565	-0.3640311	4.5128038
H	2.9778856	-0.3215784	5.6066822
H	3.6899455	-1.2558466	4.2742983

H	3.6615039	0.5291209	4.1925631
C	0.7739955	0.4114025	4.4924983
H	1.2460124	1.3681334	4.7567643
H	-0.0336913	0.6003321	3.7777329
H	0.3449760	-0.0452939	5.4000830

Int-II (Figure S125)

P	-2.2795415	-1.6632491	-0.1593783
P	-5.2262115	-2.2259191	-0.1154511
C	-6.6225201	-1.0802302	0.1844821
C	-6.4556992	-0.1245415	1.2034862
C	-7.7902599	-1.0441502	-0.5931699
C	-7.4251203	0.8497407	1.4327122
H	-5.5597070	-0.1504008	1.8244671
C	-8.7559489	-0.0580605	-0.3693031
H	-7.9490031	-1.7853317	-1.3768320
C	-8.5753301	0.8938797	0.6367762
H	-7.2834981	1.5773412	2.2331649
H	-9.6589519	-0.0411933	-0.9805670
H	-9.3310216	1.6609788	0.8075518
C	-5.7340650	-3.2652667	-1.5239380
C	-5.9112934	-4.6532973	-1.4331489
C	-5.8256718	-2.6418052	-2.7833428
C	-6.1709011	-5.4030589	-2.5836521
H	-5.8419410	-5.1501075	-0.4648284
C	-6.1001218	-3.3913537	-3.9256218
H	-5.6893137	-1.5604830	-2.8635918
C	-6.2654936	-4.7772753	-3.8285565
H	-6.3057698	-6.4822789	-2.5035396
H	-6.1785566	-2.8963766	-4.8943263
H	-6.4695915	-5.3665877	-4.7226744
C	-5.2097365	-3.3101393	1.3576011
C	-4.1050435	-4.1636787	1.5408701
C	-6.2164762	-3.2798493	2.3373313
C	-4.0107478	-4.9634426	2.6803578
H	-3.3142973	-4.2009530	0.7902111
C	-6.1103370	-4.0722436	3.4821095
H	-7.0849425	-2.6338560	2.2083762
C	-5.0077562	-4.9122077	3.6585578
H	-3.1526956	-5.6249409	2.8064514
H	-6.8983875	-4.0380541	4.2353583
H	-4.9287074	-5.5308502	4.5531106
C	-2.9420796	-0.0231956	0.4617266
C	-3.8972510	1.9691736	0.8879230
C	-3.4814964	1.4420031	2.0895282
N	-3.5617719	1.0445609	-0.0984064
N	-2.8981677	0.2086902	1.8049610
C	-3.6727175	2.0069692	3.4550583
H	-3.9023435	3.0762126	3.3834716
H	-4.5092210	1.5224030	3.9794829
H	-2.7787318	1.9028000	4.0800022
C	-4.6474163	3.2319707	0.6467531
H	-4.5985832	3.8691021	1.5367846
H	-4.2406400	3.8050432	-0.1946743
H	-5.7079510	3.0250593	0.4402580
C	-3.7546472	1.1858936	-1.5629688
H	-3.4704477	0.1997424	-1.9421893
C	-2.7608237	2.2000345	-2.1273464
H	-2.9935233	3.2244465	-1.8076224
H	-1.7399238	1.9474230	-1.8186879
H	-2.8050166	2.1756156	-3.2238593
C	-5.2044247	1.4633596	-1.9612419
H	-5.9069161	0.8237791	-1.4144553
H	-5.4912889	2.5103809	-1.8107684
H	-5.3098935	1.2505115	-3.0339900
C	-2.2773673	-0.7414310	2.7737480
H	-2.0326229	-1.6170785	2.1519933
C	-3.2696060	-1.2157750	3.8338285
H	-4.1977756	-1.5807751	3.3732179
H	-2.8227412	-2.0578402	4.3782568
H	-3.5102010	-0.4373629	4.5670967
C	-0.9667721	-0.1827281	3.3265682
H	-0.3052028	0.1355590	2.5103657
H	-1.1266037	0.6679286	4.0012321
H	-0.4544645	-0.9679485	3.8977372
C	-1.3554842	-1.5654450	-1.8284546

S	-0.2194535	-1.0169665	-0.4786193
N	-1.1608833	-2.9665353	-2.1181181
N	-1.5513890	-0.5954350	-2.8638679
C	-0.3545201	0.0205568	-3.4450232
H	0.3066212	0.3764658	-2.6460400
H	0.2126422	-0.6679094	-4.0983519
H	-0.6614450	0.8876606	-4.0469230
C	-2.5124117	-0.9919398	-3.8919197
H	-2.1006891	-1.7166324	-4.6187036
H	-3.4016372	-1.4410834	-3.4308465
H	-2.8263775	-0.0979361	-4.4514398
C	0.1109281	-3.3233534	-2.7388492
H	0.9267949	-2.7661176	-2.2597661
H	0.2957496	-4.3968852	-2.5907281
H	0.1288521	-3.1200253	-3.8267250
C	-2.2799243	-3.7915324	-2.5619824
H	-2.4256970	-3.7930931	-3.6575813
H	-2.1060609	-4.8307984	-2.2444809
H	-3.2127328	-3.4494484	-2.0994966

Int-III (Figure S125)

P	-4.2195413	-2.1137312	-1.0422709
C	-3.8708782	-0.5110245	-0.2385313
C	-4.0476584	1.6900984	0.2511724
C	-3.3398825	1.0883499	1.2672271
N	-4.3579558	0.6936728	-0.6684626
N	-3.2600211	-0.2681923	0.9600873
C	-2.7647687	1.7340312	2.4833622
H	-2.6501395	2.8109020	2.3134501
H	-3.4096163	1.5989429	3.3633840
H	-1.7740476	1.3350716	2.7298204
C	-4.4277090	3.1268059	0.1227474
H	-4.3040693	3.6316454	1.0874330
H	-3.8077803	3.6553842	-0.6155833
H	-5.4771483	3.2467599	-0.1737814
C	-5.1405305	0.8306335	-1.9233091
H	-4.9723780	-0.1397199	-2.4196755
C	-4.6085175	1.9350806	-2.8378060
H	-4.9131289	2.9368492	-2.5129542
H	-3.5129392	1.9062913	-2.9068701
H	-5.0149421	1.7819519	-3.8462375
C	-6.6347924	0.9496295	-1.6199071
H	-6.9769014	0.1032364	-1.0097574
H	-6.8725182	1.8803328	-1.0876611
H	-7.2003334	0.9483493	-2.5608795
C	-2.7044950	-1.3529799	1.8141153
H	-3.0395919	-2.2668087	1.2995025
C	-3.3209278	-1.3526781	3.2121045
H	-4.4154056	-1.2985761	3.1600899
H	-3.0642879	-2.2996009	3.7049752
H	-2.9415468	-0.5386656	3.8414237
C	-1.1767002	-1.3283966	1.8292527
H	-0.7777551	-1.2503242	0.8120945
H	-0.7799089	-0.4947925	2.4233670
H	-0.8041057	-2.2596053	2.2761297
C	-2.5784619	-2.6800137	-1.4411209
N	-1.4869701	-1.9012175	-1.7362220
N	-2.3974913	-4.0306525	-1.5707293
C	-1.6317940	-4.5942694	-2.6855528
H	-0.7170479	-5.1017339	-2.3416544
H	-2.2601090	-5.3339562	-3.2036918
H	-1.3631998	-3.8088874	-3.3991645
C	-3.1929553	-5.0142279	-0.8352368
H	-3.6483442	-4.5611051	0.0533200
H	-3.9910404	-5.4344273	-1.4663552
H	-2.5310550	-5.8355773	-0.5210195
C	-1.6043433	-0.5412541	-2.2416822
H	-2.5768856	-0.4155696	-2.7294750
H	-1.5015781	0.2121109	-1.4417604
H	-0.8103359	-0.3661232	-2.9821638
C	-0.1169150	-2.3537818	-1.4909417
H	0.4075325	-2.6228977	-2.4217674
H	0.4486227	-1.5427017	-1.0085777
H	-0.1248753	-3.2205485	-0.8222267
P	-6.4831355	-5.4175662	1.8623702
C	-8.1382012	-4.7214350	2.2038278

C	-8.3066380	-3.9425943	3.3582324
C	-9.2420619	-4.9976970	1.3821710
C	-9.5694855	-3.4522887	3.6904804
H	-7.4410923	-3.7196603	3.9847124
C	-10.5030537	-4.4978208	1.7166350
H	-9.1184334	-5.5941861	0.4773402
C	-10.6684038	-3.7283082	2.8706488
H	-9.6968391	-2.8523958	4.5924523
H	-11.3568559	-4.7104524	1.0722464
H	-11.6540825	-3.3407979	3.1309529
C	-6.4838976	-5.7909048	0.0731501
C	-6.1836974	-7.0686644	-0.4193570
C	-6.7219245	-4.7376478	-0.8247736
C	-6.1261989	-7.2902525	-1.7985604
H	-5.9987988	-7.8913453	0.2719244
C	-6.6680624	-4.9639988	-2.1991615
H	-6.9369172	-3.7399956	-0.4418088
C	-6.3697108	-6.2411517	-2.6888657
H	-5.8957957	-8.2870802	-2.1759835
H	-6.8528221	-4.1410749	-2.8904507
H	-6.3305266	-6.4181296	-3.7644806
C	-6.4668981	-7.0435080	2.6978632
C	-5.3012162	-7.4798622	3.3398357
C	-7.6030809	-7.8683719	2.6729992
C	-5.2694167	-8.7370747	3.9477341
H	-4.4312058	-6.8219063	3.3687173
C	-7.5656063	-9.1231748	3.2815949
H	-8.5191545	-7.5295170	2.1876372
C	-6.3987101	-9.5592912	3.9178717
H	-4.3617951	-9.0719871	4.4512056
H	-8.4515250	-9.7589851	3.2655579
H	-6.3740672	-10.5386672	4.3974535
S	-5.0055230	-4.2275662	2.3978875

TS-I (Figure S125)

P	1.685000900	-1.290979400	-0.927033900
P	-1.455485800	-2.501722900	0.803905600
C	0.706320400	0.087588500	-0.212934400
C	-1.062791800	1.461907100	0.058745300
C	-0.437457000	1.331083300	1.280711700
N	-0.333640800	0.703513100	-0.850285500
N	0.641285100	0.478233300	1.095002800
C	-0.858522600	1.910478100	2.586133300
H	-1.559747200	2.735173200	2.419031000
H	-1.371222200	1.155413500	3.200399700
H	-0.011387200	2.308246400	3.157266700
C	-2.299003500	2.233707700	-0.252665800
H	-2.822069900	2.469986700	0.680224800
H	-2.084229300	3.177047900	-0.774237700
H	-2.994262600	1.655155100	-0.873405100
C	-0.632148900	0.488225400	-2.290997700
H	0.313486500	0.077966600	-2.679174700
C	-0.939795800	1.793337600	-3.028489700
H	-1.981631300	2.108696700	-2.902140400
H	-0.287718200	2.612822200	-2.698146700
H	-0.777110600	1.637291100	-4.102848300
C	-1.721791000	-0.567554100	-2.474960100
H	-1.447345900	-1.510294800	-1.987196400
H	-2.682052000	-0.236267400	-2.056541700
H	-1.870783400	-0.757615100	-3.546319200
C	1.629809100	0.036867100	2.115663700
H	2.146703200	-0.795253500	1.617741900
C	0.982255200	-0.521485600	3.382110100
H	0.144913200	-1.185234500	3.143759600
H	1.733747500	-1.112478000	3.921625400
H	0.628969800	0.263440800	4.059900600
C	2.641265200	1.149447600	2.393442400
H	3.128388800	1.473781400	1.466011100
H	2.163990800	2.017480800	2.867542000
H	3.415890300	0.777000100	3.076561500
C	3.255216300	-0.150624900	-2.253891700
S	3.734055100	-0.643511300	-0.606559600
C	-2.672616400	-3.459096400	-0.189523200
C	-2.158104300	-4.135889100	-1.309941600
C	-4.043683600	-3.556328500	0.092042700
C	-2.992822700	-4.902259200	-2.123185900

H	-1.090826700	-4.068803500	-1.537015600
C	-4.880772400	-4.314601400	-0.731353600
H	-4.461785000	-3.042070300	0.958284800
C	-4.359190400	-4.989197800	-1.837892600
H	-2.578421500	-5.429568000	-2.983484400
H	-5.944799500	-4.384209900	-0.500868800
H	-5.014792000	-5.582118000	-2.476470900
C	-0.722514500	-3.809895100	1.866081600
C	0.666216500	-3.798929800	2.064827600
C	-1.493331900	-4.814882200	2.474895500
C	1.275140300	-4.760640200	2.875715800
H	1.272228400	-3.042601300	1.560747200
C	-0.885181500	-5.775990800	3.282166100
H	-2.571730100	-4.844746000	2.309147300
C	0.499190000	-5.747554700	3.487414200
H	2.356260600	-4.746214200	3.020538300
H	-1.490366700	-6.553882300	3.749468400
H	0.972249900	-6.502783200	4.116186100
C	-2.519662400	-1.551843300	1.965879800
C	-3.370869000	-0.573003900	1.418789900
C	-2.457229700	-1.674854100	3.362875300
C	-4.142826200	0.247530300	2.238953400
H	-3.439451400	-0.470008700	0.333843800
C	-3.217371400	-0.837972300	4.187742400
H	-1.821955000	-2.438556600	3.813467700
C	-4.063186300	0.123364900	3.631334600
H	-4.814495500	0.983178300	1.793387200
H	-3.156350100	-0.951240600	5.270997500
H	-4.664096800	0.766022700	4.275334200
N	3.601842700	-0.927957300	-3.379956200
N	3.056342300	1.174874700	-2.537242900
C	3.428764200	-2.375809100	-3.309291900
H	2.383854000	-2.613911100	-3.075758200
H	3.670784200	-2.795550900	-4.293649900
H	4.081361400	-2.851411200	-2.553978800
C	4.921002500	-0.564385500	-3.936874000
H	5.744169500	-0.942241100	-3.301673200
H	5.016667300	-1.004303600	-4.937418000
H	5.016322600	0.523074000	-4.022717400
C	3.109737800	2.217750600	-1.524139300
H	3.310009500	3.175890100	-2.020155000
H	2.155310600	2.299068900	-0.974283900
H	3.911189500	2.020322600	-0.805182400
C	2.504011600	1.581145100	-3.829912600
H	1.641299000	2.242156700	-3.675002000
H	3.253942300	2.131395700	-4.419443100
H	2.200479900	0.693775900	-4.393229600

TS-II (Figure S125)

P	-0.226168100	-2.054691300	-0.168616900
P	3.635554300	0.180290500	-2.009020400
C	-1.361230700	-0.971350000	0.743203700
C	-3.207016300	0.153909300	1.406611300
C	-2.232349000	0.346961600	2.362177600
N	-2.657606500	-0.666878800	0.427451800
N	-1.107879800	-0.357211600	1.941376100
C	-2.311591200	1.170918900	3.602233800
H	-3.359719700	1.378692700	3.846754300
H	-1.866207200	0.662306800	4.464850900
H	-1.802707600	2.138472100	3.482936800
C	-4.582351200	0.727282200	1.365900200
H	-4.842516400	1.144339900	2.345502600
H	-4.659606800	1.540547000	0.629827000
H	-5.339722500	-0.024845400	1.114442800
C	-3.341546800	-1.273414300	-0.742787400
H	-2.516846200	-1.791694500	-1.252574600
C	-3.913149500	-0.234707900	-1.703807400
H	-4.790685100	0.283779200	-1.298666700
H	-3.146979200	0.501263400	-1.975828000
H	-4.231302200	-0.746404600	-2.622265500
C	-4.350148400	-2.332198900	-0.294928600
H	-3.872213100	-3.065186900	0.368274800
H	-5.213650300	-1.898481500	0.225817200
H	-4.728983500	-2.864556700	-1.177663300
C	0.216601200	-0.450133500	2.609293400
H	0.810597400	-1.019678200	1.877994700

C	0.141389800	-1.269192200	3.897598800
H	-0.337135800	-2.240049500	3.713811500
H	1.160160400	-1.455049600	4.263173200
H	-0.406950600	-0.754408400	4.697029300
C	0.870511700	0.921168100	2.772337800
H	0.870915700	1.465837900	1.818448000
H	0.381689700	1.537523600	3.537090300
H	1.915248900	0.779115400	3.078947200
C	0.207093400	-1.045356200	-1.776244400
S	1.916436900	-0.775064400	-0.826848000
C	5.022585300	0.158534000	-0.844792600
C	5.287684600	-1.037658100	-0.152500400
C	5.837384500	1.283061500	-0.634499700
C	6.360881200	-1.103602000	0.735448200
H	4.654150600	-1.911533800	-0.311762600
C	6.905210900	1.208743400	0.261497200
H	5.641830900	2.210601500	-1.172893500
C	7.168390000	0.018718200	0.945668000
H	6.565844500	-2.033470700	1.266704300
H	7.537701700	2.082876900	0.419465100
H	8.005457100	-0.035167800	1.642453100
C	3.324023600	1.902174200	-2.464542000
C	2.888704900	2.779778300	-1.453588000
C	3.429003000	2.361379100	-3.786953600
C	2.572791400	4.100662000	-1.764598100
H	2.799356800	2.420347800	-0.426551900
C	3.096673200	3.683220200	-4.092152400
H	3.767868800	1.687993900	-4.574613500
C	2.669387900	4.552991600	-3.085753600
H	2.245322500	4.779391400	-0.976334900
H	3.180366900	4.035455400	-5.120744900
H	2.414645400	5.584997500	-3.328519600
C	4.147964800	-0.712225300	-3.497446400
C	5.488727300	-1.077151200	-3.705763200
C	3.173506300	-1.047644300	-4.454507900
C	5.848060300	-1.764473900	-4.866237900
H	6.247583000	-0.824625800	-2.964696400
C	3.545331600	-1.725866100	-5.614673800
H	2.123587900	-0.811559600	-4.273845300
C	4.880661600	-2.085171100	-5.822697300
H	6.889496900	-2.046459600	-5.024493100
H	2.787141500	-1.984273200	-6.354788000
H	5.166731400	-2.621262700	-6.728150700
N	0.335266800	-1.915168300	-2.928893600
C	-0.973763600	-2.339817000	-3.454000600
H	-1.655928300	-1.487894300	-3.542991100
H	-0.826017600	-2.781135400	-4.448672900
H	-1.443466900	-3.100855400	-2.799807100
C	1.200147100	-3.089538100	-2.790501900
H	0.801636400	-3.828468000	-2.067886400
H	1.272094900	-3.571479700	-3.774272400
H	2.204450600	-2.794901600	-2.474537700
N	-0.456078700	0.157302000	-2.135542200
C	-0.124157600	0.724910600	-3.452789500
H	-0.828858400	1.542544500	-3.654769800
H	0.898004000	1.147229800	-3.463450600
H	-0.207018000	-0.015272400	-4.250841200
C	-0.493488000	1.249388000	-1.162984900
H	0.417450000	1.869610300	-1.233890800
H	-1.365304100	1.887651100	-1.365970500
H	-0.563156500	0.879907700	-0.141390000

S5. References

(1) Weigand, J. J.; Feldmann, K.-O.; Henne, F. D. Carbene-stabilized phosphorus(III)-centered cations LPX_2^+ and L_2PX^{2+} (L = NHC; X = Cl, CN, N_3). *J. Am. Chem. Soc.* **2010**, *132* (46), 16321–16323. DOI: 10.1021/ja106172d.

(2) Schwedtmann, K.; Haberstroh, J.; Roediger, S.; Bauzá, A.; Frontera, A.; Hennersdorf, F.; Weigand, J. J. Formation of an imidazoliumyl-substituted $(L_C)_4P_4^{4+}$ tetracation and transition metal mediated fragmentation and insertion reaction (L_C = NHC). *Chem. Sci.* **2019**, *10* (28), 6868–6875. DOI: 10.1039/c9sc01701a.

(3) Yam, M.; Chong, J. H.; Tsang, C.-W.; Patrick, B. O.; Lam, A. E.; Gates, D. P. Scope and limitations of the base-catalyzed phospha-peterson P=C bond-forming reaction. *Inorg. Chem.* **2006**, *45* (13), 5225–5234. DOI: 10.1021/ic060236p.

(4) Wang, S.; Samedov, K.; Serin, S. C.; Gates, D. P. PhP=CPh_2 and Related Phosphaalkenes: A Solution Equilibrium between a Phosphaalkene and a 1,2-Diphosphetane. *Eur. J. Inorg. Chem.* **2016**, *2016* (26), 4144–4151. DOI: 10.1002/ejic.201600599.

(5) Pedersen, B. S.; Scheibye, S.; Clausen, K.; Lawesson, S.-O. Studies on organophosphorus compounds XXII The dimer of p-methoxyphenylthionophosphine sulfide as thiation reagent. a new route to O-substituted thioesters and dithioesters. *Bull. Soc. Chim. Belges* **1978**, *87* (4), 293–297. DOI: 10.1002/bscb.19780870407.

(6) Polshettiwar, V.; Kaushik, M. P. A new, efficient and simple method for the thionation of ketones to thioketones using P_4S_{10}/Al_2O_3. *Tetrahedron Lett.* **2004**, *45* (33), 6255–6257. DOI: 10.1016/j.tetlet.2004.06.091.

(7) Polshettiwar, V.; Nivsarkar, M.; Paradashani, D.; Kaushik, M. P. Thionation of carbonyl compounds using phosphorus pentasulfide and hexamethyldisiloxane under microwave irradiations. *J. Chem. Res.* **2004**, *2004* (7), 474–476. DOI: 10.3184/0308234042037149.

(8) Kuhn, N.; Kratz, T. Synthesis of Imidazol-2-ylidenes by Reduction of Imidazole-2(3 H)-thiones. *Synthesis* **1993**, *1993* (06), 561–562. DOI: 10.1055/s-1993-25902.

(9) Angelici, R. J., Ed. *Inorganic syntheses*:*Volume 28: Reagents for transition metal complex and organometallic syntheses*; Inorganic syntheses; J. Wiley & Sons, 1990. DOI: 10.1002/9780470132593.

(10) Kerr, W. J.; Lindsay, D. M.; Patel, V. K.; Rajamanickam, M. Efficient methods for enol phosphate synthesis using carbon-centred magnesium bases. *Organic & biomolecular chemistry* **2015**, 13 (40), 10131–10135. DOI: 10.1039/c5ob01849h.

(11) Penafiel, J.; Maron, L.; Harder, S. Early main group metal catalysis: how important is the metal? *Angew. Chem. Int. Ed.* **2015**, 54 (1), 201–206. DOI: 10.1002/anie.201408814.

(12) Henne, F. D.; Schnöckelborg, E.-M.; Feldmann, K.-O.; Grunenberg, J.; Wolf, R.; Weigand, J. J. Observation of a Chloride-Bridged P–P Bond in the Phosphorus Cation $[L(Cl)P(\mu\text{-}Cl)P(Cl)L]^+$ (L = NHC). *Organometallics* **2013**, *32* (22), 6674–6680. DOI: 10.1021/om4002268.

(13) a) Appel, R.; Schöler, H. Notiz über ein bequemes Verfahren zur Darstellung von Dichlorphosphoranen. *Chem. Ber.* **1977**, *110* (6), 2382–2384. DOI: 10.1002/cber.19771100634; b) Godfrey, S. M.; McAuliffeRobin G.; Pritchard, C. A.; Pritchard, R. G.; Sheffield, J. M.; Thompson, G. M. Structure of R_3PCl_2 compounds in the solid state and in solution: dependency of structure on R. Crystal structures of trigonal bipyramidal $(C_6F_5)_3PCl_2$, $Ph_2(C_6F_5)PCl_2$ and of ionic Prn_3PCl_2. *J. Chem. Soc., Dalton Trans.* **1997** (24), 4823–4828. DOI: 10.1039/A705201D;

(14) a) Binder, J. F.; Swidan, A.; Macdonald, C. L. B. Synthesis of Heteroleptic Phosphorus(I) Cations by P^+ Transfer. *Inorg. Chem.* **2018**, *57* (18), 11717–11725. DOI: 10.1021/acs.inorgchem.8b01822; b) Ellis, B. D.; Dyker, C. A.; Decken, A.; Macdonald, C. L. B. The synthesis, characterisation and electronic structure of N-heterocyclic carbene adducts of P(I) cations. *Chem. Comm.* **2005** (15), 1965–1967. DOI: 10.1039/b500692a; c) Schmidpeter, A.; Lochschmidt, S.; Sheldrick, W. S. A diphos-Complex of P⊕: The 1,1,3,3-Tetraphenyl-$1\lambda^5,2\lambda^3,3\lambda^5$-triphospholenyl Cation. *Angew. Chem. Int. Ed. Engl.* **1982**, *21* (1), 63–64. DOI: 10.1002/anie.198200631;

(15) a) Bauer, S.; Marinetti, A.; Mathey, F. Two new syntheses of the "phospha-wittig" reagents. *Heteroat. Chem.* **1991**, *2* (2), 277–281. DOI: 10.1002/hc.520020209; b) Marinetti, A.; Bauer, S.; Ricard, L.; Mathey, F. The "phospha-Wittig" reaction: a new method for building phosphorus-carbon double and single bonds from carbonyl compounds. *Organometallics* **1990**, *9* (3), 793–798. DOI: 10.1021/om00117a040; c) Shah, S.; Protasiewicz, J. D. 'Phospha-Wittig' reactions using isolable phosphoranylidenephosphines ArP=PR_3 (Ar = 2,6-$Mes_2C_6H_3$ or 2,4,6-$tBu_3C_6H_2$). *Chem. Commun.* **1998** (15), 1585–1586. DOI: 10.1039/a802722f;

(16) Flörke, U.; Ahmida, A.; Egold, H.; Henkel, G. Crystal structure of di-chlorido-bis-(1,3-diisopropyl-4,5-dimethyl-2H-imidazole-2-thione-κS)zinc(II). Acta crystallographica. Section E, Structure reports online 2014, 70 (Pt 11), m384. DOI: 10.1107/S1600536814023642.

(17) Klebach, T. C.; Lourens, R.; Bickelhaupt, F. Synthesis of Mesityldiphenylmethylenephosphine: a stable compound with a localized P=C bond. *J. Am. Chem. Soc.* **1978**, *100* (15), 4886–4888. DOI: 10.1021/ja00483a041.

(18) Wong, Y. O.; Freeman, L. A.; Agakidou, A. D.; Dickie, D. A.; Webster, C. E.; Gilliard, R. J. Two Carbenes versus One in Magnesium Chemistry: Synthesis of Terminal Dihalide, Dialkyl, and Grignard Reagents. *Organometallics* **2019**, 38 (3), 688–696. DOI: 10.1021/acs.organomet.8b00866.

(19) Oxford Diffraction/Agilent Technologies UK Ltd. *CrysAlisPRO*, 2016.

(20) Dolomanov, O. V.; Bourhis, L. J.; Gildea, R. J.; Howard, J. A. K.; Puschmann, H. OLEX2 : a complete structure solution, refinement and analysis program. *J Appl Crystallogr* **2009**, *42* (2), 339–341. DOI: 10.1107/S0021889808042726.
(21) Sheldrick, G. M. SHELXT - integrated space-group and crystal-structure determination. *Acta crystallographica. Section A, Foundations and advances* **2015**, *71* (Pt 1), 3–8. DOI: 10.1107/S2053273314026370.
(22) Sheldrick, G. M. Crystal structure refinement with SHELXL. *Acta crystallographica. Section C, Structural chemistry* **2015**, *71* (Pt 1), 3–8. DOI: 10.1107/S2053229614024218.
(23) Spek, A. L. Single-crystal structure validation with the program PLATON. *J Appl Crystallogr* **2003**, *36* (1), 7–13. DOI: 10.1107/S0021889802022112.
(24) Ahlrichs, R.; Bär, M.; Hacer, M.; Horn H.; Kömel, C. Electronic structure calculations on workstation computers: The program system turbomole *Chem. Phys. Lett.* **1989**, 162, 165–169. DOI 10.1016/0009-2614(89)85118-8.
(25) (a) Perdew, J. P. Density-functional approximation for the correlation energy of the inhomogeneous electron gas *Phys. Rev. B*, **1986**, 33, 8822–8824. DOI 10.1103/PhysRevB.33.8822; (b) Becke, A. D. Density-functional thermochemistry. IV. A new dynamical correlation functional and implications for exact-exchange mixing *J. Chem. Phys.* **1996**, 104, 1040-1046. DOI 10.1063/1.470829.
(26) Grimme, S.; Antony, J.; Ehrlich, S.; Krieg, H. A consistent and accurate ab initio parametrization of density functional dispersion correction (DFT-D) for the 94 elements H-Pu *J. Chem. Phys.* **2010**, 132, 154104–154119. DOI 10.1063/1.3382344.
(27) Klampt, A. The COSMO and COSMO-RS solvation models WIREs *Comput. Mol. Sci.* **2011**, 1, 699–709. DOI 10.1002/wcms.56.
(28) Neese, F. Wennmohs, F.; Becker, U.; Riplinger, C. The ORCA quantum chemistry program package *J. Chem. Phys.* **2020**, 152, 224108. DOI 10.1063/5.0004608.
(29) Cheeseman, J. R. Trucks, G. W. Keith, T. A. Frisch, M. J. A comparison of models for calculating nuclear magnetic resonance shielding tensors *J. Chem. Phys.* **1996**, 104, 5497–509. DOI 10.1063/1.471789.
(30) Takano, Y.; Houk K. N. Benchmarking the Conductor-like Polarizable Continuum Model (CPCM) for Aqueous Solvation Free Energies of Neutral and Ionic Organic Molecules *J. Chem. Theory Comput.* **2005**, 1, 70–77. DOI 10.1021/ct049977a.
